# cg04448376, cg24387542, cg08548498, and cg14621323 as a Novel Signature to Predict Prognosis in Kidney Renal Papillary Cell Carcinoma

**DOI:** 10.1155/2020/4854390

**Published:** 2020-12-17

**Authors:** Ying-Lei Wang, Ying-Ying Zhang

**Affiliations:** ^1^Department of Urinary Surgery, Yantai Affiliated Hospital of Binzhou Medical University, Yantai, Shandong 264100, China; ^2^Out-patient Department, Yantai Affiliated Hospital of Binzhou Medical University, Yantai, Shandong 264100, China

## Abstract

**Introduction:**

DNA methylation plays a vital role in prognosis prediction of cancers. In this study, we aimed to identify novel DNA methylation site biomarkers and create an efficient methylated site model for predicting survival in kidney renal papillary cell carcinoma (KIRP).

**Methods:**

DNA methylation and gene expression profile data were downloaded from The Cancer Genome Atlas (TCGA) database and the Gene Expression Omnibus (GEO) database. Differential methylated genes (DMGs) and differential expression genes (DEGs) were identified and then searched for the hub genes. Cox proportional hazards regression was applied to identify DNA methylated site biomarkers from the hub genes. Kaplan–Meier survival and ROC analyses were used to validate the effective prognostic ability of the methylation gene site biomarker. The biomarker sites were validated in the GEO cohorts. The GO and KEGG annotation was done to explore the biological function of DNA methylated site signature.

**Results:**

Nine DMGs with opposite expression patterns containing 47 methylated sites were identified. Finally, four methylated sites were identified using the hazard regression model (cg04448376, cg24387542, cg08548498, and cg14621323) located in UTY, LGALS9B, SLPI, and PFN3, respectively. These sites classified patients into high- and low-risk groups in the training cohort. The 5-year survival rates for patients with low-risk and high-risk scores were 97.5% and 75.9% (*P* < 0.001). The prognostic accuracy and signature methylation sites were validated in the test (TCGA, *n* = 87) and GEO cohorts (*n* = 14). Multivariate regression analysis showed that the signature was an independent prediction prognostic factor for KIRP. Based on this analysis, we developed methylated site signature nomogram that predicts an individual's risk of survival. Functional analysis suggested that these signature genes are involved in the biological processes of protein binding.

**Conclusions:**

Our study demonstrated that the methylated gene site signature might be a powerful prognostic tool for evaluating survival rate and guiding tailored therapy for KIRP patients.

## 1. Introduction

Renal carcinoma is a heterogeneous tumor, of which epithelial renal cell carcinoma (RCC) accounts for the most cases. There are over ten recognized histological subtypes of RCC, of which chromophobe renal cell carcinoma (ChRCC), kidney renal papillary cell carcinoma (KIRP), and kidney renal clear cell carcinoma (KIRC) are the three most common subtypes. Among these subtypes, KIRP has the second-highest morbidity rate of 10%–15%, while KIRC has the highest incidence of 75%–80% [1–5]. About 30% of RCC patients present with distant metastasis at diagnosis and have a poor prognosis. In clinical studies, patients with KIRC and ChRCC often have ideal results. However, patients who were diagnosed with KIRP have worse clinical outcomes [6–8]. Researchers have a relatively good understanding of KIRC pathogenesis compared to KIRP, as most of the RCC presented is of KIRC subtype [9, 10]. Many therapies for advanced RCC are based on blocking known KIRC pathways using mTOR inhibitors and tyrosine kinase inhibitors to regulate the HIF1*α* and VEGF pathways; however, there are very few treatments for KIRP.

KIRP is a renal parenchyma malignant tumor, including two different subtypes (type 1 and type 2), often observed with a papillary or tubulopapillary architecture [11]. Like the KIRC, VEGF inhibitors and mTOR inhibitors have been developed based on the understanding of specific molecular sites [12]. Although researchers are beginning to develop therapeutic targets for KIRP, such as foretinib and cabozantinib, these drugs are specific for type 1 KIRP but not the more aggressive type 2 KIRP [13, 14]. Therefore, we must identify new and powerful molecular markers for prediction and treatment sites, which can help develop new targeted drugs specific for KIRP.

Although cancer occurrence and development mainly depend on the alteration of tumor-associated genes, epigenetic changes such as the DNA methylation of tumor-related genes play an essential role in the molecular barrier against tumor development [15, 16]. DNA methylation is often considered a mechanism of gene silencing, and it functions directly in many cellular processes such as embryonic development, transcription, genomic imprinting, and X chromosome inactivation [17–19]. DNA methylation signatures have already been used in the early diagnosis and prognosis of cancers. For example, in breast cancer, the poor prognosis in patients may be correlated with CDH1 promoter methylation [20]. Also, the DNA methylation of the promoter regions of P16, CDH13, APC, and RUSSF1A in stage I patients with non-small-cell lung cancer may be associated with early recurrence [21, 22].

There are also a large number of methylation biomarkers that have been proposed for the prediction of RCC [23], including the single methylation biomarkers for prognosis such as CRHBP [24], RCVRN [25], AR [7], CDO1 [26], BMP-2 [27], KEAP1 [5], and DAB2IP [28]. While promising, tumorigenesis is a complex process that requires the involvement of multiple genes; thus, many of these biomarkers are imperfect [29]. Considering that the occurrence and development of KIRP is a complex process that requires the joint regulation of multiple omics, it is necessary to establish a molecular marker model with high sensitivity and strong predictive ability to elucidate the prognosis of KIRP.

In this study, we aimed to find potential survival-related DNA methylation site signatures in KIRP, which may pave the way for the development of novel prognostic markers and therapeutic targets for KIRP.

## 2. Materials and Methods

### 2.1. DNA Methylation Profiling and Gene Expression Datasets of KIRP Patients

**Figure 1:**
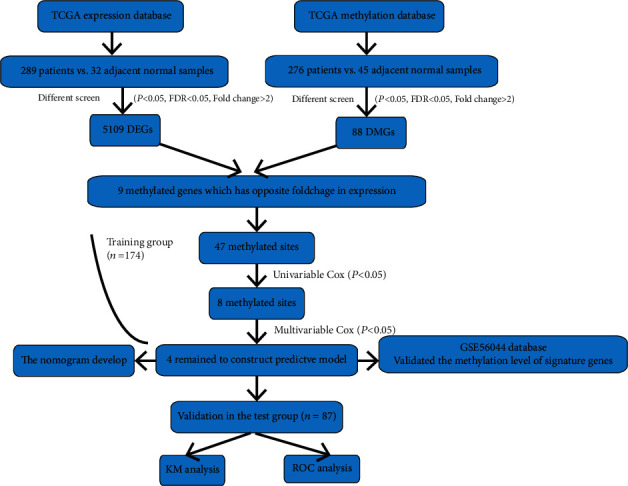
Flowchart of the study.

In the current study, the DNA methylation profiling (Illumina Human Methylation 450K Bead Chip Array) and gene expression datasets (Illumina HiSeq RNA Seq V2) were downloaded from TCGA database. A total of 276 KIRP and 45 control specimens were enrolled in the methylation dataset, while there were 289 cases and 32 controls in the gene expression dataset. Both datasets contain clinical data, including survival time, status, gender, age, and clinical stage. The clinical information of methylation data is shown in Table 1. Other DNA methylation data were retrieved from the Gene Expression Omnibus (GEO) database (GSE126441, *n* = 14), which were used to validate the methylated level of signature genes. To improve the data accuracy, we preprocessed both datasets, including removing the sites in which 70% of the methylated level were NA, and genes with missing expression values in >30% of the patients. Genes with RPKM expression values of 0 in all samples were excluded [30]. The technical route to select the DNA methylated site signature is shown in Figure 1.

### 2.2. Identification of DMGs and DEGs Associated with KIRP

To identify the differentially methylated genes (DMGs), we adopted the Benjamini-Hochberg false-discovery rate (FDR) method to adjust the *P* value for each gene. The DMGs were identified by a fold change > 2, *P* value <0.05, FDR < 0.05, and beta value > 0.1. The differentially expressed genes (DEGs) were identified by a fold change > 2, *P* value <0.05, FDR < 0.05, and FPKM > 1.

After identifying multiple DMGs and DEGs from these datasets, we screened nine hub genes, which are both differentially expressed and enriched in differential methylation between the DMGs and DEGs.

### 2.3. Constructing a Prognostic DNA Methylation Signature in the Training Dataset

Statistics were employed to build a model based on reports of a better method to create a signature module [31]. Gene methylation often occurs at specific loci. The methylation of the gene is composed of multiple methylation sites, so to make the detection more accurate, we looked for methylation sites of hub genes, then identified the sites associated with survival. Methylation sites were validated in the GEO dataset. After identifying the methylation sites of hub genes, we randomly divided all the KIRP methylation samples into two groups, the test group (87 cases) and the training group (174 cases). The two groups were separated and uncrossed. Univariate Cox proportional hazards regression analysis was used to identify the association between survival time/status and each methylation site in the training dataset [32]. To screen the most authoritative and accurate DNA methylation sites to predict KIRP prognosis, multivariate Cox regression analysis was used to build a model to assess the prognosis risk according to the following expression:

(1) $$\begin{array}{c} \text{Risk score}\left(\text{RS}\right)=\overset{n}{\underset{i=1}{\sum }}{\text{meth}}_{i}*{\text{Coef}}_{i}, \end{array}$$

where *n* represents the number of prognostic methylation gene sites, meth_*i*_ is the methylation value of the gene sites, and Coef_*i*_ is a single factor Cox regression coefficient. When the coefficient of the Coef_*i*_ < 0, we defined it as a favorable prognosis site, while the sites with the coefficient of Coef_*i*_ > 0 were considered as a poor prognosis site. Risk score (RS) is the multinode weighted sum of risk scores.

### 2.4. Statistical Analysis

The selected methylated sites were used to construct a risk model. KIRP patients were dichotomized into either high-risk or low-risk groups in the training dataset; the median risk score was used as a cutoff value. Kaplan–Meier survival analysis and ROC analysis were used to validate the effective prognostic ability of the methylation gene site signatures. We then confirmed the prognostic ability of the DNA methylation signature in the test dataset. Furthermore, multivariable Cox regression analysis was carried out to identify whether the DNA methylation signature was an independent factor in survival prediction; we considered that *P* < 0.05 indicates a statistically significant difference. All analyses were performed with the R statistical program (version 3.5.1).

### 2.5. Generating the Nomogram

We created a nomogram by using the “RMS” package of R software. The nomogram concordance index (C-index) of all patients was obtained by multivariate Cox regression analysis. The higher the C-index, the more accurate the prediction. The nomogram was used to calculate the total score of each patient. Overall scores were then used to predict 1-year, 3-year, and 5-year survival rates [33].

### 2.6. Functional Annotation of the Selected DNA Methylation Signature Genes

To further study the function of survival-related DNA methylation signature genes, we used Gene Ontology (GO) analysis (http://www.geneontology.org) to investigate the roles of all the selected genes and Kyoto Encyclopedia of Genes and Genomes (KEGG) pathway analysis (http://www.genome.jp/kegg/) to determine the significant pathways. Fisher's exact test and chi-square tests were used to select significant GO and pathway categories, with the threshold of significance of *P* < 0.05.

## 3. Results

### 3.1. Identification of DMGs and DEGs Associated with KIRP

After preprocessing the methylation dataset, 261 cancerous tissues were remaining. We identified the DEGs and DMGs in the two datasets. To determine the KIRP-related DMGs, we performed comparisons between 276 cancerous tissues and 45 adjacent tissues from KIRP patients. A total of 88 DMGs (Table S1) were identified in the methylation dataset (*P* < 0.05; Δ*β* > 0.1). Among these, there were 48 hypomethylated genes and 40 hypermethylated genes. For the DEGs identified, we compared the 289 cases and 32 controls in the gene expression dataset. We obtained 5109 DEGs (Table S2), of which 3076 were upregulated, and 2033 were downregulated. After identifying the DMGs and DEGs, there were nine overlapping genes with hypomethylated-high-expression and hypermethylated-low-expression, which contained seven hypomethylated-high-expression and two hypermethylated-low-expression genes as described in Fig. S1; the nine hub genes are shown in Table S3.

### 3.2. Identifying the Four-DNA Methylation Site Signature in the Training Group

**Figure 2:**
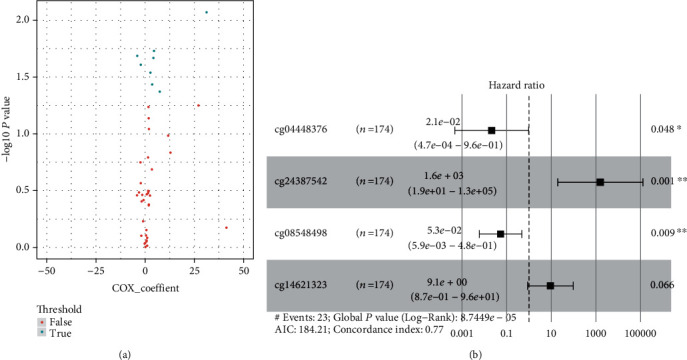
(a) Univariable and (b) multivariable Cox regression analyses of the association between the methylated site signature and the survival of KIRP patients in training group.

The 261 patients were randomly divided into two groups (training group, *n* = 174; test group, *n* = 87) to identify and test the prognostic methylated gene sites found in the KIRP patients. In total, we identified 47 methylation sites (Table S4) in the nine hub genes. Then, univariate Cox proportional hazards regression analysis was conducted in the training group to identify the methylation sites significantly associated with overall survival time from the 47 sites. Eight methylated sites were significantly related to the survival of KIRP patients (*P* < 0.05, Figure 2(a), Table S5). To select the most significant prediction power signature, we conducted a multivariable Cox regression analysis and a model with the four-methylated gene site set (cg04448376, cg24387542, cg08548498, and cg14621323, Figure 2(b)) to assess the prognosis risk that was created. The risk score (RS, Table S6) was determined as follows:

(2) $$\begin{array}{c} \text{RS}=\left(-4.15\times {\text{meth}}_{\text{cg}04448376}\right)+\left(4.58\times {\text{meth}}_{\text{cg}24387542}\right)+\left(-2.37\times {\text{meth}}_{\text{cg}08548498}\right)+\left(2.50\times {\text{meth}}_{\text{cg}14621323}\right). \end{array}$$

RS and meth are the risk score and the methylation value, respectively.

### 3.3. Identification of the Survival Power of the DNA Methylation Signature

**Figure 3:**
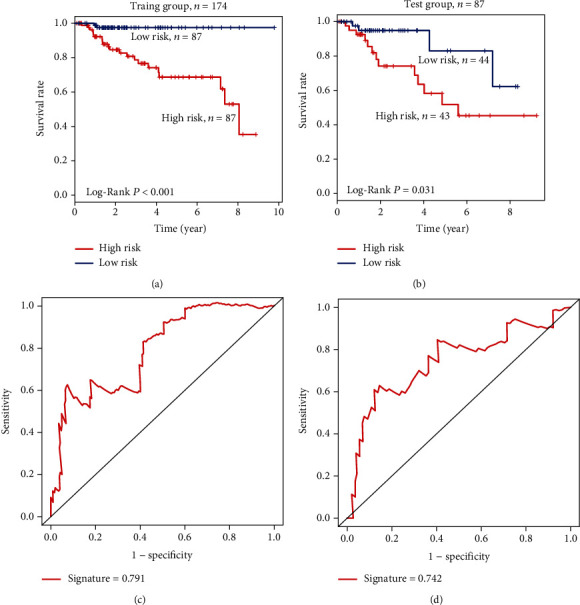
Methylated site signature predicts prognosis of KIRP patients. (a, b) Kaplan–Meier survival curves classified KIRP patients into high-risk and low-risk groups using the site signature in the training and test datasets. *P* values were calculated by log-rank test. (c, d) Results of receiver operating characteristic (ROC) analysis.

Each patient got a risk score from the selected methylated signature, and the median risk score was used as the cutoff to divide the training group patients into either the low-risk group (*n* = 87) or high-risk group (*n* = 87). Kaplan–Meier survival analysis showed that the overall survival (OS) rate of the low-risk group was significantly higher than that of the high-risk group (OS rate: 97.7% vs. 75.9%; log-rank test *P* < 0.001; Figure 3(a)). To validate the prediction power of the DNA methylation signature, we confirmed it in the test group using the same prognostic risk score model. We found significant differences between the high-risk and low-risk groups (Figure 3(b)). In the test dataset, the high-risk group had a significantly lower OS rate than the low-risk group (OS rate: 69.8% vs. 90.9%; log-rank test *P* = 0.032).

### 3.4. The Four-Methylation Site Signature Has Great Survival Predictive Power

To test the DNA methylation signature model's predictive ability, we conducted a time-dependent ROC analysis, which showed a high predictive ability of the four methylation signature sites in the training group (AUC_signature_ = 0.890, Figure 3(c)). It further indicates that the signature in our study is a new, highly accurate prognostic prediction marker. Similar results were found in the test group (AUC_signature_ = 0.900, Figure 3(d)). The sensitivity and specificity of prognostic prediction are higher than the stage (Figures 3(c) and 3(d)).

### 3.5. Nomogram of Combined Methylated Site Signature and Clinical Variables Predicts Patient's OS

**Figure 4:**
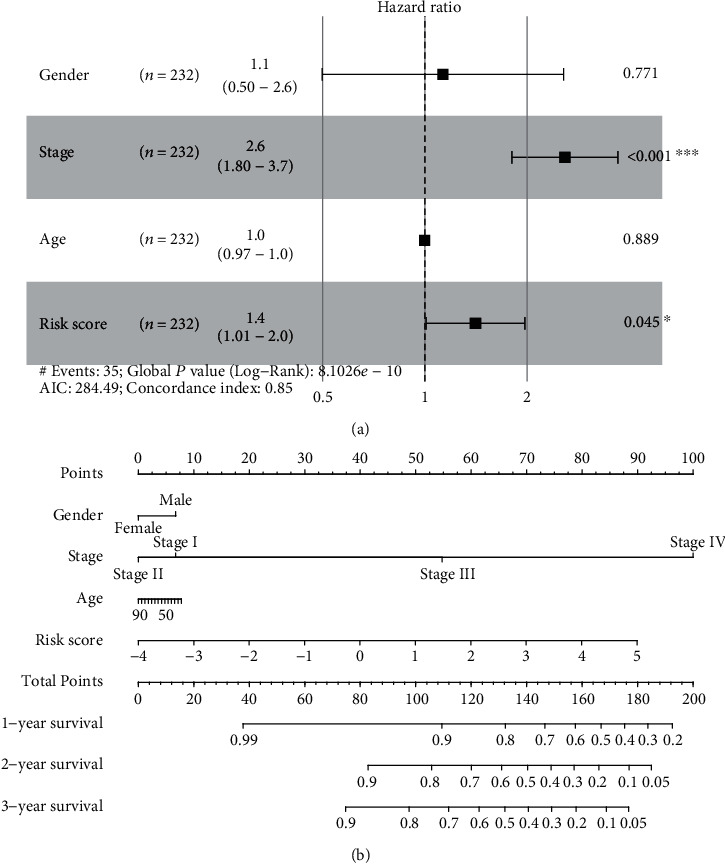
(a) Multivariable Cox regression analysis and (b) nomogram of combined methylated site signature and clinical variables predict patients' OS.

Our multivariate Cox regression model demonstrated that the signature risk score's predictive power was independent of clinical characters (high-risk group vs. low-risk group: HR = 1.40, 95% CI: 1.01–2.00, and *P* = 0.045; Figure 4(a)). According to the above analysis results, we developed a methylated gene site nomogram, which combined the clinical-related factors (stage) and methylated gene site signature. In the training group, the calibration chart of the five-year operating system is well predicted (Figure 4(b)). We have compared C-index of this risk score to the cluster of cluster signature of that in the previous study in KIRP dataset (PMID: 26536169). The results showed that our risk score was better than clusters in KIRP dataset (PMID: 26536169) in Table 2.

### 3.6. Validated Methylation Sites in Independent GEO Cohorts

**Figure 5:**
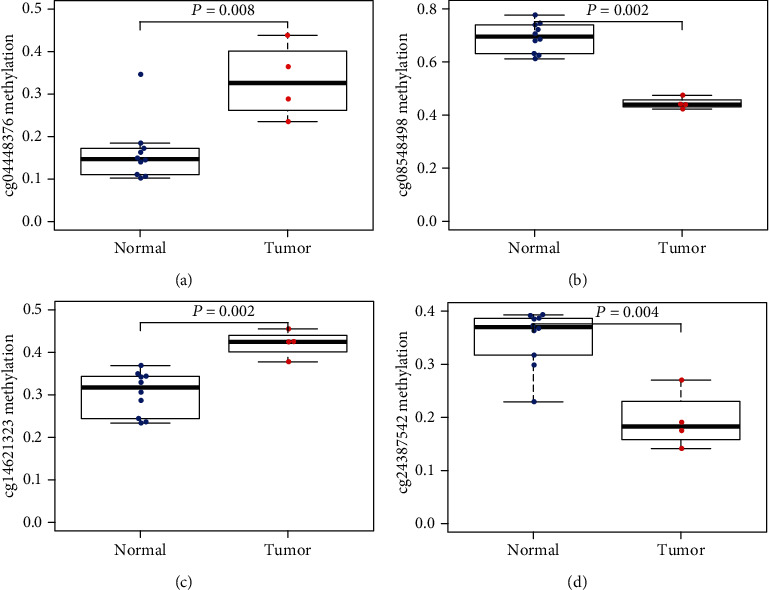
Validated methylation sites of signature in independent GEO cohorts.

To confirm the four-methylation site pattern in different populations, we evaluated the samples (4 patients vs. 10 normal) in GSE126441 (Table S7). It showed that cg04448376 and cg14621323 were downregulated, and cg08548498 and cg24387542 were upregulated in patients vs. normal (Figure 5), which is the same methylation pattern in TCGA dataset. GO and KEGG functional annotation showed that the survival-related DNA methylation signature genes were significantly enriched in only three different GO terms (*P* < 0.05). The four genes were mainly enriched in protein binding, an integral component of membrane and cytoplasm (Table S8). The four-methylated site signature may participate in tumorigenesis by regulating cellular metabolic processes.

## 4. Discussion

KIRP remains a clinical challenge due to high histologic heterogeneity, poor prognosis, and limited treatment options. KIRP is the second most prevalent phenotype of RCC [34]; however, the carcinogenesis mechanism of KIRP is not fully understood. Much of the previous research on KIRP genes has focused on some known cancer-related genes of KIRC. There is a lack of epigenetic biomarkers, and most of the prognosis of KIRP biomarker research is focused on the mRNA, lncRNA, and miRNA. For instance, Lan et al. identified seven lncRNAs that could predict the prognosis of KIRP [8]; Luo et al. identified hsa-mir-3199-2 and hsa-mir-1293 as novel prognostic biomarkers for KIRP [35]; and Gao et al. [36] found that five mRNAs (CCNB2, IGF2BP3, KIF18A, PTTG1, and BUB1) can predict KIRP patient survival. Although some prognostic markers of KIRP have been found in previous studies, the results are not consistent, and there is no analysis at multiple omics levels. Therefore, reliable molecular signatures are needed to predict the survival of KIRP patients.

Our study identified 47 methylated gene sites from nine differentially methylated genes (DMGs), with opposite differential expression patterns. We used various statistical approaches to identify four-methylated site signature from the 47 methylated sites. The signature that we selected can separate the KIRP patients into high-risk and low-risk groups with significantly different survival times in the training and test datasets, indicating that it has a powerful prediction ability. The independence of the selected DNA methylation gene signature in predicting OS in the entire dataset was identified using multivariable Cox regression analysis, which confirmed that the risk score of DNA methylation site signature maintained an independent related to OS.

The ROC curve showed that the AUC is 0.791 in the training group and 0.742 in the test group. Considering that larger AUC usually indicates better prediction power, this result further demonstrated that the DNA methylation signature in our study is a high accuracy novel prognostic marker and has significant clinical value. Also, our signature of DNA methylation sites did not depend on other clinical features. Moreover, we validated the signature of methylation gene sites and methylation sites in TCGA and GEO cohorts and demonstrated their ability to predict the overall survival of KIRP patients. We also established a methylation gene site nomogram, including methylation gene site signature and clinical-related risk factors (e.g., stage and age) to predict OS. Our study results help in understanding the development of KIRP and for developing tailored therapy and ultimately may contribute to an increase in survival rates of KIRP patients.

In addition, we analyzed the function of the selected DNA methylation genes. The four methylation sites cg04448376, cg24387542, cg08548498, and cg14621323 were located in UTY, LGALS9B, SLPI, and PFN3, respectively. SLPI is a gene-encoding secretory leukocyte protease inhibitor, 11.7 kDa serine protease inhibitor, and is a member of the whey acidic protein four-disulfide core family [37, 38]. SLPI can reduce the activities of trypsin, neutrophil elastase, chymotrypsin, and cathepsin G [39]. Therefore, SLPI may be a potential tumor marker to predict the prognosis [39]. Previous studies have shown that SLPI is related to tumor metastasis. In some high-risk, aggressive or metastatic tumors, such as the pancreatic, uterine cervix, papillary thyroid, and ovarian cancers, SLPI was often found to be highly expressed [40, 41]. However, in bladder tumors, nasopharyngeal carcinoma, and some breast cancers, SLPI has a low expression. SLPI has high expression in gastric cancer cells with serosa invasion, and SLPI overexpression in gastric cancer cell lines can improve the cell migration and invasion rate [38]. These observations are entirely consistent with the previous view that the expression of SLPI in tumors is often associated with poor prognosis. As far as we know, the present study is the first report of SLPI methylation sites as a prognostic biomarker in human KIRP. SLPI is hypomethylated and overexpressed in this report, just like the previous study demonstrated, so we believe that SPLI is a favorable biomarker for prognosis.

UTY is located on the Y chromosome and can encode a demethylase and was reported to be an epigenetic-related gene. UTY is essential in the development of teratoma through the regulation of epigenetic changes [42, 43]. In urothelial bladder cancer (UBC), 22.8% (8/35) of patients were found to have a reduced UTY copy number, and cell proliferation was found to increase in a UTY knockout. UTY also plays a vital role in some regulatory pathways, such as the NF-B and p53 pathways [44, 45]. In this study, we showed that methylation of UTY was significantly associated with KIRP survival and that UTY can act as a survival-related methylation biomarker for KIRP.

For LGALS9B and PFN3, there is very little known about their regulatory mechanisms. The LGALS9B gene was initially thought to represent a pseudogene of galectin 9; however, the association of LGALS9B gene and tumors is unclear. This gene is one of two similar loci on chromosome 17p identical to galectin 9 and is now thought to be a protein-encoding gene. We have found that its functions are primarily associated with protein binding. Thus, we suspect that it is similar in function to galectin 9. Galectin-9 was reported to be related to different aspects of tumor growth, metastasis, immunosuppression, and immunomodulation [46, 47]. In breast carcinoma, liver cancer, and cervical tumors, LGALS9 expression affects disease prognosis [48–51]. PFN3, one of the isoforms of profilin, is an actin-binding protein. Previous studies show that PFN3 is expressed in the brain, testis, and kidney [52]. Genetic variation of PFN3 is significantly related to nephrolithiasis of Japanese individuals [53]. Although the functions of LGALS9B and PFN3 are unclear, they are significantly associated with KIRP survival. Our study indicates that UTY, LGALS9B, SLPI, and PFN3 have essential roles in KIRP.

The limitations of this study need to be recognized. First, the samples of our study are entirely retrospective, and inherent biases may influence the results. Hence, we may have lost signatures that are potentially correlated with KIRP survival. Secondly, we have not further searched the mechanism of action of these DNA methylation genes in KIRP. Finally, although we identified the selected DNA methylation sites as a powerful prediction signature, applying it in a clinical setting will require more research.

## 5. Conclusions

Taken all together, by performing a comprehensive analysis for DNA methylation data, gene expressed profiles, and corresponding clinical information, our study demonstrated that the four-methylated site signature was a potential prognosis marker for KIRP, the significant and consistent correlation between our four-methylated site signature, and overall survival in two independent datasets which indicated that it is a potentially powerful prognostic marker for KIRP. In summary, we identified a novel methylated site signature to predict prognosis in KIRP. We confirmed that this signature could serve as a potentially robust and specific biomarker in the prognosis prediction and tailored therapy for KIRP patients.

## Figures and Tables

**Table 1:** Summary of patient demographics and characteristics.

Characteristic	Training (*n* = 174)	Test (*n* = 87)
Gender		
Female	43	26
Male	131	56
Age		
<62 years	78	41
≥62 years	96	46
Stage		
I	107	52
II	15	6
III	35	18
IV	8	5
Vital status		
Living	151	70
Dead	23	17

**Table 2:** Comparison of the four-methylated site prognostic signature to the published KIRP prognostic signature.

Studies	HR (95% CI)	*P*	C-index
Present study, 4-methylated site signature	3.80 (2.2-6.3)	8.102*e*-10	0.85
PMID: 26536169, the cluster signature	2.25 (1.48-3.98)	1.48*e*-03	0.613

## Data Availability

All data generated or analyzed during this study are included in this published article.

## Supplementary Materials

Supplementary Materials Table S1: DMGs. Table S2: DEGs. Table S3: the 9 hub genes. Table S4: the methylated sites of 9 hub genes. Table S5: univariate Cox proportional hazards regression analysis (*P* < 0.05) of the methylated site data in the training dataset. Table S6: the signature risk score composed of 4 site combinations in the training and test dataset. Table S7: the expression of 4 methylated sites in GSE126441. Table S8: functional analysis of the selected 9 hub genes. Fig.S1: identification of the hub genes from DMGs and DEGs. The Venn diagram shows that there are nine hub genes in 79 DMGs and 5100 DEGs. The hub genes are opposite fold change.
