# Supplementary material for: cg04448376, cg24387542, cg08548498, and cg14621323 as a Novel Signature to Predict Prognosis in Kidney Renal Papillary Cell Carcinoma
Source: Biomed Res Int. 2020 Dec 17;2020:4854390. doi: 10.1155/2020/4854390 (PMC7759405; doi:10.1155/2020/4854390)
Supplement: Supplementary Materials — Table S1: DMGs. Table S2: DEGs. Table S3: the 9 hub genes. Table S4: the methylated sites of 9 hub genes. Table S5: univariate Cox proportional hazards regression analysis (P < 0.05) of the methylated site data in the training dataset. Table S6: the signature risk score composed of 4 site combinations in the training and test dataset. Table S7: the expression of 4 methylated sites in GSE126441. Table S8: functional analysis of the selected 9 hub genes. Fig.S1: identification of the hub genes from DMGs and DEGs. The Venn diagram shows that there are nine hub genes in 79 DMGs and 5100 DEGs. The hub genes are opposite fold change. [file 4854390.f1.zip › Table S2.docx]

| **Table S2 DEGs** | | | | |
| --- | --- | --- | --- | --- |
| id | logFC | logCPM | PValue | FDR |
| MFSD4A | -4.67582 | 5.639767 | 5.90E-275 | 1.06E-270 |
| UMOD | -12.2852 | 10.00104 | 5.78E-256 | 5.17E-252 |
| CALB1 | -8.29198 | 5.463279 | 6.72E-219 | 4.01E-215 |
| EGF | -6.17655 | 5.460107 | 1.34E-207 | 5.98E-204 |
| GP2 | -8.1869 | 1.034802 | 5.20E-201 | 1.86E-197 |
| DDN | -7.01256 | 2.02265 | 2.82E-192 | 8.41E-189 |
| CRHBP | -6.0743 | 2.316755 | 8.56E-190 | 2.19E-186 |
| GGACT | -3.55088 | 3.554442 | 1.27E-189 | 2.83E-186 |
| PTGER1 | -5.36213 | 0.851526 | 8.36E-158 | 1.66E-154 |
| SLC26A4 | -5.16544 | 1.727284 | 6.98E-152 | 1.25E-148 |
| EMCN | -3.97017 | 4.321427 | 3.78E-150 | 6.15E-147 |
| ATP12A | -7.86238 | 1.047269 | 6.05E-146 | 9.03E-143 |
| SLC34A3 | -5.59876 | 2.249827 | 3.21E-140 | 4.43E-137 |
| ACPP | -5.53666 | 2.648526 | 2.68E-137 | 3.36E-134 |
| OLFM3 | -6.96171 | -2.42697 | 2.81E-137 | 3.36E-134 |
| HCRTR2 | -6.62258 | -3.2585 | 2.17E-135 | 2.43E-132 |
| MYLK3 | -4.00907 | 0.55018 | 1.11E-131 | 1.17E-128 |
| SEMA3G | -3.70794 | 3.993717 | 5.29E-130 | 5.26E-127 |
| PODXL | -4.11025 | 6.804422 | 1.04E-128 | 9.84E-126 |
| RNF212B | -4.334 | -0.18041 | 2.14E-126 | 1.92E-123 |
| TEK | -3.94263 | 3.621885 | 3.10E-126 | 2.64E-123 |
| RASL11B | -5.44586 | 2.072956 | 9.97E-123 | 8.11E-120 |
| CLIC5 | -4.97789 | 4.498405 | 5.49E-121 | 4.27E-118 |
| ESRRB | -5.91416 | 2.658196 | 1.09E-120 | 8.11E-118 |
| ST6GALNAC3 | -3.81277 | 1.777557 | 2.70E-120 | 1.93E-117 |
| SPTB | -3.20335 | 1.36552 | 3.43E-120 | 2.36E-117 |
| RANBP3L | -5.66858 | 2.16885 | 1.91E-119 | 1.27E-116 |
| PROX1 | -4.85096 | 2.142932 | 4.21E-119 | 2.69E-116 |
| CPNE6 | -8.17201 | 0.993286 | 2.31E-117 | 1.42E-114 |
| AFM | -7.59594 | 2.105522 | 1.09E-116 | 6.53E-114 |
| SLC14A2 | -7.19604 | 1.869613 | 1.35E-115 | 7.82E-113 |
| WNT8B | -4.38258 | -2.01679 | 1.81E-112 | 1.01E-109 |
| MUC6 | -4.5955 | 0.147388 | 3.95E-110 | 2.14E-107 |
| GPR182 | -4.06894 | -1.09364 | 5.06E-109 | 2.66E-106 |
| WNT9B | -5.72144 | -0.02236 | 8.34E-109 | 4.26E-106 |
| MRPS6 | -2.19371 | 5.175289 | 5.39E-107 | 2.68E-104 |
| CLCNKA | -5.81035 | 4.868823 | 1.45E-106 | 7.04E-104 |
| SIAH3 | -5.00061 | -0.49161 | 1.50E-106 | 7.08E-104 |
| FLRT1 | -4.57533 | 0.621845 | 4.70E-105 | 2.16E-102 |
| CCNI2 | -3.2194 | -0.27107 | 2.53E-104 | 1.13E-101 |
| WNK4 | -4.2995 | 4.081771 | 2.61E-103 | 1.14E-100 |
| ALB | -6.54733 | 4.813713 | 7.86E-103 | 3.35E-100 |
| KCNJ10 | -5.8515 | 3.867576 | 9.00E-103 | 3.75E-100 |
| SLC5A3 | -2.78835 | 5.814037 | 4.08E-100 | 1.66E-97 |
| SOST | -7.91853 | 1.221549 | 4.22E-100 | 1.68E-97 |
| SLC9A3 | -4.51031 | 4.663697 | 1.05E-99 | 4.09E-97 |
| ATP6V1C2 | -3.69202 | 2.361721 | 1.02E-96 | 3.88E-94 |
| CST9 | -6.89447 | -3.13513 | 5.19E-96 | 1.93E-93 |
| LINC00675 | -6.2148 | 0.050742 | 1.54E-95 | 5.63E-93 |
| RERGL | -4.30352 | 0.301243 | 1.78E-94 | 6.36E-92 |
| TMEM178A | -3.45633 | 2.413057 | 2.79E-94 | 9.80E-92 |
| INSRR | -6.89981 | 0.292226 | 6.87E-93 | 2.36E-90 |
| CRYAA | -6.46431 | -3.44404 | 7.49E-93 | 2.53E-90 |
| RBP2 | -3.99824 | -1.97081 | 8.91E-93 | 2.95E-90 |
| FAM151A | -5.58249 | 4.751899 | 5.27E-92 | 1.72E-89 |
| UNCX | -7.31546 | -2.09749 | 9.67E-89 | 3.09E-86 |
| GRM1 | -4.45953 | 0.067537 | 2.19E-88 | 6.89E-86 |
| HSPA2 | -3.53465 | 5.341414 | 1.08E-87 | 3.34E-85 |
| TMEM174 | -5.30537 | 3.887037 | 6.73E-87 | 2.04E-84 |
| SFXN2 | -2.61421 | 4.650619 | 1.71E-86 | 5.10E-84 |
| AC083902.2 | -7.0142 | -0.18192 | 1.95E-86 | 5.73E-84 |
| ASB15 | -7.11564 | 0.214646 | 2.75E-84 | 7.94E-82 |
| NFASC | -3.72756 | 4.39635 | 6.03E-83 | 1.71E-80 |
| CALCA | -6.94869 | 0.863417 | 8.43E-83 | 2.36E-80 |
| ATP1B2 | -3.57652 | 2.348918 | 3.13E-82 | 8.61E-80 |
| HMX2 | -7.92554 | -1.43826 | 8.67E-82 | 2.35E-79 |
| PTPRB | -2.94482 | 4.718392 | 9.34E-82 | 2.49E-79 |
| TMEM207 | -8.686 | 0.663223 | 2.46E-81 | 6.48E-79 |
| SLC12A1 | -7.08808 | 7.981318 | 1.97E-80 | 5.12E-78 |
| GJA3 | -4.82368 | -1.07467 | 3.55E-80 | 9.00E-78 |
| SFRP1 | -5.66289 | 5.578742 | 3.57E-80 | 9.00E-78 |
| MTURN | -2.44563 | 5.956843 | 2.85E-79 | 7.08E-77 |
| MYOZ2 | -4.06746 | -2.23817 | 4.61E-79 | 1.13E-76 |
| GADL1 | -5.13094 | -1.61619 | 3.73E-78 | 9.03E-76 |
| AGXT | -4.87437 | 2.319691 | 7.36E-78 | 1.76E-75 |
| CYP2B6 | -6.32045 | 1.210794 | 1.22E-77 | 2.88E-75 |
| FP236240.1 | -7.52446 | -2.61416 | 1.65E-77 | 3.85E-75 |
| NUGGC | -3.88856 | 0.9723 | 1.38E-76 | 3.17E-74 |
| NELL1 | -6.67171 | 2.302693 | 1.43E-76 | 3.23E-74 |
| NES | -2.77723 | 5.273878 | 2.44E-76 | 5.47E-74 |
| PDE2A | -3.06617 | 3.007044 | 3.22E-76 | 7.11E-74 |
| HELT | -6.21459 | -2.94271 | 5.89E-76 | 1.28E-73 |
| DNASE1 | -2.2909 | 4.837408 | 1.39E-75 | 3.00E-73 |
| TRPV5 | -5.40269 | -1.14124 | 1.49E-75 | 3.17E-73 |
| VSIG8 | -3.36646 | -0.94126 | 1.70E-75 | 3.59E-73 |
| ESRRG | -2.92397 | 4.173626 | 2.50E-75 | 5.21E-73 |
| ERVMER34-1 | -3.47801 | 1.279108 | 4.83E-75 | 9.94E-73 |
| KIF26A | -3.74277 | 2.020976 | 8.29E-75 | 1.69E-72 |
| CAMK2A | -4.74705 | -1.16635 | 1.02E-73 | 2.05E-71 |
| DUSP9 | -6.24855 | 4.154316 | 2.54E-73 | 5.04E-71 |
| ADGRF3 | -2.59111 | 1.209422 | 1.51E-72 | 2.96E-70 |
| GPC5 | -5.85858 | 0.711493 | 1.12E-71 | 2.18E-69 |
| ADGRV1 | -3.72418 | 1.670367 | 2.34E-71 | 4.50E-69 |
| C1orf64 | -4.77039 | -1.4323 | 3.28E-71 | 6.24E-69 |
| CYFIP2 | -2.42538 | 7.810901 | 9.23E-71 | 1.74E-68 |
| NR1I3 | -2.94209 | 0.387964 | 6.40E-70 | 1.19E-67 |
| MUC15 | -7.77403 | 2.67164 | 7.07E-70 | 1.31E-67 |
| RNF150 | -3.1831 | 3.674604 | 1.79E-69 | 3.27E-67 |
| PLA2R1 | -2.96863 | 4.412365 | 3.22E-69 | 5.83E-67 |
| FRMD7 | -6.95849 | -0.97791 | 3.42E-69 | 6.13E-67 |
| EHD3 | -2.37765 | 4.48906 | 4.47E-69 | 7.93E-67 |
| HSPB7 | -4.14745 | 2.692571 | 1.13E-68 | 1.98E-66 |
| PCDH9 | -4.0315 | 1.655142 | 1.29E-68 | 2.24E-66 |
| DLGAP2 | -3.67493 | -0.56768 | 1.34E-68 | 2.30E-66 |
| JAM2 | -2.19359 | 2.587979 | 1.92E-68 | 3.27E-66 |
| LRRC55 | -3.93193 | -1.49035 | 7.95E-68 | 1.34E-65 |
| FGF1 | -4.35975 | 4.19978 | 3.21E-67 | 5.38E-65 |
| XPNPEP2 | -5.70452 | 5.376595 | 6.29E-67 | 1.04E-64 |
| SLC28A2 | -3.35445 | 0.017674 | 1.17E-66 | 1.92E-64 |
| SYN3 | -3.07571 | -1.91488 | 1.46E-66 | 2.38E-64 |
| NPHS1 | -6.29445 | 3.607826 | 2.10E-66 | 3.39E-64 |
| CLDN16 | -4.13593 | 4.019026 | 1.74E-65 | 2.77E-63 |
| MTTP | -4.62656 | 1.085149 | 3.58E-65 | 5.68E-63 |
| TPPP2 | -3.92813 | -2.15935 | 5.14E-65 | 8.07E-63 |
| C2orf71 | -5.75144 | -1.97355 | 6.38E-65 | 9.93E-63 |
| SLC34A1 | -5.18111 | 5.458719 | 8.47E-65 | 1.31E-62 |
| AL590560.1 | -4.44222 | -3.36921 | 2.16E-64 | 3.30E-62 |
| SLC2A12 | -3.76825 | 1.196814 | 4.18E-64 | 6.34E-62 |
| IGDCC3 | -3.78679 | -2.04387 | 4.64E-64 | 6.94E-62 |
| KCNE1B | -4.71271 | -2.495 | 4.65E-64 | 6.94E-62 |
| GNA14 | -2.91234 | 1.294409 | 5.44E-64 | 8.05E-62 |
| SYP | -2.32661 | 1.12221 | 1.20E-63 | 1.76E-61 |
| ABCA4 | -4.76236 | 1.779261 | 3.13E-63 | 4.56E-61 |
| PRDM16 | -5.03994 | 2.523071 | 3.45E-63 | 4.99E-61 |
| FECH | -1.65822 | 5.370306 | 7.25E-63 | 1.04E-60 |
| DNASE1L3 | -3.17045 | 2.573009 | 3.72E-62 | 5.28E-60 |
| HSPA12B | -2.43078 | 2.216933 | 1.29E-61 | 1.82E-59 |
| AFAP1L2 | -3.38494 | 3.89174 | 1.95E-61 | 2.73E-59 |
| SMIM5 | -3.53536 | 3.239072 | 2.69E-61 | 3.73E-59 |
| AQP3 | -3.55534 | 6.504534 | 9.81E-61 | 1.35E-58 |
| ADAMTSL1 | -3.6389 | 1.637514 | 2.19E-60 | 2.99E-58 |
| ALDOB | -6.21053 | 9.480159 | 2.77E-60 | 3.76E-58 |
| SLC36A2 | -6.15642 | 3.75925 | 5.27E-60 | 7.09E-58 |
| CLCNKB | -4.68747 | 5.665924 | 5.37E-60 | 7.17E-58 |
| IGFBP5 | -3.37171 | 8.465323 | 5.85E-60 | 7.75E-58 |
| CASR | -4.65891 | 4.061566 | 2.00E-59 | 2.63E-57 |
| HPSE2 | -4.31763 | -1.67246 | 2.01E-59 | 2.63E-57 |
| TTC36 | -3.03849 | 0.02079 | 2.99E-59 | 3.87E-57 |
| GPAT3 | -2.95681 | 4.703674 | 3.62E-59 | 4.66E-57 |
| C14orf37 | -2.18051 | 3.743561 | 5.16E-59 | 6.60E-57 |
| ST8SIA6 | -3.7699 | -1.6945 | 5.39E-59 | 6.85E-57 |
| PROZ | -4.62437 | 0.755944 | 1.80E-58 | 2.26E-56 |
| ASS1 | -3.12132 | 8.082726 | 2.03E-58 | 2.54E-56 |
| CNTN1 | -4.39332 | 2.117202 | 2.98E-58 | 3.70E-56 |
| TNFRSF10B | 1.858941 | 6.956556 | 3.34E-58 | 4.12E-56 |
| MPPED2 | -3.778 | 1.815434 | 7.56E-58 | 9.27E-56 |
| SUSD2 | -3.52006 | 4.805101 | 9.79E-58 | 1.19E-55 |
| TENM2 | -3.67186 | 0.55532 | 1.12E-57 | 1.36E-55 |
| PSKH2 | -5.27187 | -3.91767 | 3.20E-57 | 3.85E-55 |
| LGI2 | -3.55738 | 2.10338 | 5.42E-57 | 6.47E-55 |
| AQP2 | -8.093 | 8.259731 | 6.98E-57 | 8.28E-55 |
| ACADSB | -2.06263 | 6.107347 | 7.53E-57 | 8.86E-55 |
| TCF21 | -3.92379 | 2.814271 | 7.89E-57 | 9.23E-55 |
| PDZD2 | -3.14566 | 3.326374 | 1.76E-56 | 2.04E-54 |
| FBP1 | -2.62843 | 6.300417 | 3.25E-56 | 3.76E-54 |
| PLG | -6.58739 | 5.323148 | 4.33E-56 | 4.97E-54 |
| JPH4 | -2.4426 | 0.514557 | 4.91E-56 | 5.60E-54 |
| SLC9A3R2 | -1.95734 | 5.624318 | 5.89E-56 | 6.67E-54 |
| TMC1 | -2.82486 | -1.86787 | 6.64E-56 | 7.48E-54 |
| TNNT2 | -4.47386 | 1.378055 | 7.14E-56 | 7.99E-54 |
| ITPKB | -1.71999 | 5.154508 | 7.78E-56 | 8.65E-54 |
| F11 | -5.10781 | 1.925667 | 1.05E-55 | 1.16E-53 |
| DDB2 | 2.125727 | 5.774513 | 1.17E-55 | 1.29E-53 |
| BBC3 | 2.622655 | 4.552682 | 1.74E-55 | 1.90E-53 |
| SH3GL3 | -4.02099 | -0.91822 | 2.23E-55 | 2.42E-53 |
| CLEC3B | -3.05473 | 3.210281 | 8.09E-55 | 8.73E-53 |
| CA8 | -4.46567 | 1.079227 | 9.12E-55 | 9.78E-53 |
| SLC4A9 | -5.1429 | 2.225131 | 9.79E-54 | 1.04E-51 |
| NKD1 | -3.33533 | 0.43702 | 2.88E-53 | 3.05E-51 |
| GPBAR1 | -2.88512 | 0.303066 | 3.74E-53 | 3.94E-51 |
| LPA | -3.83815 | -1.07857 | 5.06E-53 | 5.30E-51 |
| RHBDF2 | 2.020552 | 5.989141 | 6.01E-53 | 6.26E-51 |
| TSPAN7 | -3.21727 | 3.770222 | 7.04E-53 | 7.29E-51 |
| RALYL | -6.37705 | 0.759984 | 7.80E-53 | 8.03E-51 |
| SCNN1B | -5.73832 | 3.824434 | 9.13E-53 | 9.34E-51 |
| SEMG2 | -6.36674 | -2.86398 | 1.91E-52 | 1.94E-50 |
| CYP8B1 | -3.38758 | 3.053411 | 2.88E-52 | 2.91E-50 |
| ANKRD46 | -1.34675 | 4.119917 | 3.14E-52 | 3.16E-50 |
| ARC | -3.59739 | 0.908656 | 1.68E-51 | 1.68E-49 |
| MRGPRF | -3.42769 | 1.787363 | 2.33E-51 | 2.32E-49 |
| UPP2 | -3.49099 | 2.580444 | 3.43E-51 | 3.39E-49 |
| SLC4A1 | -6.09013 | 4.790609 | 4.60E-51 | 4.52E-49 |
| NHLRC4 | -3.50375 | 1.587627 | 5.36E-51 | 5.24E-49 |
| SCTR | -3.0248 | 1.877609 | 1.06E-50 | 1.03E-48 |
| SCUBE3 | -3.12956 | 1.482496 | 1.07E-50 | 1.03E-48 |
| USHBP1 | -2.18991 | 1.24078 | 2.18E-50 | 2.10E-48 |
| WNK1 | -1.71432 | 7.355015 | 4.41E-50 | 4.23E-48 |
| TCF24 | -3.77799 | -2.17289 | 5.08E-50 | 4.83E-48 |
| IL13RA2 | -4.14175 | -0.75178 | 5.41E-50 | 5.12E-48 |
| PCK2 | -2.49742 | 6.511471 | 6.05E-50 | 5.70E-48 |
| ARHGEF15 | -2.43342 | 2.835698 | 7.07E-50 | 6.63E-48 |
| ALAD | -1.52981 | 6.047903 | 1.00E-49 | 9.33E-48 |
| TIE1 | -2.32622 | 4.014684 | 1.16E-49 | 1.08E-47 |
| ELF5 | -5.28937 | 1.973542 | 1.44E-49 | 1.33E-47 |
| IDI1 | -1.30258 | 4.641168 | 1.46E-49 | 1.34E-47 |
| SLC9A4 | -5.65384 | 2.564474 | 1.76E-49 | 1.61E-47 |
| EFHD1 | -2.4629 | 6.316038 | 1.94E-49 | 1.76E-47 |
| TIMP3 | -3.30497 | 2.424983 | 2.00E-49 | 1.81E-47 |
| ALDH6A1 | -2.62799 | 7.35172 | 2.01E-49 | 1.81E-47 |
| NPHS2 | -7.3197 | 3.726291 | 2.03E-49 | 1.82E-47 |
| CYP4A22 | -5.18069 | 2.451503 | 1.04E-48 | 9.22E-47 |
| GCH1 | -1.77035 | 3.178618 | 1.15E-48 | 1.02E-46 |
| CTSV | -2.47395 | 2.122055 | 1.18E-48 | 1.04E-46 |
| SELENBP1 | -1.95491 | 5.692367 | 1.36E-48 | 1.19E-46 |
| RGS6 | -3.87681 | -0.92848 | 1.41E-48 | 1.23E-46 |
| ENPP6 | -4.22407 | 2.933258 | 1.85E-48 | 1.61E-46 |
| GPC3 | -3.43306 | 4.39727 | 2.28E-48 | 1.97E-46 |
| DPT | -4.12977 | 0.760938 | 2.41E-48 | 2.08E-46 |
| NR3C2 | -2.16838 | 4.132012 | 2.49E-48 | 2.13E-46 |
| SPTBN2 | -2.92488 | 6.081799 | 3.58E-48 | 3.05E-46 |
| LDLR | -3.13863 | 3.455447 | 3.62E-48 | 3.07E-46 |
| TMTC1 | -2.61331 | 3.929201 | 5.08E-48 | 4.29E-46 |
| SIM2 | -3.1644 | 3.082135 | 6.11E-48 | 5.13E-46 |
| LMO3 | -3.70894 | 0.842485 | 1.00E-47 | 8.40E-46 |
| TMEM72 | -4.1285 | 4.929017 | 1.29E-47 | 1.08E-45 |
| ATP1A2 | -2.69415 | 1.24094 | 1.36E-47 | 1.13E-45 |
| SLC8A1 | -2.70707 | 4.301163 | 1.64E-47 | 1.35E-45 |
| CNNM2 | -1.40468 | 4.380404 | 2.43E-47 | 2.00E-45 |
| UPB1 | -3.93229 | 3.064378 | 2.76E-47 | 2.26E-45 |
| HS6ST1 | -2.06183 | 5.455882 | 6.28E-47 | 5.11E-45 |
| HK2 | 3.587283 | 5.841265 | 1.12E-46 | 9.10E-45 |
| WDFY2 | -1.28664 | 3.900288 | 1.27E-46 | 1.03E-44 |
| IRX2 | -4.32742 | 2.007815 | 1.52E-46 | 1.22E-44 |
| GHR | -2.73552 | 2.616601 | 1.55E-46 | 1.24E-44 |
| MRO | -3.22971 | 3.022027 | 1.63E-46 | 1.30E-44 |
| FAM222A | -2.57264 | 2.642657 | 1.73E-46 | 1.37E-44 |
| PNPLA1 | -3.21445 | 0.348149 | 1.77E-46 | 1.39E-44 |
| ADCY4 | -2.25666 | 2.329061 | 3.43E-46 | 2.69E-44 |
| LRRC20 | 2.090012 | 4.846631 | 4.42E-46 | 3.46E-44 |
| SLC26A7 | -4.50868 | 3.511116 | 5.92E-46 | 4.61E-44 |
| REN | -4.7889 | 2.809146 | 1.55E-45 | 1.20E-43 |
| OTOGL | -4.52273 | 0.500384 | 1.84E-45 | 1.42E-43 |
| TBC1D13 | -1.1477 | 5.932283 | 2.10E-45 | 1.62E-43 |
| BAX | 1.727988 | 6.353935 | 2.58E-45 | 1.97E-43 |
| WLS | -1.67103 | 6.053739 | 2.77E-45 | 2.11E-43 |
| SCUBE1 | -3.09138 | 1.176317 | 3.05E-45 | 2.31E-43 |
| IGSF10 | -3.01455 | -0.63384 | 4.31E-45 | 3.25E-43 |
| ROBO4 | -2.31657 | 3.973913 | 4.59E-45 | 3.45E-43 |
| ZNF488 | -3.55896 | -0.7721 | 6.86E-45 | 5.14E-43 |
| SELE | -3.91441 | 1.106206 | 7.37E-45 | 5.50E-43 |
| ADGRF5 | -3.04136 | 5.613656 | 1.03E-44 | 7.68E-43 |
| SLC25A25 | -2.42451 | 4.410915 | 1.27E-44 | 9.38E-43 |
| CLDN19 | -4.90639 | 3.82996 | 1.37E-44 | 1.01E-42 |
| ATP6V1B1 | -4.41667 | 4.909472 | 1.39E-44 | 1.02E-42 |
| GJA5 | -2.68964 | 3.248975 | 2.07E-44 | 1.52E-42 |
| TFAP2B | -6.21012 | 2.211581 | 2.40E-44 | 1.75E-42 |
| CLDN14 | -2.75469 | 1.593734 | 3.21E-44 | 2.33E-42 |
| NAT8L | -3.81824 | 3.659457 | 5.27E-44 | 3.80E-42 |
| CYP27B1 | -2.97635 | 3.248655 | 6.88E-44 | 4.95E-42 |
| HRG | -5.99163 | 2.733804 | 8.81E-44 | 6.31E-42 |
| RAG2 | -3.37662 | -3.44661 | 1.41E-43 | 1.01E-41 |
| KCNK10 | -3.52791 | 0.556686 | 1.68E-43 | 1.19E-41 |
| SLC7A8 | -3.47704 | 6.432565 | 3.31E-43 | 2.34E-41 |
| GPIHBP1 | -2.67012 | 1.889329 | 5.96E-43 | 4.20E-41 |
| GPRIN1 | 3.357961 | 3.369319 | 6.89E-43 | 4.84E-41 |
| SLC14A1 | -3.65371 | 3.210115 | 7.86E-43 | 5.50E-41 |
| ACOT6 | -2.84716 | -2.40311 | 8.09E-43 | 5.64E-41 |
| GALNT15 | -2.43755 | 2.232403 | 1.12E-42 | 7.76E-41 |
| IRX1 | -5.77129 | 1.353191 | 1.45E-42 | 1.00E-40 |
| ADRA2B | -2.48749 | 0.543523 | 1.52E-42 | 1.05E-40 |
| TSPAN2 | -2.94431 | 1.588952 | 1.61E-42 | 1.10E-40 |
| PRX | -1.80555 | 2.94729 | 2.03E-42 | 1.38E-40 |
| MMRN2 | -2.0767 | 4.600372 | 2.19E-42 | 1.49E-40 |
| SLC43A2 | -1.60895 | 6.620623 | 2.46E-42 | 1.67E-40 |
| MYOM1 | -2.08222 | 1.297575 | 2.77E-42 | 1.87E-40 |
| LINC00371 | -4.05914 | -3.7097 | 3.01E-42 | 2.03E-40 |
| CHRNA4 | -5.04771 | -0.0885 | 3.14E-42 | 2.11E-40 |
| HADH | -1.55129 | 6.178378 | 3.42E-42 | 2.29E-40 |
| MAGI3 | -1.57189 | 3.754314 | 4.54E-42 | 3.02E-40 |
| HPCAL4 | -4.30389 | 1.027305 | 6.15E-42 | 4.08E-40 |
| SLC4A8 | -2.68469 | 1.656885 | 9.01E-42 | 5.95E-40 |
| GPX3 | -3.25995 | 11.17122 | 9.30E-42 | 6.12E-40 |
| LRRC2 | -3.34538 | 1.65694 | 1.24E-41 | 8.13E-40 |
| ITLN1 | -4.03852 | 0.323619 | 3.16E-41 | 2.06E-39 |
| SLC43A1 | -2.98351 | 2.390083 | 6.14E-41 | 3.99E-39 |
| TACR3 | -5.01657 | -2.88395 | 7.72E-41 | 5.00E-39 |
| MYCT1 | -2.37207 | 1.897947 | 8.92E-41 | 5.77E-39 |
| PDLIM2 | -1.96085 | 3.86701 | 1.35E-40 | 8.67E-39 |
| PLCL1 | -2.82857 | 4.628058 | 2.15E-40 | 1.38E-38 |
| ERG | -2.26133 | 2.851476 | 2.73E-40 | 1.75E-38 |
| PPP1R16B | -2.81746 | 4.167889 | 2.96E-40 | 1.89E-38 |
| FAM26E | -2.75898 | 1.494607 | 5.93E-40 | 3.76E-38 |
| G6PC | -4.97278 | 4.356446 | 6.75E-40 | 4.26E-38 |
| LNX1 | -2.23462 | 3.212356 | 6.76E-40 | 4.26E-38 |
| LDB2 | -2.27414 | 3.439288 | 7.36E-40 | 4.62E-38 |
| NRIP2 | -1.63886 | 2.9256 | 8.36E-40 | 5.24E-38 |
| RAMP3 | -2.42697 | 3.914631 | 8.42E-40 | 5.25E-38 |
| LRRC70 | -2.26975 | -1.68763 | 8.64E-40 | 5.37E-38 |
| NT5C1A | -4.86623 | -2.94091 | 1.06E-39 | 6.57E-38 |
| EPM2A | -1.50145 | 2.938176 | 1.32E-39 | 8.14E-38 |
| TBC1D24 | -1.2334 | 4.647779 | 1.34E-39 | 8.22E-38 |
| TMSB10 | 2.65739 | 10.50567 | 1.41E-39 | 8.67E-38 |
| ASPDH | -3.29671 | 2.910756 | 1.43E-39 | 8.74E-38 |
| UBE2I | 1.03875 | 6.953525 | 1.65E-39 | 1.01E-37 |
| ROS1 | -4.86774 | -1.81494 | 1.70E-39 | 1.03E-37 |
| SSTR2 | -3.01061 | 1.955682 | 1.71E-39 | 1.04E-37 |
| TYRO3 | -2.6878 | 3.434031 | 1.76E-39 | 1.06E-37 |
| FAM184B | -2.19946 | -0.68207 | 2.40E-39 | 1.44E-37 |
| SLC45A1 | -2.1807 | 1.316968 | 2.69E-39 | 1.61E-37 |
| SSUH2 | -2.40286 | -1.20941 | 2.82E-39 | 1.68E-37 |
| NR4A3 | -3.25023 | 3.396451 | 2.94E-39 | 1.75E-37 |
| CYP4A11 | -4.83573 | 5.583242 | 3.99E-39 | 2.36E-37 |
| CD34 | -2.22933 | 5.172979 | 4.00E-39 | 2.36E-37 |
| NUAK2 | -2.27025 | 4.880114 | 4.38E-39 | 2.58E-37 |
| MPP7 | -1.83727 | 3.882861 | 4.39E-39 | 2.58E-37 |
| ATP1A1 | -1.75165 | 10.64257 | 4.88E-39 | 2.86E-37 |
| PRKCE | -1.8022 | 3.696142 | 5.54E-39 | 3.23E-37 |
| FBXO21 | -1.17007 | 6.163806 | 5.76E-39 | 3.35E-37 |
| SLC12A3 | -5.35436 | 6.159767 | 5.79E-39 | 3.35E-37 |
| TNFSF9 | 4.073828 | 3.519255 | 5.80E-39 | 3.35E-37 |
| CA4 | -4.05293 | 2.414721 | 5.91E-39 | 3.40E-37 |
| EYA4 | -4.20073 | 1.194389 | 9.78E-39 | 5.61E-37 |
| NOS3 | -2.26131 | 2.802411 | 1.19E-38 | 6.82E-37 |
| TRPM6 | -3.73676 | 1.047438 | 2.05E-38 | 1.17E-36 |
| SELP | -2.38534 | 2.323544 | 2.08E-38 | 1.18E-36 |
| GPR22 | -3.19894 | -3.63714 | 2.46E-38 | 1.40E-36 |
| SREBF2 | -1.21742 | 6.674113 | 2.64E-38 | 1.49E-36 |
| TMEM213 | -4.66952 | 5.471648 | 2.94E-38 | 1.65E-36 |
| DAGLB | 1.609598 | 5.445236 | 3.27E-38 | 1.83E-36 |
| BCAM | -1.69977 | 8.456693 | 3.94E-38 | 2.20E-36 |
| SEMA6D | -2.87939 | 3.874341 | 4.37E-38 | 2.44E-36 |
| AEN | 1.300723 | 5.028304 | 7.23E-38 | 4.02E-36 |
| RCAN1 | -1.82572 | 6.370207 | 1.03E-37 | 5.71E-36 |
| FAM217A | -2.63829 | -2.47045 | 1.40E-37 | 7.74E-36 |
| NOL3 | 2.004228 | 5.714204 | 1.93E-37 | 1.06E-35 |
| GPM6A | -2.86886 | 1.548574 | 2.19E-37 | 1.20E-35 |
| MME | -3.43335 | 6.436606 | 3.51E-37 | 1.92E-35 |
| HAMP | 5.185985 | 2.267426 | 3.61E-37 | 1.97E-35 |
| STARD8 | -1.77885 | 4.567167 | 3.91E-37 | 2.13E-35 |
| C17orf62 | 1.441853 | 6.619997 | 5.13E-37 | 2.78E-35 |
| C7 | -3.68803 | 7.088548 | 6.05E-37 | 3.27E-35 |
| ADH1C | -4.09575 | 1.632701 | 6.22E-37 | 3.35E-35 |
| CLMN | -1.73822 | 5.780588 | 6.37E-37 | 3.43E-35 |
| KNG1 | -6.11636 | 6.756508 | 1.25E-36 | 6.72E-35 |
| KCNJ9 | -3.24959 | -3.13623 | 1.29E-36 | 6.92E-35 |
| SLFN13 | 2.558204 | 5.627661 | 1.59E-36 | 8.46E-35 |
| EDA2R | 2.423681 | 3.648333 | 1.69E-36 | 8.97E-35 |
| MAN1A1 | -1.61709 | 6.343841 | 1.72E-36 | 9.10E-35 |
| KLHL15 | -1.25987 | 3.098333 | 2.17E-36 | 1.15E-34 |
| LRRC28 | -1.08287 | 3.96317 | 2.38E-36 | 1.25E-34 |
| ADH1B | -3.56953 | 3.890086 | 2.55E-36 | 1.34E-34 |
| B3GALT2 | -2.48581 | -0.93639 | 3.16E-36 | 1.65E-34 |
| HOXD8 | -2.27713 | 4.717837 | 3.71E-36 | 1.94E-34 |
| KLHL33 | -2.59222 | -2.61974 | 4.05E-36 | 2.11E-34 |
| RNF149 | 1.102623 | 6.17409 | 4.08E-36 | 2.12E-34 |
| PEPD | -1.83649 | 7.596938 | 4.41E-36 | 2.28E-34 |
| SH3YL1 | -1.49983 | 5.447496 | 4.49E-36 | 2.32E-34 |
| CEACAM1 | -2.315 | 3.702466 | 4.79E-36 | 2.47E-34 |
| APOC1 | 6.530577 | 7.8145 | 6.48E-36 | 3.32E-34 |
| TREM2 | 4.724631 | 4.896946 | 7.68E-36 | 3.93E-34 |
| HPD | -4.21619 | 6.053824 | 7.75E-36 | 3.95E-34 |
| LDB3 | -2.30345 | 0.257834 | 8.83E-36 | 4.49E-34 |
| TACC1 | -1.47316 | 6.376905 | 1.13E-35 | 5.72E-34 |
| TRAPPC3L | -2.71102 | -3.43565 | 1.13E-35 | 5.72E-34 |
| SH3BP4 | -1.79128 | 5.622383 | 1.48E-35 | 7.47E-34 |
| PRODH2 | -3.95547 | 4.782967 | 1.96E-35 | 9.83E-34 |
| AGMAT | -3.26663 | 5.590641 | 2.07E-35 | 1.04E-33 |
| TTPA | -2.8426 | -1.30937 | 2.13E-35 | 1.07E-33 |
| GABRA4 | -4.79158 | -3.10306 | 2.19E-35 | 1.09E-33 |
| SLITRK3 | -3.64492 | -3.98895 | 2.52E-35 | 1.25E-33 |
| FMO5 | -1.79451 | 3.916877 | 2.57E-35 | 1.27E-33 |
| ACSL6 | -2.76671 | 1.449689 | 3.57E-35 | 1.77E-33 |
| FMN2 | -4.08044 | -0.06589 | 3.66E-35 | 1.80E-33 |
| UMODL1 | -3.42418 | -2.19606 | 3.79E-35 | 1.87E-33 |
| SLC15A4 | 1.156163 | 5.593623 | 3.89E-35 | 1.91E-33 |
| NR4A2 | -2.58809 | 3.725163 | 5.03E-35 | 2.46E-33 |
| TAPBP | 1.595692 | 8.899207 | 5.27E-35 | 2.57E-33 |
| GDNF | -3.40973 | -1.4757 | 5.71E-35 | 2.78E-33 |
| DAAM2 | -2.43523 | 2.866527 | 5.97E-35 | 2.90E-33 |
| ITGAX | 3.490758 | 5.481551 | 8.79E-35 | 4.25E-33 |
| STAC3 | 2.513494 | 2.627253 | 9.74E-35 | 4.70E-33 |
| NTN4 | -1.75026 | 6.672188 | 1.04E-34 | 4.99E-33 |
| NRN1 | -2.15662 | 1.374446 | 1.19E-34 | 5.70E-33 |
| DGKB | -3.25075 | -1.02827 | 1.31E-34 | 6.26E-33 |
| WWC2 | -1.39576 | 5.182343 | 1.62E-34 | 7.72E-33 |
| NIPAL1 | -2.558 | 2.191199 | 1.76E-34 | 8.36E-33 |
| RGPD3 | -2.83567 | -2.34221 | 1.81E-34 | 8.58E-33 |
| CDC14A | -1.39054 | 2.75378 | 1.91E-34 | 9.05E-33 |
| ZNF366 | -2.60088 | 0.317641 | 2.04E-34 | 9.63E-33 |
| NDNF | -4.93014 | 3.244814 | 2.16E-34 | 1.02E-32 |
| ADAMTS6 | -2.38081 | -0.87228 | 2.23E-34 | 1.05E-32 |
| GJD2 | -4.60861 | -3.44194 | 2.32E-34 | 1.09E-32 |
| THY1 | -2.85646 | 6.36318 | 2.37E-34 | 1.11E-32 |
| ARL15 | -1.35287 | 4.353341 | 2.46E-34 | 1.15E-32 |
| PTPN4 | -1.15171 | 4.2931 | 2.52E-34 | 1.17E-32 |
| CD276 | 1.330758 | 6.404118 | 2.63E-34 | 1.22E-32 |
| CYYR1 | -2.18232 | 3.152068 | 3.16E-34 | 1.46E-32 |
| ACSBG2 | -2.40621 | -1.40175 | 3.28E-34 | 1.51E-32 |
| SORD | -2.33189 | 4.95469 | 3.35E-34 | 1.54E-32 |
| AVPR1A | -3.6061 | 2.109188 | 3.41E-34 | 1.57E-32 |
| TMEM45B | -2.83867 | 3.315655 | 3.45E-34 | 1.58E-32 |
| STC1 | -3.04061 | 5.102738 | 3.67E-34 | 1.68E-32 |
| DUSP26 | -3.23708 | -0.20076 | 3.81E-34 | 1.74E-32 |
| C4orf32 | -1.997 | 2.410874 | 4.07E-34 | 1.85E-32 |
| AK3 | -1.12781 | 6.319556 | 4.21E-34 | 1.91E-32 |
| BHLHE41 | 2.480405 | 6.133776 | 4.41E-34 | 1.99E-32 |
| PSORS1C1 | 3.50223 | 3.483307 | 4.81E-34 | 2.17E-32 |
| AMFR | -1.22176 | 7.234903 | 5.92E-34 | 2.66E-32 |
| SPATA12 | 3.425338 | -0.13545 | 6.94E-34 | 3.12E-32 |
| TMEM61 | -2.76501 | 1.397679 | 7.30E-34 | 3.27E-32 |
| COL25A1 | -2.67659 | -0.78337 | 8.46E-34 | 3.78E-32 |
| CREB5 | 2.870077 | 6.598401 | 8.84E-34 | 3.93E-32 |
| TMEM204 | -2.1134 | 3.628846 | 9.66E-34 | 4.29E-32 |
| NOS1AP | -2.44697 | 0.56517 | 9.67E-34 | 4.29E-32 |
| IDNK | -1.49568 | 3.078554 | 1.03E-33 | 4.55E-32 |
| C16orf59 | 2.487264 | 2.108658 | 1.04E-33 | 4.57E-32 |
| C9orf84 | -3.25293 | -0.31323 | 1.04E-33 | 4.59E-32 |
| SLC6A19 | -4.04218 | 6.019112 | 1.15E-33 | 5.05E-32 |
| FDXR | 2.215393 | 5.916446 | 1.16E-33 | 5.07E-32 |
| HSD11B2 | -4.0706 | 6.857869 | 1.18E-33 | 5.14E-32 |
| AHNAK2 | 4.24666 | 7.404125 | 1.34E-33 | 5.82E-32 |
| LMX1B | -3.72074 | 1.177622 | 1.34E-33 | 5.84E-32 |
| EPAS1 | -1.85063 | 7.482214 | 1.50E-33 | 6.49E-32 |
| S100A11 | 1.785247 | 8.98566 | 1.96E-33 | 8.48E-32 |
| ARPC1B | 1.988152 | 8.097736 | 1.98E-33 | 8.54E-32 |
| IKBIP | 1.865438 | 5.237027 | 2.63E-33 | 1.13E-31 |
| PIPOX | -3.40732 | 4.932768 | 3.18E-33 | 1.37E-31 |
| RHBDF1 | 1.431411 | 5.610058 | 3.26E-33 | 1.40E-31 |
| CYP4X1 | -2.45879 | 0.939616 | 3.33E-33 | 1.42E-31 |
| TNS2 | -1.35674 | 6.193831 | 3.35E-33 | 1.43E-31 |
| FAM184A | -2.111 | 1.578402 | 3.37E-33 | 1.43E-31 |
| SULT1B1 | -2.37975 | -0.61635 | 5.03E-33 | 2.13E-31 |
| E2F1 | 2.744199 | 2.772106 | 5.49E-33 | 2.32E-31 |
| KCTD13 | 1.349808 | 4.421981 | 5.67E-33 | 2.39E-31 |
| AGTR1 | -2.89569 | 1.047555 | 6.35E-33 | 2.67E-31 |
| KCNK13 | -2.39347 | 1.746363 | 6.55E-33 | 2.75E-31 |
| PCOLCE2 | -2.85647 | 2.984315 | 7.30E-33 | 3.06E-31 |
| PRR7 | 2.738374 | 2.302605 | 7.54E-33 | 3.15E-31 |
| GPR155 | -2.11494 | 4.019771 | 8.19E-33 | 3.42E-31 |
| MFSD6L | -3.42246 | -0.02513 | 9.10E-33 | 3.79E-31 |
| ZFYVE9 | -1.24863 | 4.342641 | 1.21E-32 | 5.01E-31 |
| FAM169A | -3.12523 | 1.158026 | 1.23E-32 | 5.11E-31 |
| SNX18 | -1.34286 | 4.673246 | 1.25E-32 | 5.17E-31 |
| RDH12 | -3.00101 | 1.63458 | 1.35E-32 | 5.56E-31 |
| C9orf66 | -2.41747 | 2.988281 | 1.43E-32 | 5.89E-31 |
| EZH2 | 2.026812 | 2.921983 | 1.46E-32 | 6.00E-31 |
| NR2F1 | -2.54303 | 3.750367 | 1.47E-32 | 6.02E-31 |
| YBX3 | 1.369998 | 7.932254 | 1.66E-32 | 6.78E-31 |
| RAP1GAP | -2.19217 | 6.699611 | 1.73E-32 | 7.07E-31 |
| ANK2 | -2.99486 | 6.000378 | 1.95E-32 | 7.92E-31 |
| TLN2 | -1.59511 | 6.483048 | 2.01E-32 | 8.17E-31 |
| FAM110D | -2.17321 | 0.267862 | 2.12E-32 | 8.57E-31 |
| ZNF331 | -1.71199 | 4.349233 | 2.28E-32 | 9.20E-31 |
| SYT7 | -3.78752 | 2.587832 | 2.34E-32 | 9.44E-31 |
| MELK | 3.408675 | 1.721809 | 2.36E-32 | 9.48E-31 |
| KCNN2 | -2.57126 | -0.45007 | 2.49E-32 | 1.00E-30 |
| CYP4F2 | -5.19833 | 3.259655 | 2.92E-32 | 1.17E-30 |
| CCM2L | -2.09858 | 1.529093 | 2.96E-32 | 1.18E-30 |
| MET | 2.379498 | 9.112053 | 3.32E-32 | 1.32E-30 |
| PLIN1 | -2.81794 | 0.292979 | 3.51E-32 | 1.40E-30 |
| B3GNTL1 | 1.813094 | 3.135886 | 4.49E-32 | 1.78E-30 |
| TMPRSS2 | -3.38052 | 4.528484 | 5.35E-32 | 2.12E-30 |
| SLC22A8 | -5.56387 | 6.121648 | 5.97E-32 | 2.36E-30 |
| THRB | -1.83143 | 3.93895 | 6.03E-32 | 2.38E-30 |
| STX4 | 1.214856 | 6.057208 | 7.77E-32 | 3.06E-30 |
| GINS2 | 2.540995 | 2.683777 | 8.64E-32 | 3.39E-30 |
| CHEK2 | 1.964064 | 3.214947 | 9.31E-32 | 3.65E-30 |
| PTGFRN | 1.948062 | 6.998546 | 9.88E-32 | 3.86E-30 |
| PHLDA3 | 2.197451 | 6.271109 | 1.05E-31 | 4.10E-30 |
| TMEM88 | -1.61057 | 2.346842 | 1.11E-31 | 4.33E-30 |
| COLGALT1 | 1.35887 | 6.6791 | 1.34E-31 | 5.20E-30 |
| CACNA1S | -3.80805 | -1.96339 | 1.34E-31 | 5.20E-30 |
| ITGA8 | -2.62344 | 3.247897 | 1.39E-31 | 5.39E-30 |
| ARHGAP22 | 2.653412 | 2.718077 | 1.57E-31 | 6.05E-30 |
| CASQ2 | -2.95667 | 0.027257 | 1.77E-31 | 6.80E-30 |
| ATP8B3 | 3.699772 | 2.982724 | 1.93E-31 | 7.40E-30 |
| ADAMTSL2 | -2.3683 | 3.914788 | 2.21E-31 | 8.49E-30 |
| OPCML | -3.69021 | -0.09852 | 2.46E-31 | 9.40E-30 |
| PANK1 | -1.80215 | 4.333454 | 2.53E-31 | 9.65E-30 |
| VIM | 1.76396 | 10.44938 | 2.63E-31 | 1.00E-29 |
| MLKL | 1.629924 | 4.12741 | 2.65E-31 | 1.00E-29 |
| GSTM3 | -2.46088 | 6.097675 | 2.68E-31 | 1.01E-29 |
| GATA2 | -2.60518 | 3.399882 | 2.97E-31 | 1.12E-29 |
| LPL | -2.49513 | 4.136407 | 3.05E-31 | 1.15E-29 |
| TFCP2L1 | -2.37915 | 6.644741 | 3.06E-31 | 1.15E-29 |
| AXIN1 | 1.170985 | 5.861875 | 3.12E-31 | 1.17E-29 |
| PTPRQ | -4.70637 | -1.3682 | 3.18E-31 | 1.19E-29 |
| EPHX2 | -1.54916 | 6.453425 | 3.28E-31 | 1.23E-29 |
| KANK4 | -2.66554 | -0.23423 | 3.53E-31 | 1.32E-29 |
| UNC45B | -2.77848 | -2.39893 | 3.65E-31 | 1.36E-29 |
| KCNN3 | -2.74152 | 2.09059 | 4.09E-31 | 1.52E-29 |
| USP46 | -1.25736 | 3.855884 | 4.61E-31 | 1.71E-29 |
| C5orf67 | -2.58088 | -0.78484 | 5.56E-31 | 2.06E-29 |
| PSMB8 | 1.592962 | 6.97327 | 5.86E-31 | 2.16E-29 |
| CHFR | 1.088627 | 4.881198 | 6.59E-31 | 2.43E-29 |
| ENG | -1.5517 | 6.898419 | 7.55E-31 | 2.78E-29 |
| FOLH1 | -2.85083 | 1.724674 | 7.92E-31 | 2.90E-29 |
| APBB2 | -1.30367 | 5.068855 | 8.04E-31 | 2.94E-29 |
| HRC | -2.11752 | 0.748113 | 8.33E-31 | 3.04E-29 |
| SOCS2 | -2.05494 | 3.312859 | 9.18E-31 | 3.35E-29 |
| RUNX1T1 | -2.24957 | 0.61745 | 9.30E-31 | 3.38E-29 |
| ASPHD1 | 3.601163 | 4.741307 | 1.05E-30 | 3.81E-29 |
| CDKN2A | 4.62076 | 2.368925 | 1.16E-30 | 4.22E-29 |
| TNS1 | -1.5089 | 8.231714 | 1.22E-30 | 4.40E-29 |
| SERPINA5 | -3.6113 | 5.988543 | 1.44E-30 | 5.21E-29 |
| DNAJC16 | -1.09893 | 5.2323 | 1.49E-30 | 5.37E-29 |
| ANXA2 | 1.47557 | 9.342245 | 1.54E-30 | 5.53E-29 |
| PCDH12 | -1.83028 | 3.432782 | 1.65E-30 | 5.93E-29 |
| S1PR1 | -1.92667 | 3.799717 | 1.73E-30 | 6.19E-29 |
| TPK1 | 3.199085 | 5.766185 | 1.77E-30 | 6.34E-29 |
| PPM1K | -1.49126 | 4.751791 | 2.07E-30 | 7.40E-29 |
| UCN | 3.355329 | 1.344433 | 2.37E-30 | 8.44E-29 |
| FABP1 | -4.89624 | 3.524574 | 2.45E-30 | 8.71E-29 |
| CDT1 | 2.688587 | 2.89974 | 2.65E-30 | 9.41E-29 |
| MECOM | -3.04573 | 5.223978 | 2.76E-30 | 9.78E-29 |
| C1orf226 | -2.09777 | 2.928642 | 2.98E-30 | 1.05E-28 |
| SOX17 | -2.04862 | 0.957111 | 3.71E-30 | 1.31E-28 |
| SEZ6L2 | 3.181967 | 7.400336 | 3.94E-30 | 1.38E-28 |
| LBX2 | 3.162597 | 2.010632 | 4.93E-30 | 1.73E-28 |
| MGAT4B | 1.297965 | 8.226178 | 4.98E-30 | 1.74E-28 |
| GALNT18 | -1.67411 | 4.819898 | 5.05E-30 | 1.77E-28 |
| ART1 | -3.05087 | -3.81864 | 5.12E-30 | 1.79E-28 |
| SLC25A33 | -1.53589 | 2.906878 | 5.26E-30 | 1.83E-28 |
| PRMT8 | -3.2113 | -2.72313 | 6.35E-30 | 2.21E-28 |
| PKMYT1 | 3.43369 | 1.797529 | 6.40E-30 | 2.22E-28 |
| ANXA1 | 2.325912 | 8.207467 | 6.67E-30 | 2.31E-28 |
| FAM180A | -2.96795 | -0.16652 | 7.23E-30 | 2.50E-28 |
| SH2D5 | 3.463515 | 2.104132 | 7.83E-30 | 2.69E-28 |
| SELENOP | -2.12365 | 6.275162 | 7.83E-30 | 2.69E-28 |
| BHMT | -3.23408 | 7.215717 | 9.42E-30 | 3.24E-28 |
| PM20D1 | -3.26019 | 0.74624 | 1.27E-29 | 4.36E-28 |
| ANGPTL1 | -2.69179 | 2.274524 | 1.35E-29 | 4.61E-28 |
| ARHGAP28 | -1.89641 | 2.89813 | 1.47E-29 | 5.01E-28 |
| LGSN | -3.68528 | -0.88525 | 1.54E-29 | 5.23E-28 |
| LRRTM2 | -2.52674 | -2.37849 | 1.63E-29 | 5.53E-28 |
| ARG2 | -2.98008 | 4.169872 | 1.65E-29 | 5.59E-28 |
| TUSC5 | -4.40869 | -1.98818 | 1.67E-29 | 5.65E-28 |
| SMIM10L2B | -1.87836 | 1.662083 | 1.70E-29 | 5.74E-28 |
| LRRC10B | -2.89485 | -0.8866 | 1.91E-29 | 6.44E-28 |
| PLOD3 | 1.455229 | 7.230375 | 2.03E-29 | 6.84E-28 |
| DHH | -2.25685 | -1.84746 | 2.07E-29 | 6.94E-28 |
| ZNF521 | -2.49524 | 1.280305 | 2.07E-29 | 6.94E-28 |
| CASZ1 | -1.67131 | 3.263465 | 2.18E-29 | 7.28E-28 |
| UNC5C | -2.88683 | 0.663594 | 2.18E-29 | 7.30E-28 |
| LTBR | 1.169667 | 7.624982 | 2.41E-29 | 8.03E-28 |
| SUSD5 | -2.39498 | 0.381856 | 2.67E-29 | 8.86E-28 |
| GPX1 | 1.799499 | 8.97721 | 2.75E-29 | 9.10E-28 |
| CHP2 | -4.67441 | -0.87155 | 3.07E-29 | 1.02E-27 |
| OXGR1 | -4.48558 | -0.44756 | 3.08E-29 | 1.02E-27 |
| CCDC88B | 2.34014 | 4.548447 | 3.10E-29 | 1.02E-27 |
| ESAM | -1.78262 | 5.185873 | 3.57E-29 | 1.18E-27 |
| LIN52 | -1.05628 | 3.341533 | 3.74E-29 | 1.23E-27 |
| C1QTNF7 | -3.05536 | 0.892725 | 3.84E-29 | 1.26E-27 |
| ZP2 | -3.30461 | -3.69831 | 4.11E-29 | 1.35E-27 |
| SOX7 | -1.95505 | 1.023874 | 4.30E-29 | 1.41E-27 |
| RNF152 | -2.76722 | 5.767505 | 4.32E-29 | 1.41E-27 |
| FBLN5 | -1.84607 | 5.996028 | 4.55E-29 | 1.48E-27 |
| MCU | 1.481315 | 6.14357 | 4.70E-29 | 1.53E-27 |
| TNFRSF12A | 2.1739 | 8.200644 | 5.87E-29 | 1.90E-27 |
| TNFAIP8 | 1.581175 | 5.704247 | 6.04E-29 | 1.95E-27 |
| SDS | 4.303814 | 3.9571 | 6.07E-29 | 1.96E-27 |
| RIMKLA | 1.92798 | 4.312376 | 6.13E-29 | 1.97E-27 |
| CDKN1C | -1.93176 | 4.862159 | 6.67E-29 | 2.14E-27 |
| ODF3B | 3.014699 | 4.685243 | 7.72E-29 | 2.47E-27 |
| MYO3B | -4.11495 | 2.164952 | 7.97E-29 | 2.55E-27 |
| BMP1 | 1.979164 | 6.065844 | 8.37E-29 | 2.67E-27 |
| FGF10 | -4.41786 | -2.78115 | 9.21E-29 | 2.93E-27 |
| TBXA2R | -2.14383 | 1.236439 | 9.34E-29 | 2.97E-27 |
| SEL1L3 | 1.943416 | 7.316336 | 9.50E-29 | 3.01E-27 |
| HS6ST2 | -3.92402 | 2.88188 | 9.99E-29 | 3.16E-27 |
| C1RL | 1.353062 | 6.347049 | 1.08E-28 | 3.41E-27 |
| THOC6 | 1.598394 | 5.448644 | 1.12E-28 | 3.53E-27 |
| GAS1 | -2.15496 | 2.234587 | 1.21E-28 | 3.80E-27 |
| CGNL1 | -2.67599 | 6.777225 | 1.21E-28 | 3.81E-27 |
| NUP85 | 1.053888 | 5.150125 | 1.22E-28 | 3.81E-27 |
| CMTM4 | -1.37802 | 6.48748 | 1.28E-28 | 4.02E-27 |
| TPST2 | -1.61223 | 4.606032 | 1.62E-28 | 5.05E-27 |
| TCF7L1 | -1.70071 | 3.206043 | 1.82E-28 | 5.69E-27 |
| RCN1 | 1.429629 | 7.030048 | 2.02E-28 | 6.27E-27 |
| DCN | -2.834 | 6.382919 | 2.11E-28 | 6.56E-27 |
| KMO | -2.46145 | 3.033385 | 2.26E-28 | 7.01E-27 |
| SUSD4 | -2.55496 | 3.537058 | 2.28E-28 | 7.04E-27 |
| TYMS | 1.843517 | 4.60807 | 2.45E-28 | 7.56E-27 |
| OPHN1 | -1.29789 | 3.091632 | 2.59E-28 | 7.98E-27 |
| EPHB3 | -2.62916 | 1.875934 | 2.61E-28 | 8.02E-27 |
| KIAA1191 | -1.10641 | 7.417353 | 2.95E-28 | 9.05E-27 |
| PRR35 | -5.57578 | 1.328516 | 3.14E-28 | 9.62E-27 |
| CAT | -1.43264 | 7.312599 | 3.49E-28 | 1.07E-26 |
| NTF3 | -2.33221 | -0.72239 | 3.54E-28 | 1.08E-26 |
| PEG3 | -2.39749 | 2.33119 | 3.71E-28 | 1.13E-26 |
| KDR | -2.45277 | 4.510185 | 3.85E-28 | 1.17E-26 |
| NOVA2 | -2.04314 | 1.237595 | 4.17E-28 | 1.27E-26 |
| SOX13 | -1.3967 | 4.728971 | 4.91E-28 | 1.49E-26 |
| REEP4 | 1.388701 | 4.284141 | 5.04E-28 | 1.53E-26 |
| ACSF2 | -2.38137 | 5.860543 | 5.14E-28 | 1.55E-26 |
| ZNF44 | -1.22836 | 4.259245 | 5.33E-28 | 1.61E-26 |
| IDO2 | -2.45851 | -2.92837 | 5.39E-28 | 1.62E-26 |
| TIMP1 | 2.284623 | 8.517947 | 6.00E-28 | 1.80E-26 |
| FLT4 | -2.02538 | 3.772213 | 7.14E-28 | 2.14E-26 |
| CDH10 | -3.66737 | -3.42904 | 7.42E-28 | 2.22E-26 |
| GREM2 | -3.3055 | -1.78578 | 7.45E-28 | 2.23E-26 |
| GGT6 | -3.88054 | 4.33878 | 7.88E-28 | 2.35E-26 |
| ADTRP | -2.77575 | 1.197468 | 8.38E-28 | 2.49E-26 |
| AC005726.2 | -2.06603 | 1.946963 | 8.90E-28 | 2.65E-26 |
| CSRP1 | -1.24254 | 7.037325 | 1.05E-27 | 3.13E-26 |
| PLPPR1 | -3.28032 | 2.508531 | 1.10E-27 | 3.25E-26 |
| TKFC | -1.10455 | 6.356552 | 1.24E-27 | 3.67E-26 |
| ARHGAP27 | 1.461977 | 6.262423 | 1.25E-27 | 3.69E-26 |
| CPAMD8 | -2.90131 | 3.746696 | 1.25E-27 | 3.70E-26 |
| TMEM164 | -1.07863 | 5.13686 | 1.28E-27 | 3.78E-26 |
| AL359736.1 | -2.47057 | -3.88444 | 1.33E-27 | 3.90E-26 |
| CD63 | 1.469706 | 10.13797 | 1.38E-27 | 4.06E-26 |
| RGS7BP | -2.80017 | -0.19109 | 1.47E-27 | 4.31E-26 |
| RNF217 | 1.483168 | 4.759117 | 1.60E-27 | 4.67E-26 |
| RIN1 | 2.510193 | 3.943442 | 1.60E-27 | 4.68E-26 |
| C11orf16 | -2.42059 | -2.31469 | 1.90E-27 | 5.53E-26 |
| KLK7 | -4.7192 | 0.600447 | 1.94E-27 | 5.63E-26 |
| SLC16A2 | -1.97157 | 4.15302 | 2.02E-27 | 5.85E-26 |
| MPV17 | 1.104224 | 6.337902 | 2.02E-27 | 5.85E-26 |
| NEGR1 | -2.93078 | 1.883799 | 2.73E-27 | 7.88E-26 |
| NOTCH4 | -1.74868 | 4.154204 | 2.74E-27 | 7.90E-26 |
| LIMK1 | 1.434329 | 5.910991 | 2.90E-27 | 8.35E-26 |
| OAS1 | 1.711555 | 5.82905 | 2.94E-27 | 8.47E-26 |
| TMIGD3 | 3.556817 | 3.702428 | 3.09E-27 | 8.87E-26 |
| DNM1 | 3.110688 | 6.939127 | 3.75E-27 | 1.08E-25 |
| PDILT | -2.75206 | -3.33729 | 3.94E-27 | 1.13E-25 |
| AKIRIN1 | -1.01411 | 5.494763 | 4.14E-27 | 1.18E-25 |
| HBEGF | -1.86192 | 3.976821 | 4.22E-27 | 1.21E-25 |
| ST6GAL1 | -2.17406 | 6.961535 | 4.26E-27 | 1.21E-25 |
| GIMAP8 | -1.79574 | 3.289466 | 4.32E-27 | 1.23E-25 |
| PPP1R12B | -1.49027 | 4.979872 | 4.49E-27 | 1.28E-25 |
| ENO2 | 2.566432 | 6.295914 | 4.50E-27 | 1.28E-25 |
| SLC25A42 | -1.68014 | 4.846358 | 4.68E-27 | 1.33E-25 |
| CREM | -1.03488 | 4.424769 | 4.73E-27 | 1.34E-25 |
| SYDE2 | -1.48927 | 1.818712 | 5.62E-27 | 1.59E-25 |
| PXDN | 2.43562 | 7.501148 | 6.21E-27 | 1.75E-25 |
| CHAC1 | -2.29196 | 1.594933 | 6.38E-27 | 1.79E-25 |
| PYGM | -1.90477 | 0.258845 | 7.14E-27 | 2.00E-25 |
| TUBB3 | 3.88707 | 2.4195 | 7.37E-27 | 2.06E-25 |
| SLC5A2 | -3.9466 | 3.465362 | 7.46E-27 | 2.08E-25 |
| FBXL19 | 1.259085 | 5.180028 | 7.90E-27 | 2.20E-25 |
| SNCAIP | -2.19132 | 0.132472 | 8.13E-27 | 2.26E-25 |
| PAQR9 | 4.86279 | -0.31714 | 8.43E-27 | 2.34E-25 |
| HAO1 | -4.5771 | -2.6898 | 9.39E-27 | 2.61E-25 |
| NME1 | 1.980833 | 5.920547 | 9.51E-27 | 2.63E-25 |
| DNMT3L | -3.91044 | -2.802 | 1.03E-26 | 2.85E-25 |
| LINC00694 | -2.45585 | -2.31166 | 1.05E-26 | 2.90E-25 |
| FCN3 | -2.7421 | 1.919399 | 1.15E-26 | 3.16E-25 |
| KCNQ1 | -2.44638 | 4.816369 | 1.15E-26 | 3.16E-25 |
| RFC2 | 1.121281 | 4.751644 | 1.19E-26 | 3.26E-25 |
| TYRP1 | -4.35633 | 1.548086 | 1.22E-26 | 3.34E-25 |
| BNIPL | -2.0541 | -0.02308 | 1.31E-26 | 3.59E-25 |
| AGTR2 | -4.48723 | -3.20882 | 1.37E-26 | 3.75E-25 |
| CORO1C | 1.30949 | 7.331455 | 1.41E-26 | 3.85E-25 |
| TBC1D9 | -1.32499 | 5.429466 | 1.41E-26 | 3.85E-25 |
| FOXO1 | -1.25198 | 4.58824 | 1.67E-26 | 4.55E-25 |
| MEIS2 | -2.09825 | 3.036623 | 1.68E-26 | 4.57E-25 |
| AOC3 | -2.32541 | 3.915228 | 1.71E-26 | 4.63E-25 |
| PAK6 | -3.16145 | -1.80577 | 1.75E-26 | 4.73E-25 |
| ANGPT1 | -2.60121 | 2.069226 | 1.99E-26 | 5.38E-25 |
| TMEM140 | 1.497506 | 6.752302 | 2.01E-26 | 5.41E-25 |
| ALOX5 | 2.739989 | 6.099784 | 2.03E-26 | 5.47E-25 |
| SOGA3 | 3.40828 | 0.613668 | 2.18E-26 | 5.86E-25 |
| PTGER4 | -1.88142 | 2.895585 | 2.18E-26 | 5.86E-25 |
| CPT1A | -1.26024 | 6.657003 | 2.36E-26 | 6.34E-25 |
| CENPH | 1.652617 | 2.047863 | 2.46E-26 | 6.60E-25 |
| DMGDH | -1.95091 | 5.772108 | 2.50E-26 | 6.70E-25 |
| OSCAR | 3.155949 | 2.789532 | 2.70E-26 | 7.20E-25 |
| SEMA4A | -1.94742 | 4.134501 | 2.80E-26 | 7.48E-25 |
| SCN7A | -3.39834 | -0.10813 | 2.91E-26 | 7.76E-25 |
| SLC5A12 | -4.02636 | 5.866868 | 3.05E-26 | 8.13E-25 |
| HTRA4 | 5.421601 | 1.365787 | 3.08E-26 | 8.18E-25 |
| NCOA7 | -1.49197 | 5.826391 | 3.49E-26 | 9.24E-25 |
| STRA6 | -3.5916 | 2.705288 | 3.66E-26 | 9.69E-25 |
| ETNK2 | -1.89838 | 5.443622 | 3.70E-26 | 9.79E-25 |
| CDC45 | 2.951083 | 1.172747 | 3.73E-26 | 9.86E-25 |
| CBX7 | -1.14219 | 5.686813 | 3.90E-26 | 1.03E-24 |
| KCNA1 | -3.28755 | -3.88644 | 4.21E-26 | 1.11E-24 |
| SRGAP2B | -1.52772 | 0.867598 | 4.39E-26 | 1.15E-24 |
| GRAMD1B | -2.44819 | 3.416987 | 4.57E-26 | 1.20E-24 |
| INPP5J | -2.65839 | 4.198111 | 5.90E-26 | 1.55E-24 |
| ETV6 | 1.039879 | 5.659051 | 6.14E-26 | 1.61E-24 |
| PNPLA3 | -2.78741 | 0.369526 | 6.22E-26 | 1.62E-24 |
| TRIB2 | -1.59906 | 4.924101 | 6.24E-26 | 1.63E-24 |
| SLIT3 | -2.32884 | 4.911832 | 6.64E-26 | 1.73E-24 |
| HMGB3 | 1.618883 | 5.833458 | 7.30E-26 | 1.90E-24 |
| PLCD3 | 2.485025 | 7.144244 | 7.74E-26 | 2.01E-24 |
| GLTPD2 | -2.22761 | 2.092031 | 8.94E-26 | 2.32E-24 |
| HSPB8 | 2.109774 | 7.88499 | 9.00E-26 | 2.33E-24 |
| DES | -3.75346 | 3.585404 | 9.63E-26 | 2.49E-24 |
| HECW1 | -2.95717 | 2.807272 | 9.97E-26 | 2.57E-24 |
| KLHL3 | -2.37236 | 3.23124 | 1.02E-25 | 2.62E-24 |
| CDC42EP3 | -1.30193 | 4.807694 | 1.04E-25 | 2.67E-24 |
| NR4A1 | -2.83291 | 6.679116 | 1.10E-25 | 2.83E-24 |
| PRAM1 | 2.743025 | 2.890901 | 1.14E-25 | 2.92E-24 |
| CDC6 | 3.003533 | 2.421794 | 1.25E-25 | 3.20E-24 |
| PFN1 | 1.14544 | 9.431181 | 1.25E-25 | 3.21E-24 |
| DBT | -1.04624 | 5.374023 | 1.28E-25 | 3.26E-24 |
| TGFBR3 | -1.70754 | 5.440927 | 1.32E-25 | 3.35E-24 |
| PARP8 | 1.020721 | 5.063038 | 1.36E-25 | 3.46E-24 |
| BPI | -2.87795 | -0.15977 | 1.39E-25 | 3.53E-24 |
| NOP2 | 1.139901 | 5.253283 | 1.43E-25 | 3.62E-24 |
| MAP7D3 | 1.516524 | 4.836979 | 1.51E-25 | 3.83E-24 |
| MGAT5 | -1.10956 | 5.976593 | 1.67E-25 | 4.22E-24 |
| ARL6IP5 | 1.23317 | 7.974693 | 1.82E-25 | 4.58E-24 |
| PLD2 | 1.247847 | 5.555896 | 1.85E-25 | 4.67E-24 |
| GSTA2 | -4.19753 | 4.485745 | 1.87E-25 | 4.70E-24 |
| SLC45A4 | -1.13616 | 5.329186 | 2.00E-25 | 5.01E-24 |
| ADD3 | -1.17923 | 6.519313 | 2.38E-25 | 5.98E-24 |
| TRAIP | 1.47057 | 1.876697 | 2.63E-25 | 6.59E-24 |
| DNM3 | -1.90474 | 2.006011 | 2.69E-25 | 6.73E-24 |
| FCHSD1 | 1.396403 | 4.410983 | 2.80E-25 | 6.98E-24 |
| ADGRL4 | -1.84943 | 3.467514 | 2.80E-25 | 6.98E-24 |
| TYMP | 2.384965 | 6.400786 | 2.82E-25 | 7.03E-24 |
| PID1 | -2.00044 | 1.166881 | 2.89E-25 | 7.17E-24 |
| NR2C2AP | 1.267858 | 4.271403 | 2.99E-25 | 7.43E-24 |
| CORO7 | 1.793589 | 4.575054 | 3.31E-25 | 8.21E-24 |
| P4HB | 1.178873 | 9.865531 | 3.42E-25 | 8.46E-24 |
| LIG1 | 1.076398 | 5.05646 | 3.64E-25 | 8.98E-24 |
| TNFAIP6 | 5.510415 | 4.617915 | 3.71E-25 | 9.14E-24 |
| LSM8 | 1.12951 | 4.541925 | 4.98E-25 | 1.23E-23 |
| C1orf186 | 3.393834 | 7.395634 | 5.09E-25 | 1.25E-23 |
| TRAF2 | 1.217802 | 4.826049 | 5.09E-25 | 1.25E-23 |
| FANCA | 1.972276 | 2.750082 | 5.12E-25 | 1.26E-23 |
| PTPRO | -2.22559 | 4.601224 | 5.31E-25 | 1.30E-23 |
| C10orf55 | -2.15252 | -0.39756 | 5.41E-25 | 1.32E-23 |
| SCARB1 | 2.258967 | 6.096059 | 6.06E-25 | 1.48E-23 |
| LRIT3 | -2.17976 | -1.11235 | 6.18E-25 | 1.51E-23 |
| TRIM9 | 2.726057 | 3.617614 | 6.26E-25 | 1.52E-23 |
| APBB1IP | 2.800971 | 6.093859 | 6.69E-25 | 1.63E-23 |
| PCK1 | -3.26733 | 8.036715 | 6.70E-25 | 1.63E-23 |
| ARPC3 | 1.031282 | 7.760238 | 7.10E-25 | 1.72E-23 |
| NDST3 | -3.01066 | -1.34237 | 7.22E-25 | 1.75E-23 |
| DNAJC11 | -1.12568 | 5.294238 | 7.38E-25 | 1.79E-23 |
| UHRF1 | 2.697228 | 1.787319 | 7.60E-25 | 1.84E-23 |
| EME1 | 2.59848 | 0.721449 | 7.75E-25 | 1.87E-23 |
| TNNI1 | -2.66482 | 2.017741 | 7.79E-25 | 1.88E-23 |
| SH2D3C | -1.59177 | 3.408409 | 8.31E-25 | 2.00E-23 |
| HPCA | 3.136499 | 1.133265 | 8.59E-25 | 2.06E-23 |
| ITGA9 | -1.6164 | 3.215664 | 9.13E-25 | 2.19E-23 |
| HMX3 | -4.22814 | -4.03278 | 9.28E-25 | 2.22E-23 |
| SIGLEC8 | 5.262141 | 3.219618 | 1.05E-24 | 2.51E-23 |
| CXCL16 | 1.684133 | 7.22307 | 1.05E-24 | 2.51E-23 |
| LRRC37B | 1.142507 | 2.88182 | 1.06E-24 | 2.54E-23 |
| RASSF9 | -2.51902 | 1.121162 | 1.10E-24 | 2.62E-23 |
| FAAP24 | 1.267477 | 1.824172 | 1.18E-24 | 2.81E-23 |
| DYRK2 | 1.25508 | 5.594691 | 1.19E-24 | 2.83E-23 |
| RPL22L1 | 2.220114 | 4.876529 | 1.24E-24 | 2.93E-23 |
| EPB41L4B | -2.56485 | 2.554662 | 1.30E-24 | 3.08E-23 |
| MUC12 | 6.059508 | 3.160419 | 1.40E-24 | 3.31E-23 |
| FRY | -1.63113 | 4.848161 | 1.59E-24 | 3.74E-23 |
| CXCL12 | -2.17382 | 6.11236 | 1.61E-24 | 3.79E-23 |
| CD68 | 2.866556 | 2.081284 | 1.76E-24 | 4.15E-23 |
| AL512785.2 | -2.6241 | -3.4088 | 1.78E-24 | 4.18E-23 |
| PEAR1 | -1.8378 | 2.648621 | 1.87E-24 | 4.39E-23 |
| TLDC1 | 1.310997 | 5.305825 | 2.01E-24 | 4.71E-23 |
| KCNV1 | 4.268006 | 2.826794 | 2.12E-24 | 4.95E-23 |
| SOX18 | -1.96944 | 2.531778 | 2.38E-24 | 5.56E-23 |
| PLA2G7 | 4.210925 | 3.215742 | 2.44E-24 | 5.70E-23 |
| C1orf168 | -3.04837 | 1.173554 | 2.63E-24 | 6.13E-23 |
| CCSER1 | -2.06983 | 1.653181 | 2.67E-24 | 6.23E-23 |
| ATP4B | -2.65424 | -2.10722 | 2.71E-24 | 6.30E-23 |
| APOBEC3C | 1.987089 | 5.640434 | 2.71E-24 | 6.30E-23 |
| CYBA | 1.922482 | 8.15373 | 2.86E-24 | 6.64E-23 |
| SPON2 | 3.43677 | 8.091249 | 2.94E-24 | 6.81E-23 |
| FGF9 | -3.22536 | 2.721016 | 2.95E-24 | 6.83E-23 |
| FREM1 | -2.3441 | 3.730726 | 2.98E-24 | 6.88E-23 |
| C3orf67 | 2.426673 | 2.730379 | 3.07E-24 | 7.09E-23 |
| ARHGAP18 | -1.05208 | 5.316575 | 3.24E-24 | 7.46E-23 |
| BRSK1 | 2.674069 | 3.326476 | 3.44E-24 | 7.92E-23 |
| KDELC1 | 2.281215 | 4.867489 | 3.45E-24 | 7.94E-23 |
| ZNF710 | -1.21037 | 3.789602 | 3.61E-24 | 8.28E-23 |
| GMIP | 1.35097 | 4.295399 | 3.63E-24 | 8.33E-23 |
| NTRK1 | -2.50878 | -0.65485 | 3.95E-24 | 9.03E-23 |
| RDM1 | 3.247551 | -0.39896 | 3.95E-24 | 9.03E-23 |
| MAN1C1 | -2.56349 | 4.451238 | 4.21E-24 | 9.62E-23 |
| TMEM43 | 1.331158 | 7.529883 | 4.22E-24 | 9.63E-23 |
| CHRNA1 | 7.099365 | 1.516847 | 4.26E-24 | 9.70E-23 |
| NAP1L5 | -1.5746 | 2.446789 | 4.32E-24 | 9.82E-23 |
| PIMREG | 3.043078 | 0.921728 | 4.46E-24 | 1.01E-22 |
| LRRC46 | 2.161326 | 2.579749 | 4.83E-24 | 1.09E-22 |
| NUDT1 | 1.699767 | 3.643633 | 4.85E-24 | 1.10E-22 |
| FAM107A | -2.3363 | 4.690924 | 5.87E-24 | 1.33E-22 |
| SLC16A10 | -1.94069 | 2.849931 | 6.24E-24 | 1.41E-22 |
| CBARP | -1.81329 | 0.63258 | 6.77E-24 | 1.53E-22 |
| PAQR4 | 2.226303 | 3.112747 | 7.52E-24 | 1.69E-22 |
| BCL6B | -2.01675 | 2.572487 | 7.59E-24 | 1.71E-22 |
| RXFP1 | -1.98224 | 0.194238 | 8.32E-24 | 1.86E-22 |
| RAPGEF4 | -2.16473 | 1.698184 | 8.82E-24 | 1.97E-22 |
| C16orf89 | -3.39714 | 2.881067 | 9.60E-24 | 2.14E-22 |
| ANKRD13B | 2.339729 | 3.613982 | 9.68E-24 | 2.16E-22 |
| AURKB | 3.299756 | 1.504634 | 1.00E-23 | 2.23E-22 |
| MELTF | -2.53474 | 3.927195 | 1.03E-23 | 2.29E-22 |
| SERPINE2 | 3.347619 | 9.060163 | 1.11E-23 | 2.46E-22 |
| CADM1 | -1.27129 | 5.293232 | 1.12E-23 | 2.48E-22 |
| ERICH4 | -3.34576 | 0.37623 | 1.18E-23 | 2.60E-22 |
| FAM162B | -2.24482 | -1.35684 | 1.18E-23 | 2.60E-22 |
| NAGS | -1.76781 | 2.3576 | 1.21E-23 | 2.67E-22 |
| ERBB4 | -3.83547 | 3.287397 | 1.28E-23 | 2.81E-22 |
| UNC13C | -3.40901 | -0.8971 | 1.37E-23 | 3.00E-22 |
| NMUR2 | -3.57894 | -2.89579 | 1.39E-23 | 3.05E-22 |
| PAGE4 | -3.84175 | -3.70825 | 1.40E-23 | 3.06E-22 |
| E2F2 | 2.699168 | 0.621024 | 1.47E-23 | 3.22E-22 |
| PLAT | -2.31763 | 5.36647 | 1.56E-23 | 3.42E-22 |
| DNMBP | -1.05462 | 4.718927 | 1.80E-23 | 3.93E-22 |
| LYVE1 | -2.2213 | 2.238461 | 1.81E-23 | 3.95E-22 |
| MAPK4 | -2.69869 | 2.633463 | 1.83E-23 | 3.98E-22 |
| DNAJC5B | 4.717864 | 0.879717 | 1.86E-23 | 4.04E-22 |
| DDX25 | -2.51416 | -0.33366 | 1.92E-23 | 4.19E-22 |
| CPNE7 | 4.919364 | 4.616733 | 1.97E-23 | 4.28E-22 |
| CCL14 | -1.95135 | -0.14255 | 2.00E-23 | 4.35E-22 |
| SPC24 | 2.738955 | 1.904568 | 2.11E-23 | 4.57E-22 |
| TNFRSF1A | 1.051839 | 7.513076 | 2.27E-23 | 4.91E-22 |
| ZNF385A | 1.73233 | 5.66328 | 2.31E-23 | 4.99E-22 |
| MCUB | 1.980125 | 3.605904 | 2.35E-23 | 5.07E-22 |
| MUC3A | 4.229827 | 5.294204 | 2.40E-23 | 5.17E-22 |
| MYOM2 | -1.44972 | 3.190297 | 2.45E-23 | 5.28E-22 |
| SHBG | -1.64801 | 0.506931 | 2.47E-23 | 5.31E-22 |
| NTS | -4.24433 | -1.43523 | 2.60E-23 | 5.59E-22 |
| ASF1B | 2.683726 | 2.674034 | 2.69E-23 | 5.78E-22 |
| STON1 | -1.6027 | 3.212055 | 2.71E-23 | 5.81E-22 |
| MYBL2 | 3.785318 | 2.726243 | 2.76E-23 | 5.91E-22 |
| KIF5C | -2.2594 | 0.610535 | 2.96E-23 | 6.34E-22 |
| RFC4 | 1.160695 | 3.518443 | 2.99E-23 | 6.39E-22 |
| PRKN | -1.62321 | 3.790915 | 3.08E-23 | 6.58E-22 |
| RHOBTB3 | -1.89304 | 5.039519 | 3.16E-23 | 6.73E-22 |
| SFXN3 | 1.068955 | 6.331418 | 3.24E-23 | 6.89E-22 |
| MEIOC | -1.57837 | -0.97271 | 3.46E-23 | 7.36E-22 |
| ASNS | 1.6636 | 5.070386 | 3.70E-23 | 7.87E-22 |
| GFRA3 | -2.868 | -2.25122 | 3.71E-23 | 7.87E-22 |
| MNX1 | 4.415841 | 1.218124 | 3.77E-23 | 7.99E-22 |
| ZBTB21 | -1.07926 | 3.999654 | 4.06E-23 | 8.60E-22 |
| CCND2 | 2.465249 | 7.124567 | 4.34E-23 | 9.17E-22 |
| SPAG4 | 2.36651 | 3.937813 | 4.63E-23 | 9.78E-22 |
| BTN2A2 | 1.069939 | 4.542957 | 4.67E-23 | 9.84E-22 |
| SPATS2L | 1.280826 | 8.079526 | 5.01E-23 | 1.05E-21 |
| PPP1R36 | -2.30003 | 0.11383 | 5.04E-23 | 1.06E-21 |
| ZC3HC1 | 1.036038 | 5.16483 | 5.09E-23 | 1.07E-21 |
| VAT1 | 1.389819 | 8.951784 | 5.23E-23 | 1.10E-21 |
| SPHK2 | -1.09595 | 4.598813 | 5.44E-23 | 1.14E-21 |
| HRH1 | 2.325184 | 4.997211 | 5.48E-23 | 1.14E-21 |
| CHRM3 | -2.57268 | 0.716284 | 5.48E-23 | 1.14E-21 |
| KCNC4 | -1.86648 | 3.675666 | 5.59E-23 | 1.17E-21 |
| MYOCD | -2.71714 | 0.019911 | 5.81E-23 | 1.21E-21 |
| DAO | -2.90766 | 4.011297 | 5.82E-23 | 1.21E-21 |
| LHFPL5 | 4.072909 | -1.76668 | 5.91E-23 | 1.23E-21 |
| LMO2 | -1.11519 | 4.339476 | 6.24E-23 | 1.30E-21 |
| TNNC1 | -2.5014 | 1.002545 | 6.40E-23 | 1.33E-21 |
| TWF2 | 1.190745 | 5.981623 | 6.88E-23 | 1.42E-21 |
| CDA | -2.27474 | 1.471438 | 6.93E-23 | 1.43E-21 |
| TROAP | 3.350227 | 1.384286 | 7.14E-23 | 1.47E-21 |
| SLC13A3 | -3.69211 | 7.053178 | 7.33E-23 | 1.51E-21 |
| APOH | -3.27894 | 1.250426 | 7.91E-23 | 1.63E-21 |
| FOLR3 | -3.47318 | 0.436299 | 8.02E-23 | 1.65E-21 |
| ZMYND15 | 1.936283 | 3.094205 | 8.25E-23 | 1.70E-21 |
| NTNG1 | -2.97521 | 1.925344 | 9.61E-23 | 1.97E-21 |
| IYD | -2.54595 | 4.082947 | 9.67E-23 | 1.99E-21 |
| MVP | 1.41072 | 9.335089 | 1.01E-22 | 2.07E-21 |
| MARCKS | 1.373533 | 7.323335 | 1.06E-22 | 2.17E-21 |
| NARF | 1.26846 | 5.948261 | 1.09E-22 | 2.22E-21 |
| ISX | -3.04927 | -3.55453 | 1.13E-22 | 2.30E-21 |
| ALOX15B | 5.805734 | 3.310515 | 1.13E-22 | 2.30E-21 |
| GPR12 | -3.3166 | -4.16699 | 1.15E-22 | 2.34E-21 |
| CRYAB | 2.745996 | 11.69932 | 1.15E-22 | 2.34E-21 |
| NUTF2 | 1.092239 | 6.693782 | 1.17E-22 | 2.38E-21 |
| CYB5A | -1.31027 | 7.178992 | 1.19E-22 | 2.42E-21 |
| RAMP2 | -1.67029 | 3.399597 | 1.20E-22 | 2.43E-21 |
| GIMAP6 | -1.71323 | 3.677094 | 1.21E-22 | 2.44E-21 |
| SERPINH1 | 1.303081 | 7.569186 | 1.22E-22 | 2.46E-21 |
| PFN3 | -3.22075 | -3.58916 | 1.28E-22 | 2.57E-21 |
| SIRT7 | 1.198388 | 4.785002 | 1.34E-22 | 2.70E-21 |
| ITPR1 | -1.84218 | 5.204314 | 1.35E-22 | 2.72E-21 |
| BCO2 | 2.267231 | 3.242488 | 1.41E-22 | 2.83E-21 |
| ZNF503 | -1.43729 | 5.060789 | 1.47E-22 | 2.94E-21 |
| CABYR | 2.229074 | 0.981176 | 1.48E-22 | 2.97E-21 |
| PCYOX1 | -1.13905 | 7.180746 | 1.52E-22 | 3.03E-21 |
| CTSC | 1.78357 | 9.616637 | 1.67E-22 | 3.33E-21 |
| ZYX | 1.18833 | 8.126196 | 1.68E-22 | 3.35E-21 |
| PLEKHN1 | 3.188076 | 3.013007 | 1.73E-22 | 3.43E-21 |
| KLF9 | -1.21526 | 6.23485 | 1.79E-22 | 3.56E-21 |
| RAET1E | -3.0237 | -0.87705 | 2.07E-22 | 4.10E-21 |
| IQSEC3 | -2.44694 | 1.782546 | 2.10E-22 | 4.15E-21 |
| VAT1L | -3.78665 | 1.490997 | 2.16E-22 | 4.26E-21 |
| APCDD1L | -3.61904 | 1.168515 | 2.20E-22 | 4.33E-21 |
| LRRC32 | -1.71714 | 5.522236 | 2.30E-22 | 4.53E-21 |
| CELSR3 | 2.478376 | 2.461309 | 2.31E-22 | 4.54E-21 |
| ORAI3 | 1.495358 | 5.403293 | 2.33E-22 | 4.57E-21 |
| MPZL1 | 1.06983 | 7.240164 | 2.33E-22 | 4.58E-21 |
| SNAP25 | 2.841738 | 3.385917 | 2.51E-22 | 4.92E-21 |
| ZNF726 | -1.61901 | 0.174814 | 2.52E-22 | 4.93E-21 |
| SYTL4 | -1.26686 | 3.410624 | 2.54E-22 | 4.97E-21 |
| S1PR3 | -1.98448 | 3.621792 | 2.55E-22 | 4.98E-21 |
| CDH5 | -1.70685 | 4.482531 | 2.55E-22 | 4.98E-21 |
| RGS1 | 3.086021 | 6.200332 | 2.66E-22 | 5.17E-21 |
| MCM7 | 1.201079 | 6.43626 | 2.75E-22 | 5.35E-21 |
| PLPPR5 | 6.521096 | 2.748901 | 2.78E-22 | 5.40E-21 |
| CRACR2A | 2.322368 | 2.277563 | 2.80E-22 | 5.43E-21 |
| SLC22A23 | -1.80993 | 3.265678 | 3.20E-22 | 6.20E-21 |
| SSH2 | 1.294967 | 6.295642 | 3.20E-22 | 6.20E-21 |
| SYCE1L | 3.635872 | 3.377763 | 3.21E-22 | 6.22E-21 |
| ACADM | -1.34124 | 6.580421 | 3.29E-22 | 6.36E-21 |
| SRCIN1 | 2.958557 | 3.70236 | 3.31E-22 | 6.38E-21 |
| PLN | -2.60751 | 2.163071 | 3.39E-22 | 6.53E-21 |
| PTPN6 | 1.08004 | 5.971971 | 3.41E-22 | 6.57E-21 |
| TGFBR2 | -1.18372 | 7.130236 | 3.47E-22 | 6.67E-21 |
| B4GALT5 | 1.55056 | 8.113198 | 3.47E-22 | 6.67E-21 |
| LILRB4 | 3.265439 | 4.620572 | 3.57E-22 | 6.85E-21 |
| ADH6 | -2.60218 | 3.801855 | 3.63E-22 | 6.96E-21 |
| DONSON | 1.421142 | 3.415037 | 3.78E-22 | 7.25E-21 |
| PINK1 | -1.21247 | 5.409097 | 3.79E-22 | 7.25E-21 |
| SYT12 | 3.846328 | 3.806058 | 3.84E-22 | 7.34E-21 |
| BIVM | 1.369174 | 6.084227 | 3.90E-22 | 7.44E-21 |
| NDRG2 | -1.25667 | 6.828018 | 4.17E-22 | 7.95E-21 |
| MYOZ1 | -2.14867 | 0.200912 | 4.29E-22 | 8.17E-21 |
| PRDM12 | 2.977107 | -2.22127 | 4.55E-22 | 8.66E-21 |
| RPS6KA6 | -1.24799 | 3.892834 | 4.70E-22 | 8.94E-21 |
| KIAA2022 | -2.91965 | 0.657384 | 4.98E-22 | 9.47E-21 |
| WDR72 | -1.79257 | 6.285742 | 5.17E-22 | 9.81E-21 |
| FER1L6 | -3.33592 | 0.806952 | 5.24E-22 | 9.93E-21 |
| APOM | -2.14346 | 4.743691 | 5.36E-22 | 1.01E-20 |
| SHE | -1.48211 | 3.034218 | 5.62E-22 | 1.06E-20 |
| ASAP2 | -1.78982 | 4.428949 | 5.65E-22 | 1.07E-20 |
| DACH1 | -2.91871 | 2.753248 | 5.65E-22 | 1.07E-20 |
| NKX6-2 | -2.79878 | -3.93618 | 5.66E-22 | 1.07E-20 |
| CRB2 | -3.21685 | 2.419385 | 5.67E-22 | 1.07E-20 |
| SLC16A5 | -2.37409 | 4.280417 | 5.77E-22 | 1.09E-20 |
| RAP2B | 1.695544 | 5.805441 | 6.48E-22 | 1.22E-20 |
| DLX4 | 2.948125 | 0.177766 | 6.53E-22 | 1.23E-20 |
| TNIK | 1.705579 | 5.81899 | 6.68E-22 | 1.25E-20 |
| TMEM54 | 1.889865 | 6.317188 | 6.93E-22 | 1.30E-20 |
| TUSC3 | 1.142901 | 6.590925 | 7.14E-22 | 1.34E-20 |
| EFCC1 | -1.62014 | 0.470447 | 7.16E-22 | 1.34E-20 |
| BAZ1A | 1.171003 | 4.723779 | 7.21E-22 | 1.35E-20 |
| CNTN6 | 5.607134 | 5.345552 | 7.61E-22 | 1.42E-20 |
| C9orf116 | 1.588387 | 3.697402 | 8.00E-22 | 1.49E-20 |
| ECI2 | -1.15527 | 6.269005 | 8.04E-22 | 1.50E-20 |
| RASIP1 | -1.73758 | 3.117449 | 8.31E-22 | 1.54E-20 |
| PTGER3 | -4.20984 | 5.418693 | 8.40E-22 | 1.56E-20 |
| EMILIN3 | -1.99906 | -1.96908 | 8.82E-22 | 1.64E-20 |
| IBA57 | -1.01655 | 3.665033 | 8.96E-22 | 1.66E-20 |
| PRKAA2 | -1.11461 | 5.354144 | 8.97E-22 | 1.66E-20 |
| PDGFB | -1.2658 | 4.327318 | 9.39E-22 | 1.74E-20 |
| PARM1 | -2.29618 | 5.455281 | 1.03E-21 | 1.90E-20 |
| WEE2 | -1.81958 | -2.09324 | 1.04E-21 | 1.91E-20 |
| PCLAF | 2.729477 | 2.266141 | 1.16E-21 | 2.14E-20 |
| XYLT2 | 1.027689 | 6.277222 | 1.18E-21 | 2.16E-20 |
| CTH | -1.53866 | 2.573996 | 1.20E-21 | 2.21E-20 |
| MTUS1 | -1.42135 | 5.077171 | 1.22E-21 | 2.24E-20 |
| FAM234B | -1.18998 | 3.949028 | 1.22E-21 | 2.25E-20 |
| TMEM163 | 3.414546 | 4.649222 | 1.25E-21 | 2.29E-20 |
| MYH11 | -2.5282 | 6.153143 | 1.28E-21 | 2.34E-20 |
| IGFBP6 | 3.35895 | 7.530804 | 1.30E-21 | 2.38E-20 |
| GIPC3 | -1.76322 | 1.626511 | 1.39E-21 | 2.54E-20 |
| PI16 | -3.41432 | 0.190248 | 1.46E-21 | 2.66E-20 |
| HPGD | -2.49144 | 3.902636 | 1.47E-21 | 2.68E-20 |
| DYNLT3 | -1.04141 | 4.843539 | 1.57E-21 | 2.86E-20 |
| DPEP1 | -2.87161 | 6.608256 | 1.64E-21 | 2.98E-20 |
| HMCN2 | -2.07399 | 1.662707 | 1.65E-21 | 2.99E-20 |
| BDKRB2 | -2.81088 | 3.598224 | 1.67E-21 | 3.02E-20 |
| CCL18 | 7.403618 | 6.074269 | 1.73E-21 | 3.13E-20 |
| ATP6V0A4 | -3.43304 | 5.289273 | 1.76E-21 | 3.17E-20 |
| CELF3 | -2.40961 | -2.57986 | 1.79E-21 | 3.22E-20 |
| CKLF | 1.526378 | 2.860763 | 1.90E-21 | 3.43E-20 |
| CADPS2 | -1.23726 | 5.428303 | 1.99E-21 | 3.58E-20 |
| GABRA2 | -4.25441 | 1.231769 | 2.02E-21 | 3.63E-20 |
| PDZRN4 | -2.63512 | -0.74341 | 2.05E-21 | 3.69E-20 |
| POLE2 | 1.530233 | 1.053866 | 2.06E-21 | 3.71E-20 |
| SRGAP2C | -1.24244 | 2.130284 | 2.07E-21 | 3.72E-20 |
| HIST3H2A | 2.864713 | 2.868202 | 2.09E-21 | 3.75E-20 |
| MTFR2 | 1.976063 | 0.287903 | 2.16E-21 | 3.87E-20 |
| IKBKE | 1.333147 | 3.930356 | 2.18E-21 | 3.89E-20 |
| NRGN | -1.54384 | 2.188403 | 2.19E-21 | 3.91E-20 |
| VMP1 | 1.807208 | 8.113796 | 2.25E-21 | 4.01E-20 |
| CLDN5 | -1.70542 | 3.573733 | 2.49E-21 | 4.44E-20 |
| CATSPER1 | 3.285514 | 0.703966 | 2.52E-21 | 4.49E-20 |
| 2-Mar | -1.28079 | 5.435699 | 2.55E-21 | 4.54E-20 |
| MPG | 1.156029 | 5.087603 | 2.57E-21 | 4.57E-20 |
| PBLD | -1.8628 | 6.373809 | 2.88E-21 | 5.11E-20 |
| KIAA0040 | -1.08338 | 5.14681 | 3.05E-21 | 5.42E-20 |
| TMEM44 | 1.532759 | 4.223549 | 3.06E-21 | 5.43E-20 |
| RAB6B | 1.660852 | 5.784996 | 3.09E-21 | 5.47E-20 |
| BRPF3 | -1.02988 | 5.454991 | 3.11E-21 | 5.49E-20 |
| HS3ST2 | 7.042447 | 4.662365 | 3.11E-21 | 5.49E-20 |
| WNK3 | -2.04569 | 1.218827 | 3.19E-21 | 5.63E-20 |
| SQOR | 1.27094 | 5.778645 | 3.21E-21 | 5.65E-20 |
| SNRPB | 1.056532 | 7.172202 | 3.23E-21 | 5.69E-20 |
| TP53INP1 | 1.300257 | 6.110845 | 3.34E-21 | 5.88E-20 |
| IQGAP3 | 2.855064 | 3.026704 | 3.38E-21 | 5.94E-20 |
| PCDH18 | -2.22634 | 1.629127 | 3.50E-21 | 6.15E-20 |
| GGH | -1.61633 | 4.450095 | 3.60E-21 | 6.31E-20 |
| FHL5 | -2.56279 | 0.573216 | 3.77E-21 | 6.61E-20 |
| DLC1 | -1.29455 | 4.93253 | 4.09E-21 | 7.14E-20 |
| CD70 | 5.359337 | 3.547641 | 4.11E-21 | 7.17E-20 |
| DTNA | 1.817367 | 4.518811 | 4.19E-21 | 7.31E-20 |
| ARHGEF39 | 1.917819 | 1.973287 | 4.19E-21 | 7.31E-20 |
| SVOPL | -2.67345 | -0.07548 | 4.25E-21 | 7.40E-20 |
| COL23A1 | 3.29242 | 5.374226 | 4.29E-21 | 7.47E-20 |
| TRIM38 | 1.202505 | 6.731899 | 4.31E-21 | 7.50E-20 |
| C2orf54 | -3.44091 | 2.252917 | 4.33E-21 | 7.52E-20 |
| CLEC5A | 2.720849 | 2.058921 | 4.38E-21 | 7.59E-20 |
| PLK1 | 2.280317 | 2.295256 | 4.57E-21 | 7.91E-20 |
| PSMB9 | 1.781698 | 5.587243 | 4.62E-21 | 8.00E-20 |
| AK4 | -1.74075 | 7.029667 | 4.66E-21 | 8.05E-20 |
| CACNB3 | 1.847748 | 5.082175 | 4.79E-21 | 8.28E-20 |
| PAQR7 | -1.00794 | 5.696603 | 4.86E-21 | 8.38E-20 |
| GFRA2 | -1.89884 | -0.11734 | 5.23E-21 | 9.02E-20 |
| DTX2 | 1.232093 | 4.115865 | 5.29E-21 | 9.10E-20 |
| PRSS35 | -3.0298 | 0.650911 | 5.35E-21 | 9.19E-20 |
| DBNDD1 | 1.853218 | 6.229941 | 5.54E-21 | 9.52E-20 |
| ST3GAL1 | -1.30045 | 6.004424 | 5.65E-21 | 9.68E-20 |
| PDGFRA | -2.6526 | 3.423615 | 5.75E-21 | 9.85E-20 |
| SVEP1 | -2.30622 | 3.325565 | 5.76E-21 | 9.85E-20 |
| C1QTNF6 | 1.710343 | 4.13299 | 6.23E-21 | 1.07E-19 |
| C19orf33 | 3.440495 | 6.032198 | 6.39E-21 | 1.09E-19 |
| CMTM3 | 1.877159 | 5.687043 | 6.47E-21 | 1.10E-19 |
| TTC39A | 1.769022 | 6.091433 | 6.59E-21 | 1.12E-19 |
| CCNA2 | 2.193588 | 1.95064 | 6.81E-21 | 1.16E-19 |
| CORO6 | 3.691047 | 3.400359 | 6.86E-21 | 1.17E-19 |
| UAP1 | -1.05552 | 4.429196 | 6.87E-21 | 1.17E-19 |
| KIAA0930 | 1.199676 | 6.653318 | 6.96E-21 | 1.18E-19 |
| KIRREL2 | -3.26471 | -1.49766 | 7.06E-21 | 1.19E-19 |
| LIPE | -1.13698 | 2.391354 | 7.08E-21 | 1.20E-19 |
| FUT1 | -1.94645 | 1.024155 | 7.27E-21 | 1.23E-19 |
| ABCA12 | 3.77148 | 3.901947 | 7.35E-21 | 1.24E-19 |
| ADAMTS19 | -4.01187 | -1.90582 | 7.49E-21 | 1.26E-19 |
| KMT5C | 1.446648 | 3.560889 | 7.63E-21 | 1.29E-19 |
| RPS2 | 1.420946 | 9.511544 | 8.29E-21 | 1.40E-19 |
| TUBA1A | 1.455692 | 7.530381 | 8.78E-21 | 1.48E-19 |
| PIK3C2G | -4.12295 | 1.355292 | 8.82E-21 | 1.48E-19 |
| BSND | -4.78834 | 2.717533 | 8.84E-21 | 1.48E-19 |
| CD151 | 1.123149 | 9.13951 | 9.17E-21 | 1.54E-19 |
| AIF1L | -1.32984 | 8.835235 | 9.18E-21 | 1.54E-19 |
| FAM111B | 2.826961 | 1.538609 | 9.81E-21 | 1.64E-19 |
| RNASET2 | 2.314087 | 6.880551 | 9.90E-21 | 1.65E-19 |
| ABHD11 | 2.047889 | 7.017459 | 9.94E-21 | 1.66E-19 |
| SHROOM4 | -1.01879 | 4.294825 | 1.01E-20 | 1.68E-19 |
| TCTN1 | 1.041986 | 6.439424 | 1.09E-20 | 1.82E-19 |
| UBE2QL1 | -3.11113 | 1.431843 | 1.09E-20 | 1.82E-19 |
| C9orf172 | 1.765242 | 3.304199 | 1.10E-20 | 1.83E-19 |
| GAPDH | 1.16931 | 11.75836 | 1.11E-20 | 1.84E-19 |
| IGFBP2 | -2.09785 | 5.309259 | 1.21E-20 | 2.01E-19 |
| ANXA9 | -1.4965 | 4.094233 | 1.22E-20 | 2.03E-19 |
| MSRA | -1.64523 | 5.431744 | 1.26E-20 | 2.08E-19 |
| ADRB1 | -2.72349 | -0.90402 | 1.27E-20 | 2.11E-19 |
| CHIT1 | 7.101776 | 6.521301 | 1.29E-20 | 2.13E-19 |
| KAT2B | -1.08644 | 4.612898 | 1.31E-20 | 2.16E-19 |
| HSF4 | 2.713088 | 4.525531 | 1.37E-20 | 2.25E-19 |
| CEP55 | 3.061158 | 1.869652 | 1.55E-20 | 2.54E-19 |
| CASP4 | 1.105225 | 5.508969 | 1.60E-20 | 2.63E-19 |
| RPS19 | 1.527937 | 9.537993 | 1.63E-20 | 2.68E-19 |
| MMP11 | 2.494083 | 4.762545 | 1.64E-20 | 2.68E-19 |
| FANCI | 1.420368 | 3.425119 | 1.65E-20 | 2.71E-19 |
| SLC25A37 | 1.760238 | 6.564966 | 1.68E-20 | 2.75E-19 |
| P3H1 | 1.036837 | 5.191577 | 1.71E-20 | 2.80E-19 |
| HES6 | 1.668041 | 3.081508 | 1.71E-20 | 2.80E-19 |
| AQP4 | -2.10547 | 1.178609 | 1.80E-20 | 2.93E-19 |
| TREML1 | 3.009178 | -0.46267 | 1.88E-20 | 3.07E-19 |
| ZYG11A | -2.64046 | 0.060381 | 1.91E-20 | 3.10E-19 |
| RAB7B | 2.399346 | 2.819437 | 2.05E-20 | 3.34E-19 |
| MYOC | -2.43466 | -1.58147 | 2.15E-20 | 3.49E-19 |
| COL4A6 | -2.69417 | 2.345821 | 2.30E-20 | 3.72E-19 |
| TRAM1L1 | -1.50259 | 2.135814 | 2.31E-20 | 3.73E-19 |
| NSUN5 | 1.202469 | 5.286907 | 2.35E-20 | 3.80E-19 |
| CHTF18 | 1.689686 | 3.840797 | 2.39E-20 | 3.85E-19 |
| IL4R | 1.213892 | 6.042223 | 2.54E-20 | 4.08E-19 |
| FADS3 | 1.30741 | 5.329262 | 2.56E-20 | 4.12E-19 |
| PTGS2 | -2.25758 | 1.88423 | 2.56E-20 | 4.12E-19 |
| GK | -1.45612 | 4.641987 | 2.57E-20 | 4.13E-19 |
| FLRT2 | -2.31727 | 1.579503 | 2.65E-20 | 4.26E-19 |
| HOXA3 | 2.185956 | 5.636887 | 2.88E-20 | 4.62E-19 |
| CNGA1 | -2.58662 | 0.815653 | 2.91E-20 | 4.66E-19 |
| NAALAD2 | -1.45547 | 0.190147 | 2.94E-20 | 4.71E-19 |
| POLG2 | 1.504604 | 3.760585 | 3.02E-20 | 4.83E-19 |
| HSPA12A | -1.74595 | 4.703766 | 3.09E-20 | 4.94E-19 |
| UBE2C | 3.521253 | 2.368277 | 3.31E-20 | 5.27E-19 |
| PKHD1L1 | -2.08267 | -1.5744 | 3.61E-20 | 5.75E-19 |
| ZNF704 | -1.13699 | 5.249375 | 3.62E-20 | 5.76E-19 |
| BUB1 | 2.867165 | 2.085044 | 3.66E-20 | 5.82E-19 |
| LRRK2 | 2.368519 | 8.410445 | 3.88E-20 | 6.16E-19 |
| GRAP | -1.61877 | -0.49673 | 3.93E-20 | 6.23E-19 |
| SCN4A | -2.14166 | -0.80136 | 4.24E-20 | 6.72E-19 |
| MCM10 | 3.123666 | 0.650606 | 4.27E-20 | 6.76E-19 |
| GNG7 | -1.14407 | 4.606668 | 4.29E-20 | 6.79E-19 |
| TNNI3 | 6.277594 | 2.004913 | 4.33E-20 | 6.85E-19 |
| IGSF6 | 2.548549 | 4.026225 | 4.60E-20 | 7.26E-19 |
| SLC6A4 | -1.78371 | -0.54125 | 4.63E-20 | 7.31E-19 |
| C1orf115 | -1.02647 | 6.02526 | 4.74E-20 | 7.46E-19 |
| TRADD | 1.211184 | 5.440547 | 4.77E-20 | 7.52E-19 |
| GPC2 | 2.733502 | 0.369078 | 4.82E-20 | 7.58E-19 |
| AC104831.1 | -3.0068 | -2.24612 | 4.83E-20 | 7.59E-19 |
| ENOX1 | -1.86104 | 1.306653 | 4.87E-20 | 7.64E-19 |
| CDKL1 | -1.59234 | 2.791612 | 4.92E-20 | 7.71E-19 |
| GTF3A | 1.095388 | 6.698065 | 5.02E-20 | 7.86E-19 |
| KRT8 | 1.763953 | 10.13856 | 5.05E-20 | 7.90E-19 |
| CENPA | 3.211978 | 0.15065 | 5.05E-20 | 7.90E-19 |
| PADI2 | -1.72583 | 4.524289 | 5.07E-20 | 7.91E-19 |
| FMNL2 | 1.189992 | 6.56033 | 5.22E-20 | 8.14E-19 |
| GM2A | 1.626702 | 6.950951 | 5.39E-20 | 8.39E-19 |
| LAT2 | 1.88016 | 3.733735 | 5.46E-20 | 8.49E-19 |
| CPEB4 | -1.30708 | 5.960578 | 5.79E-20 | 9.00E-19 |
| TRMT10A | -1.01041 | 1.897036 | 5.85E-20 | 9.09E-19 |
| BIRC5 | 3.096217 | 2.641137 | 5.90E-20 | 9.15E-19 |
| FZD1 | 1.887079 | 7.823871 | 6.02E-20 | 9.33E-19 |
| PDE3A | -2.08721 | 2.908702 | 6.15E-20 | 9.53E-19 |
| PNMA2 | 2.362598 | 5.388647 | 6.22E-20 | 9.62E-19 |
| TWNK | 1.059472 | 4.218998 | 6.29E-20 | 9.73E-19 |
| OAT | -1.25043 | 5.81902 | 6.41E-20 | 9.90E-19 |
| MICAL1 | 1.374597 | 5.788657 | 6.57E-20 | 1.01E-18 |
| TBC1D1 | -1.02533 | 6.760032 | 6.66E-20 | 1.03E-18 |
| KIAA1841 | 1.247413 | 3.051771 | 6.67E-20 | 1.03E-18 |
| AC009779.3 | 2.5683 | 2.879266 | 6.94E-20 | 1.07E-18 |
| GSS | 1.312886 | 7.427785 | 6.97E-20 | 1.07E-18 |
| FAM109A | 1.442056 | 5.151756 | 7.05E-20 | 1.08E-18 |
| DTL | 2.842495 | 2.087976 | 7.14E-20 | 1.10E-18 |
| TPX2 | 2.834347 | 3.293767 | 7.26E-20 | 1.11E-18 |
| TMEM178B | 2.216736 | 5.963326 | 7.32E-20 | 1.12E-18 |
| FAM89A | -1.2049 | 2.675442 | 7.57E-20 | 1.16E-18 |
| STMN3 | 2.526117 | 6.758794 | 7.59E-20 | 1.16E-18 |
| UBE2T | 1.7554 | 2.20839 | 7.80E-20 | 1.19E-18 |
| BLVRA | 1.222357 | 6.312804 | 7.93E-20 | 1.21E-18 |
| CES3 | -2.04702 | 3.322955 | 8.27E-20 | 1.26E-18 |
| THEMIS2 | 2.216365 | 5.369894 | 8.32E-20 | 1.26E-18 |
| DACT3 | -1.85101 | 1.547714 | 8.64E-20 | 1.31E-18 |
| CLDN10 | -1.88729 | 5.343113 | 8.84E-20 | 1.34E-18 |
| SACS | 1.721923 | 4.798312 | 9.04E-20 | 1.37E-18 |
| FAM69B | -1.95655 | 2.230931 | 9.22E-20 | 1.40E-18 |
| MAD1L1 | 1.25708 | 5.613953 | 9.24E-20 | 1.40E-18 |
| PNP | -1.08359 | 6.822332 | 9.25E-20 | 1.40E-18 |
| PPP1R14B | 1.448348 | 6.210031 | 9.83E-20 | 1.48E-18 |
| FRMD1 | -2.89007 | 1.478213 | 1.02E-19 | 1.54E-18 |
| SEM1 | 1.411718 | 6.460295 | 1.02E-19 | 1.54E-18 |
| DACH2 | -3.78806 | -1.91055 | 1.02E-19 | 1.54E-18 |
| PHACTR1 | -1.58927 | 3.623559 | 1.08E-19 | 1.62E-18 |
| ACO1 | -1.22086 | 6.757531 | 1.09E-19 | 1.64E-18 |
| CLUL1 | -1.86374 | 1.159535 | 1.19E-19 | 1.79E-18 |
| GPX8 | 1.392433 | 5.194301 | 1.25E-19 | 1.88E-18 |
| RCC1 | 1.175948 | 4.773442 | 1.29E-19 | 1.94E-18 |
| CGN | -1.77159 | 5.098836 | 1.38E-19 | 2.06E-18 |
| ASPHD2 | -1.54774 | 1.879061 | 1.38E-19 | 2.06E-18 |
| BTG2 | -1.4555 | 7.373549 | 1.39E-19 | 2.08E-18 |
| SLC7A9 | -2.70949 | 4.170459 | 1.40E-19 | 2.10E-18 |
| ZMIZ2 | 1.130762 | 7.670176 | 1.43E-19 | 2.13E-18 |
| NUDT4 | -1.20002 | 5.784947 | 1.48E-19 | 2.20E-18 |
| LPAR5 | 2.317978 | 2.862513 | 1.53E-19 | 2.28E-18 |
| NME2 | 1.301008 | 6.494913 | 1.53E-19 | 2.28E-18 |
| PGR | -2.62026 | 0.853729 | 1.65E-19 | 2.44E-18 |
| C11orf54 | -1.4037 | 7.301985 | 1.66E-19 | 2.47E-18 |
| GPRC5B | -1.90611 | 5.609911 | 1.76E-19 | 2.61E-18 |
| PIK3R6 | 2.396205 | 1.644118 | 1.84E-19 | 2.72E-18 |
| TBRG4 | 1.007218 | 6.649443 | 1.84E-19 | 2.73E-18 |
| SLC7A14 | -3.3707 | -2.48856 | 1.89E-19 | 2.80E-18 |
| RAB42 | 3.878087 | 3.764492 | 2.00E-19 | 2.95E-18 |
| AL138826.1 | -4.48526 | 0.553294 | 2.09E-19 | 3.08E-18 |
| KIF18B | 3.181544 | 1.152964 | 2.12E-19 | 3.12E-18 |
| ACOX2 | -1.82027 | 4.507585 | 2.14E-19 | 3.15E-18 |
| C17orf53 | 1.75716 | 1.282623 | 2.18E-19 | 3.21E-18 |
| RPL38 | 1.501686 | 8.590455 | 2.20E-19 | 3.23E-18 |
| HJURP | 3.264223 | 1.361638 | 2.23E-19 | 3.27E-18 |
| CCDC74A | 1.882997 | 4.079073 | 2.26E-19 | 3.31E-18 |
| MDM2 | 1.05998 | 6.658701 | 2.30E-19 | 3.38E-18 |
| CENPU | 1.956621 | 2.153548 | 2.38E-19 | 3.48E-18 |
| TP53I13 | 1.377058 | 6.116625 | 2.41E-19 | 3.52E-18 |
| CCDC178 | -2.22972 | 0.54527 | 2.42E-19 | 3.54E-18 |
| RASGEF1C | 3.273765 | 1.323231 | 2.47E-19 | 3.61E-18 |
| HOXA1 | 1.535075 | 1.777057 | 2.49E-19 | 3.63E-18 |
| SPHK1 | 2.945914 | 4.741436 | 2.57E-19 | 3.75E-18 |
| LRRN4 | 3.127971 | 6.21819 | 2.58E-19 | 3.76E-18 |
| PDE7B | -1.413 | 3.250145 | 2.62E-19 | 3.81E-18 |
| CCDC78 | 2.928584 | 2.695721 | 2.65E-19 | 3.85E-18 |
| RNPEPL1 | 1.097194 | 7.448811 | 2.71E-19 | 3.93E-18 |
| EGFL7 | -1.78603 | 4.282314 | 3.08E-19 | 4.47E-18 |
| B4GALNT3 | -2.43778 | 3.496993 | 3.26E-19 | 4.72E-18 |
| WDR49 | -2.59828 | -2.8273 | 3.32E-19 | 4.80E-18 |
| DPCD | 1.135948 | 5.166045 | 3.45E-19 | 4.98E-18 |
| IQGAP2 | -1.83287 | 5.260304 | 3.48E-19 | 5.03E-18 |
| PTTG1 | 2.597051 | 2.353015 | 3.51E-19 | 5.06E-18 |
| POLQ | 2.61459 | 0.638459 | 3.71E-19 | 5.35E-18 |
| UGT8 | -1.60461 | 5.026611 | 3.77E-19 | 5.44E-18 |
| MN1 | -1.72465 | 1.030786 | 3.86E-19 | 5.55E-18 |
| HLA-A | 1.585525 | 11.06587 | 4.07E-19 | 5.85E-18 |
| PLPP1 | -1.11834 | 5.3216 | 4.11E-19 | 5.91E-18 |
| EMILIN2 | 1.903926 | 4.413805 | 4.37E-19 | 6.26E-18 |
| RPGRIP1L | 1.076875 | 4.113852 | 4.44E-19 | 6.34E-18 |
| NRM | 1.361382 | 4.506304 | 4.46E-19 | 6.37E-18 |
| NEIL3 | 4.075004 | 0.122445 | 4.51E-19 | 6.44E-18 |
| TRAPPC1 | 1.134974 | 7.115362 | 4.53E-19 | 6.46E-18 |
| NEU1 | 1.414704 | 8.199247 | 4.76E-19 | 6.78E-18 |
| SLC22A13 | -2.76792 | 2.226263 | 4.82E-19 | 6.86E-18 |
| CTSZ | 1.675008 | 8.734119 | 4.86E-19 | 6.92E-18 |
| CHRNA6 | 2.860148 | -0.67984 | 4.87E-19 | 6.93E-18 |
| ASB9 | -1.49051 | 2.547366 | 5.14E-19 | 7.30E-18 |
| SGCZ | -3.97208 | -3.54037 | 5.30E-19 | 7.52E-18 |
| KCNJ1 | -4.15969 | 5.952185 | 5.52E-19 | 7.83E-18 |
| GALNT5 | 3.937346 | 3.929862 | 5.60E-19 | 7.94E-18 |
| TOP2A | 3.313301 | 4.271907 | 5.74E-19 | 8.13E-18 |
| APOBR | 2.191752 | 3.867555 | 5.89E-19 | 8.33E-18 |
| ARHGEF26 | -1.6729 | 1.357851 | 5.90E-19 | 8.33E-18 |
| ADH4 | -3.18065 | -2.15131 | 5.91E-19 | 8.34E-18 |
| RASSF8 | -1.06348 | 5.036193 | 5.95E-19 | 8.39E-18 |
| WBSCR28 | 3.661677 | -0.0938 | 6.16E-19 | 8.68E-18 |
| TMEM52B | -3.43662 | 5.560498 | 6.55E-19 | 9.21E-18 |
| CENPK | 2.093794 | 0.930023 | 6.56E-19 | 9.23E-18 |
| HTR3B | -3.91349 | -3.55425 | 6.59E-19 | 9.26E-18 |
| GAL3ST4 | 2.270131 | 3.275975 | 7.14E-19 | 1.00E-17 |
| TRIP13 | 2.068968 | 2.35492 | 7.31E-19 | 1.02E-17 |
| PRL | -3.48003 | -4.08905 | 7.53E-19 | 1.05E-17 |
| SPAAR | -1.73474 | 0.57293 | 7.90E-19 | 1.10E-17 |
| UGCG | 1.278077 | 7.344871 | 7.94E-19 | 1.11E-17 |
| NRBP2 | 1.791044 | 6.407247 | 8.16E-19 | 1.14E-17 |
| SLC37A2 | 3.168378 | 4.513633 | 8.34E-19 | 1.16E-17 |
| KCNG2 | -2.40783 | -2.53278 | 8.42E-19 | 1.17E-17 |
| KNTC1 | 1.299607 | 3.996164 | 8.46E-19 | 1.18E-17 |
| PC | -1.56334 | 5.769932 | 8.56E-19 | 1.19E-17 |
| ACSM1 | 2.657685 | 3.176937 | 8.57E-19 | 1.19E-17 |
| ABR | 1.267481 | 7.302281 | 8.79E-19 | 1.22E-17 |
| PRLR | -2.53313 | 4.263451 | 9.29E-19 | 1.29E-17 |
| WT1 | -3.52561 | 2.014834 | 1.02E-18 | 1.41E-17 |
| 6-Sep | -1.0536 | 4.894163 | 1.02E-18 | 1.41E-17 |
| GOLGA7B | 3.110451 | 1.642894 | 1.04E-18 | 1.43E-17 |
| SNX10 | 1.440788 | 6.598618 | 1.06E-18 | 1.46E-17 |
| ZNF804A | -1.88879 | -0.76668 | 1.06E-18 | 1.47E-17 |
| PPFIA2 | -1.99198 | -0.88337 | 1.08E-18 | 1.48E-17 |
| PLCH1 | 1.644658 | 4.250698 | 1.08E-18 | 1.48E-17 |
| OTOA | 3.9146 | 0.952756 | 1.09E-18 | 1.49E-17 |
| CDCA3 | 1.792948 | 2.032257 | 1.09E-18 | 1.50E-17 |
| PLXNA3 | 1.300978 | 5.416512 | 1.11E-18 | 1.53E-17 |
| PLEKHD1 | -2.66753 | 0.06561 | 1.20E-18 | 1.64E-17 |
| RGS17 | 2.549278 | 1.94126 | 1.22E-18 | 1.68E-17 |
| LTBP1 | -2.01384 | 4.51257 | 1.30E-18 | 1.77E-17 |
| ORC6 | 1.968024 | 1.488452 | 1.32E-18 | 1.80E-17 |
| HAO2 | -2.95896 | 5.360454 | 1.33E-18 | 1.82E-17 |
| FBLN7 | 2.851075 | 3.962949 | 1.37E-18 | 1.86E-17 |
| TTC38 | -1.3213 | 6.420416 | 1.48E-18 | 2.02E-17 |
| CD300LF | 3.125334 | 2.326647 | 1.50E-18 | 2.04E-17 |
| RXRG | -2.80035 | -3.13131 | 1.52E-18 | 2.07E-17 |
| DOC2A | 4.753073 | 4.836441 | 1.53E-18 | 2.08E-17 |
| MGAT4C | -2.96383 | -2.67483 | 1.54E-18 | 2.09E-17 |
| TM4SF19 | 4.009858 | -0.13873 | 1.57E-18 | 2.13E-17 |
| LRRC45 | 1.322177 | 5.00953 | 1.58E-18 | 2.14E-17 |
| WDR90 | 1.429005 | 5.695267 | 1.60E-18 | 2.16E-17 |
| FCGR3A | 2.481674 | 5.338801 | 1.60E-18 | 2.16E-17 |
| CCBE1 | -3.07275 | 1.190131 | 1.60E-18 | 2.17E-17 |
| GPRASP2 | -1.01868 | 3.894567 | 1.96E-18 | 2.64E-17 |
| HOXD3 | -2.04813 | 0.786645 | 2.04E-18 | 2.75E-17 |
| KIAA0408 | 3.542985 | -1.16145 | 2.05E-18 | 2.76E-17 |
| CCDC137 | 1.071484 | 4.917697 | 2.06E-18 | 2.78E-17 |
| SORCS2 | -2.86125 | 3.336555 | 2.11E-18 | 2.83E-17 |
| MUC13 | -2.59109 | 3.48383 | 2.11E-18 | 2.83E-17 |
| AC009336.2 | -2.18938 | -1.48518 | 2.13E-18 | 2.85E-17 |
| IFFO1 | 1.437922 | 4.737677 | 2.21E-18 | 2.96E-17 |
| GABRB2 | -2.93656 | -0.3889 | 2.24E-18 | 3.00E-17 |
| MYCN | -2.70012 | 0.166818 | 2.25E-18 | 3.01E-17 |
| KCNJ4 | 3.348731 | 1.687515 | 2.26E-18 | 3.03E-17 |
| PYGL | 1.566975 | 5.52131 | 2.28E-18 | 3.05E-17 |
| LY86 | 2.747469 | 3.606807 | 2.33E-18 | 3.11E-17 |
| ARAP3 | -1.68017 | 3.199315 | 2.35E-18 | 3.13E-17 |
| PCDH1 | -2.23523 | 5.098271 | 2.37E-18 | 3.16E-17 |
| SETBP1 | -1.19901 | 4.623389 | 2.53E-18 | 3.37E-17 |
| P3H4 | 1.244533 | 5.742332 | 2.64E-18 | 3.52E-17 |
| FCER1G | 2.501646 | 5.17677 | 2.67E-18 | 3.55E-17 |
| IFI27 | 2.799377 | 8.146897 | 2.72E-18 | 3.62E-17 |
| RNASE2 | 2.846053 | -0.18883 | 2.77E-18 | 3.67E-17 |
| NME1-NME2 | 1.803526 | -0.06633 | 3.03E-18 | 4.02E-17 |
| SLC7A13 | -4.17216 | 1.736282 | 3.10E-18 | 4.11E-17 |
| LRRFIP2 | 1.160276 | 6.373868 | 3.24E-18 | 4.28E-17 |
| ISG20 | 1.820523 | 3.369586 | 3.90E-18 | 5.15E-17 |
| BEX5 | -1.58438 | 2.160406 | 4.04E-18 | 5.32E-17 |
| PYCARD | 2.21469 | 4.258194 | 4.15E-18 | 5.46E-17 |
| ITGA3 | 1.836742 | 9.774992 | 4.21E-18 | 5.53E-17 |
| COL14A1 | -2.20547 | 4.53608 | 4.43E-18 | 5.82E-17 |
| ERMP1 | -1.3877 | 6.309928 | 4.73E-18 | 6.20E-17 |
| EFNA3 | 1.875954 | 1.374737 | 4.95E-18 | 6.49E-17 |
| C2orf40 | -2.62287 | 2.320562 | 5.01E-18 | 6.55E-17 |
| DNASE2B | 4.900728 | 0.108872 | 5.18E-18 | 6.77E-17 |
| PXDNL | -2.85217 | 0.731284 | 5.31E-18 | 6.94E-17 |
| TNFAIP2 | 2.000793 | 7.734433 | 5.50E-18 | 7.18E-17 |
| TYROBP | 2.671423 | 6.192108 | 5.52E-18 | 7.19E-17 |
| GMPR | -1.37282 | 4.032728 | 5.58E-18 | 7.27E-17 |
| RGS12 | 1.307895 | 6.576238 | 5.68E-18 | 7.39E-17 |
| TMEM30B | -2.752 | 3.734715 | 5.69E-18 | 7.40E-17 |
| MXD3 | 1.968384 | 2.684404 | 5.74E-18 | 7.46E-17 |
| SAP30 | 1.154266 | 2.452103 | 5.94E-18 | 7.71E-17 |
| EMP3 | 1.758936 | 5.864993 | 5.98E-18 | 7.76E-17 |
| SPOCK2 | -1.99104 | 6.507094 | 6.03E-18 | 7.82E-17 |
| ADCY2 | 3.536015 | 3.524745 | 6.17E-18 | 7.99E-17 |
| GCGR | -3.54855 | 2.453744 | 6.46E-18 | 8.36E-17 |
| HTATIP2 | 1.582766 | 5.827233 | 6.52E-18 | 8.43E-17 |
| FAM46B | -2.07344 | 2.29168 | 6.55E-18 | 8.46E-17 |
| PGAP1 | -1.19733 | 3.312236 | 6.72E-18 | 8.67E-17 |
| DUSP10 | 1.81367 | 3.411372 | 7.39E-18 | 9.52E-17 |
| NCAPH | 2.255433 | 1.194837 | 7.46E-18 | 9.61E-17 |
| CCM2 | 1.031121 | 5.859908 | 7.62E-18 | 9.81E-17 |
| MESP2 | 3.107581 | 0.850482 | 7.89E-18 | 1.02E-16 |
| HGFAC | 3.850487 | 3.098992 | 7.92E-18 | 1.02E-16 |
| PDP2 | -1.01384 | 4.194824 | 7.99E-18 | 1.03E-16 |
| SOWAHA | -1.91438 | 2.420616 | 8.54E-18 | 1.09E-16 |
| NOCT | -1.39799 | 2.068684 | 8.62E-18 | 1.10E-16 |
| MITD1 | 1.03158 | 4.134394 | 8.66E-18 | 1.11E-16 |
| LPGAT1 | 1.106463 | 6.506474 | 8.71E-18 | 1.11E-16 |
| PPP1R3G | 2.335215 | 3.375947 | 8.78E-18 | 1.12E-16 |
| STAG3 | 1.914373 | 3.059949 | 8.95E-18 | 1.14E-16 |
| CLEC14A | -1.48962 | 3.593776 | 8.96E-18 | 1.14E-16 |
| FUT8 | 1.376059 | 5.488057 | 9.02E-18 | 1.15E-16 |
| PDE8B | -1.12357 | 2.990663 | 9.07E-18 | 1.16E-16 |
| MMP14 | 1.830046 | 8.386472 | 9.44E-18 | 1.20E-16 |
| CTGF | -1.7312 | 7.122064 | 9.61E-18 | 1.22E-16 |
| SYNPO2 | -2.0289 | 4.335634 | 9.67E-18 | 1.23E-16 |
| CD300A | 2.302288 | 3.431872 | 9.75E-18 | 1.24E-16 |
| GDAP1L1 | 3.899798 | 0.025252 | 9.77E-18 | 1.24E-16 |
| NPAS2 | 1.388662 | 6.084877 | 1.02E-17 | 1.29E-16 |
| CPA1 | -3.205 | -2.54516 | 1.02E-17 | 1.30E-16 |
| ACACB | -1.09982 | 5.70242 | 1.03E-17 | 1.30E-16 |
| HSD3B7 | 1.714923 | 5.993862 | 1.04E-17 | 1.32E-16 |
| KIAA1462 | -1.5288 | 4.3634 | 1.07E-17 | 1.35E-16 |
| RBPMS | 1.060987 | 8.203683 | 1.07E-17 | 1.35E-16 |
| TMEM159 | 1.060767 | 5.900497 | 1.08E-17 | 1.36E-16 |
| VSTM2L | 5.157839 | 5.130168 | 1.09E-17 | 1.37E-16 |
| INO80E | 1.050126 | 5.969717 | 1.10E-17 | 1.38E-16 |
| FOXD2 | -1.38064 | 0.551696 | 1.10E-17 | 1.39E-16 |
| GALNS | 1.131826 | 5.696478 | 1.14E-17 | 1.43E-16 |
| ACRBP | 2.134294 | 3.0309 | 1.15E-17 | 1.45E-16 |
| CEL | -2.8232 | 1.692617 | 1.15E-17 | 1.45E-16 |
| HSPB1 | 1.615484 | 10.11136 | 1.18E-17 | 1.47E-16 |
| CDK5RAP3 | 1.548489 | 7.510027 | 1.18E-17 | 1.48E-16 |
| TAP1 | 1.239797 | 6.607055 | 1.20E-17 | 1.50E-16 |
| TRABD2A | 3.131483 | 3.2752 | 1.24E-17 | 1.56E-16 |
| CSF3R | 2.478775 | 4.295748 | 1.28E-17 | 1.60E-16 |
| SULT4A1 | 3.833207 | 1.456369 | 1.28E-17 | 1.60E-16 |
| LMOD1 | -2.2525 | 3.328857 | 1.34E-17 | 1.67E-16 |
| EML6 | -1.61333 | 1.748114 | 1.35E-17 | 1.68E-16 |
| PBX1 | -1.25583 | 5.870369 | 1.35E-17 | 1.68E-16 |
| PDK4 | -2.10489 | 6.656831 | 1.37E-17 | 1.70E-16 |
| HIBCH | -1.13652 | 5.796639 | 1.38E-17 | 1.71E-16 |
| APBA2 | 2.685988 | 3.282176 | 1.46E-17 | 1.81E-16 |
| PUS1 | 1.029315 | 4.631691 | 1.46E-17 | 1.81E-16 |
| ANKRD18B | 3.893466 | 0.264395 | 1.47E-17 | 1.82E-16 |
| MRVI1 | -1.95854 | 3.389264 | 1.49E-17 | 1.85E-16 |
| ANXA4 | 1.218642 | 8.702594 | 1.50E-17 | 1.86E-16 |
| HSPB2 | 2.177458 | 3.216981 | 1.51E-17 | 1.87E-16 |
| OSBPL7 | 1.116465 | 4.607277 | 1.55E-17 | 1.91E-16 |
| LTB4R | 1.856428 | 3.261565 | 1.56E-17 | 1.93E-16 |
| OR2C1 | -1.95086 | -3.60526 | 1.61E-17 | 1.98E-16 |
| CDC25C | 3.150005 | -0.3123 | 1.66E-17 | 2.04E-16 |
| PGM5 | -1.59094 | 4.11624 | 1.68E-17 | 2.07E-16 |
| TFPI2 | 4.03476 | 8.540119 | 1.70E-17 | 2.09E-16 |
| SH3BP5 | -1.80697 | 2.879341 | 1.70E-17 | 2.09E-16 |
| LHFPL2 | 1.721622 | 4.784491 | 1.75E-17 | 2.14E-16 |
| LHX6 | -1.75113 | 0.412471 | 1.82E-17 | 2.23E-16 |
| LRRC23 | 1.183648 | 4.873612 | 1.84E-17 | 2.25E-16 |
| CLU | 2.133899 | 12.12432 | 1.96E-17 | 2.40E-16 |
| RIOX2 | -1.21376 | 3.570725 | 1.99E-17 | 2.43E-16 |
| GABRP | -2.6199 | 2.239491 | 2.08E-17 | 2.53E-16 |
| C5orf47 | -1.92705 | -2.87927 | 2.09E-17 | 2.55E-16 |
| KRT86 | 3.023232 | 2.024526 | 2.10E-17 | 2.56E-16 |
| THBS3 | 1.324068 | 5.395687 | 2.18E-17 | 2.65E-16 |
| DIO1 | -3.01287 | 4.273731 | 2.18E-17 | 2.65E-16 |
| DOK1 | 1.355848 | 3.980801 | 2.23E-17 | 2.70E-16 |
| PTCHD4 | 2.963554 | 3.60728 | 2.25E-17 | 2.72E-16 |
| DLGAP5 | 2.913477 | 0.945572 | 2.33E-17 | 2.83E-16 |
| AC114783.1 | -3.0964 | -3.98785 | 2.36E-17 | 2.86E-16 |
| ALPL | -2.06335 | 5.075437 | 2.38E-17 | 2.87E-16 |
| PARVG | 2.328186 | 4.015793 | 2.51E-17 | 3.03E-16 |
| SLC9A2 | -2.39171 | 2.598164 | 2.51E-17 | 3.03E-16 |
| TRPM2 | 2.481011 | 3.767857 | 2.55E-17 | 3.07E-16 |
| ANKRD63 | -3.1862 | -3.80537 | 2.61E-17 | 3.15E-16 |
| GPR146 | -1.30335 | 0.377073 | 2.65E-17 | 3.18E-16 |
| FHL1 | 3.485103 | 9.736957 | 2.73E-17 | 3.28E-16 |
| IDH2 | -1.2938 | 7.596646 | 2.74E-17 | 3.30E-16 |
| NEK2 | 3.006351 | 0.998388 | 2.77E-17 | 3.33E-16 |
| FLOT2 | 1.021467 | 8.269791 | 2.78E-17 | 3.34E-16 |
| C19orf81 | 3.426695 | 0.189817 | 2.81E-17 | 3.37E-16 |
| TMEM74B | 3.445329 | 2.57104 | 2.83E-17 | 3.39E-16 |
| C2orf72 | -2.33053 | 1.158929 | 2.88E-17 | 3.45E-16 |
| WIPI1 | 1.124048 | 5.835013 | 3.01E-17 | 3.60E-16 |
| LIMCH1 | -1.18514 | 5.74218 | 3.16E-17 | 3.78E-16 |
| CAV2 | 1.670121 | 6.551863 | 3.22E-17 | 3.85E-16 |
| ECM2 | -1.63787 | 3.023054 | 3.28E-17 | 3.92E-16 |
| CASTOR1 | -1.15434 | 0.998244 | 3.38E-17 | 4.03E-16 |
| APOBEC3D | 1.679431 | 2.478424 | 3.43E-17 | 4.09E-16 |
| BMP5 | -3.52278 | -0.90827 | 3.45E-17 | 4.11E-16 |
| FOXM1 | 2.107895 | 2.674623 | 3.46E-17 | 4.12E-16 |
| FAM234A | 1.21745 | 7.235187 | 3.47E-17 | 4.13E-16 |
| LAMA2 | -1.54804 | 3.186659 | 3.53E-17 | 4.20E-16 |
| DNAJB13 | 3.246106 | 1.944444 | 3.55E-17 | 4.22E-16 |
| TRMT1 | 1.108132 | 5.327563 | 3.58E-17 | 4.25E-16 |
| S100A10 | 1.138767 | 8.195433 | 3.59E-17 | 4.25E-16 |
| SNAI2 | -1.87425 | 1.629535 | 3.59E-17 | 4.26E-16 |
| PSMB10 | 1.243903 | 5.222815 | 3.65E-17 | 4.32E-16 |
| RIMS2 | 3.406024 | 3.029288 | 3.65E-17 | 4.32E-16 |
| PLPP4 | -2.02401 | 1.41264 | 3.67E-17 | 4.35E-16 |
| CYP26B1 | -1.65435 | 3.126924 | 3.73E-17 | 4.41E-16 |
| SEC61G | 1.07971 | 5.518742 | 3.82E-17 | 4.51E-16 |
| ABCC3 | 2.435052 | 7.627257 | 3.83E-17 | 4.53E-16 |
| EGR3 | -2.18935 | 2.830666 | 3.86E-17 | 4.56E-16 |
| CDHR1 | 4.865733 | 5.323434 | 3.88E-17 | 4.58E-16 |
| SIRPA | 1.268463 | 7.49863 | 3.93E-17 | 4.63E-16 |
| FRMD3 | -1.40397 | 4.309616 | 4.12E-17 | 4.85E-16 |
| RRM2 | 2.828772 | 3.162685 | 4.23E-17 | 4.98E-16 |
| BMPR1B | -3.1187 | 2.551418 | 4.27E-17 | 5.02E-16 |
| KRT18 | 1.703576 | 9.536061 | 4.28E-17 | 5.02E-16 |
| ANKS3 | 1.195702 | 5.65542 | 4.30E-17 | 5.05E-16 |
| SLC16A3 | 2.305136 | 7.22076 | 4.32E-17 | 5.06E-16 |
| MYO1F | 1.969081 | 4.931808 | 4.51E-17 | 5.29E-16 |
| NEDD4L | -1.08898 | 6.706906 | 4.58E-17 | 5.36E-16 |
| CTXN3 | -4.23625 | 2.367917 | 4.77E-17 | 5.58E-16 |
| CYP1A1 | -3.74288 | -1.20902 | 4.78E-17 | 5.59E-16 |
| RBP7 | -1.53151 | 2.505907 | 4.81E-17 | 5.62E-16 |
| VCAN | 3.076599 | 8.963079 | 4.83E-17 | 5.64E-16 |
| CLDN1 | 2.416758 | 7.889847 | 4.98E-17 | 5.80E-16 |
| CD93 | -1.48651 | 5.379796 | 5.01E-17 | 5.84E-16 |
| GRIK2 | -2.34222 | -0.53555 | 5.01E-17 | 5.84E-16 |
| PLEKHA4 | 1.539004 | 6.085392 | 5.38E-17 | 6.26E-16 |
| THSD7A | -1.7582 | 4.77781 | 5.42E-17 | 6.29E-16 |
| CDC42EP2 | -1.25851 | 3.019554 | 5.52E-17 | 6.40E-16 |
| HYAL4 | -2.90901 | -2.88899 | 5.59E-17 | 6.48E-16 |
| BTBD16 | 3.08706 | 1.190566 | 5.61E-17 | 6.50E-16 |
| CACNA2D2 | -1.59766 | 2.109837 | 5.62E-17 | 6.51E-16 |
| FOXC2 | -1.92044 | 0.937297 | 5.64E-17 | 6.53E-16 |
| C4orf48 | 2.635258 | 3.145693 | 5.79E-17 | 6.70E-16 |
| LAIR1 | 2.262044 | 4.830794 | 5.84E-17 | 6.75E-16 |
| FOXE3 | -2.07094 | -3.74622 | 5.86E-17 | 6.77E-16 |
| FFAR4 | 3.28504 | 1.276075 | 6.11E-17 | 7.04E-16 |
| EBF1 | -1.9136 | 2.100733 | 6.24E-17 | 7.18E-16 |
| CDR2 | 1.116332 | 6.41754 | 6.32E-17 | 7.27E-16 |
| SLC13A2 | -2.95546 | 4.535994 | 6.44E-17 | 7.40E-16 |
| PFKFB3 | -1.67838 | 7.177042 | 6.45E-17 | 7.42E-16 |
| MESP1 | 2.200276 | 2.146158 | 6.51E-17 | 7.48E-16 |
| OLFML3 | -1.50688 | 3.703951 | 6.69E-17 | 7.68E-16 |
| TRIM2 | -1.2586 | 6.39892 | 6.86E-17 | 7.87E-16 |
| CARHSP1 | 1.166145 | 7.029934 | 6.96E-17 | 7.97E-16 |
| EMX1 | -1.62857 | 5.236411 | 7.07E-17 | 8.09E-16 |
| CDKN3 | 2.160968 | 0.857781 | 7.17E-17 | 8.20E-16 |
| HACE1 | -1.10834 | 3.178723 | 7.19E-17 | 8.22E-16 |
| CA9 | 5.908323 | 3.976957 | 7.22E-17 | 8.25E-16 |
| NRK | -3.09829 | 3.140997 | 7.47E-17 | 8.52E-16 |
| AKAP2 | -2.04482 | -0.44861 | 7.58E-17 | 8.65E-16 |
| 4-Sep | -1.4307 | 2.880498 | 7.64E-17 | 8.71E-16 |
| PAQR6 | 2.365991 | 2.45614 | 7.82E-17 | 8.91E-16 |
| TPD52 | -1.17476 | 5.203414 | 7.96E-17 | 9.06E-16 |
| NBL1 | 2.552673 | 7.364813 | 8.10E-17 | 9.21E-16 |
| PATL2 | 1.986323 | 1.066496 | 8.13E-17 | 9.24E-16 |
| ADAMTS14 | 3.590222 | 1.991764 | 8.38E-17 | 9.52E-16 |
| CNPY3 | 1.176203 | 6.554428 | 8.46E-17 | 9.59E-16 |
| CLCF1 | 1.486217 | 5.135932 | 8.51E-17 | 9.64E-16 |
| HAGHL | 2.538153 | 4.480307 | 8.51E-17 | 9.64E-16 |
| TAF1D | 1.089679 | 5.511747 | 9.03E-17 | 1.02E-15 |
| PKD2L1 | 4.912442 | 2.018046 | 9.50E-17 | 1.07E-15 |
| ARSF | -2.73259 | 1.098166 | 1.00E-16 | 1.13E-15 |
| MTHFD1L | 1.120936 | 4.827817 | 1.01E-16 | 1.14E-15 |
| DRC7 | 2.630712 | 3.529028 | 1.06E-16 | 1.19E-15 |
| ARRB2 | 1.281061 | 5.677462 | 1.07E-16 | 1.21E-15 |
| CTNNA3 | -2.17383 | -1.55798 | 1.08E-16 | 1.21E-15 |
| RGS20 | 3.408805 | 0.281951 | 1.08E-16 | 1.22E-15 |
| LPXN | 1.219289 | 4.489614 | 1.11E-16 | 1.25E-15 |
| CACNA1C | -1.7792 | 1.889617 | 1.11E-16 | 1.25E-15 |
| CUBN | -2.41925 | 7.87212 | 1.11E-16 | 1.25E-15 |
| MYLK | -1.61116 | 6.576422 | 1.14E-16 | 1.28E-15 |
| MYBL1 | 1.867614 | 2.786593 | 1.18E-16 | 1.32E-15 |
| ATP6V1A | -1.03111 | 7.359936 | 1.20E-16 | 1.34E-15 |
| ITGAD | 5.150159 | 2.302952 | 1.21E-16 | 1.36E-15 |
| KHK | -2.18055 | 6.11093 | 1.25E-16 | 1.40E-15 |
| IL32 | 2.337154 | 8.449873 | 1.27E-16 | 1.41E-15 |
| SOWAHC | -1.23157 | 4.643541 | 1.31E-16 | 1.46E-15 |
| SCG5 | 4.02324 | 3.424087 | 1.34E-16 | 1.49E-15 |
| SEC14L5 | -1.33038 | -0.25052 | 1.34E-16 | 1.49E-15 |
| DOK3 | 1.788742 | 3.227041 | 1.35E-16 | 1.50E-15 |
| IL17B | -1.93833 | -2.53686 | 1.38E-16 | 1.54E-15 |
| SNRPF | 1.125131 | 5.119672 | 1.39E-16 | 1.54E-15 |
| ABCA3 | 1.342837 | 6.868857 | 1.41E-16 | 1.56E-15 |
| EHF | -2.72581 | 3.495973 | 1.41E-16 | 1.56E-15 |
| KCNE1 | -2.0716 | -0.79786 | 1.44E-16 | 1.60E-15 |
| ALG3 | 1.010369 | 5.686605 | 1.46E-16 | 1.61E-15 |
| CGA | -4.05042 | -2.39857 | 1.52E-16 | 1.68E-15 |
| FAM189A1 | 2.398562 | 5.183499 | 1.56E-16 | 1.73E-15 |
| ADI1 | -1.01935 | 7.054195 | 1.59E-16 | 1.76E-15 |
| GSTA1 | -3.27334 | 5.52135 | 1.64E-16 | 1.81E-15 |
| ARID5B | -1.04906 | 6.041326 | 1.64E-16 | 1.81E-15 |
| SLC23A1 | -1.90136 | 4.329053 | 1.65E-16 | 1.82E-15 |
| PDGFRB | -1.72889 | 5.443221 | 1.68E-16 | 1.85E-15 |
| RAD54L | 1.902226 | 0.799663 | 1.70E-16 | 1.87E-15 |
| RAD9A | 1.253743 | 4.6427 | 1.74E-16 | 1.91E-15 |
| ONECUT2 | 3.610291 | 2.705595 | 1.81E-16 | 1.98E-15 |
| LAPTM5 | 2.22002 | 7.676036 | 1.81E-16 | 1.98E-15 |
| SPI1 | 2.122502 | 4.899539 | 1.83E-16 | 2.00E-15 |
| CCDC152 | -1.38859 | 0.687473 | 1.85E-16 | 2.03E-15 |
| SLC25A34 | -1.71856 | 2.375789 | 1.89E-16 | 2.07E-15 |
| CDH3 | -2.81835 | 3.300827 | 1.92E-16 | 2.10E-15 |
| KRT81 | 3.21929 | -0.69697 | 1.93E-16 | 2.11E-15 |
| METTL27 | 2.679509 | 5.306041 | 1.93E-16 | 2.11E-15 |
| TAX1BP3 | 1.164609 | 5.747628 | 1.94E-16 | 2.12E-15 |
| RNASEH2A | 1.088825 | 3.948532 | 1.94E-16 | 2.12E-15 |
| CCNB2 | 1.938348 | 1.999249 | 2.00E-16 | 2.17E-15 |
| KCTD19 | 2.981538 | 0.193974 | 2.01E-16 | 2.18E-15 |
| ARHGAP24 | -1.05247 | 6.552535 | 2.04E-16 | 2.22E-15 |
| PCSK9 | -2.60947 | -2.63118 | 2.05E-16 | 2.23E-15 |
| ANLN | 2.545933 | 2.751981 | 2.13E-16 | 2.32E-15 |
| SYNE3 | -1.5653 | 1.681879 | 2.16E-16 | 2.34E-15 |
| GATS | 1.504711 | 5.211001 | 2.28E-16 | 2.47E-15 |
| APLNR | -2.36962 | 2.992584 | 2.32E-16 | 2.51E-15 |
| SLC23A3 | -2.14913 | 3.540866 | 2.35E-16 | 2.54E-15 |
| FCGR1A | 2.654615 | 2.092767 | 2.45E-16 | 2.64E-15 |
| CKM | -2.3644 | -1.26858 | 2.52E-16 | 2.72E-15 |
| L2HGDH | -1.07032 | 4.155163 | 2.62E-16 | 2.82E-15 |
| C9orf47 | -1.99925 | -2.07681 | 2.63E-16 | 2.84E-15 |
| RAD51 | 1.804467 | 1.026328 | 2.71E-16 | 2.91E-15 |
| WSCD2 | -2.86046 | 0.071415 | 2.79E-16 | 3.00E-15 |
| HACD3 | -1.03748 | 6.66171 | 2.93E-16 | 3.15E-15 |
| CD82 | -1.33511 | 5.30382 | 2.97E-16 | 3.19E-15 |
| MT1F | -2.09351 | 5.068573 | 2.97E-16 | 3.19E-15 |
| PLA2G16 | 1.602017 | 7.634221 | 3.00E-16 | 3.21E-15 |
| MICALL2 | 2.033593 | 5.517892 | 3.01E-16 | 3.23E-15 |
| TMIGD1 | -2.31644 | -0.99396 | 3.09E-16 | 3.30E-15 |
| F2R | -1.40366 | 4.572645 | 3.11E-16 | 3.32E-15 |
| NXPH2 | -4.11849 | -1.37331 | 3.12E-16 | 3.34E-15 |
| ACY1 | -1.31265 | 4.329571 | 3.14E-16 | 3.36E-15 |
| MC1R | 2.021847 | 3.218006 | 3.22E-16 | 3.43E-15 |
| CMTM7 | 1.629559 | 5.258847 | 3.24E-16 | 3.45E-15 |
| OVCH2 | -3.44053 | 0.707857 | 3.27E-16 | 3.49E-15 |
| AC025594.3 | 2.73441 | 5.362564 | 3.30E-16 | 3.52E-15 |
| SIGLEC9 | 2.282003 | 1.981139 | 3.41E-16 | 3.63E-15 |
| FAS | 1.413084 | 5.02288 | 3.44E-16 | 3.65E-15 |
| CNN1 | -2.40416 | 3.601428 | 3.45E-16 | 3.67E-15 |
| VDR | -1.66148 | 4.858248 | 3.48E-16 | 3.69E-15 |
| ALKBH6 | 1.579294 | 1.901729 | 3.57E-16 | 3.79E-15 |
| FLT1 | -1.71055 | 5.336744 | 3.58E-16 | 3.79E-15 |
| HEPH | -1.78322 | 2.683704 | 3.69E-16 | 3.91E-15 |
| RAD51AP1 | 1.521099 | 1.53189 | 3.69E-16 | 3.91E-15 |
| PPP1R35 | 1.571029 | 4.615027 | 3.88E-16 | 4.10E-15 |
| EHBP1L1 | 1.187301 | 5.888846 | 3.93E-16 | 4.14E-15 |
| IDUA | 1.580246 | 4.833381 | 4.13E-16 | 4.35E-15 |
| AP1G2 | 1.1908 | 5.410801 | 4.16E-16 | 4.39E-15 |
| DDX11 | 1.510742 | 4.734509 | 4.23E-16 | 4.46E-15 |
| NBEAL1 | -1.23325 | 3.629764 | 4.25E-16 | 4.48E-15 |
| TKT | 1.572452 | 8.562079 | 4.26E-16 | 4.48E-15 |
| SLC17A2 | 3.2191 | 2.218295 | 4.27E-16 | 4.49E-15 |
| ZNF280B | 1.509563 | 1.539979 | 4.29E-16 | 4.50E-15 |
| ZDHHC2 | -1.04334 | 5.489684 | 4.40E-16 | 4.62E-15 |
| STPG2 | -1.47692 | -1.18853 | 4.61E-16 | 4.83E-15 |
| SIM1 | -1.77641 | 4.477764 | 4.64E-16 | 4.86E-15 |
| RBM33 | 1.043834 | 5.99525 | 4.65E-16 | 4.87E-15 |
| KCNH8 | 3.516635 | 1.200149 | 5.23E-16 | 5.46E-15 |
| PTGS1 | -1.63999 | 4.476505 | 5.29E-16 | 5.52E-15 |
| SCNN1G | -4.41179 | 4.858119 | 5.66E-16 | 5.89E-15 |
| EXOC3L1 | -1.17436 | 1.934549 | 5.80E-16 | 6.03E-15 |
| SLC16A6 | 2.681133 | 2.992899 | 5.82E-16 | 6.05E-15 |
| MOGAT2 | -3.56001 | -0.82251 | 5.96E-16 | 6.19E-15 |
| LGALS1 | 1.779708 | 7.436393 | 5.98E-16 | 6.20E-15 |
| TMEM98 | 1.626383 | 6.924342 | 5.99E-16 | 6.21E-15 |
| OCIAD2 | 1.430895 | 7.857243 | 6.11E-16 | 6.33E-15 |
| ASIP | 3.341341 | 0.344393 | 6.35E-16 | 6.57E-15 |
| CCL16 | -1.89958 | -3.26256 | 6.38E-16 | 6.60E-15 |
| TMEM86A | -1.31086 | 4.196359 | 6.78E-16 | 7.00E-15 |
| TBL1Y | -3.85098 | -1.835 | 6.80E-16 | 7.02E-15 |
| SYN2 | -2.04694 | 0.598918 | 6.83E-16 | 7.04E-15 |
| TUBB4A | 3.211701 | 5.602717 | 6.95E-16 | 7.15E-15 |
| PDLIM1 | 1.193719 | 6.923718 | 7.00E-16 | 7.20E-15 |
| PSORS1C2 | 5.015256 | 0.624843 | 7.16E-16 | 7.35E-15 |
| CDC7 | 1.199759 | 2.328304 | 7.27E-16 | 7.46E-15 |
| DDC | -2.19524 | 6.459374 | 7.67E-16 | 7.87E-15 |
| MRM1 | 1.045223 | 3.783879 | 7.69E-16 | 7.88E-15 |
| MDK | 2.457629 | 6.443915 | 7.79E-16 | 7.98E-15 |
| ABHD17C | -1.19645 | 3.98922 | 8.37E-16 | 8.56E-15 |
| SEMA6A | 1.567741 | 6.5972 | 8.38E-16 | 8.56E-15 |
| VWA5B1 | -3.23828 | 0.038716 | 8.40E-16 | 8.58E-15 |
| AMACR | 2.030976 | 7.146239 | 8.51E-16 | 8.68E-15 |
| RNFT2 | 1.500253 | 2.888869 | 8.52E-16 | 8.69E-15 |
| BCL2A1 | 2.927632 | 2.247403 | 8.84E-16 | 9.00E-15 |
| CENPW | 1.501388 | 1.37175 | 9.09E-16 | 9.25E-15 |
| RNF213 | 1.203515 | 8.746015 | 9.24E-16 | 9.39E-15 |
| SYT6 | -2.07043 | 0.052151 | 9.27E-16 | 9.41E-15 |
| TEF | -1.0663 | 5.524693 | 9.28E-16 | 9.42E-15 |
| RHEBL1 | 1.544211 | -0.22003 | 9.43E-16 | 9.56E-15 |
| CLDN8 | -4.54795 | 3.833822 | 9.47E-16 | 9.61E-15 |
| SIGLEC10 | 2.029152 | 2.820511 | 9.62E-16 | 9.75E-15 |
| APOLD1 | -1.68168 | 4.293438 | 9.81E-16 | 9.94E-15 |
| SLC35F6 | 1.155407 | 7.341442 | 1.00E-15 | 1.01E-14 |
| IGFN1 | 7.394149 | 5.433958 | 1.00E-15 | 1.02E-14 |
| CRABP1 | -3.9339 | -0.28733 | 1.00E-15 | 1.02E-14 |
| CCDC3 | -1.71322 | 3.34751 | 1.01E-15 | 1.02E-14 |
| AXDND1 | -1.63343 | -0.99586 | 1.03E-15 | 1.04E-14 |
| EEF1A2 | 4.800036 | 6.110784 | 1.04E-15 | 1.05E-14 |
| BCO1 | 1.654874 | 3.484563 | 1.05E-15 | 1.06E-14 |
| C3orf62 | 1.04026 | 3.879216 | 1.12E-15 | 1.13E-14 |
| KIF20A | 2.673812 | 2.073795 | 1.14E-15 | 1.15E-14 |
| SORL1 | -1.3136 | 6.849428 | 1.15E-15 | 1.16E-14 |
| BEAN1 | 2.783009 | 2.285123 | 1.15E-15 | 1.16E-14 |
| SLC1A3 | 2.810034 | 4.020022 | 1.16E-15 | 1.16E-14 |
| ATP6V0D2 | -3.15656 | 4.534829 | 1.17E-15 | 1.18E-14 |
| CAB39L | -1.05477 | 4.17527 | 1.17E-15 | 1.18E-14 |
| ADCY1 | -2.37045 | 1.92834 | 1.18E-15 | 1.19E-14 |
| SLC11A1 | 2.198942 | 3.653069 | 1.19E-15 | 1.19E-14 |
| TNFRSF10C | 1.882146 | 3.952033 | 1.21E-15 | 1.21E-14 |
| DTX1 | -1.56404 | 3.493469 | 1.21E-15 | 1.21E-14 |
| CFB | 1.89715 | 5.016578 | 1.22E-15 | 1.22E-14 |
| TMEM38B | -1.16559 | 4.675988 | 1.23E-15 | 1.23E-14 |
| CMTM5 | -2.15313 | -3.10984 | 1.27E-15 | 1.27E-14 |
| TAF10 | 1.04196 | 6.124758 | 1.37E-15 | 1.37E-14 |
| FAM57B | 3.298825 | -1.5715 | 1.40E-15 | 1.40E-14 |
| C5AR2 | -1.75164 | 1.36314 | 1.40E-15 | 1.40E-14 |
| RXRA | -1.15802 | 5.863563 | 1.42E-15 | 1.41E-14 |
| SLCO5A1 | 2.044946 | 0.330085 | 1.45E-15 | 1.44E-14 |
| HLA-B | 1.381905 | 10.97931 | 1.46E-15 | 1.45E-14 |
| ADRA2C | -1.93551 | 2.671964 | 1.46E-15 | 1.45E-14 |
| AGBL2 | 2.010364 | 2.546018 | 1.52E-15 | 1.50E-14 |
| PLCB2 | 1.846326 | 4.437659 | 1.52E-15 | 1.51E-14 |
| DDR2 | -1.80563 | 3.172153 | 1.55E-15 | 1.53E-14 |
| JAK3 | 1.871674 | 4.43273 | 1.58E-15 | 1.56E-14 |
| FAM50A | 1.096255 | 6.680916 | 1.64E-15 | 1.62E-14 |
| FAM26D | -2.06 | -3.68128 | 1.64E-15 | 1.62E-14 |
| FAM212B | -1.07016 | 2.709463 | 1.66E-15 | 1.64E-14 |
| LRRC61 | 1.213384 | 5.49253 | 1.70E-15 | 1.68E-14 |
| NABP1 | 1.627392 | 4.916596 | 1.76E-15 | 1.73E-14 |
| IMPG2 | -1.43458 | -1.02579 | 1.79E-15 | 1.76E-14 |
| FKBP11 | 1.295893 | 4.234079 | 1.80E-15 | 1.77E-14 |
| MYO1A | 2.994584 | -0.56466 | 1.80E-15 | 1.77E-14 |
| OPRD1 | 1.922487 | -0.18282 | 1.87E-15 | 1.83E-14 |
| KNL1 | -1.53919 | 1.409216 | 1.87E-15 | 1.83E-14 |
| RASD1 | -2.87448 | 5.415828 | 1.88E-15 | 1.84E-14 |
| CDSN | 3.507919 | -2.52512 | 1.94E-15 | 1.90E-14 |
| WEE1 | -1.15481 | 4.1612 | 1.96E-15 | 1.91E-14 |
| MKI67 | 2.560507 | 3.596724 | 1.99E-15 | 1.94E-14 |
| ETV7 | 1.896147 | 2.242864 | 2.06E-15 | 2.01E-14 |
| POLR2J | 1.274824 | 5.773945 | 2.07E-15 | 2.02E-14 |
| IL1RL1 | -2.4361 | 3.336673 | 2.08E-15 | 2.03E-14 |
| PTRH2 | 1.073809 | 4.131091 | 2.22E-15 | 2.16E-14 |
| CD24 | 1.185664 | 11.11675 | 2.25E-15 | 2.18E-14 |
| SH3BGRL3 | 1.150931 | 7.389812 | 2.27E-15 | 2.21E-14 |
| IRF5 | 1.319647 | 5.772602 | 2.28E-15 | 2.21E-14 |
| CDCA8 | 1.771672 | 1.72369 | 2.32E-15 | 2.25E-14 |
| CD200 | 1.953613 | 5.415015 | 2.35E-15 | 2.28E-14 |
| PPP2R2B | -2.34737 | 1.474233 | 2.36E-15 | 2.29E-14 |
| ADIPOQ | -4.41224 | -1.05407 | 2.38E-15 | 2.31E-14 |
| ATP1B1 | -1.26942 | 9.98377 | 2.40E-15 | 2.32E-14 |
| SGPP1 | -1.06815 | 5.161801 | 2.43E-15 | 2.34E-14 |
| ABCG2 | -1.34295 | 1.992738 | 2.43E-15 | 2.34E-14 |
| CDH6 | 2.436516 | 8.702565 | 2.44E-15 | 2.35E-14 |
| RGS19 | 1.301674 | 3.735664 | 2.44E-15 | 2.35E-14 |
| ITPA | 1.044104 | 6.039366 | 2.44E-15 | 2.35E-14 |
| PRR15 | -1.54002 | 3.457338 | 2.46E-15 | 2.37E-14 |
| CXorf58 | -1.17609 | -2.54396 | 2.47E-15 | 2.38E-14 |
| CDCA7 | 2.724078 | 1.758551 | 2.50E-15 | 2.40E-14 |
| RGS14 | 1.522005 | 5.946979 | 2.50E-15 | 2.40E-14 |
| SOWAHB | -1.30005 | 3.194771 | 2.51E-15 | 2.41E-14 |
| SYT14 | 3.1175 | 2.484722 | 2.52E-15 | 2.42E-14 |
| ZMYND10 | 2.41855 | 4.451435 | 2.55E-15 | 2.44E-14 |
| BRCA1 | 1.320154 | 3.003486 | 2.59E-15 | 2.48E-14 |
| CARD9 | 1.567848 | 2.293445 | 2.60E-15 | 2.49E-14 |
| CRLS1 | 1.327431 | 7.114535 | 2.63E-15 | 2.52E-14 |
| DLG2 | -1.33171 | 1.778649 | 2.64E-15 | 2.52E-14 |
| CCL11 | -2.65803 | -1.28061 | 2.71E-15 | 2.59E-14 |
| TES | 1.261483 | 7.080963 | 2.77E-15 | 2.64E-14 |
| MT1H | -3.64283 | 3.964514 | 2.81E-15 | 2.68E-14 |
| CYP51A1 | -1.36208 | 1.85502 | 2.87E-15 | 2.73E-14 |
| EAF2 | -1.29137 | 2.205638 | 2.97E-15 | 2.82E-14 |
| 9-Mar | 1.056946 | 5.791739 | 3.06E-15 | 2.91E-14 |
| CACNA1H | -2.00781 | 4.337275 | 3.09E-15 | 2.94E-14 |
| GGT5 | -1.71356 | 4.630174 | 3.12E-15 | 2.96E-14 |
| NNMT | 3.298399 | 8.454583 | 3.28E-15 | 3.11E-14 |
| IGDCC4 | 2.416378 | 3.343799 | 3.30E-15 | 3.13E-14 |
| ACHE | 2.760248 | 4.034365 | 3.31E-15 | 3.13E-14 |
| HOMER3 | 1.214347 | 6.019186 | 3.32E-15 | 3.14E-14 |
| HEG1 | -1.28736 | 5.745094 | 3.37E-15 | 3.19E-14 |
| RHOJ | -1.3852 | 2.502201 | 3.39E-15 | 3.20E-14 |
| MFSD10 | 1.081738 | 6.695722 | 3.54E-15 | 3.34E-14 |
| SCEL | 4.997164 | 5.020304 | 3.54E-15 | 3.34E-14 |
| CER1 | -2.47576 | -3.59615 | 3.60E-15 | 3.40E-14 |
| NCAPG | 2.3829 | 1.157117 | 3.61E-15 | 3.40E-14 |
| PLCE1 | -1.17508 | 4.186983 | 3.63E-15 | 3.42E-14 |
| CCDC189 | 1.478181 | 1.654399 | 3.63E-15 | 3.42E-14 |
| TBX2 | -1.99852 | 4.767885 | 3.68E-15 | 3.46E-14 |
| SMIM18 | -1.87406 | -3.56147 | 3.72E-15 | 3.50E-14 |
| ZNF804B | -3.9736 | -1.91031 | 3.76E-15 | 3.53E-14 |
| ALDOA | 1.168186 | 11.1125 | 3.77E-15 | 3.54E-14 |
| TTC25 | 1.422948 | 3.413016 | 3.86E-15 | 3.61E-14 |
| XKR3 | 5.016001 | -1.23138 | 3.99E-15 | 3.73E-14 |
| OXCT2 | 2.969974 | -0.23677 | 4.03E-15 | 3.77E-14 |
| GMNC | -2.89866 | 0.011157 | 4.11E-15 | 3.84E-14 |
| SPAG5 | -1.54598 | 3.893135 | 4.25E-15 | 3.97E-14 |
| CCDC57 | 1.685752 | 5.927384 | 4.45E-15 | 4.15E-14 |
| DISC1 | 1.177732 | 2.765209 | 4.53E-15 | 4.22E-14 |
| FBXL8 | 1.26683 | 2.997832 | 4.54E-15 | 4.23E-14 |
| RPL23 | 1.028251 | 9.453991 | 4.56E-15 | 4.24E-14 |
| BTN3A2 | 1.131844 | 5.925381 | 4.77E-15 | 4.44E-14 |
| TCEAL2 | -2.91939 | 1.348289 | 4.79E-15 | 4.46E-14 |
| RPL27 | 1.050624 | 8.980638 | 4.80E-15 | 4.46E-14 |
| MAST1 | 2.338893 | 0.458963 | 4.85E-15 | 4.50E-14 |
| FMO4 | -1.19682 | 4.44242 | 5.00E-15 | 4.63E-14 |
| BTK | 1.975561 | 2.964349 | 5.01E-15 | 4.64E-14 |
| KIAA1210 | -2.86287 | -2.28262 | 5.08E-15 | 4.70E-14 |
| SLC7A10 | -3.05376 | -2.76343 | 5.15E-15 | 4.77E-14 |
| SHANK3 | -1.09614 | 4.804529 | 5.21E-15 | 4.82E-14 |
| HOXD10 | -2.61755 | 2.66695 | 5.29E-15 | 4.89E-14 |
| C11orf96 | -1.9098 | 3.958189 | 5.33E-15 | 4.92E-14 |
| SLC25A47 | -2.20651 | -1.16445 | 5.36E-15 | 4.95E-14 |
| GSG2 | 1.945683 | -0.88665 | 5.39E-15 | 4.97E-14 |
| MTNR1A | -2.49044 | -1.42274 | 5.50E-15 | 5.07E-14 |
| UNC93B1 | 1.215919 | 5.763239 | 5.57E-15 | 5.12E-14 |
| EDNRA | -1.71176 | 1.93577 | 5.59E-15 | 5.14E-14 |
| TOR4A | 1.125044 | 5.464696 | 5.74E-15 | 5.27E-14 |
| CENPM | 1.989085 | 1.487864 | 5.83E-15 | 5.36E-14 |
| FAM167B | -1.26097 | 2.969038 | 5.85E-15 | 5.37E-14 |
| A1CF | -2.45068 | 3.425401 | 5.89E-15 | 5.41E-14 |
| ELFN2 | 2.343144 | 4.615515 | 6.00E-15 | 5.50E-14 |
| OVGP1 | 1.870984 | 3.276217 | 6.01E-15 | 5.51E-14 |
| SLC12A5 | 2.800089 | 0.568235 | 6.04E-15 | 5.53E-14 |
| HOXC11 | 2.325364 | 2.395334 | 6.11E-15 | 5.60E-14 |
| ACOT12 | -3.38194 | -0.98149 | 6.20E-15 | 5.67E-14 |
| TBATA | -2.64054 | -3.74436 | 6.25E-15 | 5.72E-14 |
| C1orf127 | 3.002929 | -0.44465 | 6.29E-15 | 5.74E-14 |
| KLHL32 | -1.08446 | 1.217936 | 6.41E-15 | 5.86E-14 |
| DNAH3 | 2.524876 | 0.506555 | 6.48E-15 | 5.91E-14 |
| AKR1C2 | 3.527382 | 6.361049 | 6.49E-15 | 5.92E-14 |
| PELI2 | -1.05704 | 3.976331 | 6.63E-15 | 6.04E-14 |
| AURKA | 1.400037 | 2.775237 | 6.72E-15 | 6.12E-14 |
| TFR2 | 2.846836 | 0.248146 | 6.73E-15 | 6.13E-14 |
| SSH3 | 1.039414 | 6.242808 | 6.92E-15 | 6.29E-14 |
| CD300C | 1.923001 | 1.196837 | 7.02E-15 | 6.39E-14 |
| PLS3 | -1.24517 | 5.667906 | 7.04E-15 | 6.39E-14 |
| LCN6 | -2.30838 | -3.32967 | 7.18E-15 | 6.52E-14 |
| COL26A1 | -1.96649 | 2.020835 | 7.65E-15 | 6.95E-14 |
| FCGR2B | 2.903513 | 3.787045 | 7.70E-15 | 6.98E-14 |
| RHBDL3 | -1.80957 | -1.32192 | 7.75E-15 | 7.03E-14 |
| TDGF1 | -3.21539 | 1.994156 | 7.79E-15 | 7.06E-14 |
| CENPF | 2.546295 | 3.318149 | 8.18E-15 | 7.41E-14 |
| SIRPB1 | 2.846262 | 2.299051 | 8.20E-15 | 7.43E-14 |
| ASIC3 | 2.124282 | 1.275259 | 8.24E-15 | 7.46E-14 |
| TTC26 | 1.036081 | 4.403459 | 8.26E-15 | 7.47E-14 |
| E2F7 | 2.774404 | 0.114127 | 8.32E-15 | 7.52E-14 |
| GAPT | 2.012424 | 1.992302 | 8.63E-15 | 7.80E-14 |
| ANGPTL3 | -3.05469 | 3.262395 | 8.88E-15 | 8.01E-14 |
| ITLN2 | -2.74223 | -3.31576 | 9.33E-15 | 8.40E-14 |
| ADAMTS17 | -1.42634 | 0.830799 | 9.53E-15 | 8.58E-14 |
| COL4A3 | -1.40017 | 5.355908 | 9.55E-15 | 8.59E-14 |
| TRPM8 | 4.732617 | 2.83124 | 9.69E-15 | 8.71E-14 |
| ZFAT | -1.05144 | 4.039806 | 9.70E-15 | 8.72E-14 |
| C17orf97 | 1.352183 | 4.671987 | 9.88E-15 | 8.87E-14 |
| FAM43A | -1.64115 | 3.291883 | 9.92E-15 | 8.91E-14 |
| LGALS9 | 1.943103 | 5.455524 | 1.01E-14 | 9.06E-14 |
| CCZ1 | 1.120336 | 3.221983 | 1.03E-14 | 9.27E-14 |
| TRPC6 | -2.03351 | 0.407801 | 1.06E-14 | 9.49E-14 |
| SULT1C4 | 2.690679 | 6.654333 | 1.12E-14 | 9.99E-14 |
| ARHGAP45 | 1.257631 | 5.764601 | 1.12E-14 | 1.00E-13 |
| KREMEN2 | 2.779815 | -1.07983 | 1.14E-14 | 1.01E-13 |
| TNNI2 | 2.504366 | 0.170545 | 1.15E-14 | 1.02E-13 |
| GLYATL1 | -1.87085 | 6.129106 | 1.19E-14 | 1.06E-13 |
| ECSCR | -1.48987 | 0.634364 | 1.19E-14 | 1.06E-13 |
| CYR61 | -1.60487 | 7.001338 | 1.21E-14 | 1.08E-13 |
| IL20RB | 3.313638 | 1.264025 | 1.21E-14 | 1.08E-13 |
| IL11 | -2.16177 | -1.23042 | 1.26E-14 | 1.12E-13 |
| KLHL14 | -1.88254 | 2.61355 | 1.29E-14 | 1.15E-13 |
| DEPTOR | -1.22345 | 5.052511 | 1.32E-14 | 1.17E-13 |
| PYCR1 | 2.781679 | 4.16758 | 1.32E-14 | 1.17E-13 |
| COL9A2 | -1.77234 | 3.891586 | 1.32E-14 | 1.17E-13 |
| SRSF12 | 1.613125 | 2.189267 | 1.33E-14 | 1.18E-13 |
| LRRC36 | 2.403516 | 2.194932 | 1.34E-14 | 1.19E-13 |
| ITPR2 | -1.31545 | 5.469578 | 1.37E-14 | 1.21E-13 |
| ABAT | -1.74138 | 6.669248 | 1.40E-14 | 1.24E-13 |
| FGF7 | -2.35292 | 0.923902 | 1.41E-14 | 1.24E-13 |
| SYTL3 | 1.206943 | 3.610482 | 1.45E-14 | 1.28E-13 |
| ATP2A1 | 2.181677 | 1.723367 | 1.45E-14 | 1.28E-13 |
| GBP2 | 1.448128 | 6.188088 | 1.46E-14 | 1.28E-13 |
| FNDC9 | -1.89913 | -3.66318 | 1.46E-14 | 1.28E-13 |
| FBXO41 | 2.135906 | 3.319181 | 1.48E-14 | 1.31E-13 |
| LDLRAD3 | 1.412085 | 3.819548 | 1.50E-14 | 1.32E-13 |
| C12orf45 | 1.227951 | 2.84586 | 1.52E-14 | 1.34E-13 |
| CD86 | 1.961255 | 3.374908 | 1.53E-14 | 1.35E-13 |
| ADA | 1.485042 | 2.75662 | 1.55E-14 | 1.36E-13 |
| NAV2 | -1.25311 | 5.535434 | 1.55E-14 | 1.36E-13 |
| ITGA10 | -1.72506 | 0.024723 | 1.56E-14 | 1.37E-13 |
| TTC39C | 1.040722 | 3.346303 | 1.57E-14 | 1.37E-13 |
| GJA4 | -1.57835 | 3.150799 | 1.59E-14 | 1.39E-13 |
| PRAME | 3.689242 | 4.159365 | 1.59E-14 | 1.39E-13 |
| FXYD6 | -1.30946 | 4.498468 | 1.59E-14 | 1.39E-13 |
| PSMC3IP | 1.035396 | 2.214504 | 1.64E-14 | 1.44E-13 |
| C7orf57 | 2.322426 | 0.311536 | 1.66E-14 | 1.45E-13 |
| LST1 | 2.037321 | 3.776527 | 1.69E-14 | 1.48E-13 |
| MT1HL1 | -2.43663 | -4.08974 | 1.69E-14 | 1.48E-13 |
| SKA1 | 2.394947 | 0.106835 | 1.70E-14 | 1.48E-13 |
| DHRS9 | 3.306848 | 1.871391 | 1.73E-14 | 1.51E-13 |
| ENKD1 | 1.482841 | 5.868517 | 1.77E-14 | 1.54E-13 |
| CCDC40 | 1.368717 | 4.793283 | 1.77E-14 | 1.54E-13 |
| XRCC2 | 1.921751 | 0.832421 | 1.82E-14 | 1.58E-13 |
| HSPB6 | -1.97057 | 3.042866 | 1.85E-14 | 1.61E-13 |
| NPY1R | -1.61131 | 4.040347 | 1.89E-14 | 1.64E-13 |
| NLGN1 | 1.889357 | 4.617866 | 1.90E-14 | 1.65E-13 |
| FMNL1 | 1.425077 | 4.618684 | 1.95E-14 | 1.69E-13 |
| KCNS3 | 1.501112 | 4.688071 | 1.96E-14 | 1.70E-13 |
| PAQR5 | -1.34335 | 6.114452 | 1.98E-14 | 1.72E-13 |
| TRPA1 | 3.208382 | 1.278955 | 2.02E-14 | 1.75E-13 |
| CITED4 | 2.137703 | 5.67442 | 2.04E-14 | 1.77E-13 |
| IL18 | 1.43066 | 5.181256 | 2.06E-14 | 1.78E-13 |
| SLC1A4 | 1.10658 | 4.03905 | 2.16E-14 | 1.86E-13 |
| PIK3R5 | 2.274227 | 3.614365 | 2.17E-14 | 1.88E-13 |
| NEDD9 | -1.20744 | 6.01521 | 2.19E-14 | 1.89E-13 |
| HTR3A | 5.165381 | 1.199256 | 2.22E-14 | 1.92E-13 |
| TBX15 | 4.770455 | 3.071052 | 2.22E-14 | 1.92E-13 |
| CDK10 | 1.252026 | 7.008504 | 2.24E-14 | 1.93E-13 |
| PLPP3 | -1.22107 | 6.778345 | 2.24E-14 | 1.93E-13 |
| SKA3 | 2.030811 | 0.526791 | 2.26E-14 | 1.94E-13 |
| CNFN | 1.962901 | 1.242929 | 2.28E-14 | 1.96E-13 |
| PAK5 | -2.46366 | -2.02648 | 2.30E-14 | 1.97E-13 |
| TAZ | 1.087052 | 5.038305 | 2.31E-14 | 1.98E-13 |
| LSP1 | 2.129838 | 5.834873 | 2.34E-14 | 2.01E-13 |
| PBK | 2.563262 | 1.091917 | 2.37E-14 | 2.04E-13 |
| HS3ST1 | 1.988476 | 6.425023 | 2.38E-14 | 2.04E-13 |
| DRAM1 | 1.096005 | 6.589793 | 2.38E-14 | 2.04E-13 |
| VSIG10L | 1.256894 | 2.177689 | 2.50E-14 | 2.15E-13 |
| PGLS | 1.090655 | 6.202976 | 2.57E-14 | 2.20E-13 |
| AL139392.1 | -2.00529 | -2.67881 | 2.78E-14 | 2.37E-13 |
| HRK | 4.491582 | 3.549367 | 2.93E-14 | 2.50E-13 |
| AL355102.2 | -2.71336 | -3.91811 | 2.93E-14 | 2.50E-13 |
| ZNF582 | -1.01829 | 1.295472 | 2.93E-14 | 2.50E-13 |
| KL | -1.65279 | 6.912148 | 2.96E-14 | 2.52E-13 |
| SPATA16 | -2.63917 | -3.93769 | 2.99E-14 | 2.55E-13 |
| HOXD9 | -1.53729 | 3.798095 | 3.09E-14 | 2.63E-13 |
| NCF1 | 2.276331 | 2.126508 | 3.13E-14 | 2.66E-13 |
| PGGHG | 3.692314 | 8.648586 | 3.16E-14 | 2.68E-13 |
| GRM5 | 3.767984 | 1.071274 | 3.16E-14 | 2.68E-13 |
| PIDD1 | 1.218239 | 4.518766 | 3.21E-14 | 2.72E-13 |
| MEIS3 | 2.678968 | 3.761619 | 3.31E-14 | 2.80E-13 |
| RASGRF2 | -1.81109 | 2.79924 | 3.32E-14 | 2.81E-13 |
| DUSP2 | -1.93894 | 3.091779 | 3.33E-14 | 2.82E-13 |
| TRIM73 | 2.311571 | -0.29101 | 3.34E-14 | 2.83E-13 |
| CH25H | -1.81188 | 0.25851 | 3.37E-14 | 2.85E-13 |
| NPIPB6 | 2.994536 | -1.01651 | 3.43E-14 | 2.90E-13 |
| IL19 | -2.91647 | -3.83809 | 3.49E-14 | 2.95E-13 |
| SH3RF2 | 1.598869 | 3.187418 | 3.49E-14 | 2.95E-13 |
| GTSE1 | 2.111077 | 1.165506 | 3.50E-14 | 2.95E-13 |
| BAMBI | 1.740782 | 6.338787 | 3.62E-14 | 3.04E-13 |
| LILRB1 | 1.938482 | 2.919633 | 3.62E-14 | 3.05E-13 |
| MS4A14 | 2.619661 | 1.321607 | 3.71E-14 | 3.12E-13 |
| RPSA | 1.008643 | 9.03968 | 3.75E-14 | 3.15E-13 |
| MCCD1 | -3.27128 | 2.520485 | 3.77E-14 | 3.17E-13 |
| ARNTL2 | 1.781419 | 3.082718 | 3.81E-14 | 3.19E-13 |
| KIF4A | 2.360809 | 1.49504 | 3.84E-14 | 3.22E-13 |
| RNF175 | 2.250688 | 0.585364 | 3.94E-14 | 3.30E-13 |
| KIFC1 | 1.977443 | 2.090307 | 4.03E-14 | 3.37E-13 |
| GSAP | 1.216618 | 5.280771 | 4.08E-14 | 3.41E-13 |
| NKAIN1 | 2.382432 | -0.33032 | 4.14E-14 | 3.45E-13 |
| KIF14 | 2.867185 | 0.408979 | 4.17E-14 | 3.48E-13 |
| NANOS1 | -1.50634 | 1.073659 | 4.18E-14 | 3.49E-13 |
| RPS20 | 1.088409 | 9.574622 | 4.25E-14 | 3.54E-13 |
| HK3 | 2.308866 | 2.303527 | 4.32E-14 | 3.59E-13 |
| DNMT3B | 1.592699 | 3.295695 | 4.37E-14 | 3.64E-13 |
| C3orf70 | -1.7331 | 0.296927 | 4.40E-14 | 3.66E-13 |
| TAAR1 | -2.33975 | -4.19003 | 4.48E-14 | 3.72E-13 |
| GALE | 1.015533 | 6.310129 | 4.50E-14 | 3.74E-13 |
| MTCP1 | 1.509493 | 1.426735 | 4.59E-14 | 3.81E-13 |
| ERCC6L | 2.037458 | -0.56881 | 4.78E-14 | 3.96E-13 |
| PCDH15 | -3.46097 | -1.00671 | 4.83E-14 | 4.00E-13 |
| UBXN11 | 1.349717 | 5.687236 | 4.88E-14 | 4.03E-13 |
| GPR3 | -1.52414 | 0.216947 | 4.90E-14 | 4.05E-13 |
| HMGCS2 | -3.58753 | 5.101434 | 4.92E-14 | 4.06E-13 |
| F13A1 | -1.76735 | 4.126711 | 4.98E-14 | 4.11E-13 |
| MYBPH | -2.21759 | -1.2495 | 4.99E-14 | 4.11E-13 |
| ZNF273 | 1.528346 | 2.536651 | 4.99E-14 | 4.11E-13 |
| OXLD1 | 1.137118 | 5.180153 | 5.24E-14 | 4.31E-13 |
| GRASP | -1.339 | 2.850618 | 5.40E-14 | 4.44E-13 |
| PECAM1 | -1.29224 | 6.570805 | 5.44E-14 | 4.47E-13 |
| AMPH | -2.4852 | 0.981715 | 5.51E-14 | 4.53E-13 |
| TRPV6 | -2.64155 | 2.037575 | 5.58E-14 | 4.59E-13 |
| SLC25A15 | -1.04956 | 2.233123 | 5.84E-14 | 4.79E-13 |
| PLA2G3 | -3.342 | -3.24264 | 5.85E-14 | 4.80E-13 |
| TRIM74 | 2.720713 | -1.03219 | 5.88E-14 | 4.82E-13 |
| FAM3B | -2.75877 | 2.510318 | 6.00E-14 | 4.92E-13 |
| C1QB | 2.532042 | 7.326016 | 6.13E-14 | 5.02E-13 |
| PCSK4 | 1.477541 | 3.710466 | 6.18E-14 | 5.05E-13 |
| MAP3K12 | 1.226019 | 4.220309 | 6.48E-14 | 5.28E-13 |
| PRKRIP1 | 1.073174 | 5.50061 | 6.51E-14 | 5.31E-13 |
| CD72 | 1.734452 | 2.283296 | 6.67E-14 | 5.43E-13 |
| PSMB3 | 1.102871 | 7.195478 | 6.79E-14 | 5.52E-13 |
| RHBDL2 | 1.543652 | 1.508836 | 6.85E-14 | 5.57E-13 |
| RAPH1 | -1.42372 | 1.526777 | 6.88E-14 | 5.59E-13 |
| CPED1 | -1.64092 | 2.621635 | 6.90E-14 | 5.60E-13 |
| LILRA2 | 2.611973 | 2.645171 | 6.94E-14 | 5.63E-13 |
| ARHGAP11B | 1.395897 | 0.640698 | 6.99E-14 | 5.67E-13 |
| CASP1 | 1.319952 | 4.10779 | 6.99E-14 | 5.67E-13 |
| CCL23 | -1.6758 | -1.71802 | 7.48E-14 | 6.04E-13 |
| BEST4 | 1.928542 | 0.586862 | 7.62E-14 | 6.15E-13 |
| DOK4 | 1.012856 | 6.956379 | 7.75E-14 | 6.25E-13 |
| DPEP2 | 2.356117 | 2.809041 | 7.88E-14 | 6.35E-13 |
| NUF2 | 2.327119 | 0.706805 | 8.15E-14 | 6.56E-13 |
| PIGZ | 1.454687 | 2.888663 | 8.16E-14 | 6.56E-13 |
| DYNC2LI1 | 1.325422 | 6.431285 | 8.28E-14 | 6.65E-13 |
| AICDA | 3.358389 | -1.09399 | 8.32E-14 | 6.69E-13 |
| GPR84 | 2.044492 | -0.15164 | 8.34E-14 | 6.70E-13 |
| RIPOR3 | -1.71343 | 3.759508 | 8.57E-14 | 6.88E-13 |
| GABRB1 | -2.46054 | -2.70561 | 8.72E-14 | 6.99E-13 |
| APOBEC3H | 1.958038 | -0.25909 | 9.10E-14 | 7.29E-13 |
| ITGA1 | -1.16368 | 5.676681 | 9.30E-14 | 7.45E-13 |
| DEFB132 | -3.49505 | -3.89104 | 9.42E-14 | 7.53E-13 |
| TACC3 | 1.56452 | 4.196591 | 9.43E-14 | 7.54E-13 |
| DCC | -2.50186 | -1.82297 | 9.45E-14 | 7.55E-13 |
| TBC1D30 | -1.17595 | 2.212582 | 1.00E-13 | 8.00E-13 |
| KDELR3 | 1.320083 | 4.81749 | 1.00E-13 | 8.01E-13 |
| ZBBX | 2.429503 | 0.709632 | 1.01E-13 | 8.03E-13 |
| ANKRD29 | 1.658451 | 4.301316 | 1.03E-13 | 8.19E-13 |
| SNX22 | 1.840508 | 3.020641 | 1.05E-13 | 8.34E-13 |
| DNA2 | 1.514962 | 2.087103 | 1.05E-13 | 8.36E-13 |
| AHSA2 | 1.855304 | 5.929644 | 1.08E-13 | 8.60E-13 |
| KRT10 | 1.136561 | 4.997451 | 1.11E-13 | 8.85E-13 |
| C14orf80 | 1.280769 | 3.038681 | 1.11E-13 | 8.85E-13 |
| BEST3 | 1.796514 | 0.614156 | 1.14E-13 | 9.02E-13 |
| CHRNA5 | 2.522813 | 0.002042 | 1.14E-13 | 9.03E-13 |
| OIT3 | -2.46935 | 0.222169 | 1.14E-13 | 9.07E-13 |
| DLL3 | 4.126189 | -0.79504 | 1.18E-13 | 9.31E-13 |
| ZNF878 | -1.26457 | -1.6539 | 1.23E-13 | 9.69E-13 |
| RHPN1 | 1.295482 | 5.956158 | 1.23E-13 | 9.71E-13 |
| MMRN1 | -2.05984 | 0.914181 | 1.30E-13 | 1.03E-12 |
| MAP6 | -1.91069 | 2.865231 | 1.31E-13 | 1.03E-12 |
| LMO1 | -2.71493 | -1.16204 | 1.34E-13 | 1.05E-12 |
| ITGAM | 1.801792 | 4.332114 | 1.34E-13 | 1.05E-12 |
| TMEM106A | -1.16103 | 4.220946 | 1.35E-13 | 1.06E-12 |
| SRRM3 | 2.865628 | 3.018952 | 1.36E-13 | 1.07E-12 |
| NUSAP1 | 1.670863 | 3.387168 | 1.37E-13 | 1.08E-12 |
| XRCC3 | 1.125994 | 3.172656 | 1.37E-13 | 1.08E-12 |
| CLDN3 | 2.515204 | 7.870359 | 1.37E-13 | 1.08E-12 |
| GPRASP1 | -1.26771 | 3.558484 | 1.38E-13 | 1.08E-12 |
| CTXN1 | 1.739887 | 4.3402 | 1.38E-13 | 1.08E-12 |
| SLC27A3 | 1.368187 | 5.205977 | 1.40E-13 | 1.10E-12 |
| LGALS3BP | 1.199765 | 9.792845 | 1.49E-13 | 1.17E-12 |
| NUPR2 | -3.42612 | -0.32831 | 1.49E-13 | 1.17E-12 |
| GTF2H4 | 1.033898 | 1.808981 | 1.50E-13 | 1.17E-12 |
| WNT5A | 2.213106 | 5.340769 | 1.51E-13 | 1.18E-12 |
| LAMA3 | 2.451398 | 6.40904 | 1.52E-13 | 1.19E-12 |
| CDCA5 | 1.723739 | 2.04824 | 1.59E-13 | 1.24E-12 |
| ADAMTS7 | 1.545359 | 3.072777 | 1.59E-13 | 1.24E-12 |
| AC040162.1 | 1.539289 | 1.364278 | 1.60E-13 | 1.25E-12 |
| PLP2 | 1.099742 | 6.272982 | 1.61E-13 | 1.25E-12 |
| TMEM37 | -1.29287 | 6.296644 | 1.61E-13 | 1.26E-12 |
| MASP1 | 1.949938 | 5.720672 | 1.62E-13 | 1.27E-12 |
| HAVCR1 | 2.518145 | 5.893455 | 1.64E-13 | 1.28E-12 |
| APRT | 1.11429 | 7.008147 | 1.67E-13 | 1.30E-12 |
| DCHS1 | -1.39862 | 3.64408 | 1.69E-13 | 1.32E-12 |
| SLC51A | -1.03863 | 2.249305 | 1.70E-13 | 1.32E-12 |
| CSRNP1 | -1.30926 | 5.421785 | 1.71E-13 | 1.33E-12 |
| LUC7L | 1.569807 | 6.052736 | 1.78E-13 | 1.38E-12 |
| P2RX7 | 1.733358 | 3.025128 | 1.82E-13 | 1.41E-12 |
| LPIN3 | 1.188999 | 5.517608 | 1.84E-13 | 1.43E-12 |
| CDH17 | 4.083661 | 3.799583 | 1.88E-13 | 1.45E-12 |
| MILR1 | 2.217857 | 2.083681 | 1.90E-13 | 1.47E-12 |
| GYPA | -2.78622 | -1.35324 | 1.90E-13 | 1.47E-12 |
| MFAP3L | -1.04532 | 5.770044 | 1.96E-13 | 1.52E-12 |
| GRN | 1.162896 | 9.49116 | 1.97E-13 | 1.53E-12 |
| SMPDL3B | 1.352957 | 4.615701 | 2.02E-13 | 1.56E-12 |
| GAD1 | -2.38071 | 1.200633 | 2.07E-13 | 1.60E-12 |
| CLEC1A | -1.33867 | 0.547709 | 2.10E-13 | 1.62E-12 |
| SNED1 | -1.51626 | 3.144796 | 2.12E-13 | 1.64E-12 |
| AC068580.4 | 2.13015 | 0.863484 | 2.18E-13 | 1.68E-12 |
| UNC5A | 4.131541 | 2.229726 | 2.22E-13 | 1.71E-12 |
| HGF | -1.56032 | 2.607198 | 2.22E-13 | 1.71E-12 |
| SYNDIG1 | -1.80081 | 1.997075 | 2.28E-13 | 1.75E-12 |
| CCNE1 | 1.512404 | 1.386891 | 2.36E-13 | 1.81E-12 |
| MEI4 | -2.25472 | 1.401546 | 2.37E-13 | 1.82E-12 |
| CR1 | -2.01918 | 2.024084 | 2.38E-13 | 1.83E-12 |
| GAS2L3 | 2.132573 | 3.979998 | 2.43E-13 | 1.86E-12 |
| KRT85 | -2.83493 | -3.19792 | 2.45E-13 | 1.88E-12 |
| SYNE1 | -1.04225 | 6.991721 | 2.46E-13 | 1.88E-12 |
| CD37 | 1.890472 | 4.896804 | 2.48E-13 | 1.90E-12 |
| MZT2A | 1.235463 | 5.232191 | 2.53E-13 | 1.94E-12 |
| BCAN | 2.779686 | 2.538417 | 2.55E-13 | 1.94E-12 |
| DPYS | -2.35436 | 5.764171 | 2.63E-13 | 2.00E-12 |
| RPL28 | 1.127896 | 9.315708 | 2.65E-13 | 2.02E-12 |
| PANO1 | 1.33146 | -0.62002 | 2.70E-13 | 2.06E-12 |
| RAB38 | 1.169048 | 3.275613 | 2.76E-13 | 2.10E-12 |
| LILRB3 | 2.134268 | 1.432566 | 2.80E-13 | 2.13E-12 |
| HYDIN | 1.436846 | 3.677254 | 2.80E-13 | 2.13E-12 |
| MT1G | -3.21754 | 6.801298 | 2.82E-13 | 2.14E-12 |
| PKNOX2 | -2.03998 | 0.825284 | 2.87E-13 | 2.18E-12 |
| BIRC7 | 4.647458 | 3.057439 | 2.90E-13 | 2.20E-12 |
| GATA5 | -2.57254 | -0.39731 | 2.91E-13 | 2.21E-12 |
| CNR1 | 3.370155 | 2.320648 | 2.91E-13 | 2.21E-12 |
| HCFC1R1 | 1.420537 | 7.340653 | 2.93E-13 | 2.22E-12 |
| LAMC3 | -1.74904 | 3.701714 | 3.02E-13 | 2.29E-12 |
| BTBD19 | 1.436346 | 3.492182 | 3.08E-13 | 2.33E-12 |
| LDLRAD4 | -1.21941 | 3.384972 | 3.09E-13 | 2.34E-12 |
| CCNE2 | 1.306716 | 0.280116 | 3.22E-13 | 2.43E-12 |
| CSF2RA | 1.683906 | 3.314813 | 3.27E-13 | 2.47E-12 |
| DEPDC1 | 2.630879 | 0.285281 | 3.28E-13 | 2.48E-12 |
| LBHD1 | 1.121587 | 1.995119 | 3.44E-13 | 2.59E-12 |
| CLGN | 2.918528 | 2.017529 | 3.46E-13 | 2.61E-12 |
| PMFBP1 | 1.903197 | 0.083275 | 3.47E-13 | 2.61E-12 |
| RAB34 | 1.141714 | 7.027258 | 3.47E-13 | 2.61E-12 |
| GPR4 | -1.57151 | 2.361669 | 3.48E-13 | 2.62E-12 |
| TREH | -2.05052 | 3.101831 | 3.56E-13 | 2.68E-12 |
| CRYL1 | -1.01837 | 7.53612 | 3.66E-13 | 2.75E-12 |
| DGKA | 1.509398 | 4.496573 | 3.70E-13 | 2.78E-12 |
| AVPR2 | -2.84041 | 2.180017 | 3.75E-13 | 2.82E-12 |
| GALNT12 | 1.211697 | 4.16659 | 3.76E-13 | 2.82E-12 |
| EFCAB12 | 1.81844 | 3.012089 | 3.76E-13 | 2.82E-12 |
| AKR1C1 | 2.61503 | 8.210705 | 3.83E-13 | 2.87E-12 |
| QDPR | -1.05644 | 5.90781 | 3.83E-13 | 2.87E-12 |
| PCDH19 | -1.54398 | -1.15355 | 3.86E-13 | 2.89E-12 |
| CYP3A4 | -1.72952 | 0.007315 | 3.89E-13 | 2.91E-12 |
| SULT2B1 | -1.70179 | 1.234833 | 3.90E-13 | 2.91E-12 |
| ITGB8 | 1.834173 | 9.193642 | 3.93E-13 | 2.94E-12 |
| ASPG | -2.66773 | 1.914001 | 3.94E-13 | 2.95E-12 |
| CA12 | -1.74654 | 8.791192 | 3.97E-13 | 2.97E-12 |
| LNP1 | 2.154558 | 4.502053 | 3.99E-13 | 2.98E-12 |
| SPATA18 | 1.623384 | 5.412531 | 3.99E-13 | 2.98E-12 |
| TONSL | 1.027801 | 3.574042 | 4.01E-13 | 2.99E-12 |
| DENND2A | -1.80976 | 3.614539 | 4.01E-13 | 2.99E-12 |
| AGBL4 | -1.42248 | 0.738089 | 4.03E-13 | 3.00E-12 |
| XKR4 | 2.760245 | 3.051711 | 4.05E-13 | 3.02E-12 |
| C15orf48 | 2.225207 | 3.262531 | 4.11E-13 | 3.06E-12 |
| SMIM29 | 1.082992 | 4.827379 | 4.14E-13 | 3.08E-12 |
| CSTA | 2.469753 | 1.829308 | 4.27E-13 | 3.17E-12 |
| NME8 | 2.142194 | -1.1442 | 4.49E-13 | 3.33E-12 |
| AP003419.2 | 3.191404 | -2.81837 | 4.71E-13 | 3.50E-12 |
| CARD14 | 2.260848 | 1.643903 | 4.82E-13 | 3.57E-12 |
| NUDT10 | -1.96722 | -0.21661 | 4.84E-13 | 3.58E-12 |
| SULF2 | 1.415094 | 8.091008 | 4.90E-13 | 3.63E-12 |
| MARCO | 3.481569 | 3.34956 | 4.99E-13 | 3.69E-12 |
| NT5C | 1.15292 | 5.453004 | 5.10E-13 | 3.77E-12 |
| PCDH7 | -2.22783 | 1.695364 | 5.11E-13 | 3.77E-12 |
| ARHGAP11A | 1.413193 | 1.956805 | 5.25E-13 | 3.87E-12 |
| MEP1A | 4.335126 | -2.3928 | 5.26E-13 | 3.88E-12 |
| ALDH4A1 | -1.59008 | 7.326697 | 5.27E-13 | 3.89E-12 |
| CHRNB1 | 1.29447 | 4.860885 | 5.36E-13 | 3.95E-12 |
| TEPSIN | 1.158379 | 3.98888 | 5.42E-13 | 3.99E-12 |
| SLC7A11 | 4.280514 | 2.989879 | 5.42E-13 | 3.99E-12 |
| TNFAIP8L2 | 1.921263 | 2.377334 | 5.58E-13 | 4.09E-12 |
| ADAP2 | 1.253578 | 4.098171 | 5.61E-13 | 4.12E-12 |
| ARPC4-TTLL3 | 1.509816 | -0.81421 | 5.69E-13 | 4.17E-12 |
| CRYBG3 | -1.48975 | 3.181011 | 5.83E-13 | 4.27E-12 |
| FNDC5 | -1.70214 | 0.035363 | 5.98E-13 | 4.38E-12 |
| HLA-F | 1.277528 | 6.315024 | 6.18E-13 | 4.52E-12 |
| CD84 | 2.169138 | 4.012252 | 6.18E-13 | 4.52E-12 |
| AL159163.1 | 2.00448 | -1.78046 | 6.22E-13 | 4.54E-12 |
| ECHDC3 | -1.67175 | 5.213134 | 6.25E-13 | 4.56E-12 |
| BRCA2 | 1.421455 | 1.250429 | 6.33E-13 | 4.61E-12 |
| LRRN2 | -2.10822 | 3.47368 | 6.43E-13 | 4.69E-12 |
| TMEM150C | -1.35325 | 4.371206 | 6.48E-13 | 4.71E-12 |
| BRIP1 | 1.947584 | 1.225571 | 6.59E-13 | 4.79E-12 |
| ZNF276 | 1.023509 | 4.458333 | 6.59E-13 | 4.79E-12 |
| C1QC | 2.256685 | 6.998395 | 6.59E-13 | 4.79E-12 |
| SLC41A2 | 1.116357 | 4.764899 | 6.70E-13 | 4.86E-12 |
| SATB2 | 1.775828 | 5.786705 | 6.81E-13 | 4.94E-12 |
| ANO9 | 1.563376 | 4.824024 | 7.07E-13 | 5.13E-12 |
| HSD11B1 | -1.82515 | 0.075797 | 7.14E-13 | 5.17E-12 |
| CLDN11 | -2.16413 | 1.779253 | 7.18E-13 | 5.19E-12 |
| C2orf82 | 2.119646 | 2.125904 | 7.28E-13 | 5.27E-12 |
| RAC2 | 1.670384 | 4.980945 | 7.29E-13 | 5.27E-12 |
| SCX | 1.98698 | 1.097403 | 7.41E-13 | 5.35E-12 |
| BDKRB1 | -2.24709 | -0.75519 | 7.44E-13 | 5.37E-12 |
| MCM2 | 1.007055 | 4.746246 | 7.56E-13 | 5.45E-12 |
| S100A5 | -1.59054 | -1.72124 | 7.56E-13 | 5.46E-12 |
| GALNT1 | 1.037739 | 6.285092 | 7.58E-13 | 5.47E-12 |
| AQP6 | -3.20634 | 4.019517 | 7.60E-13 | 5.48E-12 |
| GAS2 | -1.3502 | 1.151865 | 7.76E-13 | 5.59E-12 |
| CDKN1A | 1.389837 | 8.382894 | 7.78E-13 | 5.60E-12 |
| CCDC88C | -1.15546 | 4.090016 | 7.93E-13 | 5.71E-12 |
| TP53I11 | -1.26789 | 4.776225 | 8.03E-13 | 5.77E-12 |
| APOL2 | 1.057262 | 6.28319 | 8.18E-13 | 5.87E-12 |
| ITGB2 | 1.983893 | 6.885456 | 8.40E-13 | 6.03E-12 |
| PACRG | -1.4228 | 2.034378 | 8.46E-13 | 6.07E-12 |
| CORO7-PAM16 | 2.078026 | -2.85246 | 8.55E-13 | 6.13E-12 |
| TRIM47 | 1.166509 | 6.150786 | 8.79E-13 | 6.29E-12 |
| EXPH5 | -1.23721 | 3.100242 | 8.89E-13 | 6.36E-12 |
| NUMBL | 1.167087 | 4.0927 | 9.06E-13 | 6.47E-12 |
| COL8A2 | 1.80302 | 3.709962 | 9.18E-13 | 6.55E-12 |
| LACTB2 | 1.180344 | 6.233525 | 9.20E-13 | 6.57E-12 |
| STIL | 1.190256 | 2.061163 | 9.32E-13 | 6.65E-12 |
| CST3 | 1.046047 | 8.915889 | 9.40E-13 | 6.70E-12 |
| DCBLD2 | 1.869261 | 7.392085 | 9.40E-13 | 6.70E-12 |
| LSMEM2 | -1.42064 | -2.47184 | 9.65E-13 | 6.87E-12 |
| FBXL6 | 1.071106 | 4.035659 | 9.67E-13 | 6.89E-12 |
| FZD7 | -1.4639 | 3.420415 | 9.86E-13 | 7.01E-12 |
| CACHD1 | -1.53458 | 3.093727 | 1.01E-12 | 7.15E-12 |
| TIMD4 | 1.770673 | 2.792097 | 1.03E-12 | 7.33E-12 |
| KCNT2 | 3.473599 | 4.304698 | 1.03E-12 | 7.34E-12 |
| ACAP3 | 1.018938 | 5.646214 | 1.05E-12 | 7.45E-12 |
| EGR2 | -1.77191 | 3.137584 | 1.06E-12 | 7.49E-12 |
| SLC45A2 | 2.845536 | 0.556228 | 1.06E-12 | 7.49E-12 |
| MYCNOS | -2.45633 | -3.07597 | 1.07E-12 | 7.58E-12 |
| FAM155B | 1.356239 | 3.178813 | 1.07E-12 | 7.58E-12 |
| STX1A | 1.238103 | 2.245343 | 1.09E-12 | 7.73E-12 |
| CAMK2N1 | -1.10002 | 6.454978 | 1.11E-12 | 7.89E-12 |
| FANK1 | 1.167259 | 3.576013 | 1.13E-12 | 7.98E-12 |
| SLC52A1 | 3.185465 | 2.225114 | 1.15E-12 | 8.16E-12 |
| COL27A1 | 1.350193 | 6.611966 | 1.16E-12 | 8.19E-12 |
| PPP1R14A | -1.57846 | 2.148816 | 1.16E-12 | 8.21E-12 |
| GPR37L1 | 1.541614 | -0.51601 | 1.18E-12 | 8.33E-12 |
| KCNA4 | -2.71123 | -3.34345 | 1.19E-12 | 8.43E-12 |
| EPN3 | -2.16721 | 2.960668 | 1.20E-12 | 8.44E-12 |
| SLC7A7 | -1.87033 | 6.234261 | 1.21E-12 | 8.53E-12 |
| RTP4 | 1.648209 | 3.562123 | 1.22E-12 | 8.58E-12 |
| CENPI | 1.699092 | -0.03515 | 1.22E-12 | 8.58E-12 |
| SARDH | -1.52642 | 4.307907 | 1.22E-12 | 8.58E-12 |
| CAMK1G | -1.52906 | 0.896515 | 1.23E-12 | 8.66E-12 |
| EPCAM | -1.20554 | 7.267165 | 1.26E-12 | 8.83E-12 |
| VSIG1 | 2.595061 | 0.130534 | 1.29E-12 | 9.08E-12 |
| ATP13A5 | -2.22049 | -2.86536 | 1.31E-12 | 9.20E-12 |
| NPC1 | 1.196395 | 6.399311 | 1.31E-12 | 9.20E-12 |
| E2F8 | 2.474336 | 0.342764 | 1.32E-12 | 9.23E-12 |
| RPL36A | 1.351897 | 5.804299 | 1.33E-12 | 9.32E-12 |
| SLC6A20 | 2.651335 | 5.830598 | 1.36E-12 | 9.52E-12 |
| ASPM | 2.820427 | 1.652895 | 1.37E-12 | 9.62E-12 |
| TTK | 2.072102 | 0.889659 | 1.38E-12 | 9.65E-12 |
| CST2 | 5.16746 | -0.46705 | 1.38E-12 | 9.69E-12 |
| KCTD4 | 2.798558 | 0.996411 | 1.40E-12 | 9.77E-12 |
| PLK2 | 1.720468 | 6.028129 | 1.42E-12 | 9.91E-12 |
| SLC5A11 | -2.40337 | 1.378947 | 1.45E-12 | 1.01E-11 |
| HLA-G | 2.674575 | 4.138121 | 1.46E-12 | 1.02E-11 |
| NCF2 | 1.967491 | 4.030776 | 1.47E-12 | 1.03E-11 |
| TMSB15B | 1.760076 | -1.72827 | 1.48E-12 | 1.03E-11 |
| NHSL1 | -1.03709 | 4.717848 | 1.51E-12 | 1.05E-11 |
| LCN2 | 3.663937 | 7.5667 | 1.57E-12 | 1.09E-11 |
| TMEM86B | 1.552713 | 1.86898 | 1.60E-12 | 1.11E-11 |
| ATG16L2 | 1.39913 | 4.849454 | 1.61E-12 | 1.11E-11 |
| CCDC113 | 1.14351 | 4.087124 | 1.61E-12 | 1.12E-11 |
| GATM | -1.68593 | 9.297232 | 1.64E-12 | 1.13E-11 |
| GRB14 | -1.51218 | 3.666821 | 1.65E-12 | 1.14E-11 |
| HIST1H2AG | 2.440634 | 0.320023 | 1.66E-12 | 1.15E-11 |
| ESCO2 | 2.171792 | 0.138024 | 1.66E-12 | 1.15E-11 |
| ALOX5AP | 1.728243 | 3.983718 | 1.67E-12 | 1.15E-11 |
| KLRD1 | 1.716638 | 1.845268 | 1.70E-12 | 1.17E-11 |
| IRX3 | 2.225311 | 6.541888 | 1.71E-12 | 1.18E-11 |
| DNAH2 | 2.353447 | 1.58741 | 1.79E-12 | 1.23E-11 |
| TOX | -1.78594 | 2.355286 | 1.80E-12 | 1.24E-11 |
| PON2 | 1.067486 | 6.812358 | 1.82E-12 | 1.25E-11 |
| NSMCE1 | 1.003479 | 6.514752 | 1.88E-12 | 1.29E-11 |
| SLC52A3 | -2.21485 | 2.949908 | 1.92E-12 | 1.32E-11 |
| A2M | -1.19774 | 8.807857 | 1.94E-12 | 1.33E-11 |
| NTRK2 | -2.19052 | 5.338037 | 1.97E-12 | 1.35E-11 |
| MMP20 | -2.29262 | -3.74797 | 1.98E-12 | 1.35E-11 |
| DGKH | 1.276074 | 5.170873 | 1.98E-12 | 1.36E-11 |
| PLOD2 | 1.201371 | 7.085451 | 1.98E-12 | 1.36E-11 |
| TMEM132A | 1.649122 | 5.662721 | 2.01E-12 | 1.38E-11 |
| PKIB | 1.675156 | 2.687613 | 2.02E-12 | 1.38E-11 |
| QRFPR | 2.840093 | 3.394217 | 2.05E-12 | 1.40E-11 |
| KLF8 | 1.377205 | 4.452311 | 2.07E-12 | 1.41E-11 |
| PDE6B | 1.448431 | 4.523106 | 2.08E-12 | 1.42E-11 |
| PTCRA | 2.768244 | -1.34785 | 2.10E-12 | 1.44E-11 |
| C1QTNF2 | -1.59549 | -0.83992 | 2.14E-12 | 1.46E-11 |
| CDH16 | -1.61273 | 9.057542 | 2.14E-12 | 1.46E-11 |
| RPL13 | 1.139869 | 10.09643 | 2.15E-12 | 1.46E-11 |
| ROMO1 | 1.25389 | 6.206129 | 2.15E-12 | 1.46E-11 |
| ZNF121 | 1.074726 | 4.730578 | 2.17E-12 | 1.48E-11 |
| PIK3AP1 | 1.276203 | 6.430755 | 2.18E-12 | 1.48E-11 |
| CYP3A5 | 2.331201 | 6.105857 | 2.19E-12 | 1.49E-11 |
| GRHL2 | -3.07864 | 2.231095 | 2.21E-12 | 1.50E-11 |
| MT1X | -1.95058 | 5.454296 | 2.24E-12 | 1.52E-11 |
| SLC13A5 | -2.50223 | -1.7486 | 2.25E-12 | 1.53E-11 |
| FAM69C | -2.04084 | -3.19619 | 2.25E-12 | 1.53E-11 |
| LTB4R2 | 1.354265 | 0.733506 | 2.30E-12 | 1.56E-11 |
| EPGN | -2.25468 | -3.43041 | 2.36E-12 | 1.60E-11 |
| PCDH11Y | -2.81239 | -3.24514 | 2.38E-12 | 1.61E-11 |
| LAT | 1.84218 | -0.13672 | 2.39E-12 | 1.62E-11 |
| PLCZ1 | -2.12678 | -3.13661 | 2.42E-12 | 1.64E-11 |
| HIST1H1E | -1.79032 | -1.94484 | 2.48E-12 | 1.68E-11 |
| PIP | 4.287284 | -0.99257 | 2.55E-12 | 1.72E-11 |
| TICRR | 2.057737 | 0.251313 | 2.56E-12 | 1.73E-11 |
| RPL37 | 1.020789 | 9.087334 | 2.56E-12 | 1.73E-11 |
| PDE4C | -1.52736 | 0.72459 | 2.57E-12 | 1.73E-11 |
| LYPD6 | -2.05645 | 0.592307 | 2.58E-12 | 1.74E-11 |
| CAVIN3 | 1.800521 | 5.306033 | 2.60E-12 | 1.75E-11 |
| DMRT2 | -3.78355 | 2.502402 | 2.61E-12 | 1.76E-11 |
| CCDC14 | 1.540106 | 5.605034 | 2.61E-12 | 1.76E-11 |
| LRGUK | 1.397651 | 0.856448 | 2.65E-12 | 1.78E-11 |
| SH3PXD2B | 1.663862 | 4.976614 | 2.68E-12 | 1.80E-11 |
| BTNL9 | -1.54495 | 2.47433 | 2.72E-12 | 1.83E-11 |
| JPT1 | 1.126438 | 6.36209 | 2.74E-12 | 1.84E-11 |
| SGSM1 | -1.49258 | 2.473632 | 2.75E-12 | 1.85E-11 |
| SLC16A7 | -1.50605 | 5.312885 | 2.78E-12 | 1.86E-11 |
| GCKR | 3.518491 | 1.150543 | 2.83E-12 | 1.90E-11 |
| SYCE3 | 2.209193 | 0.481028 | 2.89E-12 | 1.94E-11 |
| SOX9 | 1.779608 | 7.250448 | 2.94E-12 | 1.97E-11 |
| TP73 | 2.684187 | 0.470553 | 2.94E-12 | 1.97E-11 |
| TAL1 | -1.53659 | 1.113031 | 3.06E-12 | 2.04E-11 |
| C4orf47 | 1.775813 | 2.074487 | 3.06E-12 | 2.05E-11 |
| FAM167A | -1.66271 | 3.644774 | 3.08E-12 | 2.06E-11 |
| ITM2C | -1.08261 | 7.687002 | 3.14E-12 | 2.10E-11 |
| KIAA0319 | 2.705747 | 1.055998 | 3.21E-12 | 2.14E-11 |
| GRK1 | -1.84535 | -3.23896 | 3.22E-12 | 2.15E-11 |
| FAM89B | 1.013942 | 4.470809 | 3.22E-12 | 2.15E-11 |
| IFI30 | 1.633847 | 0.928605 | 3.23E-12 | 2.15E-11 |
| ZACN | 1.381429 | -0.28455 | 3.25E-12 | 2.17E-11 |
| AGRP | -1.58605 | -2.0294 | 3.35E-12 | 2.23E-11 |
| TRIM16 | 1.440465 | 4.000443 | 3.44E-12 | 2.28E-11 |
| SGIP1 | -1.88557 | 2.315866 | 3.45E-12 | 2.29E-11 |
| TACR1 | -1.89712 | 0.230315 | 3.46E-12 | 2.30E-11 |
| LSAMP | -1.91261 | 1.466225 | 3.47E-12 | 2.30E-11 |
| IL12RB2 | 3.779998 | 3.508843 | 3.49E-12 | 2.31E-11 |
| GIMAP5 | -1.3779 | 0.015319 | 3.50E-12 | 2.32E-11 |
| SPATA6L | 1.319497 | 2.414354 | 3.58E-12 | 2.37E-11 |
| WDR54 | 1.040356 | 4.896167 | 3.67E-12 | 2.43E-11 |
| EBI3 | 1.819371 | 2.151082 | 3.68E-12 | 2.44E-11 |
| FOSB | -2.37926 | 5.922548 | 3.71E-12 | 2.45E-11 |
| ADAMTS9 | 1.098425 | 6.33374 | 3.72E-12 | 2.46E-11 |
| PDE1A | -2.43122 | 4.888548 | 3.74E-12 | 2.47E-11 |
| NDC80 | 1.866218 | 1.105715 | 3.76E-12 | 2.48E-11 |
| MYEOV | 4.086102 | 1.304217 | 3.84E-12 | 2.54E-11 |
| HOXD4 | -1.58423 | 0.867316 | 3.86E-12 | 2.55E-11 |
| CDK5R1 | 1.363942 | 2.440909 | 3.87E-12 | 2.55E-11 |
| LRIT2 | 5.002065 | -1.05957 | 4.01E-12 | 2.64E-11 |
| DEF6 | 1.649291 | 3.482605 | 4.04E-12 | 2.66E-11 |
| CECR6 | 1.634987 | 0.087395 | 4.10E-12 | 2.70E-11 |
| CAPN12 | 2.077882 | 4.29793 | 4.27E-12 | 2.81E-11 |
| NME9 | 1.691907 | 1.500633 | 4.35E-12 | 2.86E-11 |
| EME2 | 1.374658 | 4.235207 | 4.36E-12 | 2.86E-11 |
| SIRPB2 | 1.824664 | 1.801579 | 4.36E-12 | 2.86E-11 |
| FCHO1 | 1.958607 | 2.3049 | 4.36E-12 | 2.87E-11 |
| AHR | 1.626177 | 6.98744 | 4.43E-12 | 2.90E-11 |
| UGT2A1 | -2.90016 | -0.10357 | 4.53E-12 | 2.97E-11 |
| SOX8 | 3.495572 | 2.939555 | 4.56E-12 | 2.99E-11 |
| PITPNM3 | -1.20014 | 2.31426 | 4.65E-12 | 3.04E-11 |
| IFI27L1 | 1.109027 | 3.586848 | 4.70E-12 | 3.07E-11 |
| AKR1D1 | 4.420416 | -1.09469 | 4.73E-12 | 3.09E-11 |
| CEBPA | 1.755966 | 4.038346 | 4.81E-12 | 3.14E-11 |
| ODAM | -3.67957 | -2.1238 | 4.85E-12 | 3.16E-11 |
| LHB | 3.053511 | -1.21418 | 4.90E-12 | 3.20E-11 |
| CAPG | 1.356594 | 8.541971 | 5.02E-12 | 3.27E-11 |
| CENPT | 1.038863 | 5.19525 | 5.05E-12 | 3.29E-11 |
| PLD4 | 1.691496 | 3.38581 | 5.07E-12 | 3.30E-11 |
| RPL36 | 1.115629 | 8.417766 | 5.13E-12 | 3.34E-11 |
| PIWIL1 | -2.303 | -2.82658 | 5.19E-12 | 3.38E-11 |
| MGAT3 | -1.83112 | 2.42618 | 5.28E-12 | 3.43E-11 |
| HMCN1 | -1.52939 | 2.251076 | 5.31E-12 | 3.45E-11 |
| ANK1 | 2.587281 | 3.680656 | 5.31E-12 | 3.45E-11 |
| DCXR | -1.31559 | 6.645778 | 5.32E-12 | 3.46E-11 |
| TCTEX1D4 | 1.685254 | -1.22456 | 5.41E-12 | 3.51E-11 |
| SCGN | 3.593177 | 4.061547 | 5.47E-12 | 3.55E-11 |
| TLR2 | 1.382814 | 4.27327 | 5.55E-12 | 3.60E-11 |
| MSC | 3.153075 | 5.458908 | 5.68E-12 | 3.68E-11 |
| TCF4 | -1.11907 | 4.184962 | 5.76E-12 | 3.72E-11 |
| NT5DC3 | 1.494757 | 4.507936 | 5.84E-12 | 3.77E-11 |
| ERFE | 2.98701 | 4.736499 | 5.93E-12 | 3.83E-11 |
| TNFRSF14 | 1.33235 | 6.918432 | 5.94E-12 | 3.83E-11 |
| TRIOBP | 1.039905 | 5.839852 | 6.01E-12 | 3.88E-11 |
| SCN10A | 4.355347 | -2.43478 | 6.02E-12 | 3.88E-11 |
| C1orf162 | 1.597837 | 3.665627 | 6.06E-12 | 3.90E-11 |
| MFAP4 | -1.75721 | 4.690305 | 6.18E-12 | 3.98E-11 |
| FBXO40 | -2.14734 | -3.28498 | 6.27E-12 | 4.03E-11 |
| SLAMF8 | 2.07589 | 3.479052 | 6.27E-12 | 4.03E-11 |
| PGBD5 | 1.751302 | 4.527804 | 6.33E-12 | 4.06E-11 |
| GPNMB | 3.007342 | 9.370856 | 6.50E-12 | 4.17E-11 |
| SYNPO | -1.39344 | 6.505766 | 6.51E-12 | 4.17E-11 |
| CR2 | -2.78243 | 1.774617 | 6.53E-12 | 4.18E-11 |
| EREG | 4.448344 | 2.148268 | 6.65E-12 | 4.25E-11 |
| C10orf99 | 5.814982 | 2.211878 | 6.70E-12 | 4.29E-11 |
| CDC20 | 1.920368 | 2.481171 | 6.73E-12 | 4.30E-11 |
| KCNJ3 | -1.56181 | 3.773543 | 6.84E-12 | 4.37E-11 |
| LENG9 | 1.135246 | 2.251623 | 6.95E-12 | 4.43E-11 |
| RHBG | -2.65446 | 2.849188 | 7.07E-12 | 4.51E-11 |
| IGFLR1 | 1.501896 | 1.288311 | 7.17E-12 | 4.57E-11 |
| PRSS57 | 3.070043 | -1.32645 | 7.22E-12 | 4.60E-11 |
| AFF2 | 2.292824 | 2.279787 | 7.34E-12 | 4.68E-11 |
| HMMR | 1.966244 | 1.499268 | 7.40E-12 | 4.71E-11 |
| NPIPB9 | 2.352994 | -2.48634 | 7.46E-12 | 4.74E-11 |
| ZP3 | 1.728904 | 2.790057 | 7.46E-12 | 4.74E-11 |
| TMEM200C | -2.20177 | 0.363872 | 7.50E-12 | 4.77E-11 |
| FABP6 | 4.995905 | 2.011549 | 7.62E-12 | 4.84E-11 |
| ELF3 | 1.262771 | 8.33569 | 7.74E-12 | 4.91E-11 |
| NIT2 | 1.224156 | 7.403052 | 7.78E-12 | 4.93E-11 |
| HMGA2 | 3.8606 | 1.95491 | 7.78E-12 | 4.93E-11 |
| C3AR1 | 1.86456 | 4.146479 | 7.82E-12 | 4.96E-11 |
| NPTXR | 1.530542 | 4.552163 | 8.10E-12 | 5.13E-11 |
| CLSPN | 1.852302 | 0.438806 | 8.21E-12 | 5.19E-11 |
| BSPRY | -1.38813 | 3.697437 | 8.37E-12 | 5.29E-11 |
| NTRK3 | -1.69301 | 2.02883 | 8.47E-12 | 5.35E-11 |
| NLGN2 | 1.226194 | 5.341436 | 8.73E-12 | 5.50E-11 |
| SELPLG | 1.720452 | 4.606768 | 8.83E-12 | 5.57E-11 |
| GATA3 | -2.51291 | 4.453168 | 9.36E-12 | 5.89E-11 |
| SERPINA4 | -2.1553 | 1.477258 | 9.43E-12 | 5.93E-11 |
| FCGR1B | 2.293345 | -0.25394 | 9.45E-12 | 5.94E-11 |
| ARNT2 | -1.21548 | 5.753282 | 9.47E-12 | 5.95E-11 |
| ACAD11 | -1.27498 | 1.323251 | 9.64E-12 | 6.06E-11 |
| PLVAP | -1.45874 | 6.271038 | 9.72E-12 | 6.10E-11 |
| IFI27L2 | 1.37162 | 5.10718 | 9.82E-12 | 6.16E-11 |
| CYP21A2 | 2.681853 | 1.345104 | 1.01E-11 | 6.32E-11 |
| SLC16A12 | -1.49127 | 6.322921 | 1.01E-11 | 6.32E-11 |
| PAH | -3.00718 | 6.375911 | 1.01E-11 | 6.32E-11 |
| MS4A6A | 1.817326 | 5.322353 | 1.02E-11 | 6.39E-11 |
| SPTLC3 | 1.152037 | 6.41769 | 1.05E-11 | 6.57E-11 |
| DNAAF3 | 1.474002 | 3.199901 | 1.06E-11 | 6.63E-11 |
| GLIPR1L2 | -1.22357 | 0.352787 | 1.08E-11 | 6.76E-11 |
| F3 | -1.68212 | 2.944787 | 1.08E-11 | 6.77E-11 |
| ZNF692 | 1.503913 | 4.798039 | 1.10E-11 | 6.84E-11 |
| TAGLN | -1.66 | 6.530751 | 1.12E-11 | 6.96E-11 |
| WAS | 1.575948 | 3.722003 | 1.14E-11 | 7.08E-11 |
| KAT2A | 1.394392 | 6.384199 | 1.14E-11 | 7.08E-11 |
| CA10 | -3.03439 | 1.745082 | 1.14E-11 | 7.10E-11 |
| SIGLEC7 | 2.282957 | 1.466315 | 1.15E-11 | 7.13E-11 |
| NFKBIZ | 1.957281 | 6.168349 | 1.17E-11 | 7.26E-11 |
| ADAMTSL4 | 1.468524 | 3.851284 | 1.18E-11 | 7.35E-11 |
| REP15 | 1.474788 | -0.54811 | 1.20E-11 | 7.45E-11 |
| STK39 | 1.047408 | 5.225757 | 1.20E-11 | 7.45E-11 |
| TBXAS1 | 1.595491 | 4.631512 | 1.21E-11 | 7.49E-11 |
| CELA1 | 2.870685 | -3.38903 | 1.22E-11 | 7.56E-11 |
| MSH5 | 1.715898 | 2.522444 | 1.23E-11 | 7.61E-11 |
| AATK | -1.33788 | 0.73469 | 1.24E-11 | 7.66E-11 |
| ANO2 | -1.49329 | -0.62242 | 1.27E-11 | 7.86E-11 |
| FXYD4 | -4.02278 | 5.025383 | 1.27E-11 | 7.87E-11 |
| CCDC65 | 1.173227 | 2.927724 | 1.28E-11 | 7.88E-11 |
| SCG2 | 3.253172 | 1.801002 | 1.28E-11 | 7.88E-11 |
| TRIM31 | 2.392187 | -0.66569 | 1.31E-11 | 8.10E-11 |
| SGCA | -1.68293 | 0.033572 | 1.33E-11 | 8.18E-11 |
| REEP6 | -1.34908 | 4.067321 | 1.36E-11 | 8.39E-11 |
| LILRA6 | 2.454632 | 1.001911 | 1.42E-11 | 8.77E-11 |
| PI3 | 4.03561 | 2.34559 | 1.43E-11 | 8.79E-11 |
| METTL26 | 1.147211 | 6.004632 | 1.45E-11 | 8.89E-11 |
| NAP1L2 | -1.459 | 2.613922 | 1.47E-11 | 9.03E-11 |
| SPC25 | 1.836085 | 0.493366 | 1.48E-11 | 9.08E-11 |
| KCNH2 | 2.711543 | 4.139088 | 1.51E-11 | 9.29E-11 |
| PROCA1 | 1.803345 | 2.946456 | 1.54E-11 | 9.46E-11 |
| RGS10 | 1.398349 | 3.993648 | 1.57E-11 | 9.59E-11 |
| DCST2 | 2.060784 | 0.61702 | 1.57E-11 | 9.61E-11 |
| VGF | 6.299879 | 4.869569 | 1.57E-11 | 9.63E-11 |
| KRTAP5-8 | -2.60723 | -1.9729 | 1.59E-11 | 9.70E-11 |
| TSNAXIP1 | 1.676172 | 3.191051 | 1.59E-11 | 9.71E-11 |
| ANO4 | 3.405324 | 2.293267 | 1.61E-11 | 9.87E-11 |
| CNKSR3 | 1.002827 | 6.672922 | 1.62E-11 | 9.91E-11 |
| DPCR1 | 2.200791 | -0.31355 | 1.64E-11 | 1.00E-10 |
| G6PD | 1.640456 | 6.706205 | 1.65E-11 | 1.01E-10 |
| TTLL3 | 1.718497 | 5.155102 | 1.65E-11 | 1.01E-10 |
| GPR150 | 3.252583 | -0.78359 | 1.68E-11 | 1.02E-10 |
| ARL11 | 1.808413 | 1.10617 | 1.71E-11 | 1.04E-10 |
| DRD1 | -2.50948 | -2.10582 | 1.72E-11 | 1.05E-10 |
| ZMAT3 | 1.072786 | 6.356108 | 1.72E-11 | 1.05E-10 |
| DCSTAMP | 3.374234 | 0.328436 | 1.74E-11 | 1.06E-10 |
| AL691442.1 | 3.231719 | -3.67461 | 1.75E-11 | 1.07E-10 |
| C1QA | 2.135312 | 7.131287 | 1.80E-11 | 1.09E-10 |
| TCN1 | 6.307155 | 2.79959 | 1.81E-11 | 1.10E-10 |
| AIFM3 | 1.659911 | 1.740753 | 1.81E-11 | 1.10E-10 |
| SLC22A12 | -2.50884 | 6.946271 | 1.83E-11 | 1.11E-10 |
| C21orf58 | 1.184921 | 1.801949 | 1.87E-11 | 1.13E-10 |
| MSR1 | 1.763316 | 4.913641 | 1.88E-11 | 1.14E-10 |
| ANXA2R | 1.460718 | 1.498059 | 1.97E-11 | 1.19E-10 |
| ADAMDEC1 | 3.242281 | 1.484192 | 1.98E-11 | 1.20E-10 |
| BUB1B | 1.706981 | 1.569409 | 1.98E-11 | 1.20E-10 |
| MYL5 | 1.090295 | 3.898075 | 1.99E-11 | 1.21E-10 |
| PLXNA4 | -2.06207 | 1.874796 | 2.00E-11 | 1.21E-10 |
| RTN4R | 1.423089 | 2.477899 | 2.00E-11 | 1.21E-10 |
| CNDP1 | -1.46048 | 1.012286 | 2.03E-11 | 1.23E-10 |
| LHFP | -1.25457 | 4.30349 | 2.03E-11 | 1.23E-10 |
| NFE2L3 | 1.183302 | 4.712546 | 2.05E-11 | 1.24E-10 |
| RIMBP2 | -2.81164 | 0.658916 | 2.07E-11 | 1.25E-10 |
| CAMKK1 | 1.152705 | 3.708483 | 2.09E-11 | 1.26E-10 |
| TMEM253 | 1.519856 | -1.4033 | 2.10E-11 | 1.27E-10 |
| ZNF83 | 1.452225 | 6.484633 | 2.17E-11 | 1.31E-10 |
| FXYD5 | 1.670088 | 6.04641 | 2.18E-11 | 1.31E-10 |
| SYNPR | -2.68075 | -4.09858 | 2.20E-11 | 1.32E-10 |
| MSX2 | 2.453231 | 0.737741 | 2.24E-11 | 1.35E-10 |
| RAB31 | 1.302659 | 6.43397 | 2.28E-11 | 1.37E-10 |
| AANAT | 1.721166 | -1.47291 | 2.32E-11 | 1.39E-10 |
| CHPF | 1.102063 | 7.758932 | 2.32E-11 | 1.39E-10 |
| DIO2 | -1.71732 | 1.980313 | 2.35E-11 | 1.41E-10 |
| SLC17A9 | 1.893132 | 2.586596 | 2.41E-11 | 1.44E-10 |
| P2RY11 | 1.06382 | 2.945626 | 2.42E-11 | 1.45E-10 |
| ANO3 | -1.77333 | 0.148131 | 2.42E-11 | 1.45E-10 |
| TTC21A | 1.292753 | 3.48421 | 2.43E-11 | 1.45E-10 |
| TRPC5 | -1.70089 | -2.90121 | 2.56E-11 | 1.53E-10 |
| SLC8A2 | 3.052045 | -0.36101 | 2.58E-11 | 1.54E-10 |
| CSMD3 | -2.71979 | -3.49304 | 2.58E-11 | 1.54E-10 |
| SMIM24 | -1.75803 | 7.00138 | 2.62E-11 | 1.56E-10 |
| OIP5 | 1.40442 | -0.33488 | 2.64E-11 | 1.57E-10 |
| MUC20 | -1.97926 | 4.331545 | 2.66E-11 | 1.58E-10 |
| TMSB4Y | -2.19958 | -0.48511 | 2.69E-11 | 1.60E-10 |
| APOL1 | 1.757081 | 8.042766 | 2.71E-11 | 1.61E-10 |
| POU5F1 | 2.132057 | 4.531156 | 2.72E-11 | 1.62E-10 |
| TNNT1 | 5.22862 | 2.081907 | 2.74E-11 | 1.63E-10 |
| SFN | 3.701546 | 5.981557 | 2.77E-11 | 1.64E-10 |
| CCDC136 | 1.429474 | 1.129963 | 2.78E-11 | 1.65E-10 |
| CARD11 | 1.988077 | 3.985994 | 2.79E-11 | 1.66E-10 |
| LRRC52 | -3.44371 | -2.57043 | 2.83E-11 | 1.68E-10 |
| GRIN2D | 2.05685 | 1.038134 | 2.87E-11 | 1.70E-10 |
| C7orf61 | 1.79083 | -1.10119 | 2.87E-11 | 1.70E-10 |
| C17orf100 | 1.110002 | 2.471864 | 2.89E-11 | 1.71E-10 |
| CLEC2D | 1.559848 | 3.071099 | 2.89E-11 | 1.71E-10 |
| TSPAN8 | -2.46397 | 3.698574 | 2.92E-11 | 1.73E-10 |
| NFAM1 | 1.78442 | 3.842895 | 2.99E-11 | 1.77E-10 |
| FGD3 | -1.12139 | 3.463673 | 3.02E-11 | 1.78E-10 |
| LPCAT1 | 1.160474 | 6.574065 | 3.03E-11 | 1.79E-10 |
| BHMT2 | -1.43996 | 7.352594 | 3.06E-11 | 1.81E-10 |
| WDR91 | 1.041509 | 6.98303 | 3.07E-11 | 1.81E-10 |
| PCDHGC5 | 2.212314 | -1.188 | 3.11E-11 | 1.84E-10 |
| ASPN | -1.64156 | 1.675073 | 3.15E-11 | 1.86E-10 |
| SMARCD3 | 1.231701 | 5.507671 | 3.20E-11 | 1.89E-10 |
| ITPKA | 2.5803 | -0.0375 | 3.30E-11 | 1.94E-10 |
| VAV1 | 1.696003 | 3.341709 | 3.32E-11 | 1.96E-10 |
| C11orf53 | -3.05329 | -3.36568 | 3.33E-11 | 1.96E-10 |
| AXL | 1.222963 | 6.135029 | 3.34E-11 | 1.96E-10 |
| LAMB1 | 1.144335 | 9.65767 | 3.34E-11 | 1.97E-10 |
| HEPACAM2 | -3.2842 | 2.269022 | 3.38E-11 | 1.99E-10 |
| TNFSF14 | 2.998455 | 2.27195 | 3.44E-11 | 2.02E-10 |
| MAP3K15 | -1.57162 | 1.671517 | 3.46E-11 | 2.03E-10 |
| MOBP | 2.36019 | -2.08526 | 3.47E-11 | 2.03E-10 |
| DNAAF1 | 1.958721 | 3.33157 | 3.47E-11 | 2.03E-10 |
| IFITM10 | 2.23678 | 5.02342 | 3.53E-11 | 2.06E-10 |
| AC005020.2 | -1.33832 | -2.67163 | 3.58E-11 | 2.09E-10 |
| SAPCD1 | 2.2597 | 0.191864 | 3.61E-11 | 2.11E-10 |
| AC137834.1 | 1.582579 | -2.16702 | 3.68E-11 | 2.15E-10 |
| RIBC2 | 1.715697 | -0.1368 | 3.80E-11 | 2.22E-10 |
| DNAI1 | 1.759763 | 2.078054 | 3.82E-11 | 2.23E-10 |
| DOK6 | -1.59031 | 2.180707 | 3.85E-11 | 2.25E-10 |
| ABCD2 | -1.56177 | -0.8894 | 3.86E-11 | 2.25E-10 |
| SCN1B | 1.813255 | 4.270156 | 3.93E-11 | 2.29E-10 |
| METRN | 1.344723 | 5.660624 | 4.05E-11 | 2.36E-10 |
| ATAD5 | 1.158422 | 1.83592 | 4.06E-11 | 2.37E-10 |
| SCARA5 | -2.24851 | -0.22843 | 4.08E-11 | 2.38E-10 |
| MALRD1 | -2.00634 | -0.71601 | 4.12E-11 | 2.40E-10 |
| CSTB | 1.199822 | 7.567211 | 4.15E-11 | 2.42E-10 |
| FIGN | 1.344114 | 4.240931 | 4.21E-11 | 2.45E-10 |
| CLEC7A | 1.562091 | 2.795888 | 4.25E-11 | 2.47E-10 |
| EHD2 | 1.257739 | 7.161992 | 4.28E-11 | 2.49E-10 |
| MYB | 1.93727 | -0.72819 | 4.31E-11 | 2.50E-10 |
| GIMAP1 | -1.12286 | 2.635757 | 4.35E-11 | 2.53E-10 |
| RND3 | 1.207006 | 5.653652 | 4.36E-11 | 2.53E-10 |
| IPCEF1 | -1.11138 | 2.512821 | 4.39E-11 | 2.54E-10 |
| PDGFA | 1.468102 | 6.397501 | 4.44E-11 | 2.57E-10 |
| KLHL6 | 1.970389 | 3.162702 | 4.45E-11 | 2.58E-10 |
| MND1 | 1.525997 | -0.45009 | 4.47E-11 | 2.59E-10 |
| GSDMB | 2.197118 | 3.30502 | 4.47E-11 | 2.59E-10 |
| UNC13D | 1.672397 | 3.929741 | 4.54E-11 | 2.63E-10 |
| SUCNR1 | -2.44552 | 4.109886 | 4.64E-11 | 2.69E-10 |
| CCDC74B | 1.770274 | 3.207163 | 4.74E-11 | 2.74E-10 |
| UGT2B7 | -1.42697 | 8.339338 | 4.76E-11 | 2.75E-10 |
| SUSD3 | -1.35883 | 3.252115 | 4.88E-11 | 2.81E-10 |
| CD33 | 1.56671 | 2.106502 | 4.96E-11 | 2.86E-10 |
| MRLN | -2.21741 | -0.61778 | 4.97E-11 | 2.86E-10 |
| FREM3 | -2.22279 | -2.88776 | 5.00E-11 | 2.88E-10 |
| LGALS9C | 2.75831 | -2.26491 | 5.04E-11 | 2.90E-10 |
| CALB2 | 2.453869 | 0.077756 | 5.05E-11 | 2.91E-10 |
| VGLL3 | -2.01442 | 1.622979 | 5.14E-11 | 2.95E-10 |
| FAM159A | 2.239024 | 0.535824 | 5.23E-11 | 3.00E-10 |
| SLC22A1 | -1.31436 | -0.09329 | 5.26E-11 | 3.02E-10 |
| CD180 | 2.037901 | 2.704674 | 5.27E-11 | 3.02E-10 |
| AC120114.5 | 1.610562 | -2.75794 | 5.46E-11 | 3.13E-10 |
| KRT80 | 1.527237 | 5.122941 | 5.48E-11 | 3.14E-10 |
| ADAM18 | 5.127745 | -3.01731 | 5.51E-11 | 3.15E-10 |
| KIAA0895L | 1.481216 | 4.972677 | 5.54E-11 | 3.17E-10 |
| PADI1 | 4.238288 | 1.383689 | 5.74E-11 | 3.28E-10 |
| H2AFJ | 1.137585 | 6.402803 | 5.80E-11 | 3.31E-10 |
| DOCK11 | 1.393039 | 5.927614 | 5.81E-11 | 3.31E-10 |
| MYO3A | 2.342829 | 2.551205 | 5.81E-11 | 3.31E-10 |
| PLCXD3 | -2.26684 | 2.386085 | 5.82E-11 | 3.32E-10 |
| CAPZA3 | -2.87511 | -3.75514 | 5.92E-11 | 3.37E-10 |
| LIPC | 2.578118 | 6.573935 | 5.94E-11 | 3.38E-10 |
| PILRA | 1.511172 | 3.450442 | 6.11E-11 | 3.47E-10 |
| EXO1 | 1.936856 | 0.491328 | 6.13E-11 | 3.48E-10 |
| GK3P | -1.4738 | -3.72308 | 6.13E-11 | 3.48E-10 |
| MROH6 | 1.561308 | 3.662828 | 6.14E-11 | 3.49E-10 |
| PRSS36 | 1.15752 | 1.365034 | 6.19E-11 | 3.51E-10 |
| EIF4EBP1 | 1.350834 | 4.884352 | 6.22E-11 | 3.53E-10 |
| EFHC1 | 1.092167 | 5.014166 | 6.28E-11 | 3.56E-10 |
| SPP1 | 1.466621 | 12.5416 | 6.29E-11 | 3.56E-10 |
| CKAP2L | 2.22271 | 0.586365 | 6.41E-11 | 3.63E-10 |
| ESPN | -1.71286 | 4.393225 | 6.46E-11 | 3.65E-10 |
| SAC3D1 | 1.020018 | 3.100526 | 6.66E-11 | 3.76E-10 |
| KRT33B | 3.407339 | -2.01527 | 6.68E-11 | 3.77E-10 |
| JAZF1 | 1.038583 | 4.36981 | 6.69E-11 | 3.77E-10 |
| CFAP52 | 1.69565 | 0.382396 | 6.69E-11 | 3.77E-10 |
| OVCH1 | -1.73344 | -3.64001 | 6.69E-11 | 3.77E-10 |
| KIF2C | 1.707739 | 2.026452 | 6.96E-11 | 3.92E-10 |
| RUNDC3A | 2.377013 | 3.035529 | 7.01E-11 | 3.95E-10 |
| ALDH3B1 | 1.120186 | 6.659021 | 7.06E-11 | 3.97E-10 |
| F12 | 2.144582 | 3.814792 | 7.26E-11 | 4.08E-10 |
| VIT | -2.43332 | -2.4425 | 7.33E-11 | 4.11E-10 |
| NCF4 | 1.668415 | 3.165637 | 7.37E-11 | 4.13E-10 |
| KLHL31 | 1.34816 | 0.470373 | 7.49E-11 | 4.20E-10 |
| CD302 | -1.19056 | 2.033651 | 7.55E-11 | 4.23E-10 |
| RAPGEF3 | -1.22956 | 5.892839 | 7.59E-11 | 4.25E-10 |
| NR1D1 | 1.160499 | 5.603319 | 7.61E-11 | 4.26E-10 |
| FXYD1 | -1.6772 | -0.93588 | 7.65E-11 | 4.28E-10 |
| ALK | 3.407981 | -0.06499 | 7.68E-11 | 4.29E-10 |
| ST6GALNAC5 | 3.593641 | 3.175471 | 7.87E-11 | 4.39E-10 |
| PSMG3 | 1.031432 | 5.173375 | 7.90E-11 | 4.41E-10 |
| C3 | 2.588262 | 10.70955 | 8.01E-11 | 4.47E-10 |
| CEP95 | 1.034795 | 5.20358 | 8.19E-11 | 4.56E-10 |
| TMEM246 | 1.259709 | 5.506302 | 8.22E-11 | 4.58E-10 |
| CLEC12A | 2.007961 | 0.774318 | 8.28E-11 | 4.61E-10 |
| KBTBD12 | -2.32587 | -0.3813 | 8.41E-11 | 4.68E-10 |
| ARHGAP9 | 1.590162 | 3.188435 | 8.50E-11 | 4.73E-10 |
| HAS2 | -1.98834 | -0.50594 | 9.07E-11 | 5.03E-10 |
| C15orf52 | 1.161151 | 5.13385 | 9.32E-11 | 5.17E-10 |
| APOD | -1.86467 | 2.755037 | 9.35E-11 | 5.18E-10 |
| TRIB3 | 2.178244 | 4.621976 | 9.48E-11 | 5.25E-10 |
| SLC16A11 | -1.78977 | 1.16185 | 9.49E-11 | 5.25E-10 |
| THBS1 | -1.39215 | 7.953654 | 9.79E-11 | 5.41E-10 |
| INHBE | 3.300798 | 2.146762 | 9.89E-11 | 5.46E-10 |
| TMPRSS6 | 2.388996 | 1.027037 | 9.96E-11 | 5.50E-10 |
| BCL11A | 1.540188 | 2.043898 | 1.00E-10 | 5.53E-10 |
| AKR1B10 | 6.385301 | 7.836008 | 1.00E-10 | 5.54E-10 |
| RPL35 | 1.021404 | 8.624018 | 1.01E-10 | 5.55E-10 |
| FAM57A | 1.082072 | 4.014469 | 1.03E-10 | 5.69E-10 |
| BATF | 2.421077 | 1.390398 | 1.04E-10 | 5.74E-10 |
| FABP4 | -2.37866 | 1.53551 | 1.05E-10 | 5.75E-10 |
| NR1H4 | 1.124886 | 5.520251 | 1.05E-10 | 5.75E-10 |
| EPOP | 1.532436 | 1.930164 | 1.06E-10 | 5.83E-10 |
| WFDC5 | 4.700643 | 2.658369 | 1.07E-10 | 5.86E-10 |
| COL24A1 | -1.70533 | -0.47503 | 1.10E-10 | 6.04E-10 |
| GLI4 | 1.062223 | 4.264991 | 1.11E-10 | 6.07E-10 |
| CCKBR | 3.157257 | -0.98556 | 1.12E-10 | 6.13E-10 |
| PLCB1 | -1.49668 | 4.106575 | 1.12E-10 | 6.14E-10 |
| B3GALT5 | 2.342807 | 4.303876 | 1.12E-10 | 6.14E-10 |
| CD40 | 1.179756 | 5.005079 | 1.13E-10 | 6.17E-10 |
| TINAGL1 | 1.031257 | 8.498876 | 1.13E-10 | 6.20E-10 |
| CTSS | 1.906805 | 6.870117 | 1.16E-10 | 6.33E-10 |
| TRIM16L | 1.591849 | 4.234715 | 1.19E-10 | 6.47E-10 |
| IFNE | 2.878972 | 0.001538 | 1.23E-10 | 6.72E-10 |
| FBF1 | 1.257101 | 3.244721 | 1.24E-10 | 6.78E-10 |
| TRIM40 | -2.46721 | -3.45863 | 1.24E-10 | 6.78E-10 |
| CCDC154 | 1.91489 | 1.363242 | 1.25E-10 | 6.82E-10 |
| RNF207 | 1.417053 | 4.776838 | 1.26E-10 | 6.84E-10 |
| WFDC12 | 4.552985 | 2.080738 | 1.26E-10 | 6.85E-10 |
| MS4A4E | 2.092024 | -0.24633 | 1.28E-10 | 6.98E-10 |
| STX1B | 1.516378 | 2.089469 | 1.28E-10 | 6.98E-10 |
| TMEM266 | -1.25815 | 1.073965 | 1.30E-10 | 7.06E-10 |
| CLECL1 | 1.886928 | -0.74046 | 1.30E-10 | 7.08E-10 |
| TLR6 | 1.529434 | 1.563794 | 1.31E-10 | 7.12E-10 |
| DOC2B | -1.97073 | 3.438774 | 1.31E-10 | 7.13E-10 |
| SH3RF3 | 1.401963 | 4.264469 | 1.32E-10 | 7.15E-10 |
| UPP1 | 1.113677 | 5.643137 | 1.34E-10 | 7.28E-10 |
| SALL3 | -3.49698 | 0.139475 | 1.34E-10 | 7.28E-10 |
| AC090004.1 | 1.83633 | -0.99633 | 1.34E-10 | 7.29E-10 |
| CDH4 | 3.118154 | 3.921333 | 1.36E-10 | 7.36E-10 |
| TRIM22 | 1.109109 | 5.835682 | 1.39E-10 | 7.51E-10 |
| LIPA | 1.225538 | 7.375487 | 1.40E-10 | 7.58E-10 |
| CDCP2 | -1.70242 | -4.05729 | 1.41E-10 | 7.63E-10 |
| SCD | 1.815532 | 7.087965 | 1.42E-10 | 7.67E-10 |
| RUFY4 | 2.880174 | -0.87681 | 1.43E-10 | 7.72E-10 |
| COL4A4 | -1.1535 | 5.642886 | 1.43E-10 | 7.72E-10 |
| ABCB6 | 1.16758 | 3.146175 | 1.43E-10 | 7.72E-10 |
| NKX2-4 | 7.255141 | -0.70003 | 1.45E-10 | 7.84E-10 |
| POLN | -1.09543 | 0.202997 | 1.47E-10 | 7.90E-10 |
| KLHL29 | 1.047546 | 4.194609 | 1.47E-10 | 7.90E-10 |
| PF4V1 | 3.013223 | 1.29307 | 1.49E-10 | 8.00E-10 |
| OMG | 4.098987 | 1.116442 | 1.49E-10 | 8.03E-10 |
| SIRPD | 2.595849 | -3.11466 | 1.50E-10 | 8.05E-10 |
| PAMR1 | -1.48777 | 1.819538 | 1.50E-10 | 8.07E-10 |
| CDH13 | -1.5623 | 3.103926 | 1.51E-10 | 8.11E-10 |
| BCL2L2-PABPN1 | 1.360232 | -1.14511 | 1.53E-10 | 8.21E-10 |
| GPR82 | 1.835687 | -0.01837 | 1.55E-10 | 8.30E-10 |
| APOL3 | -1.05931 | 3.851986 | 1.56E-10 | 8.39E-10 |
| FABP5 | 1.893369 | 2.746755 | 1.60E-10 | 8.57E-10 |
| KRT12 | 3.021474 | -2.16653 | 1.60E-10 | 8.59E-10 |
| USP2 | -1.4928 | 5.828688 | 1.61E-10 | 8.63E-10 |
| MYZAP | -2.0721 | 0.349774 | 1.66E-10 | 8.85E-10 |
| LYG1 | 2.354248 | 5.985181 | 1.71E-10 | 9.10E-10 |
| GRIP2 | -1.73655 | 1.328663 | 1.71E-10 | 9.13E-10 |
| SCNN1D | 1.651461 | 2.877313 | 1.73E-10 | 9.20E-10 |
| CNIH2 | 1.559852 | -0.5762 | 1.74E-10 | 9.29E-10 |
| NFKBID | 1.344337 | 2.952779 | 1.74E-10 | 9.30E-10 |
| KIAA1549L | -1.31389 | 1.591081 | 1.81E-10 | 9.62E-10 |
| ADAM22 | -1.0459 | 3.477679 | 1.81E-10 | 9.66E-10 |
| IGSF11 | -1.73835 | 1.886898 | 1.82E-10 | 9.71E-10 |
| PIK3R2 | 1.237335 | 0.878997 | 1.85E-10 | 9.85E-10 |
| PLAU | -1.33878 | 6.492435 | 1.85E-10 | 9.86E-10 |
| C4orf26 | 2.618496 | -2.08367 | 1.86E-10 | 9.89E-10 |
| ADD2 | 2.430471 | 1.051919 | 1.88E-10 | 9.98E-10 |
| ERRFI1 | -1.34643 | 6.058081 | 1.88E-10 | 1.00E-09 |
| SLC4A11 | -1.65226 | 4.138081 | 1.90E-10 | 1.01E-09 |
| CSF3 | -2.77863 | -2.24044 | 1.96E-10 | 1.04E-09 |
| HDAC10 | 1.092645 | 3.421568 | 1.97E-10 | 1.04E-09 |
| TMEM71 | 1.526536 | 1.747144 | 1.97E-10 | 1.04E-09 |
| XKR7 | 4.221131 | 0.105437 | 1.98E-10 | 1.05E-09 |
| KHDC1 | 1.403759 | 1.109052 | 1.99E-10 | 1.05E-09 |
| VSTM2A | 6.211261 | 4.809226 | 1.99E-10 | 1.05E-09 |
| ZG16B | 1.821737 | 0.223498 | 2.02E-10 | 1.07E-09 |
| SPEF1 | 1.40191 | 2.88218 | 2.07E-10 | 1.09E-09 |
| TGFBI | 1.559039 | 7.227577 | 2.08E-10 | 1.10E-09 |
| PLPP2 | 2.525466 | 5.183623 | 2.10E-10 | 1.11E-09 |
| C12orf75 | 1.121345 | 6.884068 | 2.12E-10 | 1.12E-09 |
| THBS2 | 1.837514 | 6.958856 | 2.12E-10 | 1.12E-09 |
| PPM1L | -1.06947 | 4.317645 | 2.13E-10 | 1.12E-09 |
| INMT | -1.47976 | 2.884662 | 2.13E-10 | 1.12E-09 |
| GRM8 | 2.071272 | 3.135755 | 2.15E-10 | 1.13E-09 |
| SLC26A1 | -1.18234 | 3.842731 | 2.18E-10 | 1.15E-09 |
| AMH | 2.997708 | 0.862505 | 2.19E-10 | 1.15E-09 |
| TNMD | -2.20273 | -0.66973 | 2.27E-10 | 1.19E-09 |
| DRD4 | 1.728073 | 0.704377 | 2.29E-10 | 1.20E-09 |
| RELL2 | 1.0595 | 1.73826 | 2.33E-10 | 1.22E-09 |
| DUSP23 | 1.177169 | 6.219083 | 2.35E-10 | 1.23E-09 |
| CDK5R2 | 3.566417 | -1.66082 | 2.36E-10 | 1.24E-09 |
| KIAA1161 | -1.07178 | 5.116256 | 2.36E-10 | 1.24E-09 |
| VTCN1 | -1.89269 | 4.652818 | 2.38E-10 | 1.25E-09 |
| WNT11 | -1.84847 | 0.517824 | 2.40E-10 | 1.26E-09 |
| CD1D | 1.332662 | 1.847908 | 2.44E-10 | 1.27E-09 |
| FAM78A | 1.440623 | 3.249885 | 2.45E-10 | 1.28E-09 |
| PRSS53 | 1.637014 | 0.75972 | 2.46E-10 | 1.29E-09 |
| MUC4 | -1.54597 | 0.807662 | 2.47E-10 | 1.29E-09 |
| MPP6 | 1.295005 | 5.396476 | 2.51E-10 | 1.31E-09 |
| CHST11 | 1.374375 | 4.665049 | 2.51E-10 | 1.31E-09 |
| CHGB | -1.90827 | 3.902087 | 2.52E-10 | 1.32E-09 |
| ADAMTSL5 | 2.06791 | 2.277903 | 2.54E-10 | 1.32E-09 |
| CDCA2 | 2.345274 | 0.696152 | 2.54E-10 | 1.33E-09 |
| BVES | -1.59672 | 1.064251 | 2.58E-10 | 1.35E-09 |
| SEZ6L | 4.139813 | 1.052436 | 2.59E-10 | 1.35E-09 |
| P2RY14 | -1.16247 | -0.20101 | 2.62E-10 | 1.36E-09 |
| C16orf74 | 2.176744 | 2.404611 | 2.62E-10 | 1.36E-09 |
| PRKG1 | -1.38573 | 2.687604 | 2.62E-10 | 1.36E-09 |
| BLM | 1.341711 | 0.902148 | 2.80E-10 | 1.45E-09 |
| IL4I1 | 1.895467 | 3.750994 | 2.80E-10 | 1.45E-09 |
| KCNS1 | 3.499519 | 3.450172 | 2.82E-10 | 1.46E-09 |
| ACTL10 | 1.266942 | 1.107847 | 2.87E-10 | 1.49E-09 |
| FAM205C | -2.10309 | -2.88649 | 2.87E-10 | 1.49E-09 |
| SLITRK5 | 2.812267 | 2.381143 | 2.89E-10 | 1.50E-09 |
| CCDC167 | 1.024277 | 3.429427 | 2.94E-10 | 1.52E-09 |
| KRT20 | 8.887013 | 3.898585 | 2.98E-10 | 1.54E-09 |
| MAPK8IP3 | 1.258396 | 6.361545 | 3.01E-10 | 1.56E-09 |
| PKDCC | 1.674244 | 5.520519 | 3.05E-10 | 1.58E-09 |
| MAFF | -1.13719 | 5.059022 | 3.13E-10 | 1.62E-09 |
| IGLON5 | 4.347927 | 0.19723 | 3.14E-10 | 1.62E-09 |
| NME3 | 1.078698 | 5.962432 | 3.15E-10 | 1.63E-09 |
| CYP1A2 | -2.63514 | -2.9028 | 3.16E-10 | 1.63E-09 |
| TRPM3 | -1.25622 | 4.524999 | 3.22E-10 | 1.66E-09 |
| OSBPL10 | 1.01861 | 6.219137 | 3.28E-10 | 1.69E-09 |
| NDUFAF8 | 1.035775 | 4.725558 | 3.28E-10 | 1.69E-09 |
| CCNL2 | 1.473645 | 6.991574 | 3.30E-10 | 1.70E-09 |
| FRG2C | -2.83163 | -0.55979 | 3.32E-10 | 1.71E-09 |
| RASSF4 | 1.293554 | 7.536938 | 3.32E-10 | 1.71E-09 |
| TXLNB | 2.284429 | 0.951698 | 3.34E-10 | 1.72E-09 |
| AC073610.3 | 1.555153 | -3.10618 | 3.40E-10 | 1.75E-09 |
| ANKLE1 | 1.362693 | -0.91598 | 3.42E-10 | 1.76E-09 |
| NEURL1B | -1.19037 | 3.663575 | 3.44E-10 | 1.77E-09 |
| CRMP1 | 1.323088 | 6.449582 | 3.44E-10 | 1.77E-09 |
| LRRC75B | 1.611985 | 6.044163 | 3.50E-10 | 1.80E-09 |
| CDC20B | -2.32543 | -0.96919 | 3.57E-10 | 1.83E-09 |
| ADAT2 | 1.108525 | 2.814266 | 3.61E-10 | 1.85E-09 |
| EMID1 | -1.15962 | 2.775796 | 3.62E-10 | 1.86E-09 |
| MORN5 | 1.956674 | 1.269257 | 3.62E-10 | 1.86E-09 |
| NCKAP1L | 1.612909 | 4.726028 | 3.66E-10 | 1.87E-09 |
| RGMA | -1.49937 | 1.07751 | 3.67E-10 | 1.88E-09 |
| FAIM2 | -1.51836 | 0.311853 | 3.68E-10 | 1.88E-09 |
| CCNO | 1.355236 | 2.825795 | 3.70E-10 | 1.90E-09 |
| DNAH1 | 1.395424 | 4.524444 | 3.77E-10 | 1.93E-09 |
| PRSS21 | 3.942482 | -0.63678 | 3.89E-10 | 1.99E-09 |
| VIP | -1.83023 | -2.8778 | 3.90E-10 | 1.99E-09 |
| SHC2 | 1.62211 | 5.243133 | 4.03E-10 | 2.05E-09 |
| AC010616.1 | 1.979823 | -2.45428 | 4.06E-10 | 2.07E-09 |
| TMPRSS9 | 1.693455 | -0.58564 | 4.11E-10 | 2.09E-09 |
| ZBED6CL | 1.050555 | 4.293615 | 4.12E-10 | 2.10E-09 |
| WDR38 | 2.505581 | -0.64527 | 4.19E-10 | 2.13E-09 |
| ARRDC2 | 1.119956 | 5.708658 | 4.20E-10 | 2.14E-09 |
| C9orf16 | 1.088917 | 6.057937 | 4.30E-10 | 2.19E-09 |
| ACOT4 | -1.37529 | 2.50706 | 4.46E-10 | 2.26E-09 |
| DCST1 | 1.853216 | -1.28977 | 4.50E-10 | 2.28E-09 |
| PALM | -1.09562 | 5.856543 | 4.52E-10 | 2.29E-09 |
| HOXA4 | 1.13159 | 3.018197 | 4.53E-10 | 2.29E-09 |
| PLA2G2D | 3.355032 | 2.71065 | 4.56E-10 | 2.31E-09 |
| LILRA4 | 2.932866 | 1.488636 | 4.57E-10 | 2.31E-09 |
| GIMAP7 | -1.1301 | 2.9171 | 4.57E-10 | 2.31E-09 |
| AL845331.2 | -1.99011 | -1.90394 | 4.59E-10 | 2.32E-09 |
| CHI3L2 | 2.840743 | 3.578267 | 4.69E-10 | 2.37E-09 |
| CEBPB | 1.368269 | 5.43322 | 4.71E-10 | 2.38E-09 |
| RNF223 | -2.16125 | -0.87911 | 4.78E-10 | 2.41E-09 |
| RSPH10B | 1.813022 | -2.529 | 4.78E-10 | 2.41E-09 |
| ARRDC5 | 2.000639 | -1.61419 | 4.85E-10 | 2.44E-09 |
| HBQ1 | 4.074526 | -1.95788 | 4.86E-10 | 2.45E-09 |
| RFX2 | 1.075136 | 3.684535 | 4.91E-10 | 2.47E-09 |
| RSPO3 | -2.04032 | 0.535878 | 4.93E-10 | 2.49E-09 |
| FAM83B | -2.67937 | 0.548179 | 5.22E-10 | 2.62E-09 |
| ADGRA3 | 1.009476 | 6.096623 | 5.29E-10 | 2.66E-09 |
| ERC2 | 1.330138 | 1.54638 | 5.31E-10 | 2.67E-09 |
| RELT | 1.129827 | 2.549003 | 5.31E-10 | 2.67E-09 |
| ENGASE | 1.132877 | 5.307832 | 5.33E-10 | 2.68E-09 |
| FGD2 | 1.53719 | 3.146828 | 5.35E-10 | 2.68E-09 |
| RNF148 | -1.19312 | -2.88892 | 5.36E-10 | 2.69E-09 |
| 10-Mar | -1.74188 | 0.111524 | 5.56E-10 | 2.78E-09 |
| AC093525.2 | 1.882824 | -1.7586 | 5.80E-10 | 2.90E-09 |
| NRTN | 1.552335 | 2.995214 | 5.92E-10 | 2.96E-09 |
| ATP2B3 | -2.4449 | -1.21165 | 5.95E-10 | 2.98E-09 |
| FAM173A | 1.130484 | 4.505079 | 6.01E-10 | 3.00E-09 |
| CST1 | 6.717475 | 0.314288 | 6.02E-10 | 3.00E-09 |
| ZNF771 | 1.167299 | 3.311482 | 6.04E-10 | 3.01E-09 |
| TRIM59 | 1.079342 | 2.166989 | 6.07E-10 | 3.03E-09 |
| FAM227A | 1.361938 | 2.665981 | 6.21E-10 | 3.09E-09 |
| HCK | 1.608804 | 4.0987 | 6.21E-10 | 3.09E-09 |
| WNT10B | 2.367728 | -0.40078 | 6.28E-10 | 3.12E-09 |
| IGF2BP3 | 4.28888 | 0.555983 | 6.32E-10 | 3.14E-09 |
| CYP2C9 | 2.449375 | 2.164257 | 6.41E-10 | 3.18E-09 |
| TDRD6 | 1.644359 | 1.268744 | 6.57E-10 | 3.26E-09 |
| SLC2A5 | -1.47093 | 4.646739 | 6.68E-10 | 3.31E-09 |
| ACRV1 | 1.683017 | -2.4879 | 6.68E-10 | 3.31E-09 |
| BAHCC1 | -1.16227 | 3.930646 | 6.71E-10 | 3.32E-09 |
| RNF224 | -1.30772 | -0.44682 | 6.74E-10 | 3.34E-09 |
| OSR1 | 1.660323 | 2.541794 | 6.83E-10 | 3.38E-09 |
| TUBAL3 | -2.63408 | 0.638742 | 6.85E-10 | 3.39E-09 |
| SPINK13 | 2.621453 | 0.230236 | 6.90E-10 | 3.41E-09 |
| ADGRF1 | -1.83253 | 5.098969 | 6.91E-10 | 3.42E-09 |
| ITGA2 | -1.11519 | 5.126996 | 7.07E-10 | 3.49E-09 |
| ART4 | -1.54661 | 0.434272 | 7.08E-10 | 3.50E-09 |
| RPS21 | 1.005124 | 8.188739 | 7.20E-10 | 3.56E-09 |
| OASL | 1.236246 | 2.393042 | 7.22E-10 | 3.56E-09 |
| PNLDC1 | -1.8227 | -2.04074 | 7.22E-10 | 3.56E-09 |
| LMNB1 | 1.237883 | 3.424802 | 7.25E-10 | 3.57E-09 |
| PTH1R | -1.57977 | 7.179554 | 7.31E-10 | 3.60E-09 |
| CLEC4A | 1.268166 | 1.196439 | 7.38E-10 | 3.63E-09 |
| NTN5 | 1.709591 | -0.10635 | 7.42E-10 | 3.65E-09 |
| PRR11 | 1.623729 | 1.789475 | 7.45E-10 | 3.66E-09 |
| CCDC18 | 1.071538 | 1.467897 | 7.63E-10 | 3.75E-09 |
| PRELID3A | 1.062129 | 0.735417 | 7.63E-10 | 3.75E-09 |
| PTX3 | -1.49951 | -0.02479 | 7.78E-10 | 3.82E-09 |
| BCHE | 2.353555 | 4.43406 | 7.85E-10 | 3.85E-09 |
| FAM26F | 1.602714 | 1.91323 | 7.88E-10 | 3.87E-09 |
| ARL4D | -1.27384 | 4.596299 | 7.90E-10 | 3.88E-09 |
| MIOX | -1.88841 | 7.85942 | 7.92E-10 | 3.88E-09 |
| KIFC2 | 1.325634 | 4.643131 | 7.92E-10 | 3.88E-09 |
| BIN1 | 1.267944 | 8.148626 | 7.94E-10 | 3.89E-09 |
| FAIM | 1.493985 | 4.781626 | 7.99E-10 | 3.91E-09 |
| HAS3 | 1.932256 | 2.878948 | 8.28E-10 | 4.05E-09 |
| BLNK | -1.25212 | 3.94036 | 8.52E-10 | 4.17E-09 |
| PTPRN | 2.979479 | -0.84961 | 8.68E-10 | 4.25E-09 |
| CCDC191 | 1.061346 | 4.464366 | 9.17E-10 | 4.48E-09 |
| SH3BP1 | 1.368238 | 3.518654 | 9.18E-10 | 4.48E-09 |
| PHGDH | -1.42981 | 5.575243 | 9.23E-10 | 4.50E-09 |
| CYP46A1 | -1.03127 | 0.524833 | 9.33E-10 | 4.55E-09 |
| TMEM52 | -1.23186 | -0.41235 | 9.34E-10 | 4.55E-09 |
| GBP4 | -1.30544 | 4.334894 | 9.47E-10 | 4.61E-09 |
| NOS2 | -1.37808 | 0.252393 | 9.54E-10 | 4.64E-09 |
| RFLNB | -1.14985 | 3.212074 | 9.56E-10 | 4.65E-09 |
| INSC | -1.99614 | -1.15768 | 9.58E-10 | 4.66E-09 |
| NOX4 | -1.47898 | 5.60361 | 9.91E-10 | 4.81E-09 |
| GOLGA6L9 | 1.591744 | 1.824184 | 9.92E-10 | 4.82E-09 |
| C19orf84 | 2.354555 | -2.22962 | 9.92E-10 | 4.82E-09 |
| LRRC4 | -1.74118 | 1.665501 | 1.00E-09 | 4.85E-09 |
| TNFRSF18 | 2.339067 | 0.722646 | 1.02E-09 | 4.92E-09 |
| REG3A | 6.738847 | -1.0756 | 1.02E-09 | 4.93E-09 |
| APCDD1 | -1.58241 | 2.650285 | 1.02E-09 | 4.95E-09 |
| SLC9C1 | 1.910508 | 0.145348 | 1.03E-09 | 4.97E-09 |
| SLC2A14 | 2.418404 | -0.35811 | 1.04E-09 | 5.03E-09 |
| MROH2B | -2.04533 | -3.8024 | 1.06E-09 | 5.13E-09 |
| PSAT1 | -1.74144 | 5.431116 | 1.07E-09 | 5.18E-09 |
| MCC | -1.11052 | 3.965838 | 1.09E-09 | 5.27E-09 |
| FAM43B | -1.5307 | 0.378066 | 1.09E-09 | 5.27E-09 |
| LRRK1 | 1.215256 | 4.405452 | 1.11E-09 | 5.35E-09 |
| C1orf228 | 1.5829 | 1.451104 | 1.12E-09 | 5.41E-09 |
| OXTR | 2.186972 | 2.807815 | 1.15E-09 | 5.52E-09 |
| BOLA2B | 1.391541 | -0.66807 | 1.16E-09 | 5.57E-09 |
| SDK1 | 1.16231 | 5.503365 | 1.17E-09 | 5.62E-09 |
| TEN1-CDK3 | 1.359403 | 0.949076 | 1.18E-09 | 5.69E-09 |
| GPT | -1.51924 | 4.299653 | 1.20E-09 | 5.74E-09 |
| ADGRE1 | 1.730866 | 0.675706 | 1.21E-09 | 5.80E-09 |
| SLFNL1 | 1.844635 | -0.27037 | 1.21E-09 | 5.81E-09 |
| SLC15A3 | 1.625722 | 5.389929 | 1.21E-09 | 5.81E-09 |
| ZNF320 | 1.091829 | 5.750256 | 1.22E-09 | 5.83E-09 |
| ITIH5 | -1.76024 | 5.130548 | 1.22E-09 | 5.84E-09 |
| NCEH1 | 1.071132 | 6.769866 | 1.22E-09 | 5.84E-09 |
| STAT4 | 1.478331 | 1.949092 | 1.22E-09 | 5.86E-09 |
| PERP | 1.144822 | 7.545238 | 1.23E-09 | 5.88E-09 |
| MMP15 | 1.372617 | 7.00801 | 1.23E-09 | 5.89E-09 |
| CXCL1 | 2.512698 | 4.521094 | 1.26E-09 | 6.01E-09 |
| CCDC84 | 1.437434 | 4.41162 | 1.27E-09 | 6.09E-09 |
| LIMS2 | -1.07693 | 4.862332 | 1.28E-09 | 6.12E-09 |
| SPOCD1 | 3.507973 | 1.288163 | 1.28E-09 | 6.13E-09 |
| SOX30 | -1.55966 | -1.26004 | 1.29E-09 | 6.15E-09 |
| RDH8 | -3.86346 | 0.306323 | 1.30E-09 | 6.19E-09 |
| GDF5 | 2.305997 | 0.681657 | 1.34E-09 | 6.39E-09 |
| SNPH | 1.098774 | 4.036097 | 1.35E-09 | 6.40E-09 |
| CLDN4 | 1.25546 | 8.957055 | 1.37E-09 | 6.52E-09 |
| GOLGA8B | 1.891727 | 5.219409 | 1.40E-09 | 6.63E-09 |
| PRKCG | 4.315257 | -0.04659 | 1.40E-09 | 6.66E-09 |
| RNF133 | -1.27463 | -3.20141 | 1.41E-09 | 6.68E-09 |
| OAF | 1.03265 | 7.418349 | 1.42E-09 | 6.73E-09 |
| FGF18 | -1.44472 | -0.81516 | 1.42E-09 | 6.75E-09 |
| GABBR2 | 2.58968 | -0.68246 | 1.43E-09 | 6.79E-09 |
| CXCR4 | 1.57411 | 6.082918 | 1.44E-09 | 6.82E-09 |
| TBX19 | 1.06249 | 1.911622 | 1.45E-09 | 6.87E-09 |
| ADCY7 | 1.398262 | 3.04694 | 1.46E-09 | 6.91E-09 |
| NKX2-8 | 4.993098 | -1.88122 | 1.47E-09 | 6.93E-09 |
| ATP6V1G3 | -3.97714 | 1.040762 | 1.48E-09 | 7.00E-09 |
| PRICKLE1 | 1.512113 | 4.75115 | 1.51E-09 | 7.11E-09 |
| ERP27 | -1.86424 | 3.527532 | 1.54E-09 | 7.26E-09 |
| GRAPL | -1.51012 | -3.91683 | 1.55E-09 | 7.31E-09 |
| GPR20 | -1.4304 | -2.04821 | 1.57E-09 | 7.37E-09 |
| PAEP | 9.850552 | 2.734954 | 1.58E-09 | 7.45E-09 |
| TUNAR | 3.956753 | 3.89717 | 1.60E-09 | 7.53E-09 |
| NOD2 | 1.485099 | 1.262291 | 1.61E-09 | 7.55E-09 |
| GADD45B | -1.24431 | 6.13757 | 1.62E-09 | 7.62E-09 |
| PPP1R14C | 1.795155 | 5.19339 | 1.63E-09 | 7.64E-09 |
| C1QTNF9 | -1.36986 | -3.12051 | 1.63E-09 | 7.66E-09 |
| KCNQ3 | 1.442412 | 3.361999 | 1.63E-09 | 7.67E-09 |
| HIST3H2BB | 2.219008 | -0.8797 | 1.65E-09 | 7.73E-09 |
| FAM177B | 1.61091 | 0.023223 | 1.69E-09 | 7.92E-09 |
| XAGE2 | -2.43466 | -4.02342 | 1.70E-09 | 7.98E-09 |
| TMSB15A | -1.4955 | -1.69917 | 1.70E-09 | 7.98E-09 |
| C6 | 3.319234 | 5.051716 | 1.71E-09 | 7.99E-09 |
| ALX1 | -1.97512 | -1.16719 | 1.74E-09 | 8.16E-09 |
| C5orf38 | -2.1154 | 0.956011 | 1.77E-09 | 8.26E-09 |
| ATP5J2-PTCD1 | 1.390619 | -2.07503 | 1.78E-09 | 8.33E-09 |
| CD101 | 1.045139 | 0.738058 | 1.80E-09 | 8.40E-09 |
| VCAM1 | 1.786948 | 8.879829 | 1.80E-09 | 8.42E-09 |
| SLAMF9 | 3.447094 | -2.07883 | 1.85E-09 | 8.63E-09 |
| FBXO43 | 1.598573 | -1.02192 | 1.86E-09 | 8.69E-09 |
| PROM2 | -2.58248 | 5.707011 | 1.87E-09 | 8.71E-09 |
| SCIMP | 1.516679 | 2.434319 | 1.87E-09 | 8.72E-09 |
| C8orf46 | 1.426999 | 2.575577 | 1.88E-09 | 8.74E-09 |
| FAM46C | -1.19653 | 3.998572 | 1.88E-09 | 8.75E-09 |
| LTBP4 | -1.04264 | 6.249346 | 1.90E-09 | 8.83E-09 |
| ADGRB3 | -1.50163 | -0.46538 | 1.91E-09 | 8.89E-09 |
| NPNT | -1.62545 | 6.328369 | 1.92E-09 | 8.92E-09 |
| SASH3 | 1.578096 | 3.996736 | 1.97E-09 | 9.15E-09 |
| PTPRH | 2.465649 | 2.048861 | 1.99E-09 | 9.23E-09 |
| RANBP17 | 1.262258 | 2.043122 | 1.99E-09 | 9.23E-09 |
| FERMT3 | 1.465675 | 4.550015 | 2.01E-09 | 9.33E-09 |
| EGR1 | -1.49607 | 8.208585 | 2.06E-09 | 9.53E-09 |
| TFF3 | -1.79821 | 0.796047 | 2.06E-09 | 9.54E-09 |
| OMD | -2.14889 | -0.77883 | 2.12E-09 | 9.79E-09 |
| HIST1H4I | 1.344035 | 1.850235 | 2.16E-09 | 9.99E-09 |
| SHH | 1.590567 | 4.841475 | 2.18E-09 | 1.01E-08 |
| ITPRIPL1 | 1.510624 | 0.712836 | 2.20E-09 | 1.02E-08 |
| MAPK15 | 2.067046 | 4.623049 | 2.21E-09 | 1.02E-08 |
| AGXT2 | -1.95381 | 5.947441 | 2.22E-09 | 1.02E-08 |
| DLL4 | -1.16387 | 3.2614 | 2.22E-09 | 1.02E-08 |
| GNA15 | 1.581241 | 2.607239 | 2.22E-09 | 1.02E-08 |
| RASSF6 | 1.010829 | 5.153517 | 2.37E-09 | 1.09E-08 |
| SIGLEC11 | -1.3303 | 0.033932 | 2.39E-09 | 1.10E-08 |
| MGAM | -2.19821 | 5.003826 | 2.44E-09 | 1.12E-08 |
| CLVS1 | 2.473891 | -1.16833 | 2.45E-09 | 1.12E-08 |
| FSTL4 | -2.21252 | 1.495473 | 2.48E-09 | 1.14E-08 |
| SAGE1 | 4.849617 | -2.32574 | 2.48E-09 | 1.14E-08 |
| TDRD5 | -1.82336 | -0.16054 | 2.51E-09 | 1.15E-08 |
| CXCL5 | 3.866421 | 3.590305 | 2.51E-09 | 1.15E-08 |
| PLAUR | 1.394404 | 4.332983 | 2.52E-09 | 1.15E-08 |
| SYT1 | -1.9638 | 0.417211 | 2.53E-09 | 1.16E-08 |
| TEX11 | 3.439634 | 1.171872 | 2.53E-09 | 1.16E-08 |
| TMEM200A | 1.231666 | 5.504673 | 2.55E-09 | 1.17E-08 |
| PIF1 | 1.714734 | -0.10576 | 2.57E-09 | 1.17E-08 |
| AGAP2 | 1.360801 | 1.749411 | 2.60E-09 | 1.19E-08 |
| CXCL6 | 3.211193 | 5.160476 | 2.61E-09 | 1.19E-08 |
| ARL4C | 1.792257 | 6.791191 | 2.64E-09 | 1.20E-08 |
| C3orf35 | 1.529099 | -0.35762 | 2.66E-09 | 1.21E-08 |
| NPIPB13 | 3.219729 | -0.60682 | 2.69E-09 | 1.23E-08 |
| CIDEA | -3.27451 | -2.78468 | 2.70E-09 | 1.23E-08 |
| RPRM | -2.15167 | -0.59742 | 2.72E-09 | 1.24E-08 |
| ANXA13 | 2.584664 | 5.13351 | 2.72E-09 | 1.24E-08 |
| RARRES3 | 1.188783 | 6.422891 | 2.72E-09 | 1.24E-08 |
| MAFA | -1.91341 | -2.75416 | 2.74E-09 | 1.24E-08 |
| TIA1 | 1.054849 | 6.331403 | 2.76E-09 | 1.25E-08 |
| CDH24 | 1.001312 | 3.183535 | 2.76E-09 | 1.25E-08 |
| TNXB | -1.22041 | 3.97761 | 2.76E-09 | 1.25E-08 |
| WISP1 | -1.32703 | 1.742987 | 2.81E-09 | 1.28E-08 |
| KLF1 | 2.460517 | -3.12018 | 2.84E-09 | 1.29E-08 |
| TESC | 1.913415 | 5.569666 | 2.87E-09 | 1.30E-08 |
| CD244 | 1.625208 | 0.55195 | 2.88E-09 | 1.30E-08 |
| SPON1 | 2.588919 | 7.610312 | 2.90E-09 | 1.31E-08 |
| RETN | 2.878542 | -1.6322 | 2.91E-09 | 1.32E-08 |
| PLEKHH2 | 2.034142 | 6.922118 | 2.95E-09 | 1.33E-08 |
| ENTPD2 | 1.720215 | 4.731065 | 2.96E-09 | 1.34E-08 |
| ATXN7L2 | 1.293215 | 3.570641 | 2.97E-09 | 1.34E-08 |
| TEKT4 | 2.170878 | -1.83064 | 2.97E-09 | 1.34E-08 |
| HPGDS | 1.392992 | 1.467973 | 2.99E-09 | 1.35E-08 |
| PSTPIP1 | 1.602066 | 2.256739 | 3.08E-09 | 1.39E-08 |
| TTLL2 | 2.665484 | -0.04283 | 3.12E-09 | 1.41E-08 |
| PIFO | 1.054417 | 4.293343 | 3.13E-09 | 1.41E-08 |
| TREM1 | 2.428921 | 1.956097 | 3.14E-09 | 1.41E-08 |
| CENPE | 1.542304 | 1.149267 | 3.26E-09 | 1.47E-08 |
| COLQ | 1.162855 | 1.880208 | 3.28E-09 | 1.47E-08 |
| OSBPL6 | -1.43526 | 1.207768 | 3.29E-09 | 1.48E-08 |
| CYP39A1 | -1.16228 | 2.251425 | 3.35E-09 | 1.51E-08 |
| RNASE3 | 2.326912 | -3.17796 | 3.36E-09 | 1.51E-08 |
| SLC1A6 | 6.458376 | 2.250597 | 3.37E-09 | 1.51E-08 |
| PLA2G4F | -2.60264 | 3.469526 | 3.49E-09 | 1.57E-08 |
| ZNF789 | 1.078915 | 3.990738 | 3.50E-09 | 1.57E-08 |
| KCP | 1.326206 | 6.554617 | 3.53E-09 | 1.58E-08 |
| CCR5 | 1.638037 | 2.824993 | 3.55E-09 | 1.59E-08 |
| TNIP3 | 2.88733 | -0.18034 | 3.55E-09 | 1.59E-08 |
| EHHADH | -1.13024 | 6.880847 | 3.56E-09 | 1.59E-08 |
| TMEM262 | 1.019661 | 0.214317 | 3.57E-09 | 1.60E-08 |
| CD5L | 4.429211 | 0.64434 | 3.68E-09 | 1.64E-08 |
| RELN | 2.878311 | 5.906811 | 3.68E-09 | 1.64E-08 |
| TRABD2B | 1.229944 | 6.853682 | 3.79E-09 | 1.69E-08 |
| LGALS3 | 1.23129 | 7.812198 | 3.79E-09 | 1.69E-08 |
| SYT9 | -1.84174 | 1.028574 | 3.80E-09 | 1.70E-08 |
| PNCK | 3.038052 | 2.180835 | 3.83E-09 | 1.71E-08 |
| IL1R2 | -1.6963 | 1.944845 | 3.90E-09 | 1.74E-08 |
| LMNTD2 | 1.335025 | 3.408903 | 3.92E-09 | 1.74E-08 |
| ADGRA2 | -1.3344 | 3.665758 | 3.94E-09 | 1.75E-08 |
| NEFL | 2.992434 | 7.7196 | 3.96E-09 | 1.76E-08 |
| RNF186 | -2.18826 | 2.932776 | 3.97E-09 | 1.77E-08 |
| DFNB59 | 1.467449 | 2.175831 | 3.99E-09 | 1.77E-08 |
| MCEMP1 | 2.513652 | -0.85071 | 3.99E-09 | 1.78E-08 |
| MOXD1 | -1.38101 | 4.180114 | 4.02E-09 | 1.79E-08 |
| FAM196A | -1.39488 | -1.91349 | 4.11E-09 | 1.82E-08 |
| FAM72C | 2.472063 | -3.07808 | 4.14E-09 | 1.83E-08 |
| RGCC | -1.15783 | 3.439963 | 4.15E-09 | 1.84E-08 |
| CREG2 | 2.137108 | 0.529921 | 4.17E-09 | 1.85E-08 |
| EIF4A1 | 1.179731 | 2.921488 | 4.19E-09 | 1.86E-08 |
| CHRNB2 | 1.596797 | 0.583105 | 4.24E-09 | 1.88E-08 |
| CD22 | 1.705309 | 3.430785 | 4.25E-09 | 1.88E-08 |
| RNASE1 | -1.35091 | 5.944692 | 4.27E-09 | 1.89E-08 |
| AR | 1.36079 | 6.228725 | 4.29E-09 | 1.90E-08 |
| ATP6AP1L | 1.053518 | 3.042792 | 4.31E-09 | 1.90E-08 |
| RDH5 | 1.547751 | 3.510593 | 4.37E-09 | 1.93E-08 |
| ACKR2 | -1.04622 | 1.025162 | 4.38E-09 | 1.94E-08 |
| DHDH | -1.39569 | 3.04978 | 4.38E-09 | 1.94E-08 |
| FGD5 | -1.27444 | 4.77772 | 4.44E-09 | 1.96E-08 |
| PLXNC1 | 1.011562 | 4.378954 | 4.49E-09 | 1.98E-08 |
| TAS2R4 | 1.818154 | -0.1169 | 4.52E-09 | 2.00E-08 |
| TK1 | 1.337748 | 5.099562 | 4.57E-09 | 2.02E-08 |
| TAS2R5 | 1.794827 | -0.28406 | 4.57E-09 | 2.02E-08 |
| STOX1 | -1.71971 | 0.957334 | 4.63E-09 | 2.04E-08 |
| PDF | 1.344195 | 3.140368 | 4.65E-09 | 2.05E-08 |
| GLRB | 1.368668 | 4.296597 | 4.66E-09 | 2.06E-08 |
| IL1A | 2.653786 | -1.49791 | 4.68E-09 | 2.06E-08 |
| CD300LB | 1.767475 | 0.846883 | 4.74E-09 | 2.09E-08 |
| WDR27 | 1.217601 | 4.237578 | 4.81E-09 | 2.12E-08 |
| HIST1H3G | 2.080303 | -1.78095 | 4.85E-09 | 2.13E-08 |
| CLDN23 | 1.012964 | 4.11869 | 4.88E-09 | 2.15E-08 |
| NXPE2 | -1.81004 | -3.90139 | 5.00E-09 | 2.20E-08 |
| ISM2 | -1.80148 | -0.09613 | 5.07E-09 | 2.23E-08 |
| LRRTM4 | 4.462094 | 0.987222 | 5.11E-09 | 2.24E-08 |
| MEX3A | 1.296827 | 3.202818 | 5.13E-09 | 2.25E-08 |
| PVRIG | 2.026771 | -1.03491 | 5.18E-09 | 2.27E-08 |
| THSD7B | -2.0476 | 0.305767 | 5.22E-09 | 2.29E-08 |
| CAMP | 3.21403 | -1.06373 | 5.22E-09 | 2.29E-08 |
| DCLK1 | 2.354401 | 4.570391 | 5.24E-09 | 2.30E-08 |
| OLIG1 | -2.17691 | 0.430757 | 5.25E-09 | 2.30E-08 |
| PCDHB9 | 1.588045 | 2.045576 | 5.36E-09 | 2.35E-08 |
| RAB9B | 1.173163 | 1.302697 | 5.38E-09 | 2.35E-08 |
| CCL8 | -1.667 | -0.10312 | 5.39E-09 | 2.36E-08 |
| ANXA8L1 | 4.205297 | -0.83176 | 5.56E-09 | 2.43E-08 |
| IRF6 | -1.08908 | 5.240795 | 5.58E-09 | 2.44E-08 |
| TINCR | -1.87421 | -0.13151 | 5.63E-09 | 2.46E-08 |
| KRT32 | 3.306637 | -3.22829 | 5.66E-09 | 2.47E-08 |
| GAREM2 | 1.456786 | 3.317268 | 5.68E-09 | 2.48E-08 |
| KRT222 | 2.420286 | 1.176679 | 5.73E-09 | 2.50E-08 |
| TRPV4 | -1.19729 | 4.734354 | 5.74E-09 | 2.50E-08 |
| SLC29A2 | -1.0178 | 3.627287 | 5.78E-09 | 2.52E-08 |
| NPIPB3 | 1.655369 | 0.630837 | 5.85E-09 | 2.55E-08 |
| HOXB13 | 5.064268 | 1.761446 | 5.91E-09 | 2.57E-08 |
| APLN | -1.38339 | 2.907103 | 5.92E-09 | 2.58E-08 |
| GPT2 | -1.05642 | 5.050523 | 5.93E-09 | 2.58E-08 |
| CFAP206 | 1.007176 | -0.89722 | 5.95E-09 | 2.59E-08 |
| RHCG | -2.96156 | 5.39326 | 5.99E-09 | 2.60E-08 |
| OGN | -2.41953 | 1.050326 | 6.17E-09 | 2.67E-08 |
| 3-Sep | 1.992996 | 2.221419 | 6.35E-09 | 2.75E-08 |
| ADAM8 | 1.409932 | 3.51734 | 6.49E-09 | 2.81E-08 |
| SIGLEC1 | 2.200864 | 4.101599 | 6.54E-09 | 2.83E-08 |
| LY96 | 1.997815 | 2.532018 | 6.56E-09 | 2.84E-08 |
| CKS2 | 1.180161 | 3.618159 | 6.66E-09 | 2.88E-08 |
| KCNN4 | 2.380873 | 2.504872 | 6.67E-09 | 2.88E-08 |
| AGAP6 | 1.334674 | 2.743356 | 6.69E-09 | 2.89E-08 |
| KRT25 | 4.218313 | -2.44199 | 6.81E-09 | 2.94E-08 |
| OTOG | 2.352675 | -0.53058 | 6.87E-09 | 2.96E-08 |
| PFKFB4 | 1.287867 | 3.396899 | 6.89E-09 | 2.97E-08 |
| ZDHHC11 | 1.578837 | 2.17812 | 6.93E-09 | 2.99E-08 |
| SYNGR4 | 1.816767 | -2.62874 | 6.96E-09 | 3.00E-08 |
| ASCL5 | 1.717064 | -2.06285 | 7.06E-09 | 3.04E-08 |
| GPS2 | 1.253531 | 2.844264 | 7.17E-09 | 3.08E-08 |
| HSPB3 | -2.57956 | -3.56298 | 7.18E-09 | 3.09E-08 |
| MACC1 | 1.483152 | 7.172545 | 7.22E-09 | 3.10E-08 |
| CGREF1 | 1.895743 | 3.694295 | 7.40E-09 | 3.18E-08 |
| CYP4F3 | -2.03 | 4.154152 | 7.45E-09 | 3.20E-08 |
| GYPE | -1.21739 | -2.38548 | 7.45E-09 | 3.20E-08 |
| 1-Mar | 1.13998 | 3.222465 | 7.47E-09 | 3.21E-08 |
| VWA2 | -1.54578 | 0.526513 | 7.49E-09 | 3.21E-08 |
| CDK3 | 1.515253 | -0.40061 | 7.49E-09 | 3.21E-08 |
| ASIC4 | 2.61083 | -2.07561 | 7.50E-09 | 3.21E-08 |
| NEBL | -1.3585 | 5.81027 | 7.50E-09 | 3.21E-08 |
| NR1I2 | 1.453106 | -0.63874 | 7.86E-09 | 3.36E-08 |
| INSL3 | 1.821481 | -1.11014 | 7.87E-09 | 3.36E-08 |
| AC010422.3 | 1.115045 | -1.17018 | 7.93E-09 | 3.39E-08 |
| GABRG1 | -2.55297 | -3.27999 | 7.94E-09 | 3.39E-08 |
| AP1M2 | -1.10794 | 5.464561 | 8.13E-09 | 3.47E-08 |
| ADAMTS8 | -1.21013 | 0.822414 | 8.17E-09 | 3.49E-08 |
| FCGR2A | 1.315269 | 4.720531 | 8.43E-09 | 3.59E-08 |
| LRRC39 | 1.685071 | 0.627293 | 8.46E-09 | 3.60E-08 |
| FKBP6 | 2.200041 | -3.07823 | 8.52E-09 | 3.63E-08 |
| ABCB4 | 1.688542 | 2.311798 | 8.64E-09 | 3.67E-08 |
| HSPG2 | -1.21842 | 7.292618 | 8.64E-09 | 3.67E-08 |
| CLIC6 | 2.616615 | 5.721249 | 8.78E-09 | 3.73E-08 |
| CD248 | -1.31918 | 3.318091 | 8.91E-09 | 3.78E-08 |
| ZNF337 | 1.432986 | 2.246731 | 9.04E-09 | 3.84E-08 |
| PKLR | -1.99725 | 4.286731 | 9.10E-09 | 3.86E-08 |
| HSPA6 | 2.497138 | 3.496161 | 9.18E-09 | 3.89E-08 |
| TSHZ3 | -1.05552 | 1.869101 | 9.35E-09 | 3.96E-08 |
| RAB37 | 1.132858 | 3.201534 | 9.37E-09 | 3.97E-08 |
| GGTLC2 | 2.070442 | -0.46527 | 9.47E-09 | 4.01E-08 |
| TIMP4 | -1.38266 | -0.13675 | 9.55E-09 | 4.04E-08 |
| LPAR6 | 1.255669 | 5.21186 | 9.75E-09 | 4.12E-08 |
| SNAI3 | 1.094595 | 0.948535 | 1.00E-08 | 4.23E-08 |
| KLK4 | 6.61858 | 4.323347 | 1.02E-08 | 4.29E-08 |
| CCL28 | 2.119277 | 5.068832 | 1.04E-08 | 4.36E-08 |
| NKPD1 | 1.964104 | -1.98597 | 1.05E-08 | 4.41E-08 |
| BNC1 | 2.883099 | 2.345653 | 1.05E-08 | 4.42E-08 |
| ITGB4 | 1.407892 | 6.999863 | 1.07E-08 | 4.52E-08 |
| LEMD1 | 2.512239 | 0.750843 | 1.08E-08 | 4.53E-08 |
| SOX5 | -1.72262 | 0.675764 | 1.08E-08 | 4.55E-08 |
| SLC12A8 | 1.24448 | 4.587726 | 1.08E-08 | 4.55E-08 |
| TNN | -1.63395 | -1.252 | 1.08E-08 | 4.56E-08 |
| KIF15 | 1.398097 | 0.599467 | 1.11E-08 | 4.66E-08 |
| SLC34A2 | 2.713548 | 8.884989 | 1.12E-08 | 4.69E-08 |
| TUBA8 | 1.391078 | -1.32714 | 1.12E-08 | 4.71E-08 |
| CLEC12B | 2.524179 | -2.71771 | 1.13E-08 | 4.75E-08 |
| TEKT3 | 1.85565 | -0.72433 | 1.14E-08 | 4.79E-08 |
| LHX2 | 3.257669 | -2.45601 | 1.15E-08 | 4.81E-08 |
| HOXB6 | -1.24437 | 4.423996 | 1.15E-08 | 4.83E-08 |
| CD300LG | -1.83239 | 0.714409 | 1.18E-08 | 4.94E-08 |
| WDR62 | 1.100667 | 1.461168 | 1.21E-08 | 5.04E-08 |
| DPP6 | -2.45037 | 2.223497 | 1.21E-08 | 5.05E-08 |
| NYAP1 | -1.28948 | 0.022843 | 1.23E-08 | 5.12E-08 |
| TSPAN10 | 2.355708 | 3.759461 | 1.24E-08 | 5.17E-08 |
| GNRH1 | 1.668589 | 0.822019 | 1.24E-08 | 5.17E-08 |
| ZNF385B | 1.600569 | 5.628587 | 1.24E-08 | 5.18E-08 |
| FAM193B | 1.148684 | 5.491702 | 1.26E-08 | 5.25E-08 |
| HHIPL2 | 4.768055 | 0.206863 | 1.27E-08 | 5.27E-08 |
| SDC3 | 1.022918 | 6.811192 | 1.28E-08 | 5.30E-08 |
| HPX | 1.832271 | -0.20939 | 1.29E-08 | 5.37E-08 |
| YPEL4 | 1.47385 | 0.008933 | 1.31E-08 | 5.42E-08 |
| SCN9A | -1.86567 | 1.171236 | 1.31E-08 | 5.42E-08 |
| PRC1 | 1.178834 | 3.054982 | 1.31E-08 | 5.43E-08 |
| EVI2A | 1.341317 | 2.600493 | 1.32E-08 | 5.46E-08 |
| GDPD2 | -1.97579 | -0.44763 | 1.32E-08 | 5.46E-08 |
| ALDH1A1 | 1.405245 | 9.997966 | 1.32E-08 | 5.47E-08 |
| INPP5D | 1.19653 | 5.155928 | 1.32E-08 | 5.48E-08 |
| PAPLN | 1.181281 | 7.510532 | 1.33E-08 | 5.53E-08 |
| CORO1A | 1.350607 | 5.426504 | 1.34E-08 | 5.54E-08 |
| C1QTNF3 | 2.659663 | 5.717013 | 1.36E-08 | 5.63E-08 |
| MAGEE2 | -1.59214 | -2.8306 | 1.36E-08 | 5.63E-08 |
| UPK3A | 4.202042 | 0.384361 | 1.41E-08 | 5.80E-08 |
| CYSLTR2 | 3.298785 | 5.544418 | 1.43E-08 | 5.89E-08 |
| TRPC3 | 3.079941 | 1.16907 | 1.43E-08 | 5.92E-08 |
| MS4A4A | 1.879598 | 3.915484 | 1.45E-08 | 5.98E-08 |
| CAMK2N2 | 2.446271 | -0.25318 | 1.45E-08 | 5.99E-08 |
| DNAH10 | -1.14254 | 0.988932 | 1.46E-08 | 6.02E-08 |
| CLNK | -2.26672 | 1.76839 | 1.47E-08 | 6.06E-08 |
| FBLN1 | -1.53455 | 5.50015 | 1.47E-08 | 6.07E-08 |
| ZP1 | 3.845893 | -2.19096 | 1.49E-08 | 6.14E-08 |
| GRM3 | -1.80054 | -2.64134 | 1.51E-08 | 6.20E-08 |
| CRP | 7.045509 | 2.743725 | 1.52E-08 | 6.24E-08 |
| HHLA2 | 2.515866 | 5.640371 | 1.52E-08 | 6.27E-08 |
| PLCH2 | -1.50147 | 3.714451 | 1.57E-08 | 6.44E-08 |
| LINC00672 | 1.603992 | 0.46138 | 1.58E-08 | 6.48E-08 |
| S100A6 | 1.139619 | 10.14235 | 1.58E-08 | 6.51E-08 |
| SHISA8 | -1.90941 | -3.0767 | 1.59E-08 | 6.55E-08 |
| NPY5R | -1.50895 | 0.590663 | 1.60E-08 | 6.55E-08 |
| KCNH6 | -1.63513 | 3.021959 | 1.62E-08 | 6.63E-08 |
| CELF5 | 2.589427 | 1.080635 | 1.63E-08 | 6.69E-08 |
| FNDC4 | 1.986943 | 4.302167 | 1.64E-08 | 6.71E-08 |
| APOBEC3G | 1.124274 | 3.481791 | 1.64E-08 | 6.71E-08 |
| DDX47 | 1.035973 | 1.104014 | 1.69E-08 | 6.91E-08 |
| SMOC2 | -1.45147 | 3.467665 | 1.70E-08 | 6.93E-08 |
| PROKR2 | -2.60216 | -4.08369 | 1.70E-08 | 6.96E-08 |
| RTEL1-TNFRSF6B | 1.316599 | 1.822767 | 1.71E-08 | 6.97E-08 |
| RGL4 | 1.430859 | 0.900616 | 1.72E-08 | 7.01E-08 |
| ST18 | -1.14562 | -1.55904 | 1.73E-08 | 7.05E-08 |
| HS3ST4 | 5.155718 | -0.77334 | 1.74E-08 | 7.09E-08 |
| CATSPERG | 2.912775 | 3.642683 | 1.75E-08 | 7.15E-08 |
| CATSPER2 | 1.176484 | 2.199057 | 1.77E-08 | 7.19E-08 |
| CALCR | -1.8025 | -0.06643 | 1.80E-08 | 7.32E-08 |
| LYZL1 | 3.044032 | -3.28888 | 1.82E-08 | 7.39E-08 |
| LANCL3 | -1.63869 | -1.50408 | 1.82E-08 | 7.41E-08 |
| EGLN3 | 1.740924 | 5.967849 | 1.83E-08 | 7.44E-08 |
| BCL2L10 | -1.24512 | 0.719233 | 1.84E-08 | 7.47E-08 |
| IFNA1 | 2.363646 | -3.6456 | 1.87E-08 | 7.61E-08 |
| PLEK | 1.557161 | 4.313747 | 1.90E-08 | 7.70E-08 |
| OTOF | 2.360923 | -1.5209 | 1.90E-08 | 7.72E-08 |
| KRT17 | 3.407379 | 5.229792 | 1.92E-08 | 7.78E-08 |
| USP44 | -1.29962 | 0.375058 | 1.92E-08 | 7.79E-08 |
| PLTP | 1.536206 | 7.630907 | 1.94E-08 | 7.85E-08 |
| MUC5B | 6.527014 | 4.534076 | 1.95E-08 | 7.90E-08 |
| TBC1D3B | 2.322592 | -2.38844 | 1.95E-08 | 7.90E-08 |
| MFSD2B | 1.161244 | -1.54317 | 1.95E-08 | 7.90E-08 |
| GPSM3 | 1.310029 | 4.415249 | 1.96E-08 | 7.94E-08 |
| GPR143 | 2.566844 | 5.417452 | 1.97E-08 | 7.96E-08 |
| KCNK7 | 1.136375 | -0.96383 | 1.97E-08 | 7.97E-08 |
| RIN3 | 1.052278 | 5.095899 | 1.97E-08 | 7.97E-08 |
| ACTG2 | -1.98764 | 2.723366 | 1.98E-08 | 7.99E-08 |
| AC008687.4 | 2.317288 | -2.17982 | 1.98E-08 | 8.01E-08 |
| DDIT3 | 1.04192 | 5.440403 | 1.99E-08 | 8.05E-08 |
| CLEC4E | 1.780292 | 2.158583 | 2.01E-08 | 8.12E-08 |
| CA6 | 2.321754 | -3.51769 | 2.01E-08 | 8.12E-08 |
| ZNF474 | 1.694862 | -1.14491 | 2.03E-08 | 8.20E-08 |
| RAD21L1 | 2.98244 | -2.99567 | 2.06E-08 | 8.30E-08 |
| PALM3 | -1.29428 | 4.331907 | 2.06E-08 | 8.31E-08 |
| SENP3-EIF4A1 | 2.134052 | -2.37658 | 2.10E-08 | 8.44E-08 |
| SLC17A7 | -1.20768 | -1.40434 | 2.12E-08 | 8.54E-08 |
| CRYGS | 1.4732 | 1.294571 | 2.12E-08 | 8.55E-08 |
| GRIN1 | 1.876349 | -1.76327 | 2.14E-08 | 8.60E-08 |
| CALCRL | -1.04501 | 4.251783 | 2.14E-08 | 8.61E-08 |
| MFGE8 | -1.14393 | 5.119373 | 2.14E-08 | 8.62E-08 |
| ARHGAP40 | 2.748718 | 3.833833 | 2.17E-08 | 8.70E-08 |
| MYH3 | 1.424129 | 2.354308 | 2.17E-08 | 8.71E-08 |
| HECW2 | -1.5533 | 2.355242 | 2.20E-08 | 8.83E-08 |
| JSRP1 | 2.481503 | 1.256189 | 2.24E-08 | 8.98E-08 |
| CPEB1 | 1.870233 | 2.563044 | 2.25E-08 | 9.03E-08 |
| FOS | -1.51475 | 7.800159 | 2.27E-08 | 9.10E-08 |
| TALDO1 | 1.146544 | 8.020462 | 2.30E-08 | 9.20E-08 |
| EN1 | 5.167728 | -0.19241 | 2.33E-08 | 9.33E-08 |
| CD53 | 1.371524 | 5.185159 | 2.34E-08 | 9.34E-08 |
| EDDM3A | -1.91306 | -1.96622 | 2.35E-08 | 9.39E-08 |
| AC106782.1 | 1.719324 | -0.76288 | 2.37E-08 | 9.45E-08 |
| NPIPB4 | 1.48247 | 1.255546 | 2.37E-08 | 9.46E-08 |
| NXN | 1.061043 | 5.274482 | 2.40E-08 | 9.60E-08 |
| ROBO2 | -1.44718 | 1.442481 | 2.41E-08 | 9.61E-08 |
| SGO1 | 1.450717 | -0.1897 | 2.42E-08 | 9.66E-08 |
| FOXI2 | -2.71234 | 0.673945 | 2.45E-08 | 9.78E-08 |
| TNFSF10 | 1.2931 | 8.644023 | 2.50E-08 | 9.98E-08 |
| UPK2 | 5.02252 | 2.233093 | 2.51E-08 | 1.00E-07 |
| RETREG1 | 1.054743 | 7.162325 | 2.51E-08 | 1.00E-07 |
| HHATL | 7.1196 | 4.676271 | 2.55E-08 | 1.02E-07 |
| CDH2 | 1.337185 | 6.644135 | 2.57E-08 | 1.02E-07 |
| SLC30A2 | -1.81875 | 3.851678 | 2.58E-08 | 1.03E-07 |
| CCNB1 | 1.031254 | 3.310876 | 2.59E-08 | 1.03E-07 |
| KLRK1 | 1.949976 | -1.49906 | 2.64E-08 | 1.05E-07 |
| SH3TC2 | 2.195223 | 0.838375 | 2.64E-08 | 1.05E-07 |
| BMP6 | -1.41581 | 3.521964 | 2.65E-08 | 1.05E-07 |
| TRIM6 | -1.03115 | 3.790696 | 2.67E-08 | 1.06E-07 |
| AL121594.3 | 1.569046 | -1.82746 | 2.69E-08 | 1.07E-07 |
| C4BPA | 3.67827 | 1.254102 | 2.70E-08 | 1.07E-07 |
| PRSS23 | -1.12939 | 5.74798 | 2.71E-08 | 1.07E-07 |
| DLGAP3 | -1.27557 | 0.115497 | 2.91E-08 | 1.15E-07 |
| VNN3 | 2.302607 | -1.59333 | 2.93E-08 | 1.16E-07 |
| UBASH3B | 1.375734 | 3.723052 | 2.94E-08 | 1.16E-07 |
| AGER | 1.30979 | 2.712053 | 2.94E-08 | 1.16E-07 |
| GINS4 | 1.001169 | 1.57771 | 2.99E-08 | 1.18E-07 |
| MYO15B | 1.258227 | 7.581352 | 3.04E-08 | 1.20E-07 |
| HAUS7 | 1.479387 | 2.479093 | 3.06E-08 | 1.21E-07 |
| TERT | 6.200348 | -0.95562 | 3.08E-08 | 1.22E-07 |
| SMCO2 | 1.716919 | -1.86461 | 3.08E-08 | 1.22E-07 |
| ACSL1 | -1.02027 | 7.768633 | 3.08E-08 | 1.22E-07 |
| GAS6 | -1.00923 | 6.787966 | 3.10E-08 | 1.22E-07 |
| KCNN1 | 2.56371 | 0.471286 | 3.12E-08 | 1.23E-07 |
| HOXC13 | 3.479678 | -0.85482 | 3.13E-08 | 1.23E-07 |
| MEF2B | 1.244536 | -0.78887 | 3.13E-08 | 1.23E-07 |
| SNX20 | 1.449746 | 2.464852 | 3.17E-08 | 1.25E-07 |
| SPAG6 | 3.379447 | 3.080428 | 3.28E-08 | 1.29E-07 |
| RAB26 | 1.361451 | 1.313306 | 3.29E-08 | 1.29E-07 |
| SLA | 1.379615 | 4.388222 | 3.29E-08 | 1.29E-07 |
| DLGAP1 | 2.078524 | 4.346111 | 3.30E-08 | 1.30E-07 |
| MAP1LC3C | 2.738157 | 1.678718 | 3.31E-08 | 1.30E-07 |
| MADCAM1 | 1.340114 | -0.18771 | 3.35E-08 | 1.31E-07 |
| PTGIR | -1.16844 | 1.25567 | 3.35E-08 | 1.31E-07 |
| PPM1N | 1.345429 | 0.018728 | 3.38E-08 | 1.33E-07 |
| HCST | 1.487789 | 2.200451 | 3.41E-08 | 1.33E-07 |
| RHOU | 1.340822 | 5.681932 | 3.43E-08 | 1.34E-07 |
| AC010326.2 | -1.08415 | -3.70584 | 3.48E-08 | 1.36E-07 |
| C9orf50 | 1.874059 | 0.805638 | 3.51E-08 | 1.37E-07 |
| STRA8 | 2.055056 | 0.211422 | 3.53E-08 | 1.38E-07 |
| LCAT | 1.276666 | 3.74674 | 3.56E-08 | 1.39E-07 |
| TYR | 2.943573 | -2.31981 | 3.59E-08 | 1.40E-07 |
| CACNA2D4 | 1.101898 | 2.030212 | 3.60E-08 | 1.40E-07 |
| ALAS2 | 2.808034 | 0.899479 | 3.62E-08 | 1.41E-07 |
| STRC | 2.09511 | -1.90553 | 3.72E-08 | 1.45E-07 |
| RASGEF1B | 1.056422 | 4.647553 | 3.73E-08 | 1.45E-07 |
| CTSK | 2.956545 | 6.320271 | 3.77E-08 | 1.47E-07 |
| ELOVL2 | 2.029414 | 0.770953 | 3.82E-08 | 1.48E-07 |
| TMC8 | 1.313028 | 3.653724 | 3.88E-08 | 1.51E-07 |
| CKMT2 | -1.87781 | 2.627011 | 3.91E-08 | 1.52E-07 |
| NPEPL1 | 1.024774 | 4.816454 | 3.96E-08 | 1.54E-07 |
| GALNT3 | -1.30814 | 3.608806 | 3.97E-08 | 1.54E-07 |
| KCTD15 | -1.29075 | 3.449038 | 3.97E-08 | 1.54E-07 |
| SCART1 | 1.539644 | 1.875605 | 4.00E-08 | 1.55E-07 |
| DAW1 | 2.015223 | 0.819128 | 4.03E-08 | 1.56E-07 |
| EVI2B | 1.35145 | 3.669009 | 4.04E-08 | 1.57E-07 |
| PCDH17 | -1.51104 | 1.944289 | 4.06E-08 | 1.57E-07 |
| OPRL1 | 1.050294 | 1.539696 | 4.15E-08 | 1.60E-07 |
| ADAM28 | 1.282631 | 4.215109 | 4.20E-08 | 1.62E-07 |
| UROC1 | 2.292071 | -1.77356 | 4.21E-08 | 1.62E-07 |
| GRIA3 | -1.71463 | 0.125874 | 4.26E-08 | 1.64E-07 |
| TMEM256-PLSCR3 | 1.166748 | -2.49226 | 4.28E-08 | 1.65E-07 |
| SPAG8 | 1.201778 | 1.846288 | 4.29E-08 | 1.65E-07 |
| PLS1 | 1.059954 | 6.533031 | 4.29E-08 | 1.65E-07 |
| HIST1H2AM | 2.023703 | -2.94972 | 4.41E-08 | 1.69E-07 |
| NR0B1 | 5.90271 | 1.332287 | 4.46E-08 | 1.71E-07 |
| CNTFR | -2.03132 | 0.208642 | 4.47E-08 | 1.72E-07 |
| RARRES2 | 1.567329 | 7.998911 | 4.52E-08 | 1.73E-07 |
| TMEM196 | 4.304161 | -0.49165 | 4.56E-08 | 1.75E-07 |
| PLA1A | 1.803508 | 5.403112 | 4.61E-08 | 1.77E-07 |
| ZNF98 | -1.91024 | -1.1453 | 4.62E-08 | 1.77E-07 |
| DMTF1 | 1.106005 | 5.947028 | 4.63E-08 | 1.77E-07 |
| PERM1 | 1.746012 | 2.433066 | 4.65E-08 | 1.78E-07 |
| ADAMTS3 | 1.374777 | 3.063933 | 4.67E-08 | 1.79E-07 |
| ZG16 | -2.0048 | -3.92839 | 4.77E-08 | 1.83E-07 |
| ST3GAL6 | -1.04807 | 3.592294 | 4.84E-08 | 1.85E-07 |
| HMGA1 | 1.11099 | 6.619412 | 4.99E-08 | 1.90E-07 |
| ITM2A | -1.33288 | 3.492553 | 5.01E-08 | 1.91E-07 |
| AKR1B15 | 5.413339 | 1.748669 | 5.01E-08 | 1.91E-07 |
| COL6A6 | -1.11575 | -2.04217 | 5.10E-08 | 1.94E-07 |
| RGN | -1.10118 | 4.756294 | 5.12E-08 | 1.95E-07 |
| ST8SIA3 | 4.754399 | -2.28824 | 5.23E-08 | 1.99E-07 |
| C17orf64 | 2.044646 | -1.77981 | 5.24E-08 | 1.99E-07 |
| RARRES1 | 2.519146 | 5.208889 | 5.27E-08 | 2.01E-07 |
| ST8SIA5 | -1.80501 | 0.465267 | 5.32E-08 | 2.02E-07 |
| BMP3 | -2.00336 | -1.34495 | 5.36E-08 | 2.04E-07 |
| KRT15 | 3.340881 | 1.911233 | 5.38E-08 | 2.04E-07 |
| RET | -1.37809 | 0.129629 | 5.38E-08 | 2.04E-07 |
| NPIPB11 | 2.080577 | 0.632445 | 5.39E-08 | 2.04E-07 |
| NAALADL2 | -1.03563 | 3.350815 | 5.41E-08 | 2.05E-07 |
| IGFL2 | 2.891752 | -2.47321 | 5.45E-08 | 2.07E-07 |
| SLC27A2 | -1.31857 | 5.839621 | 5.45E-08 | 2.07E-07 |
| NYNRIN | -1.12573 | 4.560213 | 5.51E-08 | 2.09E-07 |
| ABCB5 | 6.535067 | 3.627384 | 5.55E-08 | 2.10E-07 |
| CNTNAP1 | 1.271658 | 3.461588 | 5.59E-08 | 2.12E-07 |
| NAT14 | 1.099497 | 5.244278 | 5.75E-08 | 2.17E-07 |
| HILPDA | 1.859926 | 4.609137 | 5.90E-08 | 2.23E-07 |
| C2orf91 | 1.686542 | -1.96626 | 5.92E-08 | 2.24E-07 |
| KRT23 | 3.481242 | 3.837917 | 5.93E-08 | 2.24E-07 |
| PTGFR | -1.69762 | 2.148485 | 5.93E-08 | 2.24E-07 |
| EPS8L3 | 3.001357 | 1.293731 | 5.95E-08 | 2.25E-07 |
| LRRTM3 | -2.02137 | -4.10082 | 6.01E-08 | 2.27E-07 |
| TMEM151A | 2.252032 | -0.07129 | 6.05E-08 | 2.28E-07 |
| CPT1B | 1.395805 | 1.980388 | 6.06E-08 | 2.28E-07 |
| PDE1C | -1.86607 | 2.097356 | 6.18E-08 | 2.33E-07 |
| TRPC4 | -1.21785 | -0.60619 | 6.23E-08 | 2.34E-07 |
| CYP3A7 | -1.33977 | 1.31845 | 6.23E-08 | 2.34E-07 |
| CD80 | 1.553851 | -1.52449 | 6.29E-08 | 2.36E-07 |
| S100Z | 1.410724 | -2.04764 | 6.36E-08 | 2.39E-07 |
| COL17A1 | 3.469148 | 4.017602 | 6.62E-08 | 2.48E-07 |
| ATF3 | -1.47006 | 6.142461 | 6.66E-08 | 2.49E-07 |
| GYPC | 1.139216 | 5.993786 | 6.66E-08 | 2.49E-07 |
| CCL17 | 1.881868 | -0.12427 | 6.70E-08 | 2.51E-07 |
| RORB | -2.05072 | -0.07446 | 6.71E-08 | 2.51E-07 |
| RASL10B | -1.26244 | 1.204887 | 6.83E-08 | 2.55E-07 |
| SST | 5.776115 | 5.813649 | 6.88E-08 | 2.57E-07 |
| TENM1 | 1.71844 | 3.910529 | 6.89E-08 | 2.57E-07 |
| STX19 | -1.43858 | -2.81932 | 6.90E-08 | 2.58E-07 |
| FOXI1 | -3.56092 | 3.268987 | 6.96E-08 | 2.60E-07 |
| KIT | -1.56414 | 4.263661 | 7.01E-08 | 2.61E-07 |
| CACNB4 | -1.42028 | 1.625039 | 7.15E-08 | 2.67E-07 |
| LSMEM1 | 1.290625 | 1.113968 | 7.21E-08 | 2.69E-07 |
| CADM3 | 3.475371 | 4.626578 | 7.26E-08 | 2.70E-07 |
| REG3G | 4.233662 | 3.251612 | 7.32E-08 | 2.72E-07 |
| AZGP1 | -1.77643 | 6.102652 | 7.34E-08 | 2.73E-07 |
| NOXA1 | 1.014709 | 4.847269 | 7.40E-08 | 2.75E-07 |
| ABI3BP | 1.809621 | 7.188135 | 7.42E-08 | 2.76E-07 |
| FBLN2 | -1.32515 | 4.376964 | 7.55E-08 | 2.81E-07 |
| LILRB2 | 1.354637 | 2.871824 | 7.56E-08 | 2.81E-07 |
| SYT16 | 1.931582 | 2.764726 | 7.56E-08 | 2.81E-07 |
| ADGRG7 | 6.435317 | -0.12321 | 7.73E-08 | 2.87E-07 |
| CPLX2 | 7.734436 | 2.540032 | 7.74E-08 | 2.87E-07 |
| BMP8B | 1.763843 | 3.276153 | 7.75E-08 | 2.87E-07 |
| LFNG | 1.654614 | 5.175365 | 7.76E-08 | 2.88E-07 |
| LRRC25 | 1.426581 | 3.287713 | 7.79E-08 | 2.89E-07 |
| ACOT1 | -1.1056 | 2.766109 | 7.80E-08 | 2.89E-07 |
| SBK1 | 1.444972 | 4.315005 | 7.80E-08 | 2.89E-07 |
| CTSD | 1.098083 | 11.50167 | 7.94E-08 | 2.94E-07 |
| PLA2G12B | -2.27424 | 1.243867 | 7.95E-08 | 2.94E-07 |
| ATAD3B | 1.030932 | 4.223627 | 7.99E-08 | 2.95E-07 |
| L3MBTL1 | 1.161356 | 3.534606 | 8.05E-08 | 2.98E-07 |
| PRRG3 | -1.67568 | -1.14473 | 8.16E-08 | 3.01E-07 |
| FOXD3 | -2.41782 | -4.05051 | 8.29E-08 | 3.06E-07 |
| ZNF334 | 1.412501 | 4.006793 | 8.32E-08 | 3.07E-07 |
| EFHB | 1.246953 | 2.024675 | 8.34E-08 | 3.08E-07 |
| UPK3B | 2.515377 | 1.473522 | 8.67E-08 | 3.19E-07 |
| ATP1A3 | 3.064558 | 1.860735 | 8.79E-08 | 3.23E-07 |
| PIGR | 1.976931 | 11.27608 | 8.80E-08 | 3.23E-07 |
| ANKRD18A | 1.269541 | 1.252517 | 8.81E-08 | 3.23E-07 |
| FAM19A4 | -1.87979 | -3.08395 | 8.93E-08 | 3.28E-07 |
| FOXD4 | 1.392651 | 0.154652 | 9.24E-08 | 3.38E-07 |
| LINGO3 | 1.635251 | -1.38193 | 9.34E-08 | 3.41E-07 |
| FOXN4 | -1.7166 | -2.00542 | 9.39E-08 | 3.43E-07 |
| MIP | 1.483757 | -2.52855 | 9.43E-08 | 3.44E-07 |
| MEGF10 | -1.81637 | -2.37781 | 9.45E-08 | 3.45E-07 |
| NKG7 | 2.094347 | 3.211724 | 9.46E-08 | 3.45E-07 |
| PRR29 | 1.17119 | 2.815406 | 9.48E-08 | 3.46E-07 |
| ATP2B2 | 2.751735 | 2.914292 | 9.65E-08 | 3.52E-07 |
| RNASE6 | 1.337205 | 3.576745 | 9.65E-08 | 3.52E-07 |
| AL139260.3 | 1.204346 | -2.33861 | 9.67E-08 | 3.52E-07 |
| JAML | 1.526285 | 3.879708 | 9.80E-08 | 3.57E-07 |
| PNMT | -1.97437 | -1.13304 | 9.88E-08 | 3.59E-07 |
| ENTPD5 | -1.05539 | 6.247096 | 9.88E-08 | 3.59E-07 |
| GPR19 | 1.872974 | -1.45626 | 1.01E-07 | 3.68E-07 |
| ARMC3 | 1.832484 | 1.991207 | 1.04E-07 | 3.79E-07 |
| CD52 | 1.607987 | 3.979415 | 1.05E-07 | 3.80E-07 |
| PILRB | 1.741155 | 3.57639 | 1.05E-07 | 3.80E-07 |
| RASSF2 | 1.071703 | 4.300773 | 1.07E-07 | 3.86E-07 |
| FGF5 | -2.50185 | -1.59447 | 1.07E-07 | 3.88E-07 |
| NAV3 | 1.624137 | 1.991288 | 1.08E-07 | 3.92E-07 |
| ZNF300 | 1.156083 | 3.514285 | 1.08E-07 | 3.92E-07 |
| LYZ | 1.945272 | 6.597998 | 1.09E-07 | 3.93E-07 |
| NPAP1 | -2.00267 | -1.56383 | 1.09E-07 | 3.94E-07 |
| CXCL8 | 2.038708 | 4.116939 | 1.09E-07 | 3.95E-07 |
| NPTX2 | 2.985747 | 1.677261 | 1.10E-07 | 3.98E-07 |
| GRIN3B | 2.28148 | 1.569678 | 1.11E-07 | 4.00E-07 |
| PRSS22 | -2.04926 | 1.565441 | 1.11E-07 | 4.02E-07 |
| SYTL2 | 1.084101 | 6.976703 | 1.12E-07 | 4.06E-07 |
| FGF20 | 1.901319 | -2.17888 | 1.15E-07 | 4.16E-07 |
| LIPH | -1.12712 | 3.817794 | 1.15E-07 | 4.16E-07 |
| KRT83 | 3.239815 | -3.06928 | 1.17E-07 | 4.20E-07 |
| C9 | -1.39087 | -0.77024 | 1.17E-07 | 4.23E-07 |
| ELSPBP1 | 4.566014 | -0.90752 | 1.20E-07 | 4.33E-07 |
| SH2D2A | 1.718832 | 1.427031 | 1.20E-07 | 4.33E-07 |
| HRCT1 | 1.763912 | 3.291734 | 1.20E-07 | 4.33E-07 |
| IFITM5 | 3.923326 | -1.69149 | 1.21E-07 | 4.35E-07 |
| SLC4A4 | -1.27973 | 7.517484 | 1.21E-07 | 4.37E-07 |
| S100A9 | 2.884523 | 5.900221 | 1.22E-07 | 4.37E-07 |
| PLEKHG4B | -1.54741 | 1.856041 | 1.22E-07 | 4.39E-07 |
| AQP9 | 2.253767 | 2.258916 | 1.27E-07 | 4.55E-07 |
| UGT1A9 | -2.50044 | 5.166821 | 1.27E-07 | 4.57E-07 |
| SLC26A9 | -1.7075 | 2.883541 | 1.28E-07 | 4.59E-07 |
| TNFRSF9 | 2.1743 | 1.408164 | 1.28E-07 | 4.60E-07 |
| MUC16 | 4.253637 | 1.352747 | 1.29E-07 | 4.61E-07 |
| GCNT4 | -1.23638 | 2.756457 | 1.30E-07 | 4.66E-07 |
| SULT1A3 | 1.749976 | -3.12755 | 1.30E-07 | 4.67E-07 |
| ZNF385D | -1.62165 | 1.610026 | 1.31E-07 | 4.68E-07 |
| SLC6A7 | 2.309508 | -2.67013 | 1.32E-07 | 4.72E-07 |
| GABRR1 | -1.77215 | -3.84838 | 1.33E-07 | 4.76E-07 |
| TEDDM1 | -1.61463 | -3.50498 | 1.34E-07 | 4.77E-07 |
| MYO1G | 1.276826 | 2.889076 | 1.35E-07 | 4.81E-07 |
| APOC2 | 1.830567 | -1.13038 | 1.36E-07 | 4.84E-07 |
| MAP4K1 | 1.207334 | 2.730409 | 1.38E-07 | 4.92E-07 |
| AFP | -1.70167 | -0.67135 | 1.38E-07 | 4.94E-07 |
| TNFSF13B | 1.255676 | 2.254587 | 1.39E-07 | 4.95E-07 |
| XDH | 3.317384 | 2.566548 | 1.39E-07 | 4.96E-07 |
| RASAL3 | 1.232546 | 3.106246 | 1.39E-07 | 4.96E-07 |
| LINC00521 | 3.608015 | -3.73668 | 1.40E-07 | 4.97E-07 |
| TAL2 | -1.32594 | -1.13389 | 1.40E-07 | 4.99E-07 |
| VWA5B2 | 1.74085 | 0.655533 | 1.41E-07 | 5.01E-07 |
| ZNF23 | 1.195786 | 1.164829 | 1.44E-07 | 5.13E-07 |
| AOX1 | -1.56779 | 6.727177 | 1.45E-07 | 5.13E-07 |
| IRX5 | 1.809222 | 4.57771 | 1.45E-07 | 5.14E-07 |
| ADGRD1 | -1.13708 | 1.009555 | 1.48E-07 | 5.23E-07 |
| FGR | 1.349583 | 4.030655 | 1.48E-07 | 5.26E-07 |
| CRIP3 | 1.996065 | 2.260451 | 1.49E-07 | 5.26E-07 |
| CACNA1E | -1.62722 | 0.586327 | 1.49E-07 | 5.27E-07 |
| TM4SF4 | 3.377444 | 3.116587 | 1.52E-07 | 5.39E-07 |
| CFAP69 | 1.170396 | 3.91799 | 1.55E-07 | 5.48E-07 |
| C1orf194 | 2.304174 | -0.71506 | 1.57E-07 | 5.55E-07 |
| PITX1 | 4.352706 | 1.149522 | 1.58E-07 | 5.56E-07 |
| FKBP10 | 1.962829 | 6.464676 | 1.59E-07 | 5.62E-07 |
| ADGRG6 | 1.343281 | 6.093932 | 1.64E-07 | 5.78E-07 |
| 11-Mar | -2.40184 | -3.80227 | 1.65E-07 | 5.82E-07 |
| MS4A7 | 1.432508 | 4.420521 | 1.66E-07 | 5.83E-07 |
| CAV3 | -1.91159 | -4.04458 | 1.66E-07 | 5.85E-07 |
| ATP2C2 | -1.43414 | 1.413013 | 1.69E-07 | 5.95E-07 |
| AC004754.1 | 1.221689 | -0.02587 | 1.70E-07 | 5.97E-07 |
| EEF1G | 1.179751 | -0.13833 | 1.70E-07 | 5.97E-07 |
| TNFSF12-TNFSF13 | 1.56465 | -2.4551 | 1.71E-07 | 6.00E-07 |
| CATIP | 1.206032 | 3.281712 | 1.73E-07 | 6.06E-07 |
| GPR183 | 1.568262 | 3.509255 | 1.73E-07 | 6.09E-07 |
| PTH2R | 1.133222 | 5.215189 | 1.75E-07 | 6.13E-07 |
| RSPO1 | -2.31215 | -2.81916 | 1.76E-07 | 6.17E-07 |
| TMPRSS13 | -1.42293 | -0.22993 | 1.78E-07 | 6.23E-07 |
| SLC30A8 | -2.20883 | 0.763474 | 1.79E-07 | 6.26E-07 |
| KIF18A | 1.255371 | 0.556427 | 1.79E-07 | 6.27E-07 |
| CNTD2 | -1.6065 | 1.58042 | 1.81E-07 | 6.34E-07 |
| TXNRD1 | 1.610059 | 7.772741 | 1.84E-07 | 6.43E-07 |
| FMO3 | -1.38237 | 2.126738 | 1.86E-07 | 6.48E-07 |
| NCAN | 2.448677 | -2.33016 | 1.89E-07 | 6.60E-07 |
| CTAGE4 | 1.640691 | -1.48487 | 1.90E-07 | 6.62E-07 |
| PAGE2B | 5.968539 | -1.24961 | 1.90E-07 | 6.63E-07 |
| TLL2 | 1.574357 | 1.390459 | 1.90E-07 | 6.63E-07 |
| P2RY13 | 1.328649 | 2.317346 | 1.91E-07 | 6.66E-07 |
| LRAT | 1.840713 | 1.39167 | 1.92E-07 | 6.70E-07 |
| PABPC1L | 1.477713 | 4.993546 | 1.93E-07 | 6.72E-07 |
| SNX32 | 1.154921 | 0.397316 | 1.93E-07 | 6.73E-07 |
| ORC1 | 1.021588 | 1.163893 | 1.94E-07 | 6.75E-07 |
| SEC16B | 1.245381 | -0.64334 | 1.98E-07 | 6.87E-07 |
| CD14 | 1.387747 | 6.55946 | 1.98E-07 | 6.88E-07 |
| PTPN22 | 1.234591 | 1.336016 | 1.99E-07 | 6.92E-07 |
| CARD16 | 1.271875 | 2.288295 | 2.00E-07 | 6.93E-07 |
| HSD17B13 | -1.41858 | -0.96194 | 2.01E-07 | 6.97E-07 |
| SOD2 | 1.203539 | 10.24508 | 2.02E-07 | 7.01E-07 |
| KRT34 | 3.368275 | -2.27959 | 2.03E-07 | 7.02E-07 |
| ADRA2A | -1.35939 | 1.816317 | 2.04E-07 | 7.07E-07 |
| KRT40 | -2.02483 | -3.10115 | 2.05E-07 | 7.10E-07 |
| CRNN | -2.40285 | -3.08914 | 2.06E-07 | 7.13E-07 |
| KCNJ12 | -1.47012 | 2.197966 | 2.06E-07 | 7.15E-07 |
| ACOT11 | -1.03582 | 4.793364 | 2.07E-07 | 7.18E-07 |
| TMEM26 | -1.16087 | 1.029739 | 2.07E-07 | 7.18E-07 |
| ARHGAP20 | -1.25886 | -0.61687 | 2.09E-07 | 7.24E-07 |
| MMP25 | 1.015453 | 0.896768 | 2.09E-07 | 7.25E-07 |
| GULP1 | 1.043527 | 5.911744 | 2.10E-07 | 7.27E-07 |
| EIF5AL1 | 1.653105 | 1.994568 | 2.11E-07 | 7.30E-07 |
| FABP7 | 5.772821 | 1.007831 | 2.12E-07 | 7.32E-07 |
| LYPD6B | -2.07325 | 1.896601 | 2.12E-07 | 7.33E-07 |
| CSRP2 | -1.04479 | 4.078928 | 2.13E-07 | 7.35E-07 |
| SELENOM | -1.24253 | 4.796814 | 2.18E-07 | 7.51E-07 |
| AZU1 | 2.243923 | -2.49887 | 2.22E-07 | 7.64E-07 |
| WNT6 | -1.27973 | -1.90612 | 2.23E-07 | 7.67E-07 |
| OCM | 1.665202 | -2.38284 | 2.23E-07 | 7.67E-07 |
| SOX11 | 4.157721 | -0.00566 | 2.23E-07 | 7.70E-07 |
| GPR173 | 1.472445 | 3.118515 | 2.26E-07 | 7.76E-07 |
| PLA2G4A | -1.03262 | 2.388837 | 2.30E-07 | 7.92E-07 |
| GPR65 | 1.206926 | 2.123442 | 2.31E-07 | 7.94E-07 |
| CHST13 | 1.791212 | 3.728382 | 2.31E-07 | 7.95E-07 |
| TFPI | 1.184022 | 7.434125 | 2.33E-07 | 7.99E-07 |
| PRRT2 | 1.357505 | 1.492918 | 2.33E-07 | 7.99E-07 |
| SCRG1 | -1.43533 | -1.34875 | 2.36E-07 | 8.10E-07 |
| CLPSL2 | 2.924008 | -3.28422 | 2.40E-07 | 8.24E-07 |
| PNOC | -1.83321 | -1.40033 | 2.41E-07 | 8.28E-07 |
| AVPR1B | 1.852098 | 0.307011 | 2.42E-07 | 8.31E-07 |
| TMEM132C | -1.66097 | -0.50103 | 2.43E-07 | 8.32E-07 |
| NPIPB5 | 1.63384 | 1.80025 | 2.45E-07 | 8.39E-07 |
| TNNI3K | -1.25806 | -1.53446 | 2.46E-07 | 8.43E-07 |
| HLA-DPB1 | 1.258904 | 8.048073 | 2.48E-07 | 8.49E-07 |
| DNTT | 4.356226 | -0.77866 | 2.48E-07 | 8.49E-07 |
| C6orf141 | 2.425728 | 1.093548 | 2.49E-07 | 8.52E-07 |
| SLC4A10 | 1.831663 | -1.08544 | 2.57E-07 | 8.77E-07 |
| EGFL8 | 1.506807 | 1.784961 | 2.57E-07 | 8.78E-07 |
| DLX3 | -1.56193 | -1.44777 | 2.64E-07 | 9.01E-07 |
| GABRD | 2.18382 | 0.045195 | 2.64E-07 | 9.02E-07 |
| PLEKHG7 | -1.05032 | -1.36486 | 2.66E-07 | 9.08E-07 |
| NPR3 | -1.39786 | 6.765317 | 2.69E-07 | 9.18E-07 |
| IL10RA | 1.350603 | 4.820405 | 2.72E-07 | 9.27E-07 |
| C5orf49 | 1.166921 | 3.12109 | 2.73E-07 | 9.28E-07 |
| TRIM63 | 3.874481 | 3.67121 | 2.74E-07 | 9.31E-07 |
| ADAMTS13 | 1.110382 | 3.561526 | 2.75E-07 | 9.34E-07 |
| PRSS12 | -1.45445 | 0.050411 | 2.75E-07 | 9.37E-07 |
| GALNT11 | 1.052588 | 8.823488 | 2.76E-07 | 9.40E-07 |
| NLRP12 | 1.358236 | -0.46383 | 2.78E-07 | 9.46E-07 |
| PPP1R1A | -1.189 | 6.492766 | 2.79E-07 | 9.49E-07 |
| SFTPD | -1.04452 | 0.196646 | 2.88E-07 | 9.76E-07 |
| C2orf88 | -1.03989 | 2.647236 | 2.94E-07 | 9.94E-07 |
| FBN3 | -2.34864 | 1.047803 | 2.94E-07 | 9.95E-07 |
| MSLN | 2.655243 | 5.514612 | 2.97E-07 | 1.01E-06 |
| CRYBA1 | 1.519115 | -3.05481 | 2.99E-07 | 1.01E-06 |
| EFCAB1 | 2.617109 | 0.846843 | 3.08E-07 | 1.04E-06 |
| SLC16A8 | 1.484727 | 0.159024 | 3.12E-07 | 1.05E-06 |
| FIBCD1 | 3.732324 | 1.005972 | 3.16E-07 | 1.07E-06 |
| SCG3 | 2.662206 | 0.223087 | 3.17E-07 | 1.07E-06 |
| OVOL1 | -1.37128 | 2.803812 | 3.18E-07 | 1.08E-06 |
| HCN1 | 3.686215 | -2.04425 | 3.19E-07 | 1.08E-06 |
| PCDH11X | -2.20079 | -4.02336 | 3.20E-07 | 1.08E-06 |
| HDC | 2.112628 | 1.06667 | 3.22E-07 | 1.09E-06 |
| SCGB2A2 | -2.93943 | -2.30406 | 3.26E-07 | 1.10E-06 |
| ZNF728 | -2.07739 | -2.05554 | 3.27E-07 | 1.10E-06 |
| CHST4 | 2.158055 | -2.00706 | 3.27E-07 | 1.10E-06 |
| PTPN5 | -1.3472 | -2.0795 | 3.32E-07 | 1.12E-06 |
| NPBWR1 | 4.392414 | -0.11204 | 3.36E-07 | 1.13E-06 |
| MSH5-SAPCD1 | 1.286083 | 1.312207 | 3.40E-07 | 1.14E-06 |
| CD44 | 1.522014 | 7.190576 | 3.44E-07 | 1.16E-06 |
| FAM72D | 1.655023 | -2.64245 | 3.46E-07 | 1.16E-06 |
| CXCR6 | 1.497733 | 1.731095 | 3.48E-07 | 1.17E-06 |
| CST4 | 5.415726 | -1.51757 | 3.49E-07 | 1.17E-06 |
| RUNX2 | 1.02138 | 2.622757 | 3.49E-07 | 1.17E-06 |
| ZNF365 | 1.795284 | 1.564465 | 3.51E-07 | 1.18E-06 |
| HP | 6.025852 | 5.201948 | 3.51E-07 | 1.18E-06 |
| ST8SIA4 | 1.492732 | 3.223267 | 3.59E-07 | 1.20E-06 |
| ACTA2 | -1.13674 | 6.756902 | 3.60E-07 | 1.20E-06 |
| C2orf70 | 1.34839 | 1.273208 | 3.60E-07 | 1.21E-06 |
| VSIG4 | 1.477744 | 4.597308 | 3.61E-07 | 1.21E-06 |
| C1orf61 | 2.444271 | -2.12031 | 3.63E-07 | 1.22E-06 |
| C9orf24 | -1.3084 | 0.3322 | 3.64E-07 | 1.22E-06 |
| SSTR1 | -1.26706 | 2.215028 | 3.69E-07 | 1.23E-06 |
| SQSTM1 | 1.052325 | 8.909314 | 3.70E-07 | 1.23E-06 |
| CLEC18C | -1.86885 | 0.775563 | 3.70E-07 | 1.24E-06 |
| INA | 4.193557 | 0.93149 | 3.83E-07 | 1.28E-06 |
| DOCK2 | 1.271063 | 4.156788 | 3.98E-07 | 1.33E-06 |
| DUSP1 | -1.1017 | 7.792829 | 4.03E-07 | 1.34E-06 |
| POLR2F | 1.084217 | -1.53754 | 4.03E-07 | 1.34E-06 |
| TMEM252 | 2.255496 | 6.580416 | 4.12E-07 | 1.37E-06 |
| NOTCH3 | -1.35246 | 6.063486 | 4.17E-07 | 1.38E-06 |
| ADGRL3 | -1.65052 | 1.394423 | 4.20E-07 | 1.39E-06 |
| C3orf22 | 1.965305 | -3.52528 | 4.25E-07 | 1.41E-06 |
| BRINP2 | 3.489249 | -0.18025 | 4.26E-07 | 1.41E-06 |
| PPT2-EGFL8 | 1.015899 | 0.835096 | 4.28E-07 | 1.42E-06 |
| PRDM7 | -1.32106 | -3.16404 | 4.30E-07 | 1.42E-06 |
| C1QL4 | 2.90286 | 1.929526 | 4.36E-07 | 1.44E-06 |
| SNCB | 4.75784 | -1.05479 | 4.42E-07 | 1.46E-06 |
| FAXC | 1.250959 | 3.056928 | 4.45E-07 | 1.47E-06 |
| CCL5 | 1.740492 | 4.902738 | 4.47E-07 | 1.48E-06 |
| HIST1H2BH | 2.326524 | -2.10394 | 4.52E-07 | 1.49E-06 |
| CAMK2B | 2.013164 | 3.913893 | 4.56E-07 | 1.50E-06 |
| CAMKV | 3.210338 | -1.0405 | 4.56E-07 | 1.50E-06 |
| TMIGD2 | 1.551227 | -1.17962 | 4.64E-07 | 1.53E-06 |
| TPSAB1 | 2.128947 | 3.802367 | 4.65E-07 | 1.53E-06 |
| AGBL3 | 1.067449 | 2.977749 | 4.67E-07 | 1.54E-06 |
| PCSK1 | -1.36964 | -2.20264 | 4.71E-07 | 1.55E-06 |
| SLA2 | 1.393545 | 1.026484 | 4.73E-07 | 1.56E-06 |
| CPLX1 | 1.240063 | 2.706639 | 4.74E-07 | 1.56E-06 |
| DDIAS | 1.165307 | 0.173112 | 4.81E-07 | 1.58E-06 |
| SQLE | -1.0755 | 3.656806 | 4.82E-07 | 1.58E-06 |
| ALDH3A1 | 1.534914 | 1.981413 | 4.85E-07 | 1.59E-06 |
| CLEC1B | 1.864795 | -3.12776 | 4.99E-07 | 1.64E-06 |
| FAM198A | -1.14071 | 0.369089 | 5.05E-07 | 1.65E-06 |
| NAT16 | 3.31135 | -0.70956 | 5.10E-07 | 1.67E-06 |
| LAIR2 | 2.939829 | -1.28685 | 5.12E-07 | 1.68E-06 |
| HTR1D | 2.36005 | -1.10318 | 5.13E-07 | 1.68E-06 |
| CYP27C1 | 2.531101 | 1.255944 | 5.14E-07 | 1.68E-06 |
| LINGO4 | -1.09952 | -0.86735 | 5.24E-07 | 1.71E-06 |
| FOXH1 | 1.898036 | -1.89997 | 5.25E-07 | 1.72E-06 |
| CFHR4 | -2.34236 | -3.98733 | 5.26E-07 | 1.72E-06 |
| MRC1 | -1.20577 | 3.042722 | 5.29E-07 | 1.73E-06 |
| DQX1 | 2.832824 | -2.35635 | 5.35E-07 | 1.75E-06 |
| NPY4R | 3.186606 | -3.55056 | 5.37E-07 | 1.75E-06 |
| LMO7DN | 3.482339 | -1.49276 | 5.37E-07 | 1.75E-06 |
| TTC9 | 1.042553 | 4.377891 | 5.58E-07 | 1.82E-06 |
| SNAP91 | 3.156924 | 0.597605 | 5.60E-07 | 1.82E-06 |
| KHDRBS2 | -1.55279 | -1.60455 | 5.69E-07 | 1.85E-06 |
| HMOX1 | 1.740375 | 7.75106 | 5.70E-07 | 1.86E-06 |
| ARHGAP30 | 1.125311 | 4.379221 | 5.73E-07 | 1.86E-06 |
| RAPGEFL1 | 1.888789 | 2.916863 | 5.79E-07 | 1.88E-06 |
| EPO | -1.88505 | -1.86443 | 5.83E-07 | 1.89E-06 |
| ZNF233 | -1.09398 | -0.0551 | 5.85E-07 | 1.90E-06 |
| SLPI | 2.811997 | 8.076945 | 5.86E-07 | 1.90E-06 |
| TLR7 | 1.51367 | 2.628435 | 5.94E-07 | 1.93E-06 |
| TSPAN19 | 1.995489 | -0.71706 | 5.95E-07 | 1.93E-06 |
| TBC1D3L | 2.330411 | 0.354673 | 6.00E-07 | 1.95E-06 |
| PADI3 | 6.140137 | 2.816917 | 6.02E-07 | 1.95E-06 |
| GPA33 | 1.518408 | -1.56487 | 6.07E-07 | 1.97E-06 |
| ABO | -1.26402 | 4.891609 | 6.08E-07 | 1.97E-06 |
| GTSF1 | 1.670538 | -1.65915 | 6.13E-07 | 1.99E-06 |
| GCOM1 | -1.9643 | -1.98073 | 6.23E-07 | 2.02E-06 |
| DLEU7 | 1.322582 | -0.52426 | 6.42E-07 | 2.07E-06 |
| EGFLAM | -1.19012 | 1.746331 | 6.42E-07 | 2.08E-06 |
| PCDHB14 | 1.069693 | 3.366439 | 6.47E-07 | 2.09E-06 |
| NINJ2 | 1.223246 | 0.706724 | 6.57E-07 | 2.12E-06 |
| MARVELD1 | 1.047886 | 5.368947 | 6.58E-07 | 2.12E-06 |
| CKMT1A | 1.631622 | 2.259347 | 6.76E-07 | 2.18E-06 |
| PCDHB10 | 1.127968 | 1.842121 | 6.78E-07 | 2.19E-06 |
| PRELP | -1.42358 | 4.816977 | 6.82E-07 | 2.20E-06 |
| C5AR1 | 1.144745 | 4.453482 | 6.83E-07 | 2.20E-06 |
| FCRLB | 1.66567 | 3.453114 | 6.89E-07 | 2.22E-06 |
| SEMA5B | 1.657827 | 4.80027 | 7.24E-07 | 2.32E-06 |
| MYH7 | -1.52972 | -2.43884 | 7.30E-07 | 2.34E-06 |
| CLEC3A | -2.31471 | -3.86887 | 7.52E-07 | 2.41E-06 |
| FRMD5 | 1.754198 | 1.661572 | 7.81E-07 | 2.50E-06 |
| SPSB4 | -1.94351 | -1.0048 | 8.01E-07 | 2.56E-06 |
| LUM | -1.51787 | 5.844383 | 8.10E-07 | 2.59E-06 |
| COLEC11 | -1.21413 | 3.42752 | 8.18E-07 | 2.61E-06 |
| NIPAL4 | 3.299261 | 0.446099 | 8.28E-07 | 2.64E-06 |
| CCDC146 | 1.756051 | 5.892498 | 8.29E-07 | 2.64E-06 |
| SLIT1 | 1.42802 | -0.56722 | 8.30E-07 | 2.65E-06 |
| VSTM1 | 2.224503 | -2.31007 | 8.42E-07 | 2.68E-06 |
| WDR93 | 1.017538 | 1.639606 | 8.53E-07 | 2.72E-06 |
| GBP5 | 1.715414 | 2.896649 | 8.53E-07 | 2.72E-06 |
| LEF1 | -1.26313 | 1.629448 | 8.56E-07 | 2.73E-06 |
| GPR45 | 1.724682 | -1.91276 | 8.58E-07 | 2.73E-06 |
| RSPO4 | 1.994628 | 0.110189 | 8.58E-07 | 2.73E-06 |
| B4GALNT2 | -1.6698 | 3.835471 | 8.60E-07 | 2.73E-06 |
| REM1 | -1.07441 | -0.32338 | 8.62E-07 | 2.74E-06 |
| S100A8 | 2.755451 | 4.378508 | 8.67E-07 | 2.75E-06 |
| HIST1H2AE | 1.487349 | 0.518952 | 8.67E-07 | 2.75E-06 |
| SLC6A15 | 3.780893 | 1.168669 | 8.74E-07 | 2.77E-06 |
| PEX5L | -1.28971 | -1.60822 | 8.74E-07 | 2.77E-06 |
| TSPEAR | -1.77213 | -2.75206 | 8.75E-07 | 2.78E-06 |
| TAF7L | -1.25609 | -1.95293 | 8.91E-07 | 2.83E-06 |
| DISP2 | 1.612021 | 0.707753 | 9.08E-07 | 2.88E-06 |
| RGS8 | -1.71249 | -3.28214 | 9.10E-07 | 2.88E-06 |
| TMEM92 | 2.470648 | 1.628148 | 9.12E-07 | 2.89E-06 |
| EFNA2 | 2.349176 | -1.54368 | 9.12E-07 | 2.89E-06 |
| APOBEC3B | 1.165418 | 1.376361 | 9.17E-07 | 2.90E-06 |
| HIST1H4J | 1.88779 | -3.04523 | 9.25E-07 | 2.93E-06 |
| C19orf67 | 1.646397 | -2.22683 | 9.27E-07 | 2.93E-06 |
| CD4 | 1.180433 | 5.925263 | 9.32E-07 | 2.95E-06 |
| TM4SF19-TCTEX1D2 | 2.038481 | -3.64829 | 9.41E-07 | 2.97E-06 |
| CDCA7L | 1.276916 | 3.402785 | 9.44E-07 | 2.98E-06 |
| ADAM7 | 4.835451 | 2.559812 | 9.46E-07 | 2.99E-06 |
| CYP17A1 | -1.84196 | 4.64112 | 9.50E-07 | 3.00E-06 |
| DACT1 | -1.0299 | 2.480785 | 9.51E-07 | 3.00E-06 |
| CFAP54 | 1.417433 | 1.154393 | 9.51E-07 | 3.00E-06 |
| HCLS1 | 1.053781 | 5.303842 | 9.53E-07 | 3.01E-06 |
| VNN2 | 1.726878 | 2.726449 | 9.65E-07 | 3.04E-06 |
| AC023509.3 | 1.34656 | 0.82982 | 9.67E-07 | 3.05E-06 |
| GAL3ST3 | -1.6406 | 1.982943 | 9.71E-07 | 3.06E-06 |
| KLK14 | 1.5003 | -1.63555 | 1.00E-06 | 3.15E-06 |
| BEND6 | 1.40278 | 1.123031 | 1.03E-06 | 3.23E-06 |
| NR5A2 | -1.03976 | 0.241041 | 1.04E-06 | 3.26E-06 |
| XYLB | -1.00563 | 3.30467 | 1.04E-06 | 3.26E-06 |
| TAS2R19 | 1.344262 | -2.09976 | 1.04E-06 | 3.27E-06 |
| C3orf20 | -1.04878 | -2.11925 | 1.04E-06 | 3.28E-06 |
| RSRP1 | 1.049811 | 5.993688 | 1.05E-06 | 3.29E-06 |
| FAM46D | -1.72255 | -3.84305 | 1.06E-06 | 3.31E-06 |
| ZFY | -1.90842 | 1.367877 | 1.06E-06 | 3.32E-06 |
| PHYHIPL | 1.363245 | 6.018927 | 1.06E-06 | 3.33E-06 |
| ANKK1 | 1.416382 | -0.57252 | 1.07E-06 | 3.36E-06 |
| FAM71D | 1.434386 | -1.70424 | 1.08E-06 | 3.38E-06 |
| KCNS2 | -1.8397 | -3.00199 | 1.08E-06 | 3.38E-06 |
| LGALS12 | 2.185946 | 0.638275 | 1.09E-06 | 3.39E-06 |
| SNAI1 | -1.22229 | 1.504502 | 1.09E-06 | 3.41E-06 |
| MYT1L | 2.3996 | -2.31129 | 1.09E-06 | 3.41E-06 |
| GRIK5 | -1.67252 | 3.119832 | 1.10E-06 | 3.42E-06 |
| IL4 | 1.730537 | -3.00859 | 1.10E-06 | 3.43E-06 |
| CKMT1B | 1.666294 | 2.449054 | 1.11E-06 | 3.47E-06 |
| ADGRB2 | 1.395271 | 3.798212 | 1.11E-06 | 3.47E-06 |
| WNT1 | 1.758313 | -3.04822 | 1.11E-06 | 3.47E-06 |
| NXNL2 | 1.303101 | 3.049944 | 1.12E-06 | 3.48E-06 |
| SCRT1 | 3.992161 | 0.290477 | 1.12E-06 | 3.48E-06 |
| SATL1 | -1.58783 | -3.77964 | 1.12E-06 | 3.49E-06 |
| TSACC | 1.074912 | -0.92064 | 1.12E-06 | 3.49E-06 |
| CLIC3 | 1.714751 | 1.548348 | 1.13E-06 | 3.52E-06 |
| FAM186A | 1.375985 | -0.23517 | 1.13E-06 | 3.52E-06 |
| RAB44 | 1.830221 | -1.1284 | 1.14E-06 | 3.54E-06 |
| FAM183A | 1.88424 | 0.713593 | 1.14E-06 | 3.56E-06 |
| ZIM2 | -1.61579 | -3.56944 | 1.15E-06 | 3.57E-06 |
| LINGO1 | -1.20066 | 1.474613 | 1.17E-06 | 3.64E-06 |
| ODF3L1 | 1.20431 | -0.34166 | 1.18E-06 | 3.66E-06 |
| LCP2 | 1.056464 | 4.23036 | 1.18E-06 | 3.66E-06 |
| JPH3 | 3.144953 | 0.273041 | 1.19E-06 | 3.69E-06 |
| CHKB-CPT1B | 1.267446 | 1.071591 | 1.20E-06 | 3.73E-06 |
| MCHR1 | 2.686237 | -0.26186 | 1.21E-06 | 3.74E-06 |
| IBSP | 3.430104 | -1.99577 | 1.21E-06 | 3.75E-06 |
| HIF3A | -1.38978 | 1.738819 | 1.24E-06 | 3.83E-06 |
| NKAPL | -1.12169 | -0.72506 | 1.25E-06 | 3.88E-06 |
| ANKRD23 | 1.082132 | 0.057926 | 1.27E-06 | 3.94E-06 |
| PLB1 | 1.11328 | 1.76219 | 1.28E-06 | 3.94E-06 |
| TTYH1 | 1.973252 | 0.723326 | 1.28E-06 | 3.95E-06 |
| IL12RB1 | 1.234572 | 1.59546 | 1.28E-06 | 3.96E-06 |
| EN2 | 2.996611 | 0.826932 | 1.28E-06 | 3.96E-06 |
| UBQLNL | 1.090084 | -0.2537 | 1.28E-06 | 3.97E-06 |
| TRDN | -2.21778 | -2.65911 | 1.29E-06 | 3.99E-06 |
| CCL4 | 1.591072 | 2.800666 | 1.30E-06 | 4.00E-06 |
| CRIP1 | 1.465702 | 1.698614 | 1.31E-06 | 4.05E-06 |
| RASGRP4 | 1.073448 | 1.175698 | 1.33E-06 | 4.09E-06 |
| PRSS42 | 1.60016 | -0.99418 | 1.35E-06 | 4.15E-06 |
| AQP5 | -1.68475 | -0.23692 | 1.36E-06 | 4.18E-06 |
| YJEFN3 | 1.433656 | 2.160846 | 1.42E-06 | 4.38E-06 |
| ASPA | -1.03323 | 4.668069 | 1.43E-06 | 4.41E-06 |
| KCNQ5 | 1.768164 | -0.22719 | 1.43E-06 | 4.41E-06 |
| NEUROD2 | 1.897103 | -2.94405 | 1.44E-06 | 4.44E-06 |
| B4GALNT4 | 2.89186 | 1.609261 | 1.45E-06 | 4.44E-06 |
| LRP4 | 1.202717 | 3.833515 | 1.45E-06 | 4.46E-06 |
| LRRC3 | 1.316485 | 2.406282 | 1.47E-06 | 4.52E-06 |
| SPSB1 | 1.050001 | 5.246669 | 1.48E-06 | 4.53E-06 |
| TBX3 | -1.50059 | 3.393943 | 1.51E-06 | 4.62E-06 |
| HLA-DMA | 1.02848 | 6.487069 | 1.53E-06 | 4.69E-06 |
| CTLA4 | 1.737386 | -0.46901 | 1.64E-06 | 5.02E-06 |
| TMEM156 | 1.111022 | 0.713285 | 1.65E-06 | 5.04E-06 |
| CLCA2 | 4.959392 | 1.422762 | 1.65E-06 | 5.04E-06 |
| TEX15 | 3.04068 | -0.9403 | 1.65E-06 | 5.04E-06 |
| SPNS3 | 1.331573 | 3.668289 | 1.66E-06 | 5.06E-06 |
| KSR2 | -1.04568 | 2.757134 | 1.67E-06 | 5.09E-06 |
| QPCT | 1.506979 | 5.054645 | 1.68E-06 | 5.11E-06 |
| DRICH1 | 1.729613 | 0.102866 | 1.72E-06 | 5.23E-06 |
| NHSL2 | -1.13505 | 0.109351 | 1.72E-06 | 5.23E-06 |
| ITGAL | 1.185303 | 4.293693 | 1.72E-06 | 5.24E-06 |
| CPN2 | -2.33261 | 3.138281 | 1.72E-06 | 5.25E-06 |
| RAD51AP2 | 2.863401 | -1.85276 | 1.72E-06 | 5.25E-06 |
| DLEC1 | 1.676357 | 1.815193 | 1.72E-06 | 5.25E-06 |
| EMILIN1 | -1.12004 | 4.845811 | 1.73E-06 | 5.27E-06 |
| ROPN1L | 1.088221 | 0.107889 | 1.76E-06 | 5.33E-06 |
| CA1 | 2.116919 | -2.07647 | 1.76E-06 | 5.35E-06 |
| CFAP65 | 1.77682 | 1.672338 | 1.77E-06 | 5.38E-06 |
| CU639417.2 | -1.1992 | 2.79191 | 1.78E-06 | 5.39E-06 |
| OLFML1 | -1.20901 | 2.09755 | 1.78E-06 | 5.39E-06 |
| GLRA3 | -1.94945 | -3.72593 | 1.78E-06 | 5.40E-06 |
| NFE2 | 1.174584 | -0.2581 | 1.79E-06 | 5.43E-06 |
| KIAA1324 | 1.675092 | 1.326813 | 1.79E-06 | 5.44E-06 |
| TCHH | 1.741145 | 0.186716 | 1.80E-06 | 5.44E-06 |
| TCP11 | -1.21385 | -1.75006 | 1.82E-06 | 5.50E-06 |
| DAND5 | 2.232423 | -1.58165 | 1.82E-06 | 5.51E-06 |
| GZMA | 1.615521 | 2.387414 | 1.82E-06 | 5.52E-06 |
| CABP7 | 1.091454 | -1.32443 | 1.84E-06 | 5.58E-06 |
| TMEM221 | 1.312988 | 0.35276 | 1.85E-06 | 5.60E-06 |
| CYP7B1 | -1.0025 | 2.314218 | 1.85E-06 | 5.61E-06 |
| NUPR1 | 1.35792 | 7.202865 | 1.88E-06 | 5.68E-06 |
| TAGAP | 1.169327 | 2.855614 | 1.89E-06 | 5.71E-06 |
| BIN2 | 1.043878 | 3.207629 | 1.95E-06 | 5.87E-06 |
| MYC | 1.142823 | 6.198801 | 1.95E-06 | 5.87E-06 |
| FJX1 | 1.168703 | 3.492174 | 1.95E-06 | 5.88E-06 |
| P2RY2 | -1.05139 | 0.705145 | 1.97E-06 | 5.92E-06 |
| CSGALNACT1 | -1.2066 | 3.547054 | 2.00E-06 | 6.02E-06 |
| KRT36 | 1.976886 | -1.80696 | 2.02E-06 | 6.08E-06 |
| GBX2 | 2.872582 | -3.44757 | 2.03E-06 | 6.09E-06 |
| CTHRC1 | 1.679719 | 2.324127 | 2.03E-06 | 6.10E-06 |
| CCL3 | 1.74206 | 2.704332 | 2.03E-06 | 6.10E-06 |
| OSM | 1.620288 | 1.659418 | 2.05E-06 | 6.14E-06 |
| NPIPA3 | 1.977693 | -1.10323 | 2.06E-06 | 6.19E-06 |
| ANKRD33 | 2.455685 | -2.18053 | 2.07E-06 | 6.20E-06 |
| HS3ST3A1 | -1.63164 | -0.79652 | 2.07E-06 | 6.21E-06 |
| TG | -1.33437 | 2.584929 | 2.13E-06 | 6.36E-06 |
| TCEAL7 | -1.28019 | 0.340591 | 2.14E-06 | 6.40E-06 |
| CXCL17 | 4.09537 | -0.0469 | 2.14E-06 | 6.40E-06 |
| VWDE | 2.154664 | 1.398506 | 2.16E-06 | 6.45E-06 |
| DRC1 | 2.355669 | 0.791483 | 2.16E-06 | 6.45E-06 |
| RUNX3 | 1.310403 | 3.318404 | 2.18E-06 | 6.50E-06 |
| LHX1 | -2.26081 | 2.449572 | 2.22E-06 | 6.62E-06 |
| GJB3 | 2.170197 | 1.865758 | 2.24E-06 | 6.68E-06 |
| VIL1 | -1.63558 | 4.197593 | 2.24E-06 | 6.68E-06 |
| ADGRG5 | -1.28059 | 2.985767 | 2.25E-06 | 6.69E-06 |
| ZBP1 | 1.380402 | 0.369884 | 2.25E-06 | 6.71E-06 |
| REM2 | 1.115611 | 0.052005 | 2.28E-06 | 6.78E-06 |
| NKX2-2 | 5.532946 | -1.51985 | 2.29E-06 | 6.82E-06 |
| NMRK2 | 5.64472 | 6.247292 | 2.32E-06 | 6.89E-06 |
| CAPN8 | -1.80059 | 0.543658 | 2.35E-06 | 6.97E-06 |
| CFAP157 | 1.233432 | 2.442771 | 2.37E-06 | 7.03E-06 |
| CSF1R | 1.056297 | 6.08531 | 2.40E-06 | 7.11E-06 |
| CD163 | 1.608761 | 5.697917 | 2.42E-06 | 7.16E-06 |
| PLCG2 | -1.40155 | 5.800993 | 2.44E-06 | 7.23E-06 |
| EFEMP1 | -1.40611 | 5.594375 | 2.50E-06 | 7.38E-06 |
| RFPL3S | 1.37567 | -1.63848 | 2.53E-06 | 7.47E-06 |
| LILRA5 | 1.485616 | 1.372531 | 2.55E-06 | 7.52E-06 |
| WNT10A | -1.5699 | 1.092306 | 2.59E-06 | 7.63E-06 |
| TTN | 1.038769 | 3.329966 | 2.59E-06 | 7.64E-06 |
| TMEM8C | -1.43143 | -2.3969 | 2.60E-06 | 7.67E-06 |
| SAA2 | 3.302919 | 3.305185 | 2.62E-06 | 7.72E-06 |
| GATA4 | 4.39512 | -1.33032 | 2.63E-06 | 7.72E-06 |
| MPZL2 | 1.066484 | 5.776377 | 2.63E-06 | 7.75E-06 |
| WFDC3 | 1.18942 | 0.365077 | 2.65E-06 | 7.79E-06 |
| LOXL2 | 1.34906 | 4.686494 | 2.66E-06 | 7.81E-06 |
| AL049650.1 | 1.043955 | -3.11471 | 2.66E-06 | 7.83E-06 |
| RBM20 | 1.65711 | 3.330786 | 2.67E-06 | 7.83E-06 |
| IL12B | -1.3749 | -3.30433 | 2.68E-06 | 7.88E-06 |
| ISG15 | 1.109028 | 4.625436 | 2.70E-06 | 7.92E-06 |
| AL353588.1 | 1.294021 | -2.00921 | 2.70E-06 | 7.93E-06 |
| STAP1 | -1.88723 | 1.872204 | 2.70E-06 | 7.93E-06 |
| GPR153 | 1.228085 | 4.063851 | 2.72E-06 | 7.97E-06 |
| TMEM105 | -2.01808 | -1.52744 | 2.73E-06 | 7.99E-06 |
| GLDN | 2.054295 | 1.933931 | 2.73E-06 | 8.00E-06 |
| AC138969.1 | 1.081219 | -1.02104 | 2.73E-06 | 8.00E-06 |
| ENO3 | 1.263173 | 3.709533 | 2.75E-06 | 8.05E-06 |
| ROPN1 | 1.960981 | -3.60876 | 2.79E-06 | 8.17E-06 |
| VWA3B | -1.52975 | -1.91583 | 2.79E-06 | 8.17E-06 |
| IGF2BP2 | 1.190277 | 5.010246 | 2.83E-06 | 8.28E-06 |
| PRUNE2 | 1.249634 | 7.680016 | 2.83E-06 | 8.28E-06 |
| TAS2R20 | 1.127923 | -1.28912 | 2.85E-06 | 8.33E-06 |
| MEIOB | 1.77671 | -3.28298 | 2.85E-06 | 8.34E-06 |
| CRACR2B | 1.197547 | 4.630423 | 2.86E-06 | 8.37E-06 |
| GXYLT2 | 1.118858 | 1.903627 | 2.89E-06 | 8.43E-06 |
| HRASLS2 | -1.34579 | 1.50781 | 2.91E-06 | 8.48E-06 |
| PURG | 2.418989 | 1.113118 | 2.91E-06 | 8.50E-06 |
| HIST1H2BJ | 1.257783 | -1.19189 | 2.92E-06 | 8.52E-06 |
| PDIA2 | 2.389554 | -1.03876 | 2.95E-06 | 8.59E-06 |
| ASTL | -1.36641 | -3.57047 | 2.95E-06 | 8.60E-06 |
| CAMK1D | 1.112641 | 4.420251 | 2.96E-06 | 8.63E-06 |
| DEUP1 | -1.45014 | -2.08113 | 2.97E-06 | 8.65E-06 |
| CYBB | 1.195991 | 5.295884 | 2.97E-06 | 8.66E-06 |
| SERPINA1 | 1.450276 | 9.920699 | 2.98E-06 | 8.67E-06 |
| FAM129A | 1.090814 | 5.525556 | 2.98E-06 | 8.67E-06 |
| OSGIN1 | 1.386189 | 4.869542 | 3.02E-06 | 8.79E-06 |
| SLC39A8 | 1.171231 | 6.285705 | 3.07E-06 | 8.93E-06 |
| TREML4 | 2.363313 | -3.00189 | 3.09E-06 | 8.98E-06 |
| SYT15 | -1.04654 | -1.46695 | 3.13E-06 | 9.09E-06 |
| C8orf88 | -1.07255 | -0.0123 | 3.15E-06 | 9.14E-06 |
| SLC22A7 | -1.92008 | 4.565465 | 3.15E-06 | 9.14E-06 |
| SYT5 | 2.676105 | -0.37437 | 3.17E-06 | 9.19E-06 |
| PRR36 | 1.142145 | 3.689089 | 3.20E-06 | 9.28E-06 |
| CERS1 | 2.67782 | -0.20688 | 3.21E-06 | 9.30E-06 |
| KPNA7 | 1.962155 | -3.1083 | 3.23E-06 | 9.34E-06 |
| HCAR1 | -1.38161 | 0.905284 | 3.24E-06 | 9.38E-06 |
| TRIM36 | 1.028854 | 0.384555 | 3.25E-06 | 9.40E-06 |
| P2RY12 | 1.331043 | 0.466222 | 3.31E-06 | 9.58E-06 |
| DGKI | -1.56875 | 1.453834 | 3.33E-06 | 9.64E-06 |
| SMLR1 | -1.46019 | 0.785563 | 3.37E-06 | 9.73E-06 |
| P2RX2 | -1.81656 | -2.90383 | 3.38E-06 | 9.78E-06 |
| OPN5 | 2.329748 | -3.0041 | 3.39E-06 | 9.81E-06 |
| LCE1C | -2.27466 | -3.68072 | 3.40E-06 | 9.81E-06 |
| GZMH | 1.402233 | 1.157582 | 3.42E-06 | 9.88E-06 |
| AD000671.1 | 1.23123 | -3.48914 | 3.42E-06 | 9.88E-06 |
| LY6G5B | 1.231659 | 1.216638 | 3.43E-06 | 9.89E-06 |
| COL19A1 | -1.33889 | 0.434094 | 3.45E-06 | 9.96E-06 |
| SCN8A | 1.162772 | 2.530287 | 3.46E-06 | 9.99E-06 |
| PCDHB2 | 1.73587 | 2.688635 | 3.49E-06 | 1.01E-05 |
| PHEX | 1.546543 | 0.924757 | 3.50E-06 | 1.01E-05 |
| CPA3 | 1.980533 | 4.043522 | 3.58E-06 | 1.03E-05 |
| GRIN2A | -1.65485 | 0.501652 | 3.60E-06 | 1.04E-05 |
| CA2 | -1.10135 | 7.421438 | 3.62E-06 | 1.04E-05 |
| KRT33A | 3.390422 | -3.6115 | 3.62E-06 | 1.04E-05 |
| LIMD2 | 1.117791 | 4.082227 | 3.67E-06 | 1.05E-05 |
| CD8A | 1.74219 | 3.322786 | 3.72E-06 | 1.07E-05 |
| HES4 | 1.432053 | 3.270394 | 3.75E-06 | 1.08E-05 |
| CST7 | 1.594493 | 2.550845 | 3.80E-06 | 1.09E-05 |
| RRAD | 1.543463 | 6.121626 | 3.82E-06 | 1.10E-05 |
| RUNX1 | 1.194349 | 4.991747 | 3.83E-06 | 1.10E-05 |
| NOTUM | -1.53782 | -1.48515 | 3.84E-06 | 1.10E-05 |
| PCDHGC4 | 1.570187 | -1.54459 | 3.90E-06 | 1.12E-05 |
| SLC5A10 | -1.47772 | 5.058838 | 3.90E-06 | 1.12E-05 |
| HOXB1 | -1.82605 | -2.9027 | 3.91E-06 | 1.12E-05 |
| C19orf38 | 1.074812 | 1.308019 | 3.93E-06 | 1.13E-05 |
| PRODH | -1.046 | 3.404979 | 3.98E-06 | 1.14E-05 |
| SMC1B | 1.611168 | -1.98942 | 3.99E-06 | 1.14E-05 |
| CFAP77 | 2.229585 | -0.17098 | 3.99E-06 | 1.14E-05 |
| CD2 | 1.443936 | 3.074135 | 4.02E-06 | 1.15E-05 |
| MMP9 | 2.22676 | 4.31467 | 4.11E-06 | 1.17E-05 |
| APOC3 | -2.54716 | -0.09775 | 4.12E-06 | 1.17E-05 |
| PPAN-P2RY11 | -1.18525 | -3.98853 | 4.17E-06 | 1.19E-05 |
| GIPC2 | -1.05377 | 5.043638 | 4.17E-06 | 1.19E-05 |
| MATK | 1.498965 | 2.154059 | 4.18E-06 | 1.19E-05 |
| LINC01125 | 1.124438 | 1.231921 | 4.22E-06 | 1.20E-05 |
| CRYBB1 | 1.254831 | -0.41208 | 4.22E-06 | 1.20E-05 |
| PLEKHG6 | 1.537048 | 3.225836 | 4.23E-06 | 1.21E-05 |
| NTN3 | -1.28179 | -3.29583 | 4.24E-06 | 1.21E-05 |
| HTR7 | 1.330293 | -0.59551 | 4.27E-06 | 1.22E-05 |
| CCDC28B | 1.085367 | 3.267804 | 4.29E-06 | 1.22E-05 |
| DPPA4 | 3.411875 | -1.301 | 4.35E-06 | 1.24E-05 |
| C10orf82 | -1.56226 | -0.4542 | 4.37E-06 | 1.24E-05 |
| CD8B | 1.9941 | 1.324482 | 4.38E-06 | 1.25E-05 |
| GJB4 | 2.56988 | 0.841417 | 4.54E-06 | 1.29E-05 |
| GOLGA8A | 1.663297 | 4.698715 | 4.55E-06 | 1.29E-05 |
| CACNB1 | 1.223502 | 2.571157 | 4.62E-06 | 1.31E-05 |
| IGFALS | -1.08555 | 0.473401 | 4.66E-06 | 1.32E-05 |
| C5orf58 | 1.380469 | -2.45058 | 4.66E-06 | 1.32E-05 |
| KRTAP1-1 | 3.210902 | -2.41042 | 4.73E-06 | 1.34E-05 |
| AMZ1 | 1.725383 | -0.87496 | 4.73E-06 | 1.34E-05 |
| PTK6 | 1.896514 | 2.385439 | 4.77E-06 | 1.35E-05 |
| DNER | -1.65597 | 3.965909 | 4.84E-06 | 1.37E-05 |
| NR0B2 | -2.33774 | 2.054368 | 4.86E-06 | 1.37E-05 |
| CDRT4 | 1.261337 | -0.12504 | 4.86E-06 | 1.37E-05 |
| TRPC5OS | -1.09099 | -2.05605 | 4.87E-06 | 1.37E-05 |
| ZNF860 | 1.042147 | 2.164569 | 4.89E-06 | 1.38E-05 |
| AC138696.1 | 1.142234 | -2.91604 | 4.91E-06 | 1.38E-05 |
| HLA-DQB1 | 1.237951 | 7.239152 | 4.93E-06 | 1.39E-05 |
| LHX5 | 2.765288 | -3.23148 | 4.96E-06 | 1.40E-05 |
| FAM72B | 1.257832 | -2.10251 | 5.02E-06 | 1.41E-05 |
| TGM2 | 1.350588 | 8.658729 | 5.03E-06 | 1.42E-05 |
| VSX1 | 2.184616 | -2.35605 | 5.04E-06 | 1.42E-05 |
| GSTM5 | -1.34053 | 0.899531 | 5.06E-06 | 1.43E-05 |
| LONRF2 | -1.30786 | 3.084263 | 5.08E-06 | 1.43E-05 |
| LAMA4 | 1.317118 | 5.629469 | 5.11E-06 | 1.44E-05 |
| MYH15 | 1.435964 | -0.44011 | 5.12E-06 | 1.44E-05 |
| COX8C | 1.421967 | -3.33832 | 5.22E-06 | 1.47E-05 |
| AC069503.2 | 1.261302 | -2.13029 | 5.22E-06 | 1.47E-05 |
| LY6H | 1.669596 | -1.03766 | 5.22E-06 | 1.47E-05 |
| CFD | 1.690352 | 4.815135 | 5.22E-06 | 1.47E-05 |
| KLHL13 | -1.03874 | 4.523333 | 5.23E-06 | 1.47E-05 |
| MSLNL | 3.136979 | -1.78353 | 5.23E-06 | 1.47E-05 |
| NEFM | 2.749445 | 4.581352 | 5.23E-06 | 1.47E-05 |
| ZNRF3 | -1.01411 | -1.04541 | 5.24E-06 | 1.47E-05 |
| COL9A1 | -1.2655 | -1.56681 | 5.25E-06 | 1.47E-05 |
| CTCFL | -1.95628 | -3.1257 | 5.29E-06 | 1.48E-05 |
| GOLGA6L10 | 1.290524 | -0.68236 | 5.30E-06 | 1.49E-05 |
| SERPINI1 | -1.08165 | 3.273312 | 5.32E-06 | 1.49E-05 |
| LRFN5 | 2.914204 | 2.183144 | 5.32E-06 | 1.49E-05 |
| FRZB | -1.20528 | 3.844285 | 5.39E-06 | 1.51E-05 |
| AMDHD1 | -1.00656 | 2.339907 | 5.41E-06 | 1.52E-05 |
| ZAP70 | 1.245172 | 2.171655 | 5.42E-06 | 1.52E-05 |
| VCX3A | 2.733199 | -3.36774 | 5.44E-06 | 1.52E-05 |
| OLIG3 | 3.767698 | -1.73998 | 5.46E-06 | 1.53E-05 |
| BGLAP | 1.299412 | -0.19819 | 5.47E-06 | 1.53E-05 |
| ERVV-2 | 3.961645 | 1.460395 | 5.50E-06 | 1.54E-05 |
| ANXA10 | 2.514362 | -2.71072 | 5.51E-06 | 1.54E-05 |
| GFI1 | 1.226816 | 0.496989 | 5.56E-06 | 1.55E-05 |
| GSTT2B | -1.1349 | 0.445615 | 5.62E-06 | 1.57E-05 |
| HLA-DQB2 | 1.652812 | 3.946107 | 5.70E-06 | 1.59E-05 |
| IQANK1 | 1.002747 | 3.187069 | 5.71E-06 | 1.59E-05 |
| GPR37 | 1.810001 | 2.308569 | 5.77E-06 | 1.61E-05 |
| GDPD3 | -1.09286 | 2.369341 | 5.78E-06 | 1.61E-05 |
| STEAP4 | -1.14391 | 2.775374 | 5.81E-06 | 1.62E-05 |
| SDR42E2 | 1.098965 | -1.67803 | 5.84E-06 | 1.62E-05 |
| SPN | 1.105611 | 3.467746 | 5.84E-06 | 1.62E-05 |
| FPR3 | 1.417931 | 3.693374 | 5.98E-06 | 1.66E-05 |
| LGALS9B | 1.861661 | -3.51509 | 6.00E-06 | 1.67E-05 |
| COX6B2 | 1.924377 | -0.65217 | 6.07E-06 | 1.68E-05 |
| FAM181B | -1.46592 | -0.17057 | 6.19E-06 | 1.72E-05 |
| FAM72A | 1.011913 | -1.8101 | 6.19E-06 | 1.72E-05 |
| FAM83D | -1.10982 | 1.533153 | 6.23E-06 | 1.73E-05 |
| CYBRD1 | -1.03329 | 5.533839 | 6.30E-06 | 1.75E-05 |
| AMY2B | 1.263831 | 3.616235 | 6.31E-06 | 1.75E-05 |
| NDRG4 | 1.634109 | 3.941565 | 6.34E-06 | 1.76E-05 |
| PLK5 | 4.381148 | 0.232511 | 6.36E-06 | 1.76E-05 |
| COLEC10 | 1.729401 | 0.95137 | 6.44E-06 | 1.78E-05 |
| PCDHB16 | 1.410587 | 3.891166 | 6.55E-06 | 1.81E-05 |
| PCDHGA12 | -1.18922 | 0.207513 | 6.56E-06 | 1.81E-05 |
| OTC | -1.99976 | -4.06975 | 6.57E-06 | 1.82E-05 |
| PRTN3 | 2.523522 | -2.25204 | 6.62E-06 | 1.83E-05 |
| DPYSL5 | 3.131617 | 0.349605 | 6.66E-06 | 1.84E-05 |
| C14orf132 | -1.1453 | 3.382435 | 6.68E-06 | 1.84E-05 |
| ENPP7 | -1.65166 | 2.021342 | 6.70E-06 | 1.85E-05 |
| PLEKHG4 | 1.128317 | 3.324529 | 6.85E-06 | 1.89E-05 |
| LCN1 | 4.370766 | -3.35976 | 6.87E-06 | 1.89E-05 |
| AKR1C3 | 1.266461 | 7.495698 | 6.92E-06 | 1.90E-05 |
| KRT27 | 2.844282 | -1.80741 | 6.94E-06 | 1.91E-05 |
| ALOXE3 | 1.740004 | -1.58237 | 6.94E-06 | 1.91E-05 |
| VWA3A | 1.905113 | 0.484954 | 7.00E-06 | 1.92E-05 |
| NRG3 | 1.360236 | 1.906817 | 7.00E-06 | 1.92E-05 |
| VENTX | 1.196371 | 0.918048 | 7.02E-06 | 1.93E-05 |
| ADAM11 | 1.473372 | 0.169682 | 7.05E-06 | 1.93E-05 |
| C10orf71 | -2.67103 | -2.47327 | 7.07E-06 | 1.94E-05 |
| SLC38A3 | -1.98779 | 2.203182 | 7.15E-06 | 1.96E-05 |
| CNKSR1 | -1.62633 | 2.506629 | 7.23E-06 | 1.98E-05 |
| HAVCR2 | 1.447236 | 6.011643 | 7.26E-06 | 1.99E-05 |
| GREM1 | -1.77264 | 1.580811 | 7.28E-06 | 1.99E-05 |
| CYP26A1 | 4.622022 | 1.119025 | 7.35E-06 | 2.01E-05 |
| COL12A1 | -1.02652 | 5.147464 | 7.46E-06 | 2.04E-05 |
| C9orf57 | 3.835361 | -3.22215 | 7.60E-06 | 2.07E-05 |
| EXOC3L4 | 1.133542 | 3.662284 | 7.60E-06 | 2.08E-05 |
| MBL2 | 3.843823 | -3.12524 | 7.63E-06 | 2.08E-05 |
| CD48 | 1.275898 | 3.595733 | 7.65E-06 | 2.09E-05 |
| TMEM35A | -1.43753 | -0.89196 | 7.74E-06 | 2.11E-05 |
| MAMDC4 | 1.079369 | 4.06142 | 7.80E-06 | 2.13E-05 |
| FSHR | 2.014943 | -0.76644 | 7.90E-06 | 2.15E-05 |
| UNC79 | 1.089938 | 2.133327 | 7.93E-06 | 2.16E-05 |
| BDNF | 1.633441 | 1.475626 | 7.98E-06 | 2.17E-05 |
| CD3D | 1.564905 | 2.09112 | 7.98E-06 | 2.17E-05 |
| LY6D | 5.623532 | 2.598972 | 8.04E-06 | 2.19E-05 |
| TMSB15B | 1.283141 | -3.1648 | 8.09E-06 | 2.20E-05 |
| SAA1 | 3.015101 | 4.977827 | 8.10E-06 | 2.20E-05 |
| REG1B | 3.927961 | -0.75152 | 8.19E-06 | 2.23E-05 |
| CHRFAM7A | 1.154796 | -1.29654 | 8.34E-06 | 2.26E-05 |
| SOHLH1 | 3.797919 | -3.62165 | 8.38E-06 | 2.28E-05 |
| NEU4 | -1.67722 | 1.146773 | 8.41E-06 | 2.28E-05 |
| FGF17 | 1.525113 | -0.70224 | 8.44E-06 | 2.29E-05 |
| PDE10A | -1.4867 | 3.139846 | 8.44E-06 | 2.29E-05 |
| CEACAM4 | 1.392476 | -1.23851 | 8.56E-06 | 2.32E-05 |
| LYG2 | 1.265087 | -2.36237 | 8.56E-06 | 2.32E-05 |
| ZFR2 | 2.815822 | 0.202774 | 8.58E-06 | 2.32E-05 |
| GABBR1 | 1.114383 | 5.006839 | 8.66E-06 | 2.34E-05 |
| CRTAM | 1.454784 | -0.15496 | 8.77E-06 | 2.37E-05 |
| RTBDN | 2.336527 | -3.20291 | 8.82E-06 | 2.38E-05 |
| EFCAB13 | 1.272455 | 2.736671 | 8.85E-06 | 2.39E-05 |
| KRT7 | 1.767383 | 9.041754 | 8.88E-06 | 2.40E-05 |
| UNC5B | -1.0457 | 4.418278 | 8.92E-06 | 2.41E-05 |
| EOMES | 1.560187 | 0.782156 | 8.98E-06 | 2.42E-05 |
| FP565260.3 | -1.03688 | 0.388204 | 9.03E-06 | 2.44E-05 |
| MMP17 | 1.844382 | 0.873813 | 9.08E-06 | 2.45E-05 |
| COL13A1 | -1.02498 | 0.131534 | 9.12E-06 | 2.46E-05 |
| BIRC3 | 1.287147 | 6.185524 | 9.14E-06 | 2.46E-05 |
| AL133352.1 | 1.01123 | -2.34275 | 9.19E-06 | 2.48E-05 |
| PEBP4 | -1.41701 | -0.69317 | 9.21E-06 | 2.48E-05 |
| MAB21L2 | -1.40213 | -4.0657 | 9.24E-06 | 2.49E-05 |
| C9orf135 | -1.42364 | -0.77533 | 9.26E-06 | 2.49E-05 |
| TFAP2A | -1.76815 | 3.076173 | 9.44E-06 | 2.54E-05 |
| LAG3 | 1.949134 | 1.792699 | 9.45E-06 | 2.54E-05 |
| NPPA | 1.364992 | -2.73181 | 9.49E-06 | 2.55E-05 |
| ARMC12 | 1.32771 | -0.88924 | 9.51E-06 | 2.56E-05 |
| CHST1 | -1.10702 | 1.277313 | 9.54E-06 | 2.57E-05 |
| MRAP2 | -1.42381 | 1.219829 | 9.63E-06 | 2.59E-05 |
| CRB1 | -1.06056 | -1.20872 | 9.74E-06 | 2.62E-05 |
| SLC6A11 | 3.499194 | -1.64616 | 9.90E-06 | 2.66E-05 |
| MYO18B | -1.70638 | -2.13932 | 9.96E-06 | 2.67E-05 |
| SSTR3 | -1.47762 | -2.58705 | 1.00E-05 | 2.69E-05 |
| ACY3 | -1.05729 | 5.331389 | 1.01E-05 | 2.70E-05 |
| CD6 | 1.167066 | 2.658091 | 1.01E-05 | 2.71E-05 |
| PHF21B | -1.35551 | 0.021421 | 1.01E-05 | 2.71E-05 |
| NEURL1 | -1.51575 | -0.21429 | 1.01E-05 | 2.72E-05 |
| MAGEC2 | 8.443024 | -0.1446 | 1.02E-05 | 2.73E-05 |
| SCGB3A1 | 1.823685 | -0.13576 | 1.02E-05 | 2.74E-05 |
| CHODL | -1.51415 | 1.061223 | 1.02E-05 | 2.74E-05 |
| KIR2DL3 | 2.299936 | -3.08911 | 1.04E-05 | 2.78E-05 |
| IL9R | 1.988792 | -1.64211 | 1.04E-05 | 2.79E-05 |
| WNT2B | 1.539457 | 3.570807 | 1.06E-05 | 2.83E-05 |
| RAMP1 | -1.3057 | 1.646518 | 1.09E-05 | 2.91E-05 |
| CERS3 | 2.807983 | -1.3437 | 1.09E-05 | 2.92E-05 |
| AGAP9 | 1.121438 | 2.734636 | 1.10E-05 | 2.93E-05 |
| CALY | 1.943539 | -2.33734 | 1.10E-05 | 2.94E-05 |
| DSCAML1 | 1.012297 | 4.130989 | 1.10E-05 | 2.95E-05 |
| ADAMTS10 | 1.076872 | 3.533146 | 1.11E-05 | 2.95E-05 |
| UGT3A1 | -1.76275 | 5.464118 | 1.11E-05 | 2.96E-05 |
| TMEM130 | 1.97302 | 5.467127 | 1.11E-05 | 2.97E-05 |
| HIST1H2AI | 2.432028 | -2.60428 | 1.12E-05 | 2.99E-05 |
| C8orf4 | -1.40171 | 5.82815 | 1.13E-05 | 3.01E-05 |
| AOAH | 1.150377 | 3.173506 | 1.13E-05 | 3.01E-05 |
| THRSP | -1.79362 | 0.011715 | 1.13E-05 | 3.02E-05 |
| ITPR3 | 1.024233 | 6.885781 | 1.14E-05 | 3.03E-05 |
| SLC5A4 | -1.19909 | -1.68571 | 1.14E-05 | 3.04E-05 |
| B3GALT1 | 2.300628 | -0.36682 | 1.16E-05 | 3.08E-05 |
| OLFM4 | -2.19807 | 1.451208 | 1.16E-05 | 3.09E-05 |
| KLRB1 | 1.18935 | 1.913281 | 1.16E-05 | 3.09E-05 |
| ITGA11 | -1.181 | 3.03227 | 1.17E-05 | 3.10E-05 |
| VMO1 | 1.046723 | 1.700879 | 1.19E-05 | 3.16E-05 |
| RND2 | 1.154048 | 3.033692 | 1.20E-05 | 3.18E-05 |
| SLC6A17 | -1.63598 | 2.165223 | 1.20E-05 | 3.19E-05 |
| RASSF10 | -1.04912 | 2.686311 | 1.21E-05 | 3.20E-05 |
| TMEM108 | -1.24988 | -0.57168 | 1.21E-05 | 3.20E-05 |
| GYS2 | 1.1342 | -0.3735 | 1.22E-05 | 3.25E-05 |
| TBC1D10C | 1.098141 | 2.729011 | 1.23E-05 | 3.27E-05 |
| FBXL16 | 1.387344 | 5.608669 | 1.23E-05 | 3.27E-05 |
| TBX4 | 2.775118 | -1.96894 | 1.24E-05 | 3.28E-05 |
| UPK1B | 2.085856 | 5.024429 | 1.24E-05 | 3.29E-05 |
| ZMAT1 | 1.051232 | 5.104523 | 1.24E-05 | 3.29E-05 |
| CYP19A1 | 1.769845 | -1.9706 | 1.25E-05 | 3.32E-05 |
| HOXD1 | -1.33444 | 0.239983 | 1.26E-05 | 3.32E-05 |
| ANKRD34C | -1.84873 | -3.57441 | 1.27E-05 | 3.36E-05 |
| ATP1A4 | 2.082071 | -1.42091 | 1.28E-05 | 3.38E-05 |
| CDH1 | -1.07436 | 7.132135 | 1.28E-05 | 3.38E-05 |
| TGM5 | 3.258586 | -0.86501 | 1.32E-05 | 3.48E-05 |
| GNG8 | -1.35216 | -3.66442 | 1.34E-05 | 3.52E-05 |
| AL080251.1 | -1.29671 | -3.85572 | 1.34E-05 | 3.54E-05 |
| C2 | 1.744094 | 4.621825 | 1.35E-05 | 3.56E-05 |
| DPEP3 | 1.531687 | -2.03403 | 1.35E-05 | 3.56E-05 |
| OTX1 | 2.89941 | -1.50309 | 1.37E-05 | 3.62E-05 |
| ARHGDIG | 2.500223 | -1.56878 | 1.38E-05 | 3.64E-05 |
| TPRG1 | 1.022317 | 0.153477 | 1.39E-05 | 3.65E-05 |
| MYRFL | 1.87893 | 1.739762 | 1.39E-05 | 3.65E-05 |
| TSGA10IP | 1.622777 | -2.59574 | 1.39E-05 | 3.65E-05 |
| NPW | 1.309107 | -1.90347 | 1.40E-05 | 3.69E-05 |
| CSDC2 | -1.15819 | 4.73019 | 1.40E-05 | 3.69E-05 |
| CTSW | 1.498172 | 2.481102 | 1.40E-05 | 3.69E-05 |
| NTN1 | -1.44214 | 2.567063 | 1.41E-05 | 3.69E-05 |
| RFTN2 | -1.00302 | 1.229313 | 1.43E-05 | 3.76E-05 |
| FASLG | 1.491877 | -0.62659 | 1.44E-05 | 3.78E-05 |
| GSG1 | 2.301586 | 1.416972 | 1.45E-05 | 3.81E-05 |
| CDKN2C | 1.053624 | 3.956288 | 1.45E-05 | 3.81E-05 |
| PRDM8 | -1.04502 | -0.43138 | 1.46E-05 | 3.82E-05 |
| DBX2 | -2.23022 | -3.59005 | 1.46E-05 | 3.84E-05 |
| CD7 | 1.374892 | 2.248588 | 1.47E-05 | 3.86E-05 |
| AKAP4 | -2.21485 | -3.96665 | 1.48E-05 | 3.87E-05 |
| ADRB2 | 1.33958 | 2.493716 | 1.48E-05 | 3.87E-05 |
| GDF5OS | 1.933512 | -3.88731 | 1.48E-05 | 3.87E-05 |
| HIST1H2BL | 1.775448 | -3.49662 | 1.48E-05 | 3.88E-05 |
| UCP1 | -1.40043 | -4.0091 | 1.49E-05 | 3.90E-05 |
| AC010547.5 | 1.393106 | -2.49333 | 1.50E-05 | 3.93E-05 |
| DNAH12 | 1.040756 | -0.69519 | 1.51E-05 | 3.95E-05 |
| LILRA1 | 1.054355 | 0.824763 | 1.51E-05 | 3.95E-05 |
| AC010255.3 | 2.105422 | -3.94383 | 1.53E-05 | 3.99E-05 |
| TNFSF8 | 1.188342 | 1.059331 | 1.54E-05 | 4.04E-05 |
| DKK4 | 5.087511 | -0.3346 | 1.56E-05 | 4.07E-05 |
| CD1A | 2.373357 | -1.22966 | 1.56E-05 | 4.07E-05 |
| TLE6 | -1.22147 | 0.376544 | 1.57E-05 | 4.09E-05 |
| RBFOX3 | -1.23987 | -2.00129 | 1.57E-05 | 4.10E-05 |
| TLR8 | 1.282719 | 1.331958 | 1.58E-05 | 4.13E-05 |
| CHST9 | 1.400127 | 4.307873 | 1.59E-05 | 4.16E-05 |
| SOX3 | 3.188413 | -2.04863 | 1.59E-05 | 4.16E-05 |
| CEACAM6 | 4.248021 | 1.27472 | 1.60E-05 | 4.17E-05 |
| ZFP42 | -1.94911 | -3.84709 | 1.60E-05 | 4.17E-05 |
| GUCY2D | 1.2618 | -0.7278 | 1.62E-05 | 4.22E-05 |
| ARID3C | 1.926344 | -2.71996 | 1.62E-05 | 4.22E-05 |
| SPDEF | 2.547792 | -0.43108 | 1.62E-05 | 4.22E-05 |
| SMAD9 | 1.155531 | 4.884 | 1.64E-05 | 4.27E-05 |
| SLC13A4 | 1.228687 | 1.166637 | 1.64E-05 | 4.27E-05 |
| CHIA | -1.64627 | -2.61253 | 1.66E-05 | 4.33E-05 |
| MAGEA1 | 5.965865 | -2.57965 | 1.67E-05 | 4.34E-05 |
| OR1F1 | 3.259766 | -3.89419 | 1.67E-05 | 4.34E-05 |
| IGFBP3 | 1.651316 | 8.930204 | 1.70E-05 | 4.40E-05 |
| NRG1 | 1.073534 | 4.700503 | 1.71E-05 | 4.44E-05 |
| HIST1H3B | 2.298184 | -3.91362 | 1.72E-05 | 4.46E-05 |
| CYP4F8 | 6.287952 | 0.203668 | 1.72E-05 | 4.46E-05 |
| FO681492.1 | -1.04897 | -2.31467 | 1.73E-05 | 4.48E-05 |
| GABRB3 | -1.59441 | 3.151456 | 1.74E-05 | 4.50E-05 |
| FAM3D | 2.238974 | 1.060857 | 1.75E-05 | 4.53E-05 |
| SIX4 | -1.00094 | 1.328478 | 1.79E-05 | 4.61E-05 |
| SLC6A18 | -1.78663 | 2.893484 | 1.79E-05 | 4.61E-05 |
| CASQ1 | 1.719659 | -0.70206 | 1.79E-05 | 4.62E-05 |
| SIRPG | 1.53343 | 0.81306 | 1.80E-05 | 4.64E-05 |
| NPAS4 | -1.61827 | -3.40379 | 1.83E-05 | 4.72E-05 |
| ASCL1 | 4.728636 | -1.97549 | 1.85E-05 | 4.77E-05 |
| ISLR2 | -1.08464 | -0.84701 | 1.85E-05 | 4.78E-05 |
| SOWAHD | 1.09919 | 0.547096 | 1.89E-05 | 4.86E-05 |
| ADAMTS4 | -1.30907 | 3.331013 | 1.90E-05 | 4.88E-05 |
| VWCE | 1.425241 | 2.65409 | 1.91E-05 | 4.92E-05 |
| CNPY1 | 3.1931 | -3.21344 | 1.93E-05 | 4.97E-05 |
| GFAP | 1.760355 | 1.756949 | 1.94E-05 | 4.98E-05 |
| FLRT3 | 1.152217 | 7.201197 | 1.94E-05 | 4.98E-05 |
| CST6 | 2.085209 | 2.493538 | 1.95E-05 | 5.01E-05 |
| MUC22 | 2.255337 | -2.65458 | 1.95E-05 | 5.01E-05 |
| FAM25A | 3.66094 | -3.78453 | 1.95E-05 | 5.02E-05 |
| GPR87 | 3.974445 | 0.459336 | 1.96E-05 | 5.03E-05 |
| C17orf50 | 1.443125 | -1.63328 | 1.99E-05 | 5.10E-05 |
| CSPG4 | -1.01074 | 3.706722 | 1.99E-05 | 5.12E-05 |
| CLMP | -1.12713 | 2.048072 | 2.01E-05 | 5.14E-05 |
| CDC42BPG | 1.036213 | 5.915661 | 2.03E-05 | 5.20E-05 |
| TMC5 | 2.013852 | 2.029772 | 2.04E-05 | 5.22E-05 |
| UTY | -1.78542 | 2.264149 | 2.06E-05 | 5.28E-05 |
| GPD1 | -1.24905 | 6.939517 | 2.15E-05 | 5.51E-05 |
| SPDYA | 1.052473 | -0.53992 | 2.16E-05 | 5.51E-05 |
| WFDC2 | 1.290275 | 8.928665 | 2.16E-05 | 5.52E-05 |
| NRXN1 | -1.71968 | -1.132 | 2.18E-05 | 5.56E-05 |
| FLNC | 1.170532 | 5.791534 | 2.19E-05 | 5.60E-05 |
| AL049839.2 | -1.57444 | -1.56401 | 2.20E-05 | 5.61E-05 |
| ASRGL1 | 1.106127 | 6.863164 | 2.21E-05 | 5.64E-05 |
| MGP | -1.15292 | 6.90637 | 2.22E-05 | 5.65E-05 |
| SCT | 1.465509 | -1.68093 | 2.23E-05 | 5.68E-05 |
| BRS3 | 3.222686 | -1.58012 | 2.23E-05 | 5.68E-05 |
| THEG | 3.225222 | -3.4842 | 2.25E-05 | 5.72E-05 |
| GALNTL6 | 2.008789 | -0.65568 | 2.25E-05 | 5.73E-05 |
| JPH2 | 1.258116 | 3.660103 | 2.26E-05 | 5.76E-05 |
| ERMN | 1.249743 | -1.3182 | 2.27E-05 | 5.77E-05 |
| NQO1 | 1.729687 | 7.436871 | 2.28E-05 | 5.80E-05 |
| ATP13A4 | 1.251224 | 1.429138 | 2.33E-05 | 5.93E-05 |
| AC005885.1 | 3.542958 | -3.31995 | 2.34E-05 | 5.93E-05 |
| C17orf74 | 2.120379 | -4.1114 | 2.35E-05 | 5.96E-05 |
| PSG3 | 2.967994 | -3.48336 | 2.37E-05 | 6.02E-05 |
| AC005520.1 | 1.054991 | -2.92964 | 2.38E-05 | 6.04E-05 |
| ADORA1 | 1.075177 | 5.018595 | 2.39E-05 | 6.05E-05 |
| PLPPR3 | 2.843347 | -2.04238 | 2.40E-05 | 6.10E-05 |
| SV2B | 2.101219 | 3.035235 | 2.42E-05 | 6.14E-05 |
| ORM2 | 2.285765 | -3.25246 | 2.44E-05 | 6.17E-05 |
| CCDC73 | 1.030441 | -0.89043 | 2.44E-05 | 6.19E-05 |
| HAL | 1.127428 | -0.93811 | 2.46E-05 | 6.22E-05 |
| CLEC4C | -1.36903 | -3.67903 | 2.49E-05 | 6.29E-05 |
| DDX3Y | -1.75505 | 4.066473 | 2.49E-05 | 6.31E-05 |
| AC114296.1 | -1.0313 | -1.24437 | 2.51E-05 | 6.35E-05 |
| GNG13 | 1.931427 | -3.88445 | 2.52E-05 | 6.38E-05 |
| ASGR1 | 1.184045 | 0.67707 | 2.54E-05 | 6.43E-05 |
| HS6ST3 | -1.97667 | 0.517937 | 2.56E-05 | 6.47E-05 |
| APLP1 | -1.25332 | 1.643654 | 2.57E-05 | 6.50E-05 |
| SECTM1 | 1.145661 | 5.165444 | 2.61E-05 | 6.57E-05 |
| MSI1 | -1.23218 | -0.28203 | 2.61E-05 | 6.58E-05 |
| MOGAT1 | 1.402493 | -0.82855 | 2.62E-05 | 6.60E-05 |
| LGI3 | 1.768367 | 0.564308 | 2.65E-05 | 6.66E-05 |
| S100A1 | 1.726536 | 6.843118 | 2.65E-05 | 6.67E-05 |
| CILP2 | 1.505989 | -0.72136 | 2.65E-05 | 6.68E-05 |
| TTC16 | 1.005649 | 0.143148 | 2.68E-05 | 6.74E-05 |
| GPR141 | 1.177063 | -1.14158 | 2.68E-05 | 6.75E-05 |
| TPSB2 | 1.791889 | 3.987057 | 2.69E-05 | 6.75E-05 |
| KRTDAP | 1.728351 | -2.26712 | 2.69E-05 | 6.75E-05 |
| SHISA6 | -1.71023 | 1.248225 | 2.69E-05 | 6.77E-05 |
| RP1L1 | -1.16515 | -2.18969 | 2.70E-05 | 6.78E-05 |
| CELF4 | 1.330843 | 1.114461 | 2.75E-05 | 6.90E-05 |
| IL5RA | -1.07511 | -1.31218 | 2.79E-05 | 7.00E-05 |
| ACSM2B | -1.15987 | 7.235814 | 2.79E-05 | 7.00E-05 |
| RYR3 | 1.706643 | 2.927772 | 2.87E-05 | 7.18E-05 |
| GPR78 | 3.369681 | -0.69919 | 2.88E-05 | 7.21E-05 |
| METTL7B | 1.087384 | 6.17327 | 2.90E-05 | 7.25E-05 |
| GLOD5 | -1.13851 | 1.200023 | 2.91E-05 | 7.26E-05 |
| TGM3 | 2.267707 | 0.0367 | 2.93E-05 | 7.32E-05 |
| HTR2C | 3.852244 | -3.09134 | 2.97E-05 | 7.40E-05 |
| Z82206.1 | 1.031669 | -2.59947 | 2.97E-05 | 7.42E-05 |
| PAGE1 | 7.984221 | 0.327219 | 2.98E-05 | 7.42E-05 |
| BPIFB2 | -2.30762 | -3.8285 | 3.00E-05 | 7.48E-05 |
| PTPRT | -1.6602 | -1.54061 | 3.00E-05 | 7.49E-05 |
| DLX1 | 1.492036 | -2.54353 | 3.02E-05 | 7.54E-05 |
| BRINP3 | -2.39244 | -0.24185 | 3.04E-05 | 7.56E-05 |
| USP9Y | -1.7676 | 3.124093 | 3.04E-05 | 7.57E-05 |
| SBSN | 4.487546 | 1.088705 | 3.05E-05 | 7.58E-05 |
| FAM135B | 1.590317 | 1.831147 | 3.05E-05 | 7.59E-05 |
| FGL2 | -1.1183 | 6.140653 | 3.06E-05 | 7.61E-05 |
| PPP2R2C | 2.887954 | 2.748519 | 3.08E-05 | 7.65E-05 |
| AGAP5 | 1.030513 | -1.37396 | 3.14E-05 | 7.80E-05 |
| KLHL38 | -1.43135 | -3.19154 | 3.15E-05 | 7.84E-05 |
| AC113554.2 | -1.27454 | -3.42947 | 3.20E-05 | 7.96E-05 |
| AC087289.3 | 1.182907 | -2.43013 | 3.22E-05 | 7.98E-05 |
| CNTN5 | -1.4869 | -1.07348 | 3.22E-05 | 8.00E-05 |
| VCX | 2.211551 | -3.2427 | 3.32E-05 | 8.23E-05 |
| GLI2 | -1.07641 | 0.052401 | 3.33E-05 | 8.25E-05 |
| PIR | 1.006024 | 3.76766 | 3.34E-05 | 8.27E-05 |
| HLA-DQA1 | 1.17667 | 6.946589 | 3.35E-05 | 8.30E-05 |
| ANGPTL4 | 1.870074 | 5.522019 | 3.37E-05 | 8.34E-05 |
| FAM90A1 | 1.187888 | 0.628904 | 3.41E-05 | 8.42E-05 |
| APOL4 | -1.2678 | 2.008002 | 3.44E-05 | 8.50E-05 |
| SIX1 | 1.753977 | 0.163885 | 3.46E-05 | 8.55E-05 |
| CDH18 | -2.12221 | -2.79873 | 3.47E-05 | 8.56E-05 |
| TBX21 | 1.030452 | -0.04351 | 3.52E-05 | 8.68E-05 |
| SAA4 | 2.774962 | -1.80376 | 3.53E-05 | 8.71E-05 |
| NPIPB15 | 1.719269 | 1.779105 | 3.54E-05 | 8.72E-05 |
| GUCA2B | 2.69312 | -1.53845 | 3.55E-05 | 8.75E-05 |
| ANKRD35 | -1.07015 | -0.82754 | 3.58E-05 | 8.81E-05 |
| KIAA2012 | 1.90414 | -2.47277 | 3.62E-05 | 8.92E-05 |
| PRR25 | 1.484865 | -3.13818 | 3.64E-05 | 8.95E-05 |
| FOXD4L1 | 1.058124 | -2.05885 | 3.65E-05 | 8.99E-05 |
| LHX9 | 3.533844 | 0.316765 | 3.66E-05 | 9.00E-05 |
| SYTL1 | 1.524033 | 3.298951 | 3.69E-05 | 9.08E-05 |
| C5orf46 | 3.835297 | -2.1228 | 3.71E-05 | 9.12E-05 |
| KLRC2 | 2.011336 | -3.58676 | 3.72E-05 | 9.15E-05 |
| C8G | 1.338914 | 0.395667 | 3.72E-05 | 9.15E-05 |
| AKR1C4 | 1.473381 | -0.98196 | 3.77E-05 | 9.25E-05 |
| MYOZ3 | -1.02183 | -1.03591 | 3.78E-05 | 9.26E-05 |
| MLIP | 1.298391 | 0.08662 | 3.78E-05 | 9.27E-05 |
| SIGLEC14 | 1.3006 | 1.48159 | 3.80E-05 | 9.32E-05 |
| CD274 | 1.139842 | 3.025933 | 3.80E-05 | 9.32E-05 |
| TGM7 | -2.01921 | -3.84454 | 3.81E-05 | 9.33E-05 |
| ULBP2 | 1.226889 | 0.372877 | 3.84E-05 | 9.40E-05 |
| CCDC155 | 1.922109 | -2.65651 | 3.84E-05 | 9.41E-05 |
| KNDC1 | 1.375009 | 3.558867 | 3.86E-05 | 9.46E-05 |
| REG1A | 3.144599 | 5.232894 | 3.87E-05 | 9.47E-05 |
| NXPH4 | 2.363811 | 2.273624 | 3.92E-05 | 9.59E-05 |
| KLHDC8A | -1.40593 | -1.33016 | 3.92E-05 | 9.60E-05 |
| ASCL4 | -1.67526 | -3.21221 | 3.99E-05 | 9.75E-05 |
| PAX9 | 1.528375 | -1.14627 | 3.99E-05 | 9.76E-05 |
| MAGEA3 | 7.020868 | -0.93632 | 4.01E-05 | 9.78E-05 |
| PMCH | 3.126452 | -3.69173 | 4.06E-05 | 9.91E-05 |
| GPR1 | -1.08058 | -1.08023 | 4.08E-05 | 9.96E-05 |
| TUBA3C | 5.892824 | -1.70087 | 4.11E-05 | 0.000100106 |
| NKAIN3 | 2.477441 | -0.7371 | 4.11E-05 | 0.000100116 |
| RNF157 | 1.090024 | 5.217395 | 4.13E-05 | 0.000100537 |
| ART3 | -1.25056 | -3.29429 | 4.13E-05 | 0.000100691 |
| OBP2A | 6.003963 | -1.00267 | 4.17E-05 | 0.00010161 |
| ONECUT3 | 2.832606 | -2.15696 | 4.21E-05 | 0.000102559 |
| PARP15 | 1.140926 | 0.686123 | 4.22E-05 | 0.000102752 |
| KCND2 | 2.822229 | 0.361557 | 4.23E-05 | 0.000102925 |
| NTSR2 | 5.952322 | -1.90826 | 4.23E-05 | 0.000102947 |
| FOXF2 | 2.006814 | -0.50896 | 4.24E-05 | 0.000103118 |
| IDO1 | 1.252399 | 1.983496 | 4.29E-05 | 0.000104141 |
| BIVM-ERCC5 | 1.51144 | -3.42232 | 4.33E-05 | 0.000105069 |
| PRKAG3 | 1.655876 | -3.47717 | 4.33E-05 | 0.000105069 |
| CCDC129 | 4.061089 | -1.5365 | 4.38E-05 | 0.000106298 |
| CLEC18B | -1.24103 | 2.523418 | 4.43E-05 | 0.00010748 |
| SHOX2 | 2.629426 | -1.11575 | 4.45E-05 | 0.000107779 |
| SLC7A3 | -1.54783 | -3.78835 | 4.51E-05 | 0.000109242 |
| SLC22A9 | -1.27216 | -2.62467 | 4.51E-05 | 0.000109253 |
| ANXA8 | 4.41193 | -0.54961 | 4.51E-05 | 0.000109253 |
| MTRNR2L12 | 1.372638 | 2.416937 | 4.57E-05 | 0.000110403 |
| C2CD4B | -1.12826 | -0.05695 | 4.65E-05 | 0.000112377 |
| SLC18A2 | 1.747354 | 0.975997 | 4.70E-05 | 0.000113509 |
| C20orf141 | 2.971909 | -3.72203 | 4.78E-05 | 0.000115227 |
| GDF3 | -1.70208 | -0.52537 | 4.80E-05 | 0.000115848 |
| SP140 | 1.054746 | 1.247675 | 4.83E-05 | 0.000116381 |
| PDCD1 | 1.552181 | 1.251744 | 4.88E-05 | 0.000117643 |
| TTC6 | 3.340805 | -2.84443 | 5.00E-05 | 0.000120156 |
| CYP4F22 | 2.439332 | -0.00448 | 5.01E-05 | 0.000120481 |
| TAS2R31 | 1.059884 | -2.37235 | 5.01E-05 | 0.000120481 |
| MKRN2OS | 1.04245 | 0.530082 | 5.04E-05 | 0.000121085 |
| CDH26 | 1.564984 | -0.13245 | 5.06E-05 | 0.000121631 |
| SNCG | 1.912294 | 5.206657 | 5.09E-05 | 0.000122314 |
| ZBTB32 | 1.001746 | -0.68673 | 5.11E-05 | 0.000122752 |
| DBH | -1.06995 | 0.234098 | 5.21E-05 | 0.000124856 |
| MAGEA6 | 6.886622 | -0.34981 | 5.22E-05 | 0.000125167 |
| TMEM74 | -1.58833 | -3.50064 | 5.27E-05 | 0.00012618 |
| ARMC4 | 2.485004 | 0.846026 | 5.35E-05 | 0.000127953 |
| CEACAM3 | 1.503357 | -1.75863 | 5.35E-05 | 0.000127977 |
| AIM2 | 1.502397 | -0.41751 | 5.36E-05 | 0.000128264 |
| SAA2-SAA4 | 3.428552 | 0.545378 | 5.39E-05 | 0.000128866 |
| OR13A1 | 2.035352 | -2.84375 | 5.42E-05 | 0.000129681 |
| RTL4 | -1.11887 | -0.6108 | 5.44E-05 | 0.000129989 |
| GSDMC | 2.949542 | -1.81835 | 5.50E-05 | 0.000131344 |
| IZUMO1 | -1.05221 | -3.05747 | 5.50E-05 | 0.000131368 |
| RNF212 | -1.18165 | 2.160856 | 5.51E-05 | 0.000131556 |
| TESPA1 | 1.163422 | 1.31178 | 5.51E-05 | 0.000131588 |
| CCDC182 | 1.949552 | -3.98424 | 5.58E-05 | 0.000133282 |
| SYT4 | -1.59527 | -3.5335 | 5.67E-05 | 0.000135214 |
| CES4A | 1.338937 | 2.59071 | 5.72E-05 | 0.000136288 |
| IGFL4 | 1.796601 | -2.38331 | 5.72E-05 | 0.000136288 |
| RGS16 | -1.03506 | 3.00183 | 5.75E-05 | 0.000136931 |
| HEPHL1 | 2.283951 | 1.174023 | 5.84E-05 | 0.000139034 |
| VGLL1 | -2.16739 | 0.651814 | 5.85E-05 | 0.000139356 |
| MT1E | -1.14989 | 5.439334 | 5.97E-05 | 0.000141885 |
| ENTPD3 | -1.39869 | 0.34469 | 6.10E-05 | 0.000144899 |
| SHANK1 | 1.381609 | 0.424114 | 6.15E-05 | 0.000146012 |
| GNLY | 1.200104 | 1.877398 | 6.18E-05 | 0.000146642 |
| ERN2 | 2.567846 | -1.17666 | 6.22E-05 | 0.000147453 |
| GJB5 | 2.080846 | -0.07256 | 6.23E-05 | 0.000147691 |
| PPP1R27 | 1.399228 | -2.86913 | 6.30E-05 | 0.000149037 |
| OTOP2 | 1.882241 | -3.42185 | 6.30E-05 | 0.000149135 |
| KRT19 | 1.593748 | 9.143687 | 6.32E-05 | 0.000149503 |
| GLIS1 | 1.169477 | 3.682783 | 6.33E-05 | 0.000149805 |
| NHLH2 | 2.448152 | -3.14204 | 6.35E-05 | 0.000150168 |
| ZNF280A | 3.168614 | -3.80774 | 6.36E-05 | 0.000150548 |
| LTBP2 | -1.07679 | 4.938337 | 6.38E-05 | 0.000150814 |
| CASP5 | 1.195367 | -2.77022 | 6.39E-05 | 0.000151071 |
| PRAC2 | 3.010785 | -3.52257 | 6.39E-05 | 0.000151082 |
| WNK2 | 1.961722 | 3.874494 | 6.41E-05 | 0.000151535 |
| CD96 | 1.116875 | 2.122609 | 6.44E-05 | 0.000152171 |
| RTP3 | -1.82157 | -3.32831 | 6.46E-05 | 0.000152575 |
| PSG8 | 4.624947 | -2.20089 | 6.46E-05 | 0.000152683 |
| HCRTR1 | -1.01424 | -3.19234 | 6.47E-05 | 0.000152844 |
| CLPSL1 | 2.844932 | -3.84445 | 6.49E-05 | 0.00015329 |
| TMPRSS3 | -1.41538 | 1.98564 | 6.51E-05 | 0.000153615 |
| NHLH1 | 1.078101 | -2.3783 | 6.57E-05 | 0.000154964 |
| GAGE2A | 6.494578 | -2.15342 | 6.57E-05 | 0.000154989 |
| SFTPC | -2.04807 | -1.29818 | 6.59E-05 | 0.000155466 |
| PPEF1 | 1.124403 | -1.48552 | 6.69E-05 | 0.000157524 |
| SPATA8 | 2.540824 | -3.19814 | 6.79E-05 | 0.000159981 |
| HIST3H3 | 1.683972 | -4.02397 | 6.81E-05 | 0.000160227 |
| S100P | 3.472273 | 3.178127 | 6.87E-05 | 0.000161719 |
| C4B | 1.198289 | 6.077273 | 6.94E-05 | 0.000163168 |
| TUBA3D | 1.428832 | -1.07408 | 6.99E-05 | 0.000164239 |
| KEL | 1.848308 | 0.372225 | 7.03E-05 | 0.000165199 |
| TPSD1 | 2.15526 | 0.81754 | 7.06E-05 | 0.000165749 |
| IRX6 | 1.627273 | 3.924387 | 7.07E-05 | 0.000165965 |
| USH1G | 1.591022 | 1.339107 | 7.07E-05 | 0.000166014 |
| TEKT5 | 1.650817 | -2.88899 | 7.08E-05 | 0.000166253 |
| CEP295NL | 1.045207 | -2.77147 | 7.15E-05 | 0.000167701 |
| MST1R | 1.471204 | 2.869247 | 7.18E-05 | 0.000168479 |
| MS4A6E | 2.138211 | -3.88171 | 7.23E-05 | 0.000169489 |
| ADGRF4 | 2.42035 | 0.122563 | 7.38E-05 | 0.000172873 |
| CNMD | 3.254749 | -0.49972 | 7.40E-05 | 0.000173232 |
| KRT2 | -1.352 | -3.62758 | 7.42E-05 | 0.000173707 |
| ORM1 | 3.056432 | -2.59342 | 7.42E-05 | 0.000173802 |
| ADGRG3 | 1.43634 | 0.402246 | 7.46E-05 | 0.000174562 |
| CARMIL2 | 1.15627 | 1.288091 | 7.46E-05 | 0.000174657 |
| GRID2 | -1.85728 | -3.03965 | 7.47E-05 | 0.000174754 |
| SHC3 | -1.25603 | 1.299623 | 7.51E-05 | 0.000175778 |
| FOXL2 | 2.578584 | -3.39956 | 7.63E-05 | 0.000178346 |
| TEX19 | 2.477876 | -3.51001 | 7.65E-05 | 0.000178745 |
| SLC28A3 | 1.88557 | -0.84433 | 7.79E-05 | 0.000181811 |
| ADAM12 | 1.178403 | 2.722869 | 7.81E-05 | 0.000182239 |
| DPYSL4 | 1.880267 | 0.437854 | 7.95E-05 | 0.000185322 |
| POU2F3 | -1.19175 | -0.06395 | 7.99E-05 | 0.000186112 |
| MEOX2 | -1.61478 | -1.35651 | 8.01E-05 | 0.000186521 |
| SELENOV | 3.132741 | -2.11526 | 8.05E-05 | 0.000187457 |
| IL22RA1 | 1.054542 | 2.751513 | 8.09E-05 | 0.00018834 |
| BARX1 | 3.486472 | -2.49975 | 8.12E-05 | 0.000188952 |
| UTS2 | 1.999163 | -2.19477 | 8.20E-05 | 0.000190731 |
| AL121845.2 | -1.35989 | -3.78936 | 8.21E-05 | 0.000190847 |
| PYDC1 | 4.548617 | -0.35098 | 8.21E-05 | 0.000190871 |
| TMEM45A | 1.361288 | 2.606749 | 8.30E-05 | 0.000192825 |
| CCDC168 | 1.135558 | -2.19165 | 8.30E-05 | 0.000192909 |
| ACTL8 | 3.817551 | -2.5414 | 8.46E-05 | 0.000196239 |
| HSH2D | 1.196315 | 2.204219 | 8.49E-05 | 0.000196756 |
| HOXA13 | 3.039655 | 0.608379 | 8.49E-05 | 0.000196862 |
| ACTL6B | 1.982423 | -3.71179 | 8.52E-05 | 0.00019756 |
| EPHA10 | 1.784259 | 1.124003 | 8.77E-05 | 0.000202884 |
| PYHIN1 | 1.273907 | 0.482147 | 8.80E-05 | 0.000203509 |
| HORMAD1 | 2.055163 | -2.83682 | 8.86E-05 | 0.000204868 |
| CPA4 | 1.919923 | 0.92113 | 8.94E-05 | 0.000206399 |
| B4GALNT1 | 1.823167 | 1.558364 | 8.95E-05 | 0.000206713 |
| LGI4 | -1.09425 | 1.078605 | 8.96E-05 | 0.000206723 |
| MMP7 | 1.305441 | 8.970091 | 8.96E-05 | 0.000206723 |
| HSD17B3 | 1.336344 | -0.36456 | 9.00E-05 | 0.000207561 |
| SCHIP1 | 1.224098 | 2.123536 | 9.04E-05 | 0.000208566 |
| CXorf65 | 1.361895 | -2.22441 | 9.06E-05 | 0.000208918 |
| RNF222 | 1.346031 | -2.94392 | 9.08E-05 | 0.000209355 |
| TBC1D3E | 2.510828 | -2.82038 | 9.22E-05 | 0.000212394 |
| AC244197.3 | 1.112629 | 0.568295 | 9.25E-05 | 0.000213162 |
| KLHL35 | 1.188215 | -0.21262 | 9.26E-05 | 0.000213254 |
| KRTAP5-1 | 1.217794 | -1.27046 | 9.45E-05 | 0.000217421 |
| CFAP74 | 1.628656 | -1.41363 | 9.49E-05 | 0.000218315 |
| ADGRE3 | 1.283374 | -0.8862 | 9.51E-05 | 0.000218666 |
| XK | -1.33183 | 0.754336 | 9.53E-05 | 0.000218968 |
| SERPINA10 | -1.03583 | -2.45084 | 9.69E-05 | 0.000222482 |
| IL23R | 1.31556 | -2.61065 | 9.73E-05 | 0.000223334 |
| TRIM67 | 1.982917 | -0.19895 | 9.75E-05 | 0.000223872 |
| CT83 | 5.686534 | -2.82297 | 9.76E-05 | 0.000224083 |
| MPO | 1.40992 | -1.00373 | 9.77E-05 | 0.000224124 |
| ZNF716 | 3.8141 | -3.23043 | 9.84E-05 | 0.000225712 |
| CCL19 | -1.67539 | 2.012 | 9.98E-05 | 0.00022861 |
| MMP10 | 4.710094 | 0.072792 | 0.000100572 | 0.000230192 |
| KCNIP3 | 1.126569 | 5.457918 | 0.000101591 | 0.000232376 |
| FLJ22763 | -1.56301 | 1.066394 | 0.000101884 | 0.000233017 |
| RAB27B | 1.654838 | 2.231198 | 0.000102622 | 0.000234616 |
| C14orf180 | -1.72837 | -1.1233 | 0.000102848 | 0.000235072 |
| GCSAML | 1.549282 | -0.8552 | 0.000103075 | 0.00023556 |
| GGT2 | 1.429555 | 0.197021 | 0.000103249 | 0.000235897 |
| YBX2 | 1.45902 | 2.286126 | 0.000103496 | 0.000236402 |
| SIGLEC12 | 1.618774 | -1.55819 | 0.000103705 | 0.000236849 |
| EIF1AY | -1.64902 | 2.475995 | 0.000103782 | 0.000236965 |
| EPHA7 | 1.303983 | 5.781303 | 0.000105055 | 0.000239778 |
| SLCO1C1 | -1.00498 | -2.49928 | 0.000106197 | 0.000242354 |
| IL17RE | -1.04741 | 2.883205 | 0.000106577 | 0.000243102 |
| SOSTDC1 | 2.303108 | 7.35274 | 0.00010801 | 0.000246209 |
| OLFM2 | 1.266678 | 4.145785 | 0.000108435 | 0.000247084 |
| AQP8 | 1.397786 | -3.06618 | 0.000108992 | 0.000248259 |
| JAKMIP1 | 1.402093 | -0.5221 | 0.000110421 | 0.000251257 |
| EFS | -1.31462 | 2.023092 | 0.000111548 | 0.000253725 |
| FBLL1 | 1.699602 | 0.376945 | 0.000111911 | 0.000254462 |
| AMIGO3 | 1.158607 | -3.49223 | 0.000111914 | 0.000254462 |
| CT47B1 | 7.109397 | -1.2106 | 0.000112801 | 0.000256412 |
| KCNC2 | 2.528581 | -4.13766 | 0.000113197 | 0.000257214 |
| DUOX1 | 1.086636 | 2.348429 | 0.000113651 | 0.000258114 |
| NXPH3 | -1.03219 | 0.113764 | 0.000113815 | 0.000258455 |
| TSLP | 1.871333 | 1.62659 | 0.000115834 | 0.000262773 |
| ZNF888 | 1.016375 | 0.758784 | 0.00011623 | 0.000263571 |
| TMPRSS11A | 3.902558 | -1.37819 | 0.00011755 | 0.000266328 |
| SLC22A6 | -2.07594 | 7.084265 | 0.000117568 | 0.000266337 |
| MS4A2 | 1.586656 | 1.56695 | 0.00011782 | 0.000266771 |
| ZNF90 | 1.136252 | 2.218751 | 0.000117945 | 0.00026702 |
| GSG1L2 | 4.993641 | -2.46708 | 0.000118399 | 0.000267912 |
| SPATA21 | 2.435912 | -2.33715 | 0.000118467 | 0.000268032 |
| FAM81B | 1.450424 | 1.929033 | 0.000119575 | 0.000270472 |
| F10 | 1.817137 | 4.272332 | 0.000119893 | 0.000271089 |
| AC233992.2 | 1.517012 | -3.10827 | 0.000120702 | 0.000272815 |
| SLC9A9 | 1.002959 | 3.471492 | 0.000121616 | 0.000274775 |
| APOBEC1 | 3.280657 | -3.72438 | 0.000121666 | 0.000274853 |
| RBM46 | 1.976374 | -2.66323 | 0.00012181 | 0.00027511 |
| SERPINF1 | 1.512927 | 6.489829 | 0.000121999 | 0.000275503 |
| XG | -1.64597 | -1.77362 | 0.000122442 | 0.000276328 |
| ADAMTS2 | 1.151099 | 3.77849 | 0.000124334 | 0.00028035 |
| HS3ST3B1 | -1.29916 | 1.988849 | 0.000124582 | 0.000280875 |
| IFNG | 2.080304 | -2.5515 | 0.000125394 | 0.000282563 |
| UGT2B11 | 1.648761 | 0.728751 | 0.000127726 | 0.00028749 |
| ITIH1 | 2.808069 | -1.60902 | 0.000127894 | 0.000287797 |
| MMP13 | 5.128709 | -0.25786 | 0.000128232 | 0.000288413 |
| STX16-NPEPL1 | 1.181119 | 0.538786 | 0.000128982 | 0.00028999 |
| SERPINB5 | 4.29893 | 0.401808 | 0.000130578 | 0.000293468 |
| MAGEA10 | 5.857768 | -0.90942 | 0.000130695 | 0.000293696 |
| PTPRU | 1.213083 | 5.816045 | 0.000130785 | 0.00029386 |
| CDH22 | 2.201694 | 2.441473 | 0.000131165 | 0.000294567 |
| ZPLD1 | 2.462177 | 2.590641 | 0.000131379 | 0.000294973 |
| ARX | 3.457555 | 0.602976 | 0.000131729 | 0.000295647 |
| ADRA1B | -1.00105 | 1.154812 | 0.000132951 | 0.000298165 |
| CD3E | 1.219482 | 3.592344 | 0.000133107 | 0.000298478 |
| C1QTNF8 | 1.977174 | -3.41727 | 0.000133391 | 0.000299039 |
| SHISA3 | -1.38899 | 4.447149 | 0.000134014 | 0.00030036 |
| OR1N1 | 4.469113 | -2.58793 | 0.000134367 | 0.000301115 |
| KCNMA1 | 1.109735 | 4.344508 | 0.000135809 | 0.000304118 |
| F2RL3 | -1.08662 | 1.544814 | 0.000136496 | 0.00030558 |
| CLEC18A | -1.30356 | 1.425434 | 0.000137031 | 0.000306663 |
| IL31RA | 1.545351 | -1.39477 | 0.000137226 | 0.000307022 |
| GZMK | 1.521101 | 2.175351 | 0.000137568 | 0.000307709 |
| PSG1 | 2.907044 | -3.24463 | 0.000137593 | 0.000307717 |
| TMPRSS4 | -1.80346 | 3.961201 | 0.000137611 | 0.000307717 |
| ERVV-1 | 3.91827 | 1.668398 | 0.000138127 | 0.000308614 |
| FAM83A | 3.340573 | 0.866824 | 0.000138735 | 0.000309933 |
| UGT1A8 | 4.421848 | 0.933058 | 0.000140348 | 0.000313263 |
| HIST1H3H | 1.669951 | 0.244965 | 0.000142121 | 0.000316944 |
| NKX2-5 | 3.459552 | -3.06537 | 0.000143098 | 0.000319004 |
| CALML3 | -2.03108 | 2.841596 | 0.000143191 | 0.00031917 |
| CACNA1G | 1.388511 | -2.1966 | 0.000143968 | 0.000320743 |
| MAGEA4 | 4.707149 | -3.28565 | 0.000144348 | 0.00032155 |
| CADM2 | -1.63727 | -1.96963 | 0.000144676 | 0.000322241 |
| XCL2 | 1.296074 | -1.4459 | 0.000145247 | 0.000323431 |
| SYNGR3 | 1.311403 | 2.536967 | 0.00014565 | 0.000324208 |
| PSG9 | 2.91523 | -1.3741 | 0.000146113 | 0.000325198 |
| ADGRF2 | 1.869165 | 0.353068 | 0.000149324 | 0.000331849 |
| EBF4 | 1.061792 | 4.475628 | 0.000152166 | 0.000337878 |
| SAP25 | 1.221894 | -1.81451 | 0.000152169 | 0.000337878 |
| FTCD | -1.2993 | 6.029847 | 0.000152418 | 0.000338347 |
| TBX20 | 3.528753 | -3.33795 | 0.000153654 | 0.000340922 |
| SHISA9 | 1.109016 | 3.967544 | 0.000153958 | 0.000341513 |
| MANSC4 | 1.748898 | -1.78908 | 0.000154423 | 0.000342459 |
| NRXN3 | 1.638362 | 3.683905 | 0.000154517 | 0.000342626 |
| PPP4R3CP | 4.834767 | -3.46312 | 0.000154546 | 0.000342647 |
| LAMA1 | 1.095808 | 5.654972 | 0.000155543 | 0.000344729 |
| HTR1B | -1.33555 | -2.36374 | 0.000160351 | 0.000354945 |
| RHOXF1 | 1.585923 | -1.07769 | 0.000161284 | 0.000356877 |
| SYCP2L | 1.146363 | -0.28666 | 0.000161617 | 0.000357571 |
| LINGO2 | -1.44576 | -0.87863 | 0.000162378 | 0.000359122 |
| S100A2 | -1.18752 | 4.914111 | 0.000162742 | 0.000359882 |
| RGS13 | 1.425354 | -1.35664 | 0.000163105 | 0.000360595 |
| SPTA1 | 1.456766 | -2.39381 | 0.000164263 | 0.000363021 |
| SCARA3 | 1.259151 | 6.654384 | 0.000164573 | 0.000363481 |
| KRT31 | 2.06163 | -2.32262 | 0.000165455 | 0.000365205 |
| C4orf50 | 1.968889 | -2.48163 | 0.000165864 | 0.000366063 |
| ADGRE2 | 1.087805 | 2.26258 | 0.000165932 | 0.000366168 |
| PMP2 | -2.03432 | -2.77075 | 0.000166351 | 0.000366955 |
| SP9 | 4.162576 | -3.52823 | 0.000166756 | 0.00036776 |
| GRIK1 | 1.406118 | -0.93898 | 0.000167279 | 0.000368822 |
| CALML5 | 7.578054 | 2.599207 | 0.000167971 | 0.000370211 |
| S100A7 | 5.79104 | 2.120772 | 0.000168135 | 0.000370481 |
| FOXA1 | 3.203845 | 0.916454 | 0.000168343 | 0.000370848 |
| PIANP | 1.557705 | 0.984279 | 0.000170783 | 0.000376085 |
| HSD17B6 | -1.11569 | -0.5885 | 0.000171064 | 0.000376657 |
| TTLL10 | -1.01172 | -1.97787 | 0.000171196 | 0.000376902 |
| PNPLA5 | 3.247322 | -3.41838 | 0.000172287 | 0.00037899 |
| ACVR1C | -1.20032 | -0.37425 | 0.000172293 | 0.00037899 |
| MAP2 | 1.189508 | 4.764878 | 0.000173231 | 0.000381006 |
| POPDC3 | 2.678395 | -0.64416 | 0.000174221 | 0.000382947 |
| TEX45 | 1.177647 | -1.15197 | 0.000175046 | 0.000384572 |
| GUCA1C | -1.41395 | -2.18449 | 0.00017615 | 0.000386809 |
| SYN1 | 1.091267 | 1.162572 | 0.000177604 | 0.000389809 |
| C15orf59 | -1.04 | 3.494842 | 0.000177974 | 0.000390526 |
| TM4SF5 | -1.29162 | 2.299376 | 0.000179781 | 0.000394201 |
| IL20RA | 1.887545 | 1.709894 | 0.000181343 | 0.000397528 |
| RD3 | 1.857486 | -2.86691 | 0.000181652 | 0.000398108 |
| MYO16 | -1.03301 | -1.04645 | 0.000182311 | 0.000399455 |
| KCTD8 | -1.60315 | -0.73662 | 0.000183019 | 0.00040081 |
| RTL1 | 5.12337 | -1.77399 | 0.000183973 | 0.000402653 |
| EDARADD | -1.1797 | 0.196364 | 0.000185252 | 0.000405206 |
| RASL10A | 1.169665 | -0.38705 | 0.000185487 | 0.000405669 |
| TMEM155 | 3.104836 | -1.60556 | 0.000186176 | 0.000407026 |
| FN1 | 1.601381 | 9.785168 | 0.000186209 | 0.000407049 |
| RAB25 | -1.61602 | 4.014269 | 0.000186524 | 0.000407637 |
| PRSS50 | 1.626279 | 0.518609 | 0.000187216 | 0.000409052 |
| DHRS2 | 3.243515 | 4.623225 | 0.000188976 | 0.000412392 |
| ENPP3 | 1.30697 | 4.742848 | 0.000190067 | 0.000414471 |
| KRTAP16-1 | 1.781764 | -3.7924 | 0.000192615 | 0.000419771 |
| MAGEA12 | 5.305771 | -1.86869 | 0.000192916 | 0.000420275 |
| FFAR2 | 1.26787 | -1.32887 | 0.000194977 | 0.000424505 |
| CCR3 | 1.318888 | -2.16042 | 0.000195584 | 0.000425671 |
| TSPAN5 | -1.27885 | 3.101803 | 0.000196247 | 0.000427011 |
| PRG2 | 1.761188 | -3.57323 | 0.000196315 | 0.000427107 |
| CCL4L2 | 1.330586 | 2.133118 | 0.000197792 | 0.000430112 |
| PAGE5 | 3.127836 | -1.12018 | 0.000198159 | 0.000430858 |
| KISS1R | 2.031841 | -1.77337 | 0.000201004 | 0.000436884 |
| IGSF9 | -1.18405 | 0.715462 | 0.000201622 | 0.000438067 |
| FPR1 | 1.034309 | 2.848862 | 0.000201844 | 0.000438337 |
| KRT9 | 2.511417 | -3.88588 | 0.000203059 | 0.000440921 |
| ACKR4 | 1.165883 | 1.121955 | 0.000203229 | 0.000441185 |
| A4GNT | 1.348564 | 0.15345 | 0.000204626 | 0.000444 |
| SPTBN5 | 1.199805 | 3.302542 | 0.000207082 | 0.000449168 |
| RAB3B | 1.352523 | 3.865347 | 0.000210093 | 0.000455256 |
| CTSE | 2.899475 | 3.118116 | 0.000210331 | 0.000455718 |
| GRHL3 | 1.71215 | 1.648231 | 0.000210534 | 0.000456102 |
| KRT75 | 3.95494 | -3.17993 | 0.00021135 | 0.000457592 |
| SPATA46 | -1.14287 | -2.92794 | 0.000214766 | 0.000464764 |
| DUX4 | 3.375878 | -3.97713 | 0.000216749 | 0.000468829 |
| OR2A4 | 2.309038 | -3.9528 | 0.000218254 | 0.000471914 |
| LAD1 | -1.14633 | 5.492047 | 0.000218701 | 0.000472821 |
| NFE4 | 1.680976 | -2.93895 | 0.000219497 | 0.00047437 |
| AADACL4 | -1.73773 | -3.50735 | 0.000221425 | 0.000478307 |
| AC024940.1 | 1.555986 | -2.71833 | 0.00022172 | 0.000478887 |
| GJB6 | 3.948062 | 0.863383 | 0.000227581 | 0.000490834 |
| ASB11 | -1.20327 | -3.95068 | 0.000230281 | 0.000496179 |
| OR2T10 | -1.46378 | -0.7254 | 0.000230521 | 0.000496636 |
| MOV10L1 | -1.02067 | -2.00617 | 0.000234459 | 0.000504697 |
| POU3F4 | -2.41739 | -0.55527 | 0.000235582 | 0.000507053 |
| CABP4 | 1.596476 | 1.534549 | 0.000237878 | 0.000511564 |
| CYP7A1 | -1.11156 | -3.8175 | 0.00023806 | 0.000511893 |
| WNT3A | -1.40181 | -2.49626 | 0.000242551 | 0.000520925 |
| DOK2 | 1.021522 | 2.582223 | 0.000242581 | 0.000520927 |
| SIK1 | -1.41475 | 1.269557 | 0.000242804 | 0.000521343 |
| TLX2 | 1.404731 | -3.96213 | 0.000243675 | 0.000523024 |
| ISL1 | 4.111327 | -2.40859 | 0.000244043 | 0.000523725 |
| CD247 | 1.012929 | 1.988015 | 0.000244179 | 0.000523855 |
| CSMD1 | -1.32284 | 1.442863 | 0.000245195 | 0.000525846 |
| ELN | -1.01875 | 4.405082 | 0.000245432 | 0.00052629 |
| MSMB | 3.171818 | -1.53187 | 0.000249171 | 0.00053386 |
| TLX1 | 2.744111 | -3.27598 | 0.000250441 | 0.000536389 |
| KCNB2 | 2.022654 | -1.00492 | 0.000252614 | 0.000540675 |
| CCR9 | 1.190916 | 0.346837 | 0.000252623 | 0.000540675 |
| CCER2 | 1.081943 | -1.03713 | 0.000254325 | 0.000543928 |
| HOXD11 | -1.46176 | 0.36911 | 0.000254391 | 0.000544004 |
| GUCA1A | 3.447775 | -2.59277 | 0.000257517 | 0.000550228 |
| UNC13A | 1.59673 | 0.877739 | 0.000258452 | 0.000552094 |
| NGB | 2.189947 | -3.23838 | 0.000258802 | 0.000552776 |
| DOK5 | 1.012236 | 3.72161 | 0.00026416 | 0.000563413 |
| RNF151 | 1.350717 | -3.67377 | 0.000266451 | 0.000568029 |
| KLRC1 | 1.404358 | -1.91049 | 0.000266516 | 0.000568101 |
| CDH15 | -1.21001 | -2.39858 | 0.000266881 | 0.000568676 |
| EGR4 | -1.42546 | -2.53612 | 0.000268118 | 0.000571039 |
| GRIK3 | 2.551896 | 1.337447 | 0.000269086 | 0.000572828 |
| LY6K | -1.33375 | -0.73933 | 0.000270332 | 0.000575138 |
| ABCB11 | 2.708259 | -3.00005 | 0.000272407 | 0.000579139 |
| MEDAG | -1.00841 | 1.543583 | 0.000272606 | 0.000579495 |
| SLC18A3 | 3.44818 | -2.43592 | 0.000272703 | 0.00057957 |
| HTR6 | 2.510607 | -2.73362 | 0.000272843 | 0.000579724 |
| GOLGA6L4 | 1.240332 | -2.07506 | 0.00027357 | 0.000581198 |
| LEFTY1 | 2.472986 | 3.790758 | 0.000274775 | 0.00058348 |
| MMP1 | 2.546847 | 2.407426 | 0.000275733 | 0.000585307 |
| DMRT1 | 3.229253 | -2.80322 | 0.000280171 | 0.000594165 |
| LHCGR | 3.153471 | 0.281544 | 0.000281653 | 0.000597024 |
| C6orf15 | 2.556659 | -3.99472 | 0.000281848 | 0.000597367 |
| PLA2G4D | 2.540652 | -2.30549 | 0.00029436 | 0.000622633 |
| RAX | 4.63427 | -2.19864 | 0.000295171 | 0.0006242 |
| LRRC31 | 1.307875 | -0.27412 | 0.000295778 | 0.000625411 |
| PCDHGA1 | 1.153634 | 0.56645 | 0.000299371 | 0.000632634 |
| CHL1 | 1.189313 | 7.154549 | 0.000302361 | 0.000638651 |
| FAM216B | -1.76865 | -4.10402 | 0.000303342 | 0.000640572 |
| PTGER2 | 1.079222 | 3.0517 | 0.000306337 | 0.000646362 |
| HLA-DQA2 | 1.530021 | 4.424574 | 0.000309149 | 0.000651757 |
| GIPR | 1.208828 | 1.93929 | 0.000312353 | 0.000658076 |
| ZNF99 | -1.34878 | -1.87835 | 0.000312366 | 0.000658076 |
| ANKS4B | -1.13669 | 3.927642 | 0.000312493 | 0.000658264 |
| CHRM1 | -1.50629 | -1.52879 | 0.000312679 | 0.00065858 |
| PRSS33 | 4.145006 | 0.116821 | 0.000312779 | 0.000658713 |
| TMEM40 | 2.663301 | -0.19309 | 0.000315664 | 0.000664476 |
| EDAR | 1.415593 | 2.596725 | 0.00032398 | 0.000681101 |
| FDCSP | 4.559928 | 0.91986 | 0.00032602 | 0.000685308 |
| NRXN2 | 1.004673 | 4.768039 | 0.00032976 | 0.000692845 |
| AC245041.1 | 2.125121 | -4.16104 | 0.000332933 | 0.000698905 |
| WISP2 | 1.599959 | 1.040075 | 0.000337158 | 0.000707061 |
| NLRP10 | -1.39684 | -4.16667 | 0.000340796 | 0.000714187 |
| POU4F1 | 2.464846 | -2.9838 | 0.000341048 | 0.000714633 |
| GLB1L3 | -1.60358 | -0.96501 | 0.000341148 | 0.000714758 |
| NPPB | 3.122498 | -3.27037 | 0.000343116 | 0.000718408 |
| ZFHX4 | 1.590759 | 2.832587 | 0.000344074 | 0.000720213 |
| GPX2 | 2.577881 | 3.761202 | 0.000344918 | 0.000721812 |
| C3orf80 | -1.00825 | -1.50768 | 0.00034747 | 0.000726811 |
| PTHLH | 1.583461 | 0.312109 | 0.000351773 | 0.000735384 |
| PCBP3 | -1.09033 | 1.228043 | 0.000354102 | 0.000739648 |
| KCNJ6 | -1.36017 | -2.01183 | 0.000356498 | 0.000744393 |
| RBP5 | -1.07641 | 7.312384 | 0.000356956 | 0.000745175 |
| TRIM72 | 2.296353 | -0.24956 | 0.000360894 | 0.000753044 |
| FOXD1 | -1.23964 | 0.159221 | 0.000366917 | 0.000764719 |
| GNGT1 | 1.74111 | -0.35335 | 0.000373005 | 0.000776595 |
| PODNL1 | 1.661059 | 0.858298 | 0.000374107 | 0.000778707 |
| MAGEA11 | 5.074061 | -2.57481 | 0.000376262 | 0.00078283 |
| GH2 | 3.812593 | -3.98678 | 0.000382253 | 0.000794464 |
| TSNAX-DISC1 | 1.334531 | -3.98874 | 0.000385058 | 0.000800106 |
| SLX1A | 1.333789 | -3.8814 | 0.000385935 | 0.000801836 |
| RFPL4A | 1.300604 | -3.18966 | 0.000395933 | 0.000821154 |
| SYT3 | 1.333696 | 1.536096 | 0.000395967 | 0.000821154 |
| PRSS38 | 3.910478 | -3.51564 | 0.00039733 | 0.000823694 |
| CDH11 | -1.04389 | 3.592312 | 0.000400822 | 0.000830549 |
| TBX5 | 3.088015 | -3.10669 | 0.000405552 | 0.000840156 |
| ADAMTS20 | 2.785716 | -3.47174 | 0.000406798 | 0.000842542 |
| SSC5D | 1.183662 | 4.086406 | 0.000410583 | 0.000849982 |
| IL10 | 1.135798 | -1.00581 | 0.000410628 | 0.000849982 |
| AC005154.6 | 1.577043 | -3.21414 | 0.000414955 | 0.000858542 |
| OLR1 | 1.023177 | 4.657557 | 0.000417551 | 0.000863644 |
| SH2D1A | 1.13662 | 0.643345 | 0.000427133 | 0.000882311 |
| SPIC | 1.824783 | -3.56419 | 0.000430584 | 0.000888721 |
| FAM19A3 | 1.147416 | -2.49745 | 0.00043153 | 0.000890382 |
| PLCB4 | 1.302641 | 4.915284 | 0.000433756 | 0.000894546 |
| TAT | 1.014841 | -1.04395 | 0.000435438 | 0.000897913 |
| NPIPA5 | 1.17758 | 0.348923 | 0.000441374 | 0.00090921 |
| DMRTA2 | 1.697119 | -3.47974 | 0.000444462 | 0.00091536 |
| TNR | 3.338187 | 1.151631 | 0.00044729 | 0.000920549 |
| GLP1R | 2.344911 | -0.12158 | 0.000447621 | 0.000921126 |
| RADIL | 1.329993 | 1.189446 | 0.000448193 | 0.000922195 |
| NOBOX | 2.416323 | -3.51349 | 0.000451082 | 0.000927714 |
| CCK | -1.76467 | -4.01118 | 0.000454576 | 0.000934256 |
| ISL2 | 1.919618 | -3.28879 | 0.000454959 | 0.000934936 |
| ZNF114 | 2.081785 | 1.111944 | 0.000456732 | 0.000938148 |
| XCL1 | 1.154448 | -1.03481 | 0.000465854 | 0.000955898 |
| PLP1 | -1.38106 | -1.15494 | 0.000468652 | 0.000961308 |
| DIRAS2 | 1.209168 | 2.690106 | 0.000468825 | 0.000961554 |
| DUSP4 | 1.51456 | 3.298753 | 0.000480029 | 0.000983406 |
| DMBX1 | 2.372384 | -3.46676 | 0.000481985 | 0.000986961 |
| GRIA2 | -1.58331 | -1.95377 | 0.00048633 | 0.000995289 |
| MRGPRE | 2.543293 | -0.96806 | 0.000501976 | 0.001025316 |
| PRDM13 | 2.998626 | -3.96318 | 0.000505537 | 0.001032236 |
| CD27 | 1.228227 | 1.918487 | 0.000514915 | 0.001050843 |
| CDCP1 | 1.120125 | 5.675859 | 0.000515619 | 0.001052102 |
| OTOS | 2.538189 | -2.1651 | 0.000515807 | 0.001052365 |
| FZD9 | 1.158006 | -1.03988 | 0.000517437 | 0.001055331 |
| CHRDL1 | -1.37712 | 3.622722 | 0.000521119 | 0.001062356 |
| ASIC5 | -1.89717 | -3.93754 | 0.000527117 | 0.001073851 |
| CNNM1 | 1.117142 | 3.617182 | 0.000528332 | 0.001076202 |
| FUT7 | 1.008395 | -1.38976 | 0.000530855 | 0.001080604 |
| SRD5A2 | -1.37357 | -1.29845 | 0.000533489 | 0.001085595 |
| MCIDAS | 1.198499 | -1.119 | 0.000540202 | 0.001098632 |
| NPIPB7 | 1.228721 | -3.34652 | 0.000540983 | 0.001100094 |
| IL2RB | 1.0205 | 3.036669 | 0.000541404 | 0.001100826 |
| AMHR2 | 1.36659 | -2.64315 | 0.000542929 | 0.001103676 |
| TAC3 | 2.505065 | -2.71497 | 0.000547401 | 0.001112515 |
| CCL24 | 1.737327 | -1.50029 | 0.000548501 | 0.001114496 |
| NKX6-1 | 3.603935 | -0.13927 | 0.000555399 | 0.001127233 |
| CRLF2 | 1.212756 | -2.82695 | 0.000555577 | 0.001127466 |
| NETO2 | 1.294554 | 3.15449 | 0.000565783 | 0.001146749 |
| SLC26A3 | 1.787015 | -1.43487 | 0.000568749 | 0.001152109 |
| LGALS14 | 4.332041 | -3.50189 | 0.000574297 | 0.001163083 |
| PAPPA2 | -1.8523 | 5.295555 | 0.000574968 | 0.001164311 |
| MUC1 | -1.02289 | 7.499393 | 0.000584371 | 0.001182416 |
| EDIL3 | 1.238932 | 6.237976 | 0.000593158 | 0.001199245 |
| PLPPR4 | -1.00921 | -0.37855 | 0.000600535 | 0.001212791 |
| SPRR2D | -1.70541 | -3.40295 | 0.000600653 | 0.001212892 |
| C1orf87 | 2.272317 | -0.48871 | 0.00060942 | 0.001229341 |
| CCL3L1 | 1.280082 | 1.573446 | 0.000616887 | 0.001242869 |
| RLBP1 | 2.217915 | -4.09092 | 0.000620104 | 0.001248812 |
| STUM | -1.2691 | 3.782348 | 0.000631178 | 0.001270232 |
| ZNF705A | 1.591412 | -4.04334 | 0.00063168 | 0.0012711 |
| KLRG2 | -1.05271 | 2.23205 | 0.000633309 | 0.001274234 |
| ALX4 | -1.29261 | -2.08248 | 0.000636613 | 0.001280594 |
| PAX5 | -1.40859 | -0.59208 | 0.000636984 | 0.001281196 |
| COL1A1 | 1.31069 | 8.301896 | 0.000637453 | 0.001281708 |
| SLC15A1 | 1.250851 | 4.726448 | 0.000639072 | 0.001284675 |
| ADAD2 | -1.05723 | -2.41517 | 0.000641997 | 0.00128983 |
| ASB18 | 1.872503 | -2.38617 | 0.000644228 | 0.001294022 |
| ZNF541 | 1.179725 | 2.620965 | 0.000649556 | 0.001304285 |
| PCDHA8 | 1.203708 | -2.06638 | 0.000655578 | 0.001315934 |
| PF4 | 1.269324 | -1.89907 | 0.000660804 | 0.001326128 |
| CASP14 | 6.15037 | 4.864727 | 0.000672889 | 0.001349019 |
| CFP | 1.040584 | 1.807462 | 0.000682208 | 0.00136643 |
| FAM71F1 | -1.18948 | -3.60279 | 0.000685655 | 0.001372459 |
| GPHA2 | 1.528797 | -1.58113 | 0.000686215 | 0.001373275 |
| WNT5B | 1.157164 | 2.57533 | 0.000686215 | 0.001373275 |
| SSX1 | 4.148654 | -2.33729 | 0.00069135 | 0.001382777 |
| MAJIN | 1.29605 | 0.897357 | 0.000693208 | 0.001386184 |
| KCNC1 | 1.184522 | -2.32509 | 0.000693875 | 0.001387362 |
| SRPX | -1.03227 | 1.558369 | 0.000694951 | 0.001388893 |
| ZBED2 | 1.696228 | 1.167097 | 0.000698109 | 0.001394893 |
| KCNA7 | 1.348455 | -3.91294 | 0.000701732 | 0.001401819 |
| GAGE12J | 4.157443 | -3.82217 | 0.000703471 | 0.001405136 |
| KRTAP2-3 | 2.063628 | -1.10302 | 0.000704614 | 0.001407263 |
| CSAG1 | 4.106335 | -2.28745 | 0.000707349 | 0.001412252 |
| IL36B | 2.767363 | -3.58077 | 0.000708578 | 0.001414391 |
| PCDHA7 | 1.348226 | -0.28589 | 0.00071356 | 0.001423542 |
| ZNF648 | 1.887815 | -2.68333 | 0.000722511 | 0.001440917 |
| FOXA3 | -1.16998 | 0.581167 | 0.000734643 | 0.001462831 |
| FANCD2OS | 1.478494 | -4.05116 | 0.000739359 | 0.001471238 |
| RSPH10B2 | 1.046811 | -3.36839 | 0.000740706 | 0.001473756 |
| DLX2 | 2.103418 | -3.02781 | 0.000742841 | 0.00147784 |
| MAGEA8 | 2.010333 | -2.82973 | 0.000754516 | 0.001499233 |
| DLL1 | -1.05457 | 2.865289 | 0.000758235 | 0.001505621 |
| HLA-DOB | 1.101747 | 1.36792 | 0.000769017 | 0.001524662 |
| LRRC74B | 1.711563 | -2.85192 | 0.000772893 | 0.001532176 |
| P4HA3 | 1.169317 | 0.536107 | 0.00077348 | 0.001533001 |
| FOXD4L4 | 1.484872 | -3.958 | 0.000780727 | 0.001546509 |
| TMEM88B | -1.56554 | -4.11285 | 0.000798917 | 0.001579568 |
| GDA | 1.298809 | 7.616965 | 0.00080463 | 0.00158946 |
| PRSS37 | 1.264325 | -3.01357 | 0.0008062 | 0.001592385 |
| NPFFR2 | 1.919975 | -0.79632 | 0.000807106 | 0.001593999 |
| ACTC1 | -1.3151 | 0.399555 | 0.000814677 | 0.001608595 |
| KRTAP9-6 | 4.353779 | -3.04864 | 0.000816463 | 0.00161159 |
| AC117457.1 | -1.36462 | -3.66262 | 0.000823784 | 0.001624607 |
| CLEC4M | -1.41658 | -3.49151 | 0.0008316 | 0.001637857 |
| INHBC | 1.224915 | -0.50105 | 0.000832152 | 0.001638763 |
| CARD17 | 1.560265 | -4.00686 | 0.000846397 | 0.00166535 |
| SIX6 | 4.565037 | -2.7856 | 0.000863117 | 0.00169657 |
| IL2RA | 1.086434 | 0.91348 | 0.000863986 | 0.001698092 |
| FOXL2NB | 2.525478 | -3.34379 | 0.000864274 | 0.00169847 |
| C1orf158 | 2.733867 | -1.79721 | 0.000867988 | 0.001705208 |
| MAGEB1 | 4.559932 | -3.36425 | 0.000868259 | 0.001705366 |
| HABP2 | 1.61513 | 5.771939 | 0.000871006 | 0.001710198 |
| KRT16 | 2.868126 | 2.274178 | 0.000877836 | 0.001722665 |
| SFTPA2 | -1.41306 | -1.21765 | 0.000880858 | 0.001728026 |
| DDX53 | 3.030787 | -4.07761 | 0.000883106 | 0.001732057 |
| RIPPLY1 | 2.556005 | 3.287423 | 0.000884581 | 0.001734379 |
| FOXR2 | 4.403212 | -3.38243 | 0.000892141 | 0.001748628 |
| SIGLEC15 | 1.315631 | 0.805572 | 0.00089956 | 0.001762591 |
| CXCL3 | 1.092131 | 0.692759 | 0.000931366 | 0.001821413 |
| BTG4 | 1.66919 | -4.02754 | 0.000931412 | 0.001821413 |
| OR2B6 | 1.576109 | -3.98069 | 0.000959236 | 0.001872142 |
| CD164L2 | 1.302065 | -0.99342 | 0.000960425 | 0.001874258 |
| DSC3 | 1.988249 | -0.61111 | 0.000961138 | 0.001875241 |
| PASD1 | 3.950021 | -3.26104 | 0.00096855 | 0.001888466 |
| RBBP8NL | -1.96408 | -0.50449 | 0.000984326 | 0.001916931 |
| CACNG4 | -1.17587 | -1.34842 | 0.000993705 | 0.001933301 |
| SLC1A2 | -1.11325 | -1.41103 | 0.001004586 | 0.001953197 |
| MUC17 | 2.500177 | -2.28263 | 0.001005573 | 0.001954904 |
| KCNK15 | 1.246252 | 2.028253 | 0.001010623 | 0.001963443 |
| TMEM255A | 1.472824 | 3.525298 | 0.001013179 | 0.001967982 |
| NEUROG3 | 1.916273 | -3.46489 | 0.001028389 | 0.001996225 |
| TP53AIP1 | -1.29285 | -1.37364 | 0.001030816 | 0.002000503 |
| UGT1A10 | 4.232078 | 0.624154 | 0.00103739 | 0.002012389 |
| DIO3 | -1.41002 | -2.47752 | 0.001050249 | 0.002035789 |
| ZNF683 | 1.069591 | 0.086547 | 0.001052523 | 0.002039755 |
| TMPRSS12 | -1.10034 | -3.32108 | 0.001055609 | 0.00204507 |
| MYH13 | 2.316456 | -3.18662 | 0.001056493 | 0.002046563 |
| PRIMA1 | -1.2868 | 0.671624 | 0.001060049 | 0.002053006 |
| OR2H2 | 1.786587 | -3.9467 | 0.001081052 | 0.002089839 |
| NXPE4 | 2.027026 | -0.00356 | 0.001084528 | 0.002095777 |
| PGA5 | 1.465249 | -0.68665 | 0.001084592 | 0.002095777 |
| SPINK2 | 2.082934 | -0.69119 | 0.001092156 | 0.002109482 |
| BEND4 | -1.11081 | -2.94847 | 0.001100632 | 0.002125395 |
| PLA2G2F | 4.563038 | 1.324115 | 0.001128859 | 0.002177319 |
| FOXJ1 | 1.28107 | 4.006918 | 0.0011532 | 0.002220679 |
| C10orf142 | 1.525479 | -1.69706 | 0.001154745 | 0.002223415 |
| NRN1L | 1.394721 | -4.09113 | 0.001159933 | 0.002232924 |
| CHRND | 1.707096 | -3.30575 | 0.001160436 | 0.002233652 |
| HIST1H1D | -1.07786 | -3.29734 | 0.001161854 | 0.00223614 |
| GCG | 4.65281 | -2.12956 | 0.001174595 | 0.002259205 |
| CT62 | -1.39296 | -3.77926 | 0.001177576 | 0.002264209 |
| PACSIN1 | 1.014489 | 1.94395 | 0.00118066 | 0.002269407 |
| PNLIPRP3 | 4.753107 | -0.53591 | 0.001183606 | 0.002274826 |
| UGT1A3 | 2.188201 | -2.98532 | 0.001187391 | 0.002281365 |
| UNC5D | 1.206997 | 4.067993 | 0.001200231 | 0.002304552 |
| HBM | 2.235362 | -3.79604 | 0.001209657 | 0.002321655 |
| FAM196B | -1.29118 | -1.21616 | 0.001212204 | 0.002325944 |
| A2ML1 | 1.644731 | -0.75193 | 0.001212384 | 0.002325944 |
| ADA2 | 1.036259 | 6.763016 | 0.001233502 | 0.002364378 |
| CSF2 | 1.319872 | -3.81797 | 0.001254345 | 0.002402528 |
| NDUFA4L2 | 1.492118 | 4.832609 | 0.001259694 | 0.002411483 |
| KRT79 | 1.549277 | -3.69678 | 0.001269394 | 0.002428754 |
| FAM83C | 2.630737 | -1.09187 | 0.001272281 | 0.002434018 |
| KRTAP5-10 | 1.344646 | -1.69829 | 0.001276158 | 0.002440653 |
| C2CD4A | 1.70359 | 3.273147 | 0.001278708 | 0.002445268 |
| SCGB1A1 | -1.16495 | -3.95949 | 0.001285592 | 0.00245712 |
| CCDC144A | 1.895793 | -0.38013 | 0.001288704 | 0.002462542 |
| PROK1 | -1.0459 | -3.40156 | 0.001296759 | 0.002477404 |
| MS4A15 | 3.088297 | -3.03807 | 0.001328948 | 0.002534032 |
| FABP2 | -1.33334 | -2.91991 | 0.001347169 | 0.002566863 |
| SLAMF7 | 1.067071 | 2.979428 | 0.001362761 | 0.002594361 |
| WFDC10B | 1.514378 | -3.86852 | 0.001363447 | 0.002595391 |
| CACNG6 | 2.449515 | -1.89026 | 0.001370711 | 0.00260811 |
| SNTG2 | 1.350084 | -0.40425 | 0.001378289 | 0.002621137 |
| KLHL1 | -1.45582 | -2.60503 | 0.0013806 | 0.002625031 |
| TCEAL6 | -1.60933 | -2.63175 | 0.001382525 | 0.002628354 |
| C1orf141 | -1.07741 | -3.93276 | 0.00139295 | 0.00264733 |
| CNGA3 | -1.3453 | -3.46804 | 0.001396172 | 0.002652889 |
| B3GAT1 | 1.267013 | 3.106529 | 0.0013995 | 0.002658368 |
| C8orf74 | 2.396113 | -3.47883 | 0.001399942 | 0.002658925 |
| GRM4 | 1.986825 | -2.08421 | 0.001416168 | 0.002687134 |
| WBSCR17 | -1.54373 | 2.195527 | 0.00141967 | 0.002693251 |
| CDHR4 | 1.332709 | -1.80065 | 0.001424747 | 0.002702309 |
| NRSN1 | 1.142591 | -2.48378 | 0.001428923 | 0.002709251 |
| PCDHA2 | 1.196637 | 0.043034 | 0.001459612 | 0.002763753 |
| DPPA2 | 1.947673 | -0.96448 | 0.001468254 | 0.002779527 |
| NPPC | 1.786486 | -0.86202 | 0.001470618 | 0.002783413 |
| RTP2 | 2.54248 | -3.27778 | 0.001475647 | 0.002791162 |
| GOLGA8M | 1.076731 | -0.83115 | 0.001488537 | 0.00281348 |
| GPR35 | 1.005911 | 2.826811 | 0.001488546 | 0.00281348 |
| KCNK17 | 1.184598 | -2.44118 | 0.001488965 | 0.002813975 |
| HSD3B2 | -1.10345 | -1.63238 | 0.001493085 | 0.002821164 |
| CSPG5 | 1.023419 | -0.6622 | 0.001496515 | 0.002827048 |
| TBC1D3D | 2.811276 | -2.31188 | 0.0014972 | 0.002827795 |
| GIF | 1.330652 | -3.19549 | 0.001499668 | 0.002832109 |
| GAD2 | 2.422634 | -3.49427 | 0.001502454 | 0.002836173 |
| LIN28B | 3.439449 | -3.93006 | 0.001525276 | 0.002876524 |
| PAX7 | 2.7693 | -3.85913 | 0.001526562 | 0.002878344 |
| CDX2 | 2.338984 | -3.56019 | 0.00153056 | 0.002885576 |
| ELMOD1 | -1.26149 | 0.585672 | 0.001535994 | 0.002894602 |
| CACNA1I | 1.148245 | -1.63307 | 0.001539765 | 0.002901403 |
| PHGR1 | 3.018667 | -3.55792 | 0.001541421 | 0.002904218 |
| AC005324.3 | 1.391473 | -3.45439 | 0.001541743 | 0.002904518 |
| CYP11A1 | 1.436056 | 1.331334 | 0.001556267 | 0.00293003 |
| DUSP13 | 1.985491 | -2.73849 | 0.001579695 | 0.002970078 |
| CFHR1 | -1.17084 | -1.94144 | 0.001595838 | 0.002997596 |
| GGTLC3 | 1.478051 | -3.24858 | 0.001602616 | 0.003009381 |
| KHDC1L | 1.396875 | -4.03603 | 0.001628988 | 0.003056017 |
| CNGB1 | 1.276945 | -1.39851 | 0.001633395 | 0.003063963 |
| ST6GAL2 | 1.332535 | 2.249806 | 0.00163737 | 0.003070777 |
| LEFTY2 | -1.34656 | -1.40384 | 0.001645902 | 0.003085485 |
| NOS1 | -1.65053 | 3.089595 | 0.001656848 | 0.00310503 |
| R3HDML | -1.39081 | -3.70229 | 0.001657662 | 0.003106229 |
| CDRT1 | 1.01262 | -2.67002 | 0.001689735 | 0.003162028 |
| LECT2 | 1.208027 | -1.62261 | 0.001702449 | 0.003184154 |
| PRSS3 | -1.52225 | -0.18686 | 0.001705597 | 0.003189376 |
| PCDH20 | 1.37492 | -1.3543 | 0.00171848 | 0.003211788 |
| FRG2B | -2.06572 | -2.77958 | 0.001732406 | 0.003236278 |
| NAT8 | -1.05977 | 7.995738 | 0.001732487 | 0.003236278 |
| KRT4 | -1.55649 | -0.95294 | 0.001741727 | 0.003250824 |
| PPBP | 1.65978 | -1.0067 | 0.001772123 | 0.003302736 |
| LAMP5 | 2.283501 | 3.091187 | 0.001782113 | 0.003320317 |
| KRTAP5-5 | 1.901788 | -3.85497 | 0.00180863 | 0.003364818 |
| EFNB3 | 1.264315 | 3.168818 | 0.001821956 | 0.003387499 |
| CLEC4D | 1.043602 | -2.28145 | 0.001822779 | 0.003387976 |
| NETO1 | 1.827634 | -1.47961 | 0.001832462 | 0.003404556 |
| CXCL13 | 2.236289 | 1.614172 | 0.001833045 | 0.003405286 |
| CHRNG | 1.046727 | -3.34474 | 0.001868321 | 0.00346758 |
| CEACAM20 | 1.386948 | -4.06757 | 0.001870062 | 0.003470452 |
| BECN2 | 2.700819 | -4.02576 | 0.001926239 | 0.003570634 |
| SEC14L3 | 1.620016 | -4.0307 | 0.001928339 | 0.003573785 |
| TIGIT | 1.127806 | 0.96991 | 0.001932995 | 0.003580932 |
| MLXIPL | 1.027291 | 5.657179 | 0.001956239 | 0.003622118 |
| MAGEC1 | 4.599968 | -1.71436 | 0.001960856 | 0.003629916 |
| MFSD2A | 1.013908 | 3.268353 | 0.001975855 | 0.003655414 |
| ALLC | -1.29697 | -2.68299 | 0.001980018 | 0.003662736 |
| GAGE1 | 3.886661 | -3.14597 | 0.001994395 | 0.003687808 |
| TUBA3E | 2.189088 | -2.57835 | 0.002024685 | 0.003739181 |
| KIF5A | -1.06219 | 0.598769 | 0.002029578 | 0.003747446 |
| TMEM236 | 1.129775 | 0.300288 | 0.002058018 | 0.00379526 |
| C10orf107 | 1.181545 | 1.830606 | 0.002059191 | 0.00379703 |
| ANKRD62 | 1.655409 | -4.13015 | 0.002090116 | 0.003850882 |
| TAS2R3 | 1.177989 | -3.62499 | 0.002107413 | 0.003879955 |
| UGT2B17 | 1.388946 | -2.11962 | 0.002112493 | 0.003888507 |
| SFTPA1 | -1.68546 | -1.62684 | 0.002120283 | 0.003900842 |
| PCDHGB2 | 1.168657 | 2.007516 | 0.002132129 | 0.003922231 |
| MAB21L1 | -1.04424 | -2.7077 | 0.002144054 | 0.00394093 |
| MLPH | 1.189853 | 3.562625 | 0.002156837 | 0.003961984 |
| DCAF8L2 | 3.482177 | -3.59279 | 0.002207869 | 0.004047836 |
| AC091167.7 | -1.0012 | -4.04603 | 0.002226534 | 0.004080383 |
| COX7B2 | 3.779856 | -3.56168 | 0.002228864 | 0.004083817 |
| ATG9B | 1.026476 | 0.72582 | 0.002267002 | 0.004150721 |
| SLC38A4 | -1.023 | 2.140968 | 0.002271884 | 0.004159235 |
| TSPY2 | 3.595365 | -4.02281 | 0.00231782 | 0.004237697 |
| NMU | 2.244143 | 0.956572 | 0.00231877 | 0.004238771 |
| RTP5 | 1.477787 | -2.97146 | 0.002318881 | 0.004238771 |
| OR4A47 | 3.343132 | -3.98594 | 0.002337981 | 0.00427194 |
| MAP3K19 | 1.388682 | -2.55825 | 0.002346152 | 0.004285995 |
| CXCL2 | 1.126461 | 3.301914 | 0.002346996 | 0.004287099 |
| RSPH6A | 1.361266 | -2.84429 | 0.002348712 | 0.004289796 |
| STEAP1B | 1.318905 | -2.12572 | 0.002362651 | 0.004314123 |
| FOXP2 | 1.090882 | 3.823457 | 0.002362754 | 0.004314123 |
| AOC1 | -1.06718 | 8.037939 | 0.002380358 | 0.004343607 |
| EFCAB8 | 1.270428 | -2.19886 | 0.002391016 | 0.004360389 |
| KIR2DL1 | 1.337932 | -3.37756 | 0.002392245 | 0.004362185 |
| WNT7B | -1.08558 | 3.004568 | 0.002410433 | 0.004391772 |
| TMEM145 | 1.08172 | -0.12212 | 0.002416326 | 0.004402061 |
| C1QL2 | 2.102034 | -3.58208 | 0.002472792 | 0.004496693 |
| TEPP | 1.198584 | -0.66994 | 0.002497877 | 0.004540925 |
| KCNIP1 | -1.05617 | 0.977404 | 0.002513488 | 0.004565596 |
| SLC5A5 | 1.27241 | -2.35805 | 0.002515861 | 0.004568979 |
| MTNR1B | 3.280483 | -3.72949 | 0.002538275 | 0.004606881 |
| HEATR9 | 1.100952 | -2.95503 | 0.002539087 | 0.004607887 |
| ADIG | 1.232246 | -2.35022 | 0.002557242 | 0.00463718 |
| DLX5 | 1.083635 | -1.34101 | 0.002566815 | 0.00465349 |
| SIGLEC6 | 1.304475 | -0.69113 | 0.002585888 | 0.004686644 |
| TAS2R1 | 3.399835 | -2.27382 | 0.00259785 | 0.00470737 |
| MMP8 | 2.085187 | -2.59517 | 0.002621326 | 0.004746546 |
| CACNG8 | 1.119284 | -1.414 | 0.002634524 | 0.004768033 |
| ADGRB1 | 1.625414 | 2.982807 | 0.002643299 | 0.004782648 |
| SPRR3 | -1.78603 | -2.06522 | 0.002644546 | 0.004784235 |
| RHOXF2 | 3.114439 | -3.95134 | 0.002662476 | 0.004815701 |
| SYTL5 | -1.31724 | 0.309444 | 0.002689573 | 0.004864219 |
| ETNPPL | -1.10963 | 1.283048 | 0.002701105 | 0.004883596 |
| TRIML2 | 2.246999 | -3.32733 | 0.002723508 | 0.004922608 |
| CRYBG2 | 1.066884 | 0.70925 | 0.002729783 | 0.004931958 |
| SIT1 | 1.001245 | 0.846136 | 0.002753721 | 0.004972197 |
| KCNH7 | 1.25949 | -1.28915 | 0.002780887 | 0.005017199 |
| PLA2G4E | 1.166087 | -3.41943 | 0.002844265 | 0.005123283 |
| FGG | 2.808989 | 2.765617 | 0.002885644 | 0.00519155 |
| AADAC | 2.819761 | -3.20257 | 0.00289575 | 0.005207636 |
| AC026464.1 | 2.130158 | -2.86148 | 0.002951373 | 0.005297613 |
| SYT8 | 2.058525 | 1.085781 | 0.002972977 | 0.005333126 |
| CWH43 | -1.19381 | 3.025683 | 0.002976341 | 0.005338091 |
| MIA | 1.740353 | -2.29216 | 0.002993613 | 0.00536853 |
| UGT1A6 | 1.514619 | 5.647634 | 0.002999036 | 0.005377717 |
| SORCS3 | -1.33386 | -0.50297 | 0.003000676 | 0.005380119 |
| KCNQ2 | 2.965685 | 0.737244 | 0.003001565 | 0.005381173 |
| SIX3 | 2.468482 | 0.063546 | 0.003012673 | 0.005400546 |
| 14-Sep | 2.170902 | -3.69117 | 0.003052625 | 0.005468332 |
| ACP5 | 1.079322 | 7.472134 | 0.003072634 | 0.005501422 |
| ANGPTL8 | 1.589205 | 0.648067 | 0.003074322 | 0.005503893 |
| SALL4 | 1.615701 | -0.78143 | 0.003132735 | 0.005603427 |
| NWD2 | 1.739744 | -2.98537 | 0.003202827 | 0.005719082 |
| OPN4 | 1.626767 | -3.78999 | 0.003216998 | 0.005740951 |
| TEX101 | 1.945716 | -3.18979 | 0.00322075 | 0.005746501 |
| SFRP4 | 1.057751 | 3.586411 | 0.003225963 | 0.005755228 |
| GRID1 | 1.391878 | 1.804172 | 0.00324282 | 0.005783573 |
| SNX31 | 2.380461 | -0.28969 | 0.003282258 | 0.005848728 |
| COL6A5 | -1.08442 | -2.95312 | 0.003283962 | 0.005851121 |
| HIST2H3C | 1.91499 | -3.76071 | 0.003295698 | 0.005870278 |
| PAGE2 | 2.540047 | -3.59081 | 0.003301561 | 0.005880136 |
| AC015813.2 | 1.497184 | -4.02497 | 0.003307147 | 0.005889498 |
| PON3 | -1.39442 | 0.415632 | 0.003326166 | 0.005919834 |
| TNC | 1.074272 | 8.257418 | 0.003356095 | 0.005967761 |
| PRSS46 | 1.405315 | -2.4746 | 0.003358474 | 0.005971399 |
| F2 | 1.541589 | -0.62311 | 0.003369585 | 0.005989965 |
| PKP3 | 1.273615 | 3.704585 | 0.003371134 | 0.005991528 |
| MYBPC1 | 1.985889 | -1.67483 | 0.003404589 | 0.006047385 |
| MAGEA9B | 3.432569 | -3.9335 | 0.003447524 | 0.00611727 |
| KCNB1 | 1.130576 | 2.217403 | 0.003447692 | 0.00611727 |
| NLRP9 | 1.257139 | -1.5512 | 0.003479549 | 0.006168902 |
| TBR1 | 1.850865 | -3.91146 | 0.003485576 | 0.006177752 |
| C17orf99 | 1.461142 | -2.36712 | 0.003489987 | 0.006184957 |
| BRSK2 | 1.193937 | 0.471455 | 0.003504292 | 0.006209079 |
| CRYBA2 | 2.37926 | -3.91055 | 0.003512298 | 0.006222224 |
| MEGF11 | 1.217653 | 3.112312 | 0.003536541 | 0.006261261 |
| LRP1B | -1.13178 | 0.943548 | 0.003573642 | 0.006321946 |
| CBSL | 1.26073 | -1.65218 | 0.003577512 | 0.006327507 |
| DTHD1 | 1.142081 | -1.76021 | 0.003577846 | 0.006327507 |
| GABRG2 | 2.899317 | -3.36923 | 0.003585393 | 0.006340228 |
| MS4A10 | -1.83928 | -1.3079 | 0.003594158 | 0.006353844 |
| ESPNL | 1.113981 | 0.924316 | 0.00360023 | 0.00636395 |
| MEOX1 | -1.12777 | -1.70877 | 0.003622582 | 0.006399037 |
| CYP11B1 | 3.704481 | -2.9752 | 0.003646827 | 0.006439958 |
| CTSG | 1.310995 | 1.266156 | 0.003653459 | 0.006450042 |
| RBPJL | 1.525639 | -2.60271 | 0.003656718 | 0.006454657 |
| FAM71C | 1.294585 | -4.07961 | 0.003659012 | 0.006457653 |
| GAGE10 | 1.299434 | -3.7085 | 0.003711574 | 0.0065459 |
| EGFL6 | -1.39048 | -0.08351 | 0.003736327 | 0.006588906 |
| CMA1 | 1.339994 | -0.65247 | 0.003771928 | 0.00664645 |
| CCL21 | -1.22395 | 3.088294 | 0.003789418 | 0.006675297 |
| PCDHA9 | 1.04545 | -1.88415 | 0.003807014 | 0.006702995 |
| TMEM132D | 1.448028 | 0.048471 | 0.003855295 | 0.006781334 |
| AC010325.1 | 1.083133 | -3.96583 | 0.003863253 | 0.006793997 |
| ANKRD1 | 1.195271 | 0.90113 | 0.003871709 | 0.00680753 |
| KRT39 | 1.812058 | -3.10632 | 0.003940053 | 0.006920221 |
| ASTN1 | 2.067008 | 1.39749 | 0.003952259 | 0.006940297 |
| STAR | 1.341706 | -1.859 | 0.003953908 | 0.006941329 |
| NR5A1 | 2.23438 | -3.59245 | 0.003971358 | 0.006970416 |
| ICOS | 1.043951 | -1.02775 | 0.003981231 | 0.006985691 |
| HIST1H3J | 1.447768 | -4.07619 | 0.003982879 | 0.006987897 |
| OBP2B | 1.828749 | -3.97025 | 0.003983528 | 0.006988351 |
| SLC17A8 | 1.270107 | -0.84736 | 0.00398933 | 0.006997844 |
| KRT13 | 3.030604 | 5.049509 | 0.004046776 | 0.007089581 |
| DLK1 | -1.75757 | -1.55646 | 0.004082247 | 0.007146826 |
| LCE5A | 1.184084 | -3.74303 | 0.004089098 | 0.007158121 |
| C9orf152 | -1.09793 | -2.39326 | 0.004091495 | 0.007161616 |
| CD207 | 1.134818 | 0.946866 | 0.004101769 | 0.007178196 |
| NOL4 | 1.614459 | 2.217006 | 0.004106511 | 0.007185091 |
| AC098850.4 | 1.89482 | -1.40012 | 0.004119819 | 0.007206965 |
| KDM5D | -1.20468 | 3.651078 | 0.004127573 | 0.007219825 |
| TAAR8 | 1.451406 | -3.86726 | 0.004142869 | 0.007243748 |
| KCTD16 | 1.046688 | 4.035367 | 0.004179849 | 0.007304839 |
| TMEM114 | 1.549754 | -3.93577 | 0.004192937 | 0.007325568 |
| TLX3 | 2.824998 | -3.93964 | 0.004226064 | 0.007380562 |
| RFPL4B | -1.23126 | -3.6631 | 0.004229639 | 0.007385365 |
| ADAM33 | -1.02052 | -0.24115 | 0.004233288 | 0.007391016 |
| MAGEB18 | 3.089667 | -3.97377 | 0.004282055 | 0.007471059 |
| ZDHHC22 | 1.692545 | -4.04109 | 0.004341717 | 0.007567042 |
| DLX6 | 1.544971 | -2.64186 | 0.004354709 | 0.007587468 |
| TMEFF2 | 1.215974 | -0.99859 | 0.004432316 | 0.007717429 |
| SOX1 | 2.484422 | -4.00734 | 0.00448418 | 0.007803938 |
| BEND2 | 2.946521 | -2.72067 | 0.004505014 | 0.007836387 |
| GREB1L | 1.035196 | 2.005322 | 0.004519113 | 0.00785862 |
| RFX6 | 1.723692 | -3.98434 | 0.004536971 | 0.007887376 |
| WISP3 | 1.356182 | 2.199759 | 0.004540022 | 0.007891915 |
| TMEM179 | 1.419428 | 0.344363 | 0.004569637 | 0.007938769 |
| FOXG1 | 2.057294 | -3.12672 | 0.004590522 | 0.007968092 |
| FOXE1 | 1.790738 | 0.223134 | 0.004620627 | 0.008017237 |
| COL11A1 | 1.39682 | 0.509852 | 0.004638982 | 0.008046746 |
| KRT77 | 2.492324 | -3.95661 | 0.004687096 | 0.00812312 |
| IL1RN | 1.105012 | 2.215325 | 0.004710118 | 0.008159859 |
| LPAR3 | 2.011618 | -1.23526 | 0.004726832 | 0.008184854 |
| SERPINB11 | 3.496757 | -3.09447 | 0.004777854 | 0.008269203 |
| NLGN4Y | -1.24389 | 1.482564 | 0.004867393 | 0.008414408 |
| RBP4 | 1.528789 | 8.394524 | 0.004902211 | 0.008469692 |
| ADGRG4 | 1.884877 | -2.16369 | 0.004904403 | 0.008472662 |
| ZIC1 | 2.471364 | -1.01022 | 0.00491733 | 0.008493355 |
| MYBPC2 | 1.099317 | 0.29388 | 0.004961459 | 0.008563791 |
| KRT1 | 1.18916 | -1.54964 | 0.005022082 | 0.008659632 |
| NAA11 | 2.222243 | -3.98506 | 0.005037412 | 0.008684841 |
| RFPL2 | 1.034307 | -2.36357 | 0.005042101 | 0.008692089 |
| LMNTD1 | -1.33304 | -4.10082 | 0.005060344 | 0.008721019 |
| FAM9C | 2.144922 | -3.07601 | 0.005070854 | 0.008738289 |
| FAM133A | 1.581103 | -1.25534 | 0.005136726 | 0.00884329 |
| BFSP2 | -1.04704 | -2.54782 | 0.005163853 | 0.008887428 |
| ANKRD30BL | 1.922357 | -3.70097 | 0.005241797 | 0.009007748 |
| SLC30A10 | 2.345668 | -2.7249 | 0.005243071 | 0.009009043 |
| PLXNB3 | 1.138834 | 1.409036 | 0.00525495 | 0.009027722 |
| POF1B | 1.271064 | 3.628453 | 0.005267348 | 0.009047285 |
| OR1N2 | 3.253967 | -3.61417 | 0.005288069 | 0.009081133 |
| GRIA4 | 1.684472 | 2.322208 | 0.005300421 | 0.009100598 |
| OR52N4 | 1.008319 | -2.562 | 0.005333058 | 0.009152246 |
| FUT9 | 1.95853 | 1.207533 | 0.005356933 | 0.009191456 |
| IL2 | 1.267419 | -3.96782 | 0.005439592 | 0.009320775 |
| AL772284.2 | 2.972903 | -4.16394 | 0.005449641 | 0.009337101 |
| SPDYC | -1.05225 | -3.32487 | 0.005493626 | 0.009408859 |
| CALN1 | 1.921627 | -1.41799 | 0.005504139 | 0.009424311 |
| MAS1 | -1.11812 | -3.87755 | 0.00555214 | 0.009500894 |
| SP8 | 2.081439 | -4.13852 | 0.005574247 | 0.009534168 |
| ZIC3 | 2.563777 | -2.03752 | 0.005592269 | 0.009562251 |
| NLRP4 | 1.608464 | -3.08084 | 0.005662137 | 0.009673403 |
| SLITRK6 | 3.268163 | 1.227925 | 0.00569861 | 0.009731071 |
| PRSS54 | 1.005169 | -3.72579 | 0.005701446 | 0.009734986 |
| ADH1A | -1.04964 | -3.41442 | 0.005734429 | 0.009786635 |
| AIRE | 1.301726 | -1.64923 | 0.005834879 | 0.009946687 |
| DEFA4 | 1.42081 | -3.98432 | 0.005852793 | 0.009971528 |
| KLK13 | 1.337616 | -2.45287 | 0.005876143 | 0.010007501 |
| SLITRK1 | 2.58032 | -0.75186 | 0.005955507 | 0.01012821 |
| MOGAT3 | 1.298896 | 1.951721 | 0.006042173 | 0.010261949 |
| APOA2 | 2.404042 | 0.042198 | 0.00605252 | 0.010278547 |
| PNMA8C | 1.087227 | -2.89106 | 0.006066641 | 0.010299596 |
| SERPINA7 | 1.54752 | -0.17907 | 0.006124536 | 0.010392959 |
| SBK2 | 1.125403 | -2.55355 | 0.006179343 | 0.010478016 |
| MMP16 | 1.120323 | 0.308023 | 0.006189579 | 0.010493385 |
| GAP43 | 1.325956 | -2.10744 | 0.006231029 | 0.01055466 |
| SLX1B | 1.0966 | -3.61001 | 0.006287857 | 0.010644877 |
| FSIP2 | 1.036744 | 1.184047 | 0.006307581 | 0.010675239 |
| ANTXRL | 2.348942 | -2.78147 | 0.006337728 | 0.010724234 |
| FGF21 | 2.428114 | -3.79093 | 0.006359515 | 0.010759066 |
| ALPPL2 | 2.492632 | -3.79755 | 0.006371367 | 0.01077708 |
| AC002429.2 | 1.016212 | -3.06463 | 0.006461329 | 0.010919963 |
| KRTAP4-1 | 3.115731 | -4.03861 | 0.006462568 | 0.010921026 |
| PCSK2 | -1.25387 | -1.4289 | 0.00650052 | 0.010981014 |
| TCF23 | -1.12516 | -2.29741 | 0.006551786 | 0.011062395 |
| SLCO1B3 | 3.189153 | -3.40005 | 0.006650958 | 0.011218204 |
| SERPINA11 | 1.697938 | -4.08248 | 0.006671715 | 0.011251095 |
| PPEF2 | 1.033631 | -3.07833 | 0.006680778 | 0.011263196 |
| PLET1 | 1.710519 | -3.59952 | 0.006704348 | 0.011301868 |
| CGB7 | 1.325411 | -3.05352 | 0.006709099 | 0.011307747 |
| FST | -1.18289 | 0.656686 | 0.006724264 | 0.01132904 |
| HES7 | 1.022741 | -2.67729 | 0.006728069 | 0.011334384 |
| IVL | 2.989093 | -0.24507 | 0.006772856 | 0.011399106 |
| VCX3B | 1.039749 | -3.22676 | 0.006799426 | 0.011437902 |
| ZIC4 | 2.551798 | -1.98901 | 0.006816251 | 0.011462444 |
| APELA | -1.20049 | 0.168216 | 0.006922324 | 0.011624439 |
| KLK5 | -1.4538 | -1.05913 | 0.0069337 | 0.011641358 |
| STPG4 | 1.063216 | -3.18269 | 0.006969344 | 0.011699009 |
| ZIC5 | 2.901415 | -1.95307 | 0.006992877 | 0.01173631 |
| ETV3L | 1.332124 | -3.97448 | 0.007014122 | 0.011767554 |
| PGPEP1L | -1.16561 | -2.09435 | 0.007057925 | 0.011835496 |
| OPRK1 | 2.634868 | -2.67778 | 0.007094172 | 0.01189071 |
| ALPP | 2.53108 | -3.2594 | 0.007100516 | 0.011900228 |
| NTF4 | 1.681583 | -2.01027 | 0.007164319 | 0.01199705 |
| CP | 1.549121 | 4.803677 | 0.007177189 | 0.012016354 |
| TRIM54 | 1.130541 | 1.65162 | 0.007223257 | 0.012081055 |
| NEUROD1 | -1.44977 | -3.9994 | 0.007243002 | 0.012106161 |
| ASB5 | -1.25638 | -1.109 | 0.007358884 | 0.012289522 |
| SCGB2A1 | 1.375948 | 1.564786 | 0.00756661 | 0.012618771 |
| DMRT3 | 2.06681 | -3.64812 | 0.00758793 | 0.012651968 |
| LCN8 | -1.2526 | -3.02931 | 0.007615693 | 0.012694713 |
| CXorf67 | 1.221548 | -2.80902 | 0.007687737 | 0.012800501 |
| DMP1 | 1.510259 | -3.79458 | 0.007787614 | 0.012949937 |
| TMPRSS15 | 2.546318 | -2.08832 | 0.007821661 | 0.013002931 |
| KLRF2 | 1.473967 | -3.92058 | 0.007838302 | 0.013028174 |
| ERICH3 | 1.190674 | -1.16732 | 0.007981669 | 0.013243106 |
| TM4SF20 | 1.238902 | -3.07 | 0.008001871 | 0.013264329 |
| TNNT3 | 1.518248 | -0.31907 | 0.008017465 | 0.013287719 |
| SLCO1B1 | 2.196295 | -3.61622 | 0.008068613 | 0.013361354 |
| CEACAM5 | 2.306665 | -1.18136 | 0.008146528 | 0.013481649 |
| C17orf98 | 1.320252 | -3.45075 | 0.008187097 | 0.013543778 |
| CXCL11 | 1.049143 | -0.107 | 0.008282101 | 0.013689557 |
| TCERG1L | 1.39192 | -1.52712 | 0.008293341 | 0.01370687 |
| INHA | 1.071639 | 1.210438 | 0.008315798 | 0.013737643 |
| NANOG | 1.031794 | -3.6924 | 0.00837209 | 0.013822983 |
| AQP12B | 2.712487 | -4.06914 | 0.008433477 | 0.013920487 |
| UTF1 | 1.029841 | -4.066 | 0.00855332 | 0.014105296 |
| DYDC1 | 1.403768 | -3.91587 | 0.008573619 | 0.014136166 |
| WNT2 | 1.319471 | 0.735799 | 0.008665976 | 0.014279238 |
| TRIM29 | 1.240826 | 3.817641 | 0.008684717 | 0.014303535 |
| TP63 | 1.797555 | 1.823698 | 0.00873412 | 0.014378286 |
| PITX2 | 1.990453 | 1.652778 | 0.008778207 | 0.014444222 |
| IGLL1 | 1.136387 | -2.68843 | 0.008780629 | 0.014446878 |
| CPNE4 | 1.171548 | 2.419378 | 0.00882296 | 0.014508525 |
| GDF6 | -1.04443 | -0.71895 | 0.008879765 | 0.014588535 |
| IP6K3 | -1.09115 | 1.911029 | 0.008966953 | 0.014718267 |
| CPB2 | 1.196165 | -3.03757 | 0.009159093 | 0.015018496 |
| CCDC177 | 1.462839 | -3.7795 | 0.009199944 | 0.015081337 |
| CTAGE15 | 1.058495 | -2.03033 | 0.009201674 | 0.015082791 |
| SULT2A1 | 2.306143 | -3.00662 | 0.009290706 | 0.015220364 |
| HS3ST6 | -1.15674 | -1.54254 | 0.009462237 | 0.015482952 |
| ZNF536 | -1.08034 | -2.30866 | 0.009646054 | 0.015759242 |
| SHOX | 1.473444 | -3.62278 | 0.009726096 | 0.015882763 |
| CCDC144NL | 1.817263 | -3.7391 | 0.009729951 | 0.015887608 |
| BAAT | 1.468353 | -0.73643 | 0.010006057 | 0.016310182 |
| MAGEB10 | 2.246699 | -4.1739 | 0.010040015 | 0.016361066 |
| OR1L8 | -1.00143 | -3.2528 | 0.010056068 | 0.016384243 |
| NLRP7 | 1.553664 | -2.03113 | 0.010075355 | 0.016414173 |
| ZAN | 1.805363 | -2.26342 | 0.010154611 | 0.016538776 |
| SERPINB4 | 3.204851 | -1.15332 | 0.010326632 | 0.016800603 |
| TTC24 | 1.042332 | -2.55814 | 0.010396904 | 0.016904175 |
| CACNG5 | -1.06497 | -3.49831 | 0.010503418 | 0.017064956 |
| GABRA3 | 2.183517 | -2.03259 | 0.010512829 | 0.017078696 |
| FFAR1 | 1.147772 | -3.24258 | 0.010624219 | 0.017248697 |
| PGLYRP3 | 2.321406 | -3.90828 | 0.010665626 | 0.017308074 |
| GTSF1L | 2.150235 | -3.51857 | 0.010822191 | 0.017544652 |
| DRD5 | 1.010455 | -3.42687 | 0.011000809 | 0.017808417 |
| TAGLN3 | 1.076643 | 2.994198 | 0.011148001 | 0.018033649 |
| HCRT | 1.034463 | -3.77147 | 0.011319116 | 0.01828897 |
| SLC44A5 | 1.342885 | 2.984371 | 0.011349593 | 0.018333249 |
| UGT2B15 | 1.443004 | -3.47845 | 0.011383098 | 0.018382393 |
| TSGA13 | 1.070646 | -4.0069 | 0.011829112 | 0.019045955 |
| CLCA4 | 2.782081 | -0.92999 | 0.011880364 | 0.019119877 |
| CIB4 | -1.05276 | -2.95827 | 0.011918617 | 0.019179716 |
| KRT72 | 1.140206 | -3.69748 | 0.011995104 | 0.019290662 |
| DKK1 | 1.434667 | -0.15279 | 0.012098166 | 0.019437199 |
| ARL14 | 1.738094 | 0.143703 | 0.012274431 | 0.019706242 |
| MMP3 | 1.952021 | -2.01465 | 0.012749051 | 0.020404181 |
| P2RX3 | 1.289454 | -3.30258 | 0.012948975 | 0.020703787 |
| DDX43 | 1.408542 | 1.094437 | 0.012989164 | 0.02076248 |
| FRG2 | -1.5827 | -3.67868 | 0.013050862 | 0.020855516 |
| FAM153A | 1.302258 | 0.849651 | 0.013089031 | 0.020907178 |
| LDHC | -1.01555 | -1.04795 | 0.013251477 | 0.02113083 |
| IL22RA2 | 1.65658 | -3.56148 | 0.013349492 | 0.021264394 |
| MROH9 | 1.184574 | -4.11173 | 0.013431972 | 0.021382457 |
| KIR3DL1 | 1.251066 | -2.89584 | 0.013505607 | 0.021488212 |
| KCNK9 | 1.469433 | -1.03018 | 0.013640721 | 0.021691618 |
| STMN2 | 1.873078 | -1.96969 | 0.013648895 | 0.021702689 |
| CBLN4 | 1.482672 | 1.303834 | 0.013771362 | 0.021872162 |
| GPRC6A | -1.68086 | -1.08891 | 0.013806685 | 0.021920486 |
| GIP | 1.723852 | -3.93415 | 0.013817971 | 0.021934513 |
| ZIC2 | 2.314946 | -0.87884 | 0.013933956 | 0.022104905 |
| OOEP | 1.994185 | -0.67063 | 0.014123152 | 0.022363428 |
| EPYC | 1.77116 | -3.83343 | 0.014221076 | 0.022512512 |
| AC073896.1 | 1.017194 | -4.17473 | 0.014717229 | 0.023238345 |
| KCNK12 | 1.319742 | -0.78512 | 0.015172991 | 0.023901065 |
| VAX1 | 2.058283 | -3.27552 | 0.015211681 | 0.023949368 |
| PLAC1 | 1.57907 | -3.26279 | 0.015331994 | 0.024119696 |
| HOXD13 | 1.561155 | -2.861 | 0.015359103 | 0.02415819 |
| KLRC4 | 1.12363 | -3.88878 | 0.015466573 | 0.024322861 |
| TH | 1.203017 | -1.64906 | 0.015639553 | 0.024558214 |
| CD177 | 1.215739 | -0.16424 | 0.015785094 | 0.024767199 |
| TMPRSS11E | 2.586926 | -1.12619 | 0.015865019 | 0.024886058 |
| C6orf118 | 1.272505 | -1.53925 | 0.015870335 | 0.024892217 |
| NMNAT2 | 1.131311 | 1.416954 | 0.016200428 | 0.025361072 |
| RPS4Y1 | -1.06585 | 5.925476 | 0.016523921 | 0.025829087 |
| SERPINB3 | 3.042852 | 0.141862 | 0.016561464 | 0.025883252 |
| MMP12 | 1.471874 | -0.15472 | 0.01674455 | 0.026145148 |
| FOXI3 | 2.142109 | -3.48387 | 0.016760891 | 0.026165232 |
| SPRR2A | -1.16214 | -2.53264 | 0.017011833 | 0.02653846 |
| GJB7 | 1.108561 | -3.22753 | 0.017040031 | 0.026577815 |
| LIPI | 1.233483 | -3.4756 | 0.017047458 | 0.026587084 |
| TNFSF11 | 1.109596 | -1.66942 | 0.017094671 | 0.026653751 |
| TTLL6 | 1.023279 | 2.61107 | 0.01764688 | 0.027438269 |
| HTR2A | 1.27304 | -0.57327 | 0.017678253 | 0.027482276 |
| MUCL1 | 2.270981 | -2.3073 | 0.017968924 | 0.027905067 |
| ZSCAN10 | 1.249641 | -3.71066 | 0.018208274 | 0.028247363 |
| CT55 | 1.488447 | -3.73448 | 0.018772814 | 0.02907277 |
| CYP24A1 | 1.21119 | 5.838367 | 0.018978404 | 0.029373371 |
| EPHA6 | 1.210814 | 0.360142 | 0.019038136 | 0.029463272 |
| TMEFF1 | 1.147739 | -3.39874 | 0.019157368 | 0.029624745 |
| STXBP5L | 1.978382 | -1.96492 | 0.019403666 | 0.029974967 |
| RXFP3 | 1.289136 | -4.13194 | 0.019904392 | 0.030689796 |
| NKX3-2 | 1.236167 | -2.85042 | 0.019975367 | 0.030788622 |
| TEX48 | 1.581882 | -4.10178 | 0.020089627 | 0.030956737 |
| ARSH | -1.18607 | -4.08081 | 0.020111795 | 0.030985562 |
| KCNA2 | 1.380565 | -1.46522 | 0.020287097 | 0.031226082 |
| NKX2-3 | 1.93179 | -2.58269 | 0.020718625 | 0.031813699 |
| KIF1A | 1.594625 | 2.658655 | 0.02078993 | 0.031911337 |
| DAPL1 | 1.454004 | 0.799892 | 0.020954216 | 0.032150632 |
| LHX8 | 2.252161 | -3.74766 | 0.021239536 | 0.032549348 |
| SNTG1 | -1.36301 | -1.63554 | 0.021759911 | 0.033269915 |
| NAP1L6 | -1.0862 | -0.83878 | 0.022183763 | 0.033885334 |
| C14orf39 | 1.936756 | -3.58784 | 0.022599404 | 0.034479838 |
| BRDT | 1.272566 | -3.972 | 0.022819884 | 0.03477769 |
| CDH8 | 1.351189 | -1.59065 | 0.0228821 | 0.034860636 |
| CT45A1 | 2.417673 | -4.16204 | 0.023582051 | 0.035829426 |
| SERPINA3 | 1.10244 | -0.21072 | 0.023674498 | 0.035951577 |
| SHD | 1.277667 | -2.01316 | 0.023701266 | 0.035989172 |
| MYPN | 1.035106 | -3.13822 | 0.023968067 | 0.036360371 |
| PGLYRP4 | 1.596415 | -3.46858 | 0.02404875 | 0.036467317 |
| VNN1 | 1.175416 | 5.86411 | 0.024157525 | 0.036626057 |
| AGR3 | 1.430849 | 1.510175 | 0.024334113 | 0.036878172 |
| MYH1 | 1.26595 | -2.05794 | 0.024573289 | 0.037209139 |
| CLCA1 | 2.259418 | -3.86067 | 0.024980996 | 0.037781751 |
| LRRC14B | 1.52077 | -1.01236 | 0.025329392 | 0.038273102 |
| MMP26 | 1.1708 | -4.02772 | 0.025821278 | 0.038925408 |
| KRT5 | 1.630056 | 2.036268 | 0.025867811 | 0.038978968 |
| UGT1A1 | 1.211796 | -2.24076 | 0.025934108 | 0.039061545 |
| FETUB | -1.09449 | -1.97022 | 0.026108789 | 0.039304804 |
| SLC6A14 | 1.955111 | -1.94815 | 0.026124295 | 0.039324841 |
| MYBPHL | 1.342237 | -3.16423 | 0.026478207 | 0.03981406 |
| DYNAP | 1.500675 | -4.16889 | 0.026648068 | 0.040035842 |
| PRSS56 | 1.865919 | -2.85487 | 0.026890622 | 0.040366375 |
| FFAR3 | 1.023195 | -3.71319 | 0.027528849 | 0.041262156 |
| CCL25 | 1.39149 | -3.2521 | 0.027833324 | 0.041676649 |
| CRISP2 | -1.3239 | -1.41246 | 0.028623321 | 0.042734452 |
| ALX3 | 2.104477 | -3.85246 | 0.028975811 | 0.043221065 |
| SLCO1A2 | -1.1207 | 0.484607 | 0.029256937 | 0.043611328 |
| DNAH11 | 1.12327 | 1.156489 | 0.02971162 | 0.044237521 |
| PADI6 | 1.400944 | -3.78661 | 0.030568648 | 0.04540403 |
| RPL10L | 1.258279 | -3.86466 | 0.031174333 | 0.046207811 |
| LIN28A | 1.199533 | -3.68822 | 0.031401585 | 0.046525391 |
| TNFRSF17 | 1.090346 | -0.38517 | 0.031558979 | 0.04672765 |
| KLF14 | 1.074695 | -2.38936 | 0.031726356 | 0.046948293 |
| CALHM1 | 1.392196 | -3.45507 | 0.031835963 | 0.047087134 |
| FAM153C | 1.105246 | -1.43652 | 0.032276334 | 0.047691181 |
| ARPP21 | 1.027505 | 0.881831 | 0.032487441 | 0.047983307 |
| MUC5AC | 1.154341 | -3.50745 | 0.032541949 | 0.048055884 |
| PGLYRP2 | 1.079386 | -2.17356 | 0.032678866 | 0.048230223 |
| KCNG4 | 1.190642 | -3.86293 | 0.033020137 | 0.048693753 |
