# Supplementary material for: cg04448376, cg24387542, cg08548498, and cg14621323 as a Novel Signature to Predict Prognosis in Kidney Renal Papillary Cell Carcinoma
Source: Biomed Res Int. 2020 Dec 17;2020:4854390. doi: 10.1155/2020/4854390 (PMC7759405; doi:10.1155/2020/4854390)
Supplement: Supplementary Materials — Table S1: DMGs. Table S2: DEGs. Table S3: the 9 hub genes. Table S4: the methylated sites of 9 hub genes. Table S5: univariate Cox proportional hazards regression analysis (P < 0.05) of the methylated site data in the training dataset. Table S6: the signature risk score composed of 4 site combinations in the training and test dataset. Table S7: the expression of 4 methylated sites in GSE126441. Table S8: functional analysis of the selected 9 hub genes. Fig.S1: identification of the hub genes from DMGs and DEGs. The Venn diagram shows that there are nine hub genes in 79 DMGs and 5100 DEGs. The hub genes are opposite fold change. [file 4854390.f1.zip › Table S4.pdf]

Table S4 the methylated sites of 9 hub genes

id	TCGA.SX.A	TCGA.SX.A	TCGA.2Z.A	TCGA.4A.A	TCGA.2Z.A	TCGA.GL.6	TCGA.BQ.7	TCGA.BQ.7
cg1570123	0.142277	0.063389	0.04754	0.761979	0.108136	0.087139	0.048761	0.056677
cg0326652	0.297474	0.032528	0	0.03179	0.140461	0.16701	0.034841	0
cg2009565	0.95397	0.946393	0.924622	0.942704	0.880359	0.932394	0.935494	0.853836
cg2746738	0.016796	0.01763	0.028423	0.020779	0.030816	0.043512	0.04382	0.020822
cg0795097	0	0	0	0	0	0	0	0
cg0219252	0.090998	0.08471	0.213555	0.076374	0.199236	0.096881	0.273412	0.319942
cg2348034	0.036616	0.046364	0.046439	0.098779	0.093049	0.504586	0.092158	0.173817
cg2524718	0.013172	0.015389	0.014156	0.015313	0.013907	0.012622	0.012382	0.015226
cg0479091	0	0.045018	0	0.062676	0.32951	0.258934	0.049165	0
cg2761935	0.079884	0.140534	0.119748	0.061805	0.124326	0.117155	0.140057	0.163741
cg1359741	0.086164	0.090849	0.112755	0.117036	0.115862	0.221571	0.158706	0.11588
cg0343001	0.145288	0.027668	0	0.022293	0.102825	0.107849	0.023228	0
cg0051044	0.057644	0.038468	0.0337	0.032877	0.043659	0.048383	0.043439	0.054814
cg1421342	0	0	0	0	0	0	0	0
cg1646310	0	0	0	0	0	0	0	0
cg1756069	0	0	0	0	0	0	0	0
cg0883522	0.029216	0.079881	0.035469	0.030003	0.057122	0.038465	0.074049	0.085504
cg0647920	0.159583	0.04581	0	0.022322	0.093005	0.075397	0.034914	0
cg0014215	0.038477	0.038388	0.036392	0.02846	0.034143	0.033776	0.041245	0.041519
cg0444837	0	0.04312	0	0.069862	0	0.294694	0.061763	0
cg1296687	0.07994	0.185936	0.072192	0.296171	0.141523	0.526557	0.258054	0.204295
cg0493510	0.228862	0.277766	0.044271	0.225846	0.517269	0.033835	0.542854	0.033509
cg2173744	0.17911	0.466662	0.076092	0.130628	0.114569	0.068052	0.346485	0.455011
cg1095266	0.087822	0.071979	0.0832	0.076938	0.083235	0.108794	0.083679	0.093105
cg2438754	0.103115	0.072597	0.097873	0.142736	0.120568	0.228472	0.178323	0.116543
cg2388901	0.551546	0.680015	0.136317	0.920758	0.364392	0.926285	0.819469	0.810124
cg2106445	0.116949	0.19762	0.088105	0.064431	0.138746	0.114474	0.170575	0.216998
cg0064246	0.560712	0.034851	0.031363	0.081322	0.02103	0.823506	0.719541	0.072279
cg2682421	0.050099	0.038599	0.038632	0.700003	0.054198	0.077892	0.049089	0.047203
cg1460940	0.233005	0.690086	0.106878	0.857697	0.141384	0.750839	0.791828	0.679084
cg0684762	0.950888	0.238873	0.200905	0.618624	0.09162	0.814872	0.7543	0.240543
cg2613814	0.01368	0.018313	0.015999	0.008863	0.015608	0.015084	0.02447	0.019127
cg0721976	0.122109	0.110879	0.076345	0.174654	0.099063	0.115512	0.117421	0.128199
cg0854849	0.306952	0.066269	0.095256	0.650982	0.43211	0.819754	0.594725	0.438606
cg1938343	0.044654	0.031309	0.026181	0.52063	0.032579	0.034097	0.031172	0.026537
cg2594127	0	0	0	0	0	0	0	0
cg1058774	0.065663	0.065326	0.056197	0.0417	0.06601	0.059025	0.086863	0.063376
cg1724364	0.123379	0.164453	0.331894	0.156606	0.246718	0.153033	0.370553	0.402867
cg0766899	0.024715	0.041531	0.051285	0.288529	0.031394	0.302149	0.235056	0.102355
cg0029266	0.063592	0.070995	0.077046	0.052767	0.076773	0.06963	0.07274	0.056832
cg0128708	0.898214	0.517705	0.586124	0.484205	0.424175	0.856183	0.807232	0.43082
cg0682295	0.013777	0.01204	0.012855	0.011146	0.013634	0.01047	0.009236	0.011816
cg1985376	0.134042	0.335636	0.071778	0.074351	0.15023	0.175306	0.313403	0.299568
cg0126410	0.048129	0.093932	0.04024	0.027876	0.04732	0.051732	0.077392	0.091948
cg1462132	0.580772	0.350191	0.454634	0.866197	0.428559	0.851743	0.819771	0.497061
cg1973373	0.065856	0.067223	0.040433	0.043298	0.098543	0.05433	0.151204	0.181373
cg2680829	0.040079	0.03042	0.045322	0.027708	0.056877	0.056153	0.053949	0.062883

TCGA. A4. 8	TCGA. SX. A	TCGA. 5P. A	TCGA. G7. 6	TCGA. 2Z. A	TCGA. EV. 5	TCGA. SX. A	TCGA. 4A. A	TCGA. A4. A
0. 048366	0. 080362	0. 089791	0. 041486	0. 043099	0. 04152	0. 069683	0. 049257	0. 070893
0. 035489	0. 093358	0. 045049	0. 040537	0. 105259	0. 055317	0. 21442	0	0. 041245
0. 91694	0. 848165	0. 923154	0. 910324	0. 791571	0. 675672	0. 382666	0. 848188	0. 933389
0. 021397	0. 021503	0. 025586	0. 034172	0. 019891	0. 027964	0. 015907	0. 019001	0. 017631
0	0	0	0	0	0	0	0	0
0. 421717	0. 288162	0. 289838	0. 586054	0. 215206	0. 379024	0. 118789	0. 79885	0. 519604
0. 072125	0. 029471	0. 468614	0. 095824	0. 064458	0. 072724	0. 045091	0. 120488	0. 053594
0. 01598	0. 013684	0. 016825	0. 011706	0. 011674	0. 014761	0. 011799	0. 010755	0. 01141
0. 029453	0. 166765	0. 052556	0. 047379	0. 256896	0. 062779	0. 395919	0	0. 108063
0. 090219	0. 108909	0. 055665	0. 26766	0. 129054	0. 153994	0. 033541	0. 166567	0. 107102
0. 170406	0. 068766	0. 500228	0. 107197	0. 050706	0. 116655	0. 19634	0. 180085	0. 067294
0. 022592	0. 062192	0. 036353	0. 028041	0. 07919	0. 026748	0. 119033	0	0. 037764
0. 063383	0. 045519	0. 062593	0. 107587	0. 033802	0. 072052	0. 057223	0. 068327	0. 0517
0	0	0	0	0	0	0	0	0
0	0	0	0	0	0	0	0	0
0	0	0	0	0	0	0	0	0
0. 042352	0. 036228	0. 056914	0. 153388	0. 049405	0. 044202	0. 034857	0. 145505	0. 043609
0. 039516	0. 063916	0. 017682	0. 052737	0. 054947	0. 044893	0. 102365	0	0. 04796
0. 031364	0. 041369	0. 038362	0. 037098	0. 023226	0. 035379	0. 046789	0. 039455	0. 032533
0. 083217	0. 223989	0. 052284	0. 100824	0. 308054	0. 115644	0. 37599	0	0. 14407
0. 129557	0. 046781	0. 563972	0. 158245	0. 115042	0. 113937	0. 04171	0. 198572	0. 077239
0. 03027	0. 189151	0. 051323	0. 124924	0. 03587	0. 072083	0. 092111	0. 067371	0. 05501
0. 062408	0. 260795	0. 212571	0. 383222	0. 257609	0. 191253	0. 15491	0. 290439	0. 167665
0. 105533	0. 088498	0. 104985	0. 090318	0. 07326	0. 091695	0. 085708	0. 106827	0. 083221
0. 136798	0. 070297	0. 477609	0. 113454	0. 064658	0. 123017	0. 221151	0. 198998	0. 084857
0. 549545	0. 15892	0. 918311	0. 619213	0. 543569	0. 260956	0. 083517	0. 656104	0. 466228
0. 09253	0. 202345	0. 119279	0. 310777	0. 185128	0. 249983	0. 176559	0. 292833	0. 189927
0. 019956	0. 062274	0. 379634	0. 035027	0. 033085	0. 028487	0. 019838	0. 135336	0. 293542
0. 033932	0. 0445	0. 052627	0. 044846	0. 029893	0. 048234	0. 049483	0. 040091	0. 053349
0. 564771	0. 12893	0. 788801	0. 457516	0. 226029	0. 259847	0. 094355	0. 562533	0. 230651
0. 024462	0. 147547	0. 523091	0. 105494	0. 101489	0. 055928	0. 031016	0. 28647	0. 726558
0. 014354	0. 019442	0. 011987	0. 025764	0. 01379	0. 012514	0. 014536	0. 012946	0. 015545
0. 07569	0. 096033	0. 229612	0. 107228	0. 104973	0. 116375	0. 062542	0. 334262	0. 093192
0. 544012	0. 129329	0. 850263	0. 420007	0. 622757	0. 124491	0. 091722	0. 259101	0. 264453
0. 024938	0. 036685	0. 050169	0. 025846	0. 02745	0. 032032	0. 051495	0. 03753	0. 027079
0	0	0	0	0	0	0	0	0
0. 055018	0. 046181	0. 056423	0. 079743	0. 048141	0. 059541	0. 074104	0. 074061	0. 046233
0. 678274	0. 388891	0. 408916	0. 595491	0. 320733	0. 469638	0. 180902	0. 799936	0. 52276
0. 031476	0. 037382	0. 518771	0. 078452	0. 030795	0. 088361	0. 039708	0. 06314	0. 029605
0. 071082	0. 063815	0. 078169	0. 080987	0. 059723	0. 075805	0. 076398	0. 070485	0. 069229
0. 283041	0. 248948	0. 723341	0. 588835	0. 351319	0. 360888	0. 267216	0. 588846	0. 564544
0. 014811	0. 014584	0. 019006	0. 010641	0. 009895	0. 011832	0. 012804	0. 010101	0. 013476
0. 071254	0. 236717	0. 077964	0. 329206	0. 172779	0. 23429	0. 151119	0. 245985	0. 190479
0. 045797	0. 056305	0. 048226	0. 159242	0. 061389	0. 098387	0. 053633	0. 094525	0. 056579
0. 427297	0. 437198	0. 921526	0. 612383	0. 499762	0. 514517	0. 361138	0. 722142	0. 520838
0. 074354	0. 150591	0. 076881	0. 394549	0. 140763	0. 201455	0. 04828	0. 47638	0. 193873
0. 035373	0. 043446	0. 072518	0. 097616	0. 052476	0. 062558	0. 049669	0. 074728	0. 045373

TCGA.AL.A	TCGA.HE.A	TCGA.DZ.6	TCGA.Y8.A	TCGA.A4.A	TCGA.UZ.A	TCGA.B1.A	TCGA.SX.A	TCGA.Y8.A
0.400981	0.041266	0.051786	0.041334	0.079106	0.03346	0.049594	0.061547	0.03108
0	0.040922	0.030402	0.050417	0.107216	0.065182	0.060113	0.076178	0.275738
0.933306	0.832626	0.939042	0.824363	0.95509	0.925971	0.921233	0.931405	0.800328
0.045589	0.039694	0.021393	0.017682	0.027549	0.017157	0.021275	0.02277	0.017471
0	0	0	0	0	0	0	0	0
0.362608	0.348505	0.244188	0.442412	0.231744	0.471138	0.248548	0.223262	0.082121
0.067768	0.027495	0.149959	0.039657	0.057699	0.032563	0.027119	0.430974	0.075781
0.016359	0.014561	0.016338	0.012856	0.015597	0.012364	0.013513	0.016849	0.012975
0.392767	0.121497	0.042627	0.120395	0.191546	0.080571	0.140748	0.15057	0
0.225249	0.17831	0.094615	0.086811	0.208626	0.128161	0.082046	0.062862	0.070052
0.083641	0.128267	0.069053	0.055599	0.110107	0.050783	0.058566	0.182397	0.083198
0	0.045078	0.028587	0.035865	0.061959	0.037343	0.041724	0.058495	0.163216
0.075199	0.045389	0.041925	0.064922	0.061277	0.038523	0.048501	0.057406	0.03798
0	0	0	0	0	0	0	0	0
0	0	0	0	0	0	0	0	0
0	0	0	0	0	0	0	0	0
0.056045	0.046791	0.058523	0.047118	0.081165	0.040244	0.031962	0.066504	0.037818
0	0.054795	0.035057	0.056193	0.073443	0.041737	0.0568	0.054561	0.083779
0.037726	0.031647	0.029662	0.02049	0.044066	0.024891	0.025591	0.041432	0.028765
0	0.18786	0.049809	0.151284	0.402123	0	0.27107	0.229155	0
0.163274	0.076261	0.228091	0.125557	0.095056	0.048435	0.043538	0.448421	0.102319
0.769039	0.027389	0.065782	0.026402	0.06264	0.028534	0.363267	0.052411	0.03622
0.148539	0.291063	0.086847	0.177786	0.329901	0.242421	0.158923	0.19251	0.184792
0.106673	0.096423	0.114133	0.078278	0.111577	0.081398	0.083678	0.08908	0.067273
0.066517	0.126591	0.078196	0.044938	0.106364	0.047573	0.061228	0.215438	0.096112
0.772561	0.298512	0.40637	0.607021	0.36954	0.402025	0.491876	0.846924	0.229987
0.186626	0.181796	0.073511	0.220883	0.195457	0.23002	0.175106	0.116564	0.08383
0.694006	0.090705	0.391993	0.045537	0.043734	0.073335	0.153572	0.178059	0.011811
0.331868	0.031655	0.077567	0.03411	0.048825	0.036466	0.043272	0.054052	0.043968
0.483968	0.221226	0.230047	0.386295	0.215082	0.189015	0.149016	0.632077	0.107368
0.796847	0.111121	0.767021	0.072029	0.240144	0.172655	0.47239	0.517575	0.019637
0.016353	0.021628	0.015619	0.011749	0.016662	0.012335	0.015235	0.018997	0.011702
0.090047	0.072315	0.125076	0.073358	0.097434	0.078492	0.093794	0.144588	0.210204
0.249554	0.349951	0.402118	0.369615	0.227103	0.108247	0.100562	0.732955	0.396195
0.246471	0.022054	0.039361	0.028214	0.042254	0.028464	0.022509	0.035555	0.043358
0	0	0	0	0	0	0	0	0
0.059956	0.057444	0.047606	0.053101	0.07242	0.054368	0.046713	0.051872	0.049138
0.401758	0.399912	0.335167	0.512714	0.324066	0.525793	0.371077	0.275934	0.142612
0.063026	0.033585	0.053074	0.082329	0.03735	0.02797	0.032143	0.1305	0.02631
0.078747	0.081846	0.050633	0.071827	0.079355	0.053014	0.063352	0.064547	0.056832
0.842642	0.43706	0.850671	0.386258	0.376814	0.43902	0.506214	0.655104	0.219581
0.016701	0.014747	0.010753	0.014966	0.014997	0.009304	0.012545	0.016753	0.010816
0.185738	0.185952	0.068841	0.165949	0.251051	0.248037	0.197587	0.128405	0.134936
0.069829	0.067536	0.060716	0.063776	0.073616	0.056971	0.048448	0.048913	0.054892
0.887524	0.546704	0.885683	0.545788	0.503214	0.5537	0.501068	0.449602	0.305508
0.280423	0.148283	0.060116	0.203045	0.147295	0.197047	0.171266	0.095719	0.041001
0.079983	0.054156	0.069375	0.058432	0.075605	0.051352	0.037507	0.040553	0.025652

TCGA.F9.A	TCGA.DZ.6	TCGA.2Z.A	TCGA.IA.A	TCGA.A4.8	TCGA.B9.A	TCGA.F9.A	TCGA.A4.7	TCGA.BQ.5
0.065244	0.056667	0.04666	0.047378	0.040143	0.166009	0.098513	0.046774	0.05364
0	0.045142	0.045385	0.07214	0.133965	0.234933	0	0	0
0.836342	0.923085	0.592957	0.665477	0.436289	0.904352	0.939541	0.689164	0.960239
0.024648	0.027183	0.020122	0.028597	0.021128	0.023419	0.03014	0.031361	0.020496
0	0	0	0	0	0	0	0	0
0.200085	0.483242	0.261781	0.497635	0.129606	0.118114	0.158239	0.162026	0.096232
0.060166	0.110121	0.053827	0.034515	0.142675	0.245965	0.086434	0.030129	0.15641
0.016601	0.012352	0.014607	0.015729	0.017881	0.016594	0.019103	0.012434	0.016546
0	0.066609	0.159652	0.067731	0.217742	0.317366	0	0	0
0.069537	0.234288	0.129678	0.131833	0.120519	0.166011	0.1625	0.144294	0.13842
0.278213	0.13848	0.056417	0.090586	0.152937	0.200343	0.07596	0.079592	0.178024
0	0.030582	0.0615	0.035591	0.050429	0.126695	0	0	0
0.077664	0.067443	0.046352	0.053189	0.041135	0.040289	0.062431	0.046106	0.0411
0	0	0	0	0	0	0	0	0
0	0	0	0	0	0	0	0	0
0	0	0	0	0	0	0	0	0
0.047161	0.122192	0.086403	0.107148	0.036612	0.099711	0.048595	0.055812	0.050249
0	0.054641	0.069573	0.059722	0.060928	0.081905	0	0	0
0.043132	0.039051	0.16016	0.042811	0.040557	0.33818	0.051012	0.036887	0.045329
0	0.092262	0.292855	0.138202	0.323746	0	0	0	0
0.106692	0.156393	0.092176	0.0754	0.189409	0.356091	0.153076	0.103837	0.227899
0.31072	0.04237	0.036977	0.029467	0.028398	0.148723	0.133968	0.04094	0.030366
0.178165	0.320254	0.371325	0.259825	0.100411	0.377123	0.273875	0.087844	0.134798
0.085185	0.126826	0.084641	0.092884	0.103338	0.088491	0.094841	0.10975	0.101031
0.330116	0.118957	0.051649	0.106338	0.16973	0.175762	0.081571	0.099079	0.176153
0.202562	0.453026	0.469318	0.403059	0.4547	0.80345	0.434756	0.138696	0.694591
0.187292	0.299007	0.20248	0.226375	0.132802	0.24516	0.172969	0.100424	0.29277
0.567642	0.155333	0.018269	0.070903	0.029436	0.693358	0.041565	0.018769	0.661797
0.054578	0.043902	0.033788	0.044695	0.046308	0.156942	0.045852	0.054132	0.047762
0.234808	0.333622	0.27482	0.340467	0.154588	0.531422	0.275395	0.151825	0.420639
0.600486	0.303094	0.026507	0.100434	0.26463	0.744223	0.038149	0.138262	0.747722
0.017386	0.017549	0.24073	0.015478	0.015222	0.46349	0.018007	0.014739	0.016588
0.150905	0.109093	0.095752	0.118893	0.066539	0.294758	0.162034	0.124417	0.118843
0.136414	0.267971	0.476699	0.061588	0.697065	0.733559	0.542454	0.117334	0.327213
0.055336	0.030938	0.027839	0.036355	0.026752	0.105467	0.061147	0.030847	0.029638
0	0	0	0	0	0	0	0	0
0.053557	0.071932	0.136748	0.059408	0.054116	0.38347	0.038728	0.050723	0.064362
0.262974	0.516599	0.316136	0.59017	0.194052	0.203483	0.240805	0.246965	0.163302
0.04851	0.118722	0.034073	0.047319	0.037451	0.242536	0.069027	0.042244	0.124175
0.069018	0.065241	0.115614	0.072995	0.079954	0.368879	0.072303	0.068066	0.074676
0.708375	0.622424	0.327439	0.470448	0.2467	0.741009	0.253386	0.270223	0.934735
0.015615	0.01082	0.012769	0.013115	0.01256	0.014459	0.015176	0.013002	0.016346
0.177132	0.373666	0.270882	0.175806	0.160173	0.382808	0.153436	0.156058	0.471841
0.062174	0.124902	0.062629	0.110332	0.064463	0.126217	0.07564	0.067063	0.074431
0.681704	0.603814	0.50352	0.590758	0.41498	0.81499	0.414134	0.38263	0.946206
0.093449	0.25897	0.117275	0.240071	0.077427	0.035134	0.105786	0.099099	0.06095
0.056337	0.072717	0.054096	0.074348	0.056944	0.03261	0.055498	0.055978	0.052534

TCGA.2Z.A	TCGA.IZ.A	TCGA.DW.7	TCGA.G7.6	TCGA.A4.A	TCGA.5P.A	TCGA.BQ.5	TCGA.UZ.A	TCGA.4A.A
0.044142	0.051125	0.036462	0.055048	0.060986	0.09241	0.306714	0.058483	0.049474
0.053962	0.082019	0.066206	0.072976	0.042908	0	0.036464	0.287772	0
0.931283	0.912931	0.751563	0.819288	0.926647	0.613638	0.924563	0.209758	0.43637
0.020864	0.018945	0.025676	0.025022	0.036416	0.022719	0.05177	0.018377	0.014924
0	0	0	0	0	0	0	0	0
0.336705	0.201197	0.243682	0.269665	0.422087	0.443873	0.242749	0.100959	0.11044
0.043478	0.027637	0.094313	0.431522	0.035004	0.839112	0.169048	0.035131	0.045492
0.012567	0.014561	0.015615	0.014853	0.012323	0.014326	0.02231	0.015523	0.01254
0.122744	0.248106	0.1259	0.109323	0.122697	0	0.051529	0.327806	0
0.111677	0.092881	0.153974	0.144749	0.211393	0.185283	0.131724	0.065697	0.089191
0.046868	0.085722	0.085373	0.068166	0.06386	0.296466	0.127122	0.065199	0.079769
0.045828	0.079644	0.046695	0.045415	0.028715	0	0.028144	0.237662	0
0.042591	0.032697	0.047939	0.044588	0.069722	0.04904	0.068148	0.046137	0.043641
0	0	0	0	0	0	0	0	0
0	0	0	0	0	0	0	0	0
0	0	0	0	0	0	0	0	0
0.051141	0.045024	0.097046	0.06467	0.103247	0.218027	0.08119	0.030118	0.020848
0.037045	0.085974	0.055095	0.058056	0.048531	0	0.079719	0.140694	0
0.028471	0.024829	0.057892	0.042191	0.040199	0.040496	0.096353	0.035978	0.024586
0.202091	0.400523	0.253398	0.275749	0.103352	0	0.05587	0	0
0.114492	0.032438	0.152613	0.452931	0.074814	0.464566	0.2254	0.139993	0.072509
0.044743	0.403519	0.038049	0.030499	0.084878	0.118097	0.401136	0.110179	0.157432
0.186106	0.072579	0.160804	0.223226	0.259018	0.753753	0.095867	0.116391	0.127389
0.107908	0.079936	0.090289	0.112858	0.084012	0.085174	0.12244	0.090197	0.085882
0.050326	0.092967	0.071697	0.062635	0.087478	0.262966	0.138188	0.05449	0.088587
0.545653	0.16051	0.423686	0.657386	0.30141	0.812835	0.654965	0.124806	0.447561
0.179627	0.117988	0.198035	0.220228	0.321715	0.545961	0.153663	0.080814	0.137174
0.087498	0.02733	0.071402	0.023619	0.30803	0.034586	0.664233	0.016837	0.010428
0.036533	0.041372	0.052718	0.04085	0.04225	0.048641	0.234421	0.039658	0.045222
0.283224	0.119668	0.243282	0.316988	0.185351	0.793271	0.233188	0.056364	0.121031
0.189953	0.290355	0.041514	0.069838	0.524194	0.034771	0.696447	0.017923	0.012265
0.019261	0.018323	0.015358	0.03063	0.023767	0.032828	0.101825	0.016052	0.012706
0.116154	0.069778	0.153331	0.11415	0.097373	0.20225	0.075743	0.076279	0.28209
0.294162	0.073647	0.589977	0.765597	0.05577	0.894663	0.219379	0.614123	0.199014
0.03243	0.029204	0.03175	0.031385	0.030611	0.030932	0.216642	0.028096	0.036832
0	0	0	0	0	0	0	0	0
0.059076	0.062308	0.066927	0.058223	0.06978	0.060131	0.128008	0.052995	0.044174
0.456611	0.274861	0.265833	0.346948	0.510868	0.820088	0.369392	0.169864	0.211002
0.040635	0.038704	0.044139	0.047539	0.046425	0.531069	0.068588	0.036838	0.029215
0.060978	0.067505	0.085349	0.069226	0.077132	0.076208	0.123041	0.059355	0.062812
0.48481	0.412999	0.418689	0.475044	0.597735	0.321155	0.743259	0.177153	0.127746
0.010042	0.015724	0.014752	0.012279	0.013414	0.015538	0.019009	0.014377	0.010652
0.164496	0.11931	0.220124	0.272893	0.342722	0.374773	0.245357	0.053833	0.351391
0.050867	0.04483	0.061765	0.066685	0.110639	0.314185	0.09274	0.035445	0.038203
0.572194	0.456274	0.525002	0.52248	0.600685	0.432697	0.7787	0.387293	0.331865
0.15751	0.063631	0.153099	0.135742	0.313924	0.348952	0.1136	0.046233	0.064308
0.056933	0.037389	0.061092	0.081724	0.062769	0.055909	0.065672	0.039278	0.027906

TCGA.B3.A	TCGA.DW.7	TCGA.F9.A	TCGA.BQ.5	TCGA.SX.A	TCGA.HE.7	TCGA.DZ.6	TCGA.A4.8	TCGA.P4.A
0.149914	0.074138	0.204815	0.053612	0.069509	0.043348	0.042283	0.042646	0.117401
0.119029	0.080411	0	0	0	0.032022	0.030092	0.09403	0.091668
0.938537	0.771933	0.882959	0.93613	0.940603	0.927821	0.91435	0.936151	0.893971
0.038673	0.026981	0.128496	0.019012	0.02189	0.032193	0.030386	0.02158	0.018933
0	0	0	0	0	0	0	0	0
0.154405	0.151696	0.50697	0.629436	0.347094	0.433139	0.268931	0.151418	0.14764
0.504646	0.203963	0.04894	0.099525	0.042958	0.064535	0.035048	0.148617	0.03777
0.015954	0.01199	0.014651	0.014939	0.013754	0.012012	0.011906	0.016901	0.013792
0.300487	0.225021	0	0	0	0.070303	0.046202	0.25695	0.209029
0.157024	0.136467	0.156438	0.22836	0.13961	0.154116	0.164532	0.162241	0.052077
0.086553	0.211529	0.100099	0.064306	0.045813	0.077868	0.080654	0.078473	0.082352
0.085901	0.067106	0	0	0	0.036487	0.029587	0.061066	0.06447
0.068877	0.044683	0.056839	0.086561	0.053595	0.060959	0.042506	0.052521	0.04107
0	0	0	0	0	0	0	0	0
0	0	0	0	0	0	0	0	0
0	0	0	0	0	0	0	0	0
0.060163	0.044039	0.128033	0.156486	0.070092	0.11671	0.072857	0.042415	0.042532
0.065224	0.055583	0	0	0	0.035789	0.04452	0.106027	0.059165
0.044979	0.029611	0.042708	0.040163	0.035753	0.045727	0.036211	0.037383	0.032914
0.367506	0.325055	0	0	0	0.07594	0.049025	0.380217	0.428055
0.524642	0.340565	0.120296	0.158405	0.100197	0.108528	0.059736	0.1881	0.061062
0.068788	0.057465	0.219914	0.024968	0.072594	0.024696	0.027173	0.069027	0.055497
0.203102	0.077352	0.181835	0.311365	0.2111	0.220135	0.067341	0.244533	0.239609
0.118032	0.073981	0.080549	0.113466	0.085116	0.116323	0.110005	0.112551	0.080032
0.111474	0.189898	0.093082	0.06253	0.071518	0.071802	0.075127	0.078537	0.097464
0.603822	0.62378	0.547486	0.535429	0.655115	0.486963	0.228128	0.502609	0.845634
0.137986	0.149739	0.237563	0.398793	0.180018	0.233776	0.136795	0.278079	0.112883
0.096423	0.018283	0.480216	0.149202	0.155971	0.112602	0.135925	0.071554	0.278068
0.065409	0.038884	0.189238	0.043696	0.035979	0.042483	0.047042	0.033882	0.052564
0.262378	0.237348	0.201283	0.346124	0.326615	0.335319	0.178603	0.282949	0.441369
0.167966	0.026781	0.530737	0.299015	0.403321	0.215168	0.075948	0.102718	0.719863
0.020553	0.014464	0.017604	0.013615	0.01391	0.015695	0.022009	0.02303	0.013525
0.099153	0.107468	0.205615	0.143282	0.10272	0.129171	0.086065	0.096659	0.094418
0.800783	0.405354	0.087722	0.164877	0.374137	0.229787	0.063627	0.362767	0.316385
0.090753	0.028142	0.08923	0.036618	0.032643	0.030956	0.030052	0.02537	0.024241
0	0	0	0	0	0	0	0	0
0.056471	0.055385	0.074938	0.049206	0.052444	0.063199	0.055074	0.082451	0.037197
0.205212	0.201099	0.701768	0.668267	0.439966	0.478059	0.309605	0.155454	0.244404
0.042692	0.029856	0.03583	0.097821	0.035747	0.045253	0.04528	0.026974	0.0237
0.078689	0.065128	0.090126	0.072114	0.072059	0.07969	0.087262	0.07267	0.064375
0.546432	0.585067	0.715458	0.615506	0.677744	0.549176	0.326928	0.463873	0.719534
0.015911	0.010597	0.012884	0.012779	0.017162	0.01493	0.011058	0.019301	0.012079
0.121602	0.132155	0.290755	0.392595	0.218137	0.233743	0.222285	0.41519	0.226948
0.057123	0.047219	0.116862	0.173356	0.045232	0.117434	0.068507	0.067566	0.033799
0.464675	0.449764	0.800193	0.618583	0.527055	0.609253	0.451587	0.44691	0.486039
0.063931	0.061582	0.191259	0.429457	0.154776	0.206457	0.131797	0.075786	0.041667
0.047945	0.046728	0.046417	0.104273	0.060471	0.096501	0.06235	0.064946	0.033615

TCGA.DW.7	TCGA.P4.A	TCGA.BQ.7	TCGA.UZ.A	TCGA.P4.A	TCGA.5P.A	TCGA.BQ.5	TCGA.MH.A	TCGA.G7.6
0.038178	0.158485	0.070375	0.087228	0.0576	0.040459	0.070184	0.256923	0.06431
0.052869	0.115972	0.138885	0	0.305855	0	0	0.059642	0.311161
0.915633	0.927079	0.957288	0.766416	0.923781	0.472741	0.717332	0.941724	0.957095
0.025868	0.106701	0.020067	0.014914	0.036074	0.026774	0.031911	0.02886	0.015178
0	0	0	0	0	0	0	0	0
0.320733	0.291738	0.136068	0.048999	0.147476	0.236984	0.334386	0.247118	0.071987
0.086508	0.032469	0.153396	0.543575	0.127676	0.06965	0.174904	0.315879	0.303104
0.01499	0.016677	0.01324	0.013831	0.017198	0.012676	0.012164	0.016812	0.01592
0.081833	0.285523	0.350644	0.382608	0.336197	0	0	0.176785	0.429435
0.177208	0.154817	0.169394	0.039807	0.185468	0.075643	0.142162	0.145405	0.107514
0.076084	0.088873	0.104975	0.032564	0.086143	0.058595	0.22415	0.073659	0.07904
0.035308	0.092938	0.071559	0	0.112583	0	0	0.055616	0.307836
0.052329	0.066973	0.04307	0.035761	0.05856	0.044356	0.145551	0.055906	0.047773
0	0	0	0	0	0	0	0	0
0	0	0	0	0	0	0	0	0
0	0	0	0	0	0	0	0	0
0.055923	0.052814	0.039167	0.027393	0.039805	0.041585	0.048513	0.048294	0.047664
0.049852	0.079258	0.078538	0.248521	0.097213	0.474928	0	0.070617	0.143326
0.034133	0.035344	0.032268	0.030113	0.039282	0.037136	0.032157	0.031139	0.030689
0.171348	0.399624	0.469394	0	0.444145	0	0	0.333845	0.418587
0.169417	0.080853	0.170494	0.57284	0.208988	0.089425	0.316497	0.341512	0.349139
0.033013	0.430658	0.030814	0.117231	0.049315	0.052408	0.129422	0.226518	0.313377
0.092747	0.11638	0.111241	0.078491	0.169701	0.101441	0.12485	0.120952	0.204321
0.080261	0.099466	0.089033	0.093926	0.094053	0.070016	0.110909	0.100228	0.10754
0.058792	0.084548	0.105424	0.050945	0.08087	0.060937	0.22426	0.080659	0.059414
0.522316	0.106664	0.660214	0.68803	0.629944	0.838136	0.683307	0.67737	0.469908
0.194554	0.145486	0.194066	0.051183	0.141046	0.104724	0.139279	0.147409	0.115654
0.053668	0.669531	0.86489	0.014918	0.257076	0.029782	0.021531	0.067138	0.411138
0.042063	0.140315	0.055514	0.0369	0.046563	0.047586	0.053006	0.150716	0.051949
0.281987	0.141655	0.37619	0.35301	0.416143	0.749974	0.626732	0.333572	0.414714
0.107207	0.712057	0.964015	0.045912	0.754204	0.027199	0.029969	0.243053	0.800423
0.014624	0.041692	0.021703	0.012817	0.017753	0.013272	0.014724	0.014716	0.017052
0.11357	0.076526	0.126387	0.098408	0.09483	0.117748	0.152052	0.080883	0.132837
0.527828	0.06481	0.193824	0.915125	0.342914	0.467656	0.564167	0.683397	0.809628
0.031366	0.103829	0.024899	0.029624	0.03027	0.028945	0.030866	0.106316	0.033628
0	0	0	0	0	0	0	0	0
0.061036	0.072987	0.073625	0.051667	0.067768	0.042136	0.068133	0.046187	0.044171
0.380838	0.386952	0.202677	0.069013	0.174773	0.277115	0.45358	0.305789	0.133575
0.048107	0.031783	0.033808	0.053826	0.038171	0.044736	0.062824	0.038121	0.036005
0.079759	0.100286	0.065257	0.052729	0.084202	0.072675	0.072687	0.063163	0.058899
0.398854	0.80943	0.930766	0.094694	0.647032	0.387438	0.253047	0.546276	0.747679
0.013624	0.014116	0.012315	0.015008	0.014046	0.012881	0.012696	0.014827	0.012201
0.281079	0.156641	0.325775	0.066901	0.20959	0.081613	0.170108	0.138269	0.105359
0.071979	0.062388	0.05643	0.025548	0.055431	0.043665	0.090256	0.045353	0.070672
0.541222	0.808134	0.95521	0.172381	0.509227	0.466954	0.409278	0.534454	0.608143
0.237451	0.088316	0.098104	0.034512	0.076272	0.064012	0.179581	0.183126	0.067393
0.063285	0.055187	0.063169	0.03367	0.041525	0.054103	0.0935	0.04504	0.055241

TCGA.DW.7	TCGA.5P.A	TCGA.MH.A	TCGA.SX.A	TCGA.B1.A	TCGA.BQ.5	TCGA.P4.A	TCGA.2Z.A	TCGA.B1.A
0.060999	0.047403	0.473884	0.080695	0.042569	0.040613	0.046351	0.070375	0.050324
0.047882	0.203898	0.040951	0	0	0.044545	0.254413	0	0.039123
0.781263	0.934269	0.939227	0.947607	0.926584	0.922285	0.674304	0.929614	0.76135
0.030125	0.020484	0.018137	0.023491	0.019553	0.029432	0.02184	0.033922	0.303562
0	0	0	0	0	0	0	0	0
0.340965	0.141962	0.899059	0.083462	0.342118	0.5111	0.100365	0.273818	0.313914
0.062956	0.029293	0.483221	0.081605	0.410948	0.076227	0.070013	0.054205	0.388424
0.013866	0.012501	0.013132	0.014353	0.011468	0.019847	0.013355	0.012024	0.43174
0.083252	0.264171	0.065288	0	0	0.080255	0.281939	0	0.11492
0.106687	0.141952	0.162847	0.08632	0.100153	0.248188	0.103555	0.12391	0.236448
0.086811	0.068375	0.200951	0.079911	0.076525	0.355271	0.050661	0.124551	0.211203
0.050481	0.178784	0.026749	0	0	0.032179	0.16166	0	0.036649
0.054126	0.045975	0.082944	0.037238	0.044547	0.100457	0.040451	0.071508	0.070977
0	0	0	0	0	0	0	0	0
0	0	0	0	0	0	0	0	0
0	0	0	0	0	0	0	0	0
0.05562	0.032242	0.237696	0.068586	0.042366	0.163658	0.045232	0.079068	0.165675
0.056524	0.099084	0.039428	0	0	0.036277	0.0832	0	0.06732
0.029471	0.027267	0.038669	0.048106	0.027461	0.050638	0.029788	0.032941	0.298259
0.173662	0	0.05151	0	0	0.312813	0	0	0.167223
0.121005	0.079996	0.411673	0.204733	0.555591	0.142555	0.083763	0.315706	0.465168
0.043209	0.034981	0.210085	0.052995	0.033014	0.037936	0.030032	0.180989	0.024955
0.154674	0.095998	0.774563	0.229973	0.139455	0.154432	0.06891	0.156109	0.371468
0.092862	0.087292	0.082028	0.063653	0.101715	0.094427	0.084753	0.078585	0.391998
0.07016	0.057362	0.202007	0.083796	0.084297	0.258321	0.047978	0.121745	0.202019
0.226303	0.491885	0.806049	0.111567	0.641265	0.782286	0.642713	0.810454	0.730227
0.165509	0.07883	0.433612	0.127736	0.175772	0.313098	0.100466	0.177201	0.27835
0.035352	0.037742	0.020918	0.038291	0.152333	0.617221	0.027685	0.587717	0.017633
0.043434	0.037148	0.459215	0.058359	0.039918	0.042939	0.036664	0.039857	0.05398
0.186575	0.275408	0.766742	0.103398	0.699295	0.461401	0.132396	0.697054	0.634529
0.048042	0.293725	0.021769	0.068787	0.550548	0.562606	0.031272	0.63786	0.039932
0.018489	0.015907	0.026452	0.013185	0.01667	0.029687	0.017347	0.012612	0.434977
0.099399	0.090768	0.420162	0.150027	0.076438	0.124122	0.101727	0.114287	0.265907
0.17846	0.198491	0.71966	0.596207	0.644795	0.169326	0.187689	0.113176	0.581799
0.030236	0.025336	0.401278	0.056655	0.035746	0.028097	0.026504	0.025817	0.029195
0	0	0	0	0	0	0	0	0
0.050196	0.051298	0.057591	0.073549	0.050784	0.100455	0.075296	0.065795	0.351706
0.382609	0.16385	0.947592	0.162139	0.408179	0.382056	0.194172	0.394825	0.371019
0.045346	0.025952	0.525037	0.050651	0.134143	0.099186	0.024147	0.044556	0.342214
0.068045	0.065572	0.070203	0.077808	0.065407	0.088382	0.08009	0.066479	0.320702
0.356128	0.363758	0.322084	0.846291	0.783495	0.733914	0.237312	0.770952	0.388065
0.01621	0.013075	0.012823	0.013456	0.011865	0.01462	0.013831	0.010315	0.290794
0.175879	0.084978	0.486933	0.18637	0.157216	0.24979	0.095588	0.155549	0.402629
0.046713	0.047416	0.232836	0.070329	0.054636	0.112163	0.055053	0.092623	0.156738
0.49574	0.389352	0.511851	0.270598	0.81804	0.667795	0.404354	0.768897	0.595018
0.209194	0.061157	0.113008	0.058419	0.144895	0.257265	0.033261	0.155981	0.179534
0.051304	0.048098	0.061579	0.028224	0.057401	0.062742	0.048656	0.06213	0.067494

TCGA.G7.A	TCGA.UZ.A	TCGA.GL.A	TCGA.G7.A	TCGA.KV.A	TCGA.A4.7	TCGA.DW.5	TCGA.KV.A	TCGA.BQ.5
0.061188	0.058051	0.093747	0.080762	0.091594	0.043912	0.218854	0.042695	0.093816
0.054683	0	0.087365	0.030601	0.164513	0.036739	0	0.040331	0.038643
0.838458	0.949459	0.946522	0.91741	0.948426	0.85788	0.281654	0.76425	0.817759
0.01954	0.036974	0.030308	0.013371	0.027451	0.036319	0.026181	0.021955	0.090114
0	0	0	0	0	0	0	0	0
0.208111	0.516668	0.291901	0.068408	0.13231	0.586107	0.142792	0.466511	0.703647
0.262162	0.088759	0.077667	0.080335	0.158918	0.081298	0.113606	0.089843	0.145288
0.014645	0.014533	0.013434	0.013163	0.016946	0.012349	0.012998	0.013094	0.019483
0.064262	0	0.155214	0.058733	0.22906	0.046741	0	0.080507	0.035232
0.02639	0.168803	0.164681	0.03683	0.158092	0.236891	0.071707	0.151764	0.156259
0.129402	0.175843	0.058722	0.04924	0.075687	0.153266	0.230943	0.194276	0.256997
0.04493	0	0.052919	0.024405	0.080104	0.031275	0	0.036013	0.033487
0.113466	0.102177	0.038676	0.029264	0.053368	0.106044	0.043891	0.064666	0.084312
0	0	0	0	0	0	0	0	0
0	0	0	0	0	0	0	0	0
0	0	0	0	0	0	0	0	0
0.046768	0.084569	0.040803	0.037718	0.069093	0.144685	0.046781	0.095868	0.114801
0.049502	0	0.06421	0.059953	0.095158	0.041065	0	0.064997	0.071136
0.039837	0.055182	0.03839	0.039898	0.04466	0.043988	0.027663	0.029642	0.029324
0.042438	0	0.277917	0.089095	0.330602	0.062323	0	0.119639	0.063635
0.28253	0.113003	0.141925	0.361472	0.209692	0.157517	0.755414	0.161426	0.224769
0.071742	0.097617	0.357393	0.130372	0.307466	0.037662	0.192011	0.300354	0.042959
0.451535	0.195493	0.197056	0.068077	0.447079	0.243765	0.080696	0.169057	0.180346
0.087236	0.105891	0.098142	0.079048	0.104822	0.107829	0.097083	0.085019	0.119282
0.113956	0.158986	0.048677	0.055651	0.08739	0.150666	0.223422	0.196102	0.226798
0.753084	0.850242	0.698852	0.860664	0.609435	0.460967	0.673065	0.569971	0.319516
0.259756	0.199083	0.185567	0.076226	0.211654	0.307038	0.081881	0.236213	0.312182
0.020254	0.221753	0.446585	0.710948	0.189242	0.31059	0.01498	0.052254	0.375502
0.053093	0.066696	0.054146	0.058872	0.04971	0.047407	0.078947	0.037113	0.068462
0.464292	0.614989	0.275277	0.215571	0.281764	0.533355	0.837846	0.407437	0.357671
0.037944	0.350736	0.713521	0.879085	0.583883	0.312493	0.026388	0.114334	0.437102
0.01944	0.012579	0.01749	0.018832	0.019296	0.029065	0.013731	0.01636	0.017562
0.117695	0.112849	0.06455	0.064318	0.101834	0.124469	0.145361	0.071844	0.085248
0.844008	0.440815	0.627646	0.279852	0.675678	0.185796	0.659279	0.25734	0.258693
0.055293	0.047147	0.033793	0.058115	0.044471	0.036181	0.042477	0.029285	0.034844
0	0	0	0	0	0	0	0	0
0.045321	0.080201	0.05623	0.054493	0.069957	0.079765	0.07635	0.045102	0.068849
0.301557	0.644252	0.351725	0.120134	0.210855	0.58605	0.159524	0.551845	0.759037
0.043773	0.072745	0.037851	0.03563	0.062416	0.10081	0.062841	0.062536	0.082018
0.070633	0.086643	0.073281	0.058039	0.059962	0.102858	0.06108	0.062607	0.078205
0.309251	0.494771	0.714459	0.898401	0.616027	0.674377	0.108468	0.439641	0.621268
0.016264	0.018109	0.013367	0.01199	0.015901	0.011012	0.011306	0.01124	0.013806
0.201132	0.227045	0.158723	0.065046	0.260376	0.292652	0.113472	0.234408	0.275535
0.047597	0.07183	0.063655	0.022281	0.086771	0.157807	0.03989	0.070528	0.119809
0.505063	0.591292	0.569332	0.864887	0.478289	0.720475	0.291624	0.575031	0.661126
0.083862	0.180463	0.189856	0.037103	0.082945	0.363185	0.093073	0.248625	0.252367
0.05341	0.062768	0.058227	0.027294	0.038837	0.084787	0.035595	0.080792	0.081387

TCGA.IZ.8	TCGA.BQ.7	TCGA.BQ.5	TCGA.B1.5	TCGA.A4.A	TCGA.UZ.A	TCGA.2Z.A	TCGA.IZ.A	TCGA.UZ.A
0.063546	0.220206	0.073982	0.031177	0.057376	0.195498	0.173106	0.049352	0.083279
0.249711	0.258732	0.046943	0.031432	0	0.031258	0	0.061496	0.053295
0.207577	0.949434	0.937512	0.921567	0.956238	0.914205	0.948729	0.935742	0.806132
0.025541	0.033647	0.019085	0.05201	0.013872	0.022169	0.031572	0.034945	0.022364
0	0	0	0	0	0	0	0	0
0.064902	0.067572	0.411768	0.670756	0.180134	0.091683	0.04551	0.37306	0.384833
0.112201	0.122917	0.034772	0.076157	0.148441	0.369862	0.037641	0.033993	0.13287
0.014093	0.015992	0.015271	0.022822	0.01408	0.018124	0.010506	0.015124	0.01312
0.259078	0.390842	0.088032	0.044312	0	0.057096	0.274152	0.134104	0.143942
0.140448	0.14959	0.133387	0.195743	0.100431	0.085099	0.098553	0.106711	0.105685
0.065673	0.06992	0.053567	0.072019	0.077544	0.136585	0.061576	0.075246	0.196071
0.146601	0.153271	0.03477	0.032263	0	0.033195	0.226809	0.041674	0.049569
0.038484	0.042474	0.04853	0.066293	0.051272	0.046146	0.050637	0.044144	0.051173
0	0	0	0	0	0	0	0	0
0	0	0	0	0	0	0	0	0
0	0	0	0	0	0	0	0	0
0.029494	0.057056	0.06593	0.117006	0.036305	0.044798	0.034588	0.069257	0.133115
0.098822	0.113168	0.040893	0.041263	0	0.046024	0.135247	0.061184	0.042871
0.03282	0.034086	0.034838	0.044676	0.019931	0.027839	0.028814	0.025391	0.033698
0.4259	0.419403	0.098088	0.145423	0	0.072829	0	0.241874	0.258333
0.208819	0.230937	0.07291	0.112248	0.218528	0.522485	0.076791	0.049638	0.205701
0.252039	0.237021	0.071899	0.277839	0.069057	0.535078	0.285826	0.095354	0.070988
0.078223	0.0901	0.200717	0.186438	0.112175	0.090273	0.079937	0.152404	0.2156
0.099809	0.084225	0.098304	0.079366	0.087079	0.078328	0.084528	0.06913	0.083267
0.07666	0.081954	0.06212	0.220529	0.077399	0.152967	0.064448	0.077591	0.189454
0.297149	0.850234	0.634916	0.704358	0.473774	0.837203	0.351628	0.591044	0.766125
0.086177	0.105633	0.2558	0.276111	0.126913	0.094982	0.059043	0.184998	0.210091
0.017333	0.555844	0.651445	0.172685	0.093088	0.675912	0.105911	0.030375	0.053785
0.04259	0.151143	0.044666	0.037621	0.039868	0.116083	0.085602	0.042994	0.048085
0.1319	0.278961	0.249519	0.413489	0.212666	0.801964	0.105745	0.242648	0.573098
0.020061	0.880803	0.777946	0.213765	0.392548	0.711106	0.575474	0.277461	0.286452
0.016666	0.022154	0.015051	0.021448	0.014932	0.015821	0.012785	0.017365	0.017249
0.092734	0.101241	0.116658	0.155383	0.078796	0.16131	0.098458	0.079785	0.124271
0.62528	0.454827	0.069904	0.12382	0.773093	0.876122	0.253527	0.138753	0.354042
0.03183	0.066645	0.025396	0.038332	0.03045	0.057469	0.060909	0.029861	0.029628
0	0	0	0	0	0	0	0	0
0.050098	0.069465	0.057205	0.429448	0.046994	0.045667	0.049626	0.056678	0.076525
0.107207	0.120259	0.470952	0.627596	0.284335	0.131412	0.081847	0.413857	0.489961
0.028351	0.066583	0.041646	0.052084	0.022851	0.318914	0.020569	0.033398	0.077771
0.0807	0.074455	0.058367	0.117904	0.058934	0.06154	0.064583	0.068704	0.068485
0.146422	0.519405	0.85652	0.575655	0.466141	0.758248	0.518677	0.473303	0.470005
0.012974	0.011397	0.010813	0.025295	0.015491	0.015269	0.011416	0.013206	0.00923
0.233687	0.171118	0.251591	0.264057	0.154172	0.184147	0.107309	0.22707	0.258853
0.042428	0.054	0.036768	0.207696	0.053769	0.054474	0.04897	0.055029	0.124061
0.340544	0.366179	0.829009	0.56965	0.473422	0.752852	0.420426	0.532062	0.529777
0.045176	0.040413	0.161228	0.442469	0.094197	0.03326	0.029199	0.100335	0.201032
0.046068	0.045924	0.04928	0.082408	0.042633	0.043348	0.033696	0.059009	0.059132

TCGA.A4.8	TCGA.BQ.5	TCGA.BQ.7	TCGA.IA.A	TCGA.A4.7	TCGA.UZ.A	TCGA.IA.A	TCGA.BQ.5	TCGA.A4.8
0.059971	0.073369	0.274951	0.037607	0.051883	0.052962	0.046434	0.094787	0.215075
0.104489	0.053397	0	0	0.142659	0.18116	0.091692	0.041344	0
0.919757	0.877594	0.941558	0.941115	0.628039	0.541238	0.905453	0.936912	0.937264
0.028605	0.025654	0.028616	0.018205	0.027518	0.039003	0.024149	0.023413	0.022811
0	0	0	0	0	0	0	0	0
0.146591	0.709598	0.186233	0.193053	0.083404	0.110405	0.357966	0.422116	0.172469
0.139425	0.17536	0.620665	0.03786	0.19559	0.044368	0.033472	0.067978	0.076569
0.013785	0.021873	0.014538	0.01479	0.012737	0.016589	0.01338	0.013177	0.014518
0.207987	0.050982	0	0	0.325124	0.25939	0.096032	0.073389	0.429058
0.163528	0.203963	0.123998	0.096358	0.114434	0.104268	0.114094	0.121677	0.13352
0.127436	0.185522	0.434515	0.055747	0.0758	0.109384	0.12751	0.088109	0.063982
0.056133	0.032441	0	0	0.11105	0.08256	0.040676	0.041643	0
0.037985	0.083638	0.046026	0.040568	0.041397	0.042442	0.054067	0.064507	0.050464
0	0	0	0	0	0	0	0	0
0	0	0	0	0	0	0	0	0
0	0	0	0	0	0	0	0	0
0.033319	0.172138	0.04419	0.056941	0.051023	0.061707	0.053919	0.065155	0.042343
0.076851	0.063484	0	0	0.071571	0.073578	0.060453	0.075218	0
0.033816	0.045822	0.039139	0.0284	0.042396	0.040892	0.033111	0.028724	0.03302
0.310198	0.069004	0	0	0	0	0.149377	0	0
0.221969	0.284089	0.706769	0.0803	0.285626	0.064449	0.08352	0.149003	0.148572
0.052274	0.046978	0.463842	0.036246	0.041191	0.048846	0.05959	0.270151	0.634982
0.120324	0.341439	0.11426	0.196606	0.063782	0.131161	0.320665	0.195699	0.098007
0.108656	0.136684	0.10431	0.066023	0.097956	0.099121	0.107557	0.127168	0.084359
0.119342	0.183063	0.408281	0.059238	0.087993	0.109969	0.09509	0.085127	0.056832
0.812889	0.657547	0.847564	0.732002	0.376423	0.448711	0.541756	0.623681	0.358867
0.138894	0.420848	0.163831	0.195232	0.093208	0.106489	0.242356	0.26903	0.102214
0.048965	0.095897	0.137239	0.758793	0.0246	0.015794	0.386477	0.376851	0.249553
0.036099	0.052291	0.212431	0.035944	0.048303	0.034671	0.049553	0.045027	0.143368
0.357633	0.50472	0.818511	0.311498	0.188951	0.146244	0.341473	0.370948	0.159283
0.158672	0.287757	0.306628	0.886619	0.020162	0.024649	0.746931	0.616572	0.6143
0.015019	0.039415	0.017175	0.019012	0.01828	0.013661	0.020229	0.014271	0.015091
0.083997	0.080271	0.119167	0.181859	0.103295	0.081559	0.129958	0.087159	0.064488
0.725997	0.365701	0.907319	0.084983	0.683358	0.136932	0.114406	0.414468	0.605552
0.032211	0.031095	0.139095	0.024834	0.033605	0.035991	0.039814	0.020187	0.114167
0	0	0	0	0	0	0	0	0
0.052714	0.070509	0.049196	0.060485	0.084305	0.076184	0.103739	0.06589	0.07098
0.216516	0.725454	0.235797	0.262141	0.164759	0.16615	0.447771	0.538104	0.218519
0.055773	0.103139	0.623427	0.077426	0.044242	0.036795	0.041262	0.040084	0.02903
0.060914	0.089968	0.053141	0.073261	0.081326	0.078992	0.093742	0.078373	0.070447
0.464992	0.593605	0.708972	0.893241	0.154867	0.275566	0.72732	0.736182	0.61649
0.013344	0.013968	0.013894	0.012776	0.014605	0.012888	0.015622	0.016103	0.016472
0.256118	0.408348	0.119734	0.255803	0.14951	0.099217	0.211569	0.367186	0.119737
0.048037	0.185159	0.049158	0.059591	0.047864	0.052837	0.05851	0.069731	0.042889
0.476696	0.640009	0.737467	0.959268	0.390736	0.382152	0.607437	0.654192	0.455189
0.097975	0.375474	0.157034	0.074846	0.061898	0.045869	0.143122	0.177537	0.085804
0.062519	0.112608	0.058353	0.049618	0.04351	0.047189	0.080909	0.063828	0.051303

TCGA.Y8.A	TCGA.2Z.A	TCGA.A4.A	TCGA.IA.A	TCGA.MH.A	TCGA.F9.A	TCGA.DW.5	TCGA.BQ.5	TCGA.MH.A
0.04248	0.04092	0.064875	0.031788	0.094211	0.042807	0.03103	0.082734	0.036116
0.026449	0.03885	0.072544	0	0	0	0.174764	0	0.047275
0.871407	0.775421	0.648237	0.927729	0.94271	0.875245	0.920932	0.902377	0.79377
0.013037	0.0267	0.035999	0.021291	0.027532	0.021362	0.024536	0.028368	0.029512
0	0	0	0	0	0	0	0	0
0.647621	0.391866	0.187886	0.184966	0.129549	0.308961	0.124156	0.508157	0.298463
0.087024	0.1022	0.064604	0.173858	0.035612	0.065587	0.368876	0.205045	0.146631
0.008241	0.013111	0.019746	0.014073	0.014245	0.014756	0.016581	0.01387	0.015309
0.06568	0.098996	0.200186	0	0	0	0.161579	0	0.09789
0.155164	0.106664	0.29605	0.187964	0.064597	0.112016	0.132132	0.136025	0.182929
0.104832	0.165042	0.165805	0.120635	0.100477	0.061758	0.125338	0.130953	0.082561
0.017853	0.030262	0.799599	0	0	0.319844	0.057726	0	0.041406
0.058062	0.038788	0.049617	0.047767	0.045358	0.057168	0.05445	0.09517	0.03646
0	0	0	0	0	0	0	0	0
0	0	0	0	0	0	0	0	0
0	0	0	0	0	0	0	0	0
0.085437	0.0689	0.061939	0.071156	0.040162	0.203361	0.054496	0.090663	0.03605
0.019966	0.052962	0.817113	0	0	0.393178	0.048493	0	0.063412
0.027484	0.029482	0.033499	0.027636	0.038844	0.66835	0.045993	0.055225	0.027682
0.060647	0.232122	0.904925	0	0	0	0	0	0.222328
0.160181	0.191739	0.109982	0.183971	0.102766	0.20619	0.414503	0.343322	0.21076
0.036638	0.051736	0.044208	0.032649	0.124332	0.029784	0.038575	0.138536	0.02678
0.429098	0.111015	0.171421	0.557341	0.175115	0.732813	0.176073	0.280468	0.10039
0.064277	0.077983	0.100577	0.068592	0.090624	0.086248	0.093508	0.094727	0.080382
0.099913	0.168721	0.142416	0.139685	0.096404	0.062014	0.139123	0.145487	0.076718
0.653375	0.602958	0.668442	0.751236	0.382501	0.838654	0.602181	0.750413	0.490032
0.295795	0.141639	0.254013	0.335802	0.098742	0.307325	0.242156	0.27321	0.168927
0.313488	0.075396	0.044384	0.081675	0.679943	0.027203	0.019044	0.05183	0.091384
0.03529	0.042406	0.050923	0.037978	0.052124	0.031747	0.039243	0.071622	0.041876
0.449753	0.329281	0.387054	0.738137	0.140867	0.531653	0.302723	0.697204	0.227905
0.450412	0.161383	0.063446	0.146708	0.879344	0.042241	0.028293	0.071199	0.095833
0.009583	0.016298	0.02066	0.019074	0.028671	0.530623	0.014777	0.016262	0.019928
0.248824	0.080425	0.141252	0.215535	0.086945	0.539831	0.160608	0.245246	0.070898
0.201301	0.165895	0.091159	0.574794	0.215198	0.595702	0.81753	0.482293	0.524519
0.023449	0.030042	0.039091	0.029021	0.042301	0.020193	0.026107	0.027421	0.024342
0	0	0	0	0	0	0	0	0
0.050645	0.065021	0.071839	0.046974	0.055026	0.577535	0.046975	0.073253	0.063098
0.774341	0.463321	0.229935	0.240109	0.210511	0.43357	0.223146	0.585134	0.347215
0.054762	0.036962	0.048153	0.090868	0.038088	0.034224	0.060925	0.1731	0.028147
0.045751	0.079808	0.083514	0.059459	0.054887	0.543538	0.071918	0.063356	0.071218
0.641544	0.523276	0.339768	0.39829	0.825769	0.245587	0.231085	0.526004	0.480869
0.007181	0.01439	0.015194	0.014288	0.016762	0.014577	0.013894	0.013689	0.013548
0.303991	0.13721	0.471497	0.439533	0.092283	0.382559	0.338897	0.221234	0.20423
0.085608	0.054048	0.081452	0.144606	0.059729	0.253433	0.071259	0.094159	0.045505
0.759874	0.58092	0.422946	0.44848	0.820247	0.407366	0.426315	0.627786	0.549758
0.244903	0.153991	0.068982	0.079497	0.102147	0.091267	0.136123	0.355023	0.112008
0.06317	0.077818	0.050785	0.039438	0.040581	0.047326	0.058047	0.094617	0.045628

TCGA.BQ.7	TCGA.UZ.A	TCGA.A4.7	TCGA.UZ.A	TCGA.PJ.A	TCGA.A4.A	TCGA.B1.7	TCGA.BQ.7	TCGA.J7.6
0.055944	0.056638	0.074529	0.044309	0.111755	0.071046	0.060288	0.05764	0.052774
0.055911	0	0.061163	0.124018	0	0	0	0	0.054321
0.95744	0.934326	0.920303	0.391454	0.921711	0.936309	0.914524	0.924108	0.827439
0.027635	0.025449	0.028808	0.017804	0.017206	0.015451	0.023283	0.029617	0.025184
0	0	0	0	0	0	0	0	0
0.362773	0.133921	0.293772	0.104853	0.243543	0.054777	0.223985	0.270449	0.278918
0.051505	0.136941	0.279165	0.032045	0.060403	0.051176	0.119341	0.236644	0.070245
0.014095	0.016505	0.012512	0.012748	0.014082	0.015163	0.014954	0.015694	0.016264
0.090554	0	0.121875	0.219191	0	0.468648	0	0	0.082347
0.175544	0.068015	0.131206	0.051466	0.049459	0.056822	0.133733	0.161978	0.143564
0.082882	0.157323	0.077224	0.071478	0.118703	0.048532	0.080377	0.086898	0.078622
0.044721	0	0.064881	0.083824	0.406244	0	0	0	0.060956
0.05876	0.048925	0.054352	0.043169	0.077671	0.042698	0.050623	0.047953	0.049278
0	0	0	0	0	0	0	0	0
0	0	0	0	0	0	0	0	0
0	0	0	0	0	0	0	0	0
0.087601	0.037842	0.046893	0.047587	0.079119	0.030278	0.051151	0.055888	0.098328
0.064456	0	0.04851	0.06313	0	0.472992	0	0	0.066897
0.045419	0.02377	0.03854	0.027405	0.038672	0.027905	0.025564	0.049097	0.03731
0.112177	0	0.25852	0.41185	0	0	0	0	0.290174
0.100177	0.191368	0.284251	0.083732	0.159339	0.043417	0.175586	0.221287	0.127841
0.083649	0.028627	0.061753	0.037077	0.350675	0.564354	0.089326	0.070422	0.042899
0.330198	0.08108	0.298362	0.07503	0.203831	0.097273	0.094328	0.310419	0.117285
0.096624	0.055297	0.101761	0.088061	0.098349	0.090741	0.099839	0.118422	0.100241
0.079025	0.182702	0.098913	0.064375	0.104072	0.05811	0.071359	0.103914	0.080003
0.745437	0.864844	0.769369	0.094822	0.778137	0.936146	0.467678	0.624245	0.327405
0.192562	0.09572	0.219581	0.088696	0.217571	0.05545	0.135449	0.223743	0.155797
0.067122	0.690749	0.043029	0.017308	0.199735	0.891946	0.034767	0.054527	0.11263
0.039964	0.031327	0.050961	0.031767	0.047746	0.052314	0.042615	0.049061	0.054744
0.485865	0.494618	0.509117	0.115267	0.591887	0.881625	0.186409	0.602132	0.192271
0.411389	0.827768	0.317113	0.027682	0.260646	0.940067	0.542857	0.168747	0.082133
0.01864	0.013337	0.018473	0.015227	0.02224	0.01771	0.017292	0.036547	0.015333
0.115527	0.087257	0.132452	0.077002	0.112446	0.108334	0.110707	0.125008	0.11957
0.508666	0.545127	0.807565	0.144788	0.535216	0.085563	0.531166	0.588515	0.272143
0.023974	0.024885	0.027848	0.029591	0.038594	0.033669	0.025871	0.027272	0.031554
0	0	0	0	0	0	0	0	0
0.066752	0.051222	0.063693	0.044937	0.067083	0.041263	0.060961	0.067414	0.053697
0.42596	0.201833	0.36796	0.162985	0.314063	0.115831	0.25522	0.320233	0.355563
0.038531	0.11062	0.074358	0.028539	0.068423	0.054549	0.033181	0.077512	0.05944
0.075449	0.071814	0.087798	0.061154	0.075333	0.050614	0.062187	0.061773	0.072775
0.515981	0.80784	0.52198	0.230257	0.751808	0.932656	0.366369	0.465433	0.428163
0.011094	0.017143	0.011176	0.014495	0.016219	0.015352	0.01418	0.011525	0.013481
0.193845	0.107652	0.271559	0.103497	0.121517	0.082722	0.1356	0.263143	0.19655
0.076567	0.042836	0.07238	0.055498	0.080041	0.032595	0.068848	0.06639	0.09484
0.495499	0.893902	0.617442	0.387877	0.736033	0.918048	0.439661	0.516752	0.509817
0.190102	0.054147	0.219953	0.035969	0.147919	0.036956	0.084171	0.14432	0.156638
0.07227	0.04046	0.061384	0.044553	0.066664	0.034223	0.050627	0.059903	0.050218

TCGA.BQ.7	TCGA.GL.7	TCGA.A4.A	TCGA.5P.A	TCGA.A4.A	TCGA.B9.5	TCGA.G7.6	TCGA.2Z.A	TCGA.2Z.A
0.03452	0.046531	0.15124	0.040619	0.0396	0.050296	0.066495	0.048147	0.044402
0.051235	0	0	0.073882	0	0.069125	0.031389	0.095332	0.032949
0.931707	0.948218	0.94068	0.533809	0.877762	0.859745	0.93834	0.913126	0.565187
0.021418	0.028367	0.020363	0.036652	0.019505	0.039718	0.022404	0.02361	0.021157
0	0	0	0	0	0	0	0	0
0.403818	0.238552	0.232799	0.94402	0.104275	0.26982	0.303997	0.172663	0.181027
0.075951	0.051787	0.311007	0.070702	0.175951	0.049993	0.033673	0.028514	0.077472
0.011861	0.013862	0.015438	0.017759	0.015766	0.013724	0.010642	0.013236	0.011952
0.103456	0	0	0.231578	0	0.075721	0.050636	0.272619	0.057642
0.140755	0.133372	0.07922	0.148607	0.127674	0.144189	0.119281	0.093953	0.078388
0.083471	0.077982	0.050466	0.51101	0.065444	0.068789	0.05403	0.061676	0.233656
0.029641	0	0.400042	0.070949	0	0.039882	0.025611	0.055061	0.025934
0.074909	0.044779	0.046065	0.058009	0.045592	0.045963	0.055719	0.035952	0.054231
0	0	0	0	0	0	0	0	0
0	0	0	0	0	0	0	0	0
0	0	0	0	0	0	0	0	0
0.066465	0.035707	0.078226	0.355358	0.023404	0.10981	0.062717	0.058774	0.048322
0.055786	0	0	0.065834	0	0.050837	0.037302	0.050191	0.029669
0.032872	0.041213	0.028799	0.060642	0.032617	0.034588	0.039119	0.026115	0.026704
0.131575	0	0	0.429817	0	0.269684	0.045246	0.327624	0.081308
0.103757	0.047132	0.405369	0.503706	0.307987	0.088142	0.084756	0.076205	0.108461
0.031909	0.039509	0.424543	0.518347	0.344153	0.027563	0.077318	0.046869	0.07683
0.167205	0.094231	0.084848	0.80917	0.198213	0.175604	0.05988	0.079917	0.088614
0.109789	0.082914	0.086634	0.073717	0.089903	0.094343	0.080404	0.080326	0.081646
0.101188	0.066987	0.0534	0.551029	0.068979	0.123372	0.072569	0.070636	0.210331
0.35515	0.258349	0.647314	0.488562	0.671654	0.565943	0.228686	0.148955	0.059061
0.224921	0.181657	0.131479	0.334054	0.15176	0.237454	0.106807	0.106654	0.056069
0.247984	0.079018	0.15712	0.017136	0.042261	0.053995	0.037384	0.032222	0.020229
0.033781	0.046036	0.056898	0.042213	0.039518	0.038062	0.05352	0.035026	0.038296
0.35084	0.112452	0.215632	0.718311	0.282487	0.333082	0.203783	0.123041	0.107129
0.436209	0.286246	0.466515	0.018929	0.090116	0.107062	0.695499	0.035802	0.017371
0.015911	0.013819	0.01934	0.014412	0.01971	0.020495	0.014276	0.017003	0.011421
0.164627	0.082884	0.081865	0.087377	0.092769	0.130254	0.087942	0.08445	0.068257
0.269592	0.079776	0.766024	0.83118	0.781342	0.233566	0.056901	0.098374	0.099803
0.026327	0.033317	0.036857	0.034356	0.023622	0.021877	0.027338	0.031827	0.032188
0	0	0	0	0	0	0	0	0
0.07114	0.058224	0.063118	0.064377	0.057729	0.043828	0.0712	0.055846	0.057937
0.437962	0.312647	0.266027	0.946284	0.186508	0.406027	0.319144	0.234458	0.508028
0.079646	0.043197	0.029321	0.029414	0.034929	0.03157	0.044806	0.028723	0.031007
0.072164	0.059569	0.058699	0.085771	0.076993	0.059163	0.069202	0.068834	0.074066
0.594413	0.388171	0.622853	0.186883	0.324275	0.515543	0.273869	0.329431	0.112833
0.011294	0.01032	0.01365	0.012926	0.01641	0.022286	0.014536	0.015395	0.01186
0.235007	0.159649	0.138063	0.091837	0.170108	0.232547	0.116292	0.138315	0.057752
0.092012	0.057312	0.068434	0.437693	0.040237	0.104255	0.069728	0.044902	0.055465
0.567891	0.480856	0.524059	0.389568	0.421759	0.401936	0.479339	0.421406	0.310564
0.237917	0.112667	0.057361	0.052731	0.089263	0.162839	0.135567	0.093649	0.047238
0.080136	0.046463	0.048992	0.047994	0.036363	0.086177	0.063955	0.054893	0.043447

TCGA.BQ.5	TCGA.A4.8	TCGA.IZ.8	TCGA.A4.8	TCGA.B9.A	TCGA.A4.7	TCGA.B9.A	TCGA.2Z.A	TCGA.2Z.A
0.05021	0.061291	0.056135	0.038718	0.052946	0.732969	0.532851	0.050709	0.061944
0	0.070128	0.144519	0.037467	0.109968	0	0.029509	0.250197	0.050775
0.951608	0.943595	0.949555	0.887892	0.674707	0.935694	0.918945	0.599611	0.930069
0.034743	0.020841	0.029678	0.032812	0.020915	0.021611	0.020833	0.019211	0.0306
0	0	0	0	0	0	0	0	0
0.125868	0.166755	0.113322	0.395075	0.166023	0.059581	0.365935	0.069074	0.293412
0.043429	0.280038	0.079759	0.060792	0.118615	0.733789	0.143889	0.222798	0.084156
0.01578	0.015549	0.015048	0.01466	0.014687	0.016856	0.01067	0.014958	0.01765
0	0.123417	0.308116	0.029819	0.16845	0	0.045025	0.296771	0.16334
0.087171	0.140512	0.118892	0.192327	0.135518	0.074769	0.075911	0.085738	0.271568
0.041377	0.085554	0.218308	0.132637	0.052244	0.163257	0.114754	0.054101	0.071762
0	0.052469	0.089951	0.026148	0.072901	0	0.04056	0.201626	0.058061
0.04442	0.04651	0.038618	0.069481	0.039942	0.050585	0.049724	0.040578	0.03865
0	0	0	0	0	0	0	0	0
0	0	0	0	0	0	0	0	0
0	0	0	0	0	0	0	0	0
0.04076	0.061062	0.039234	0.119571	0.038336	0.041627	0.049087	0.036048	0.162988
0	0.07633	0.100671	0.058065	0.07056	0	0.023087	0.105995	0.043634
0.041717	0.038589	0.035181	0.036219	0.043208	0.023989	0.025835	0.039112	0.126923
0	0.364623	0.424731	0.055072	0.328345	0	0.077885	0	0.299299
0.071378	0.377122	0.140558	0.1093	0.214849	0.769377	0.187218	0.327929	0.121445
0.022006	0.028369	0.164718	0.028606	0.036177	0.590027	0.742722	0.033515	0.044181
0.087597	0.085873	0.112349	0.27609	0.138971	0.16091	0.169353	0.093346	0.493107
0.100252	0.081523	0.104452	0.104478	0.075777	0.091555	0.087265	0.088653	0.085083
0.093571	0.066666	0.203751	0.11845	0.046279	0.158573	0.148336	0.048426	0.092745
0.557793	0.895186	0.818677	0.496215	0.462235	0.819768	0.862294	0.739954	0.487731
0.107571	0.130475	0.094467	0.286467	0.176345	0.086465	0.131249	0.073889	0.476224
0.196089	0.50603	0.80423	0.329548	0.018185	0.297929	0.654447	0.020975	0.253063
0.044679	0.051132	0.041754	0.036128	0.039181	0.748423	0.523208	0.045404	0.03696
0.320863	0.821278	0.724588	0.278883	0.169807	0.455215	0.633231	0.516928	0.319153
0.936682	0.867839	0.865681	0.353925	0.02576	0.793153	0.708544	0.061026	0.402334
0.012885	0.016198	0.016424	0.01556	0.027572	0.014665	0.014027	0.01616	0.256348
0.102609	0.088643	0.073954	0.082223	0.074146	0.157179	0.203841	0.08356	0.381935
0.064177	0.744267	0.260938	0.137557	0.690771	0.908633	0.791327	0.850588	0.517034
0.027336	0.0265	0.026433	0.025725	0.031374	0.65676	0.366812	0.031526	0.031459
0	0	0	0	0	0	0	0	0
0.063489	0.054053	0.062378	0.057375	0.051994	0.045745	0.046409	0.060811	0.151017
0.226217	0.205906	0.192712	0.4582	0.226521	0.130844	0.42162	0.107885	0.314709
0.033611	0.039655	0.111765	0.053682	0.039131	0.089453	0.254105	0.023136	0.034107
0.070712	0.067379	0.080925	0.080558	0.063872	0.056914	0.061742	0.068819	0.125622
0.548374	0.809382	0.881601	0.648912	0.340564	0.675768	0.849508	0.248396	0.702372
0.01453	0.015911	0.0149	0.015165	0.01273	0.016477	0.012469	0.013912	0.012472
0.115073	0.13975	0.111601	0.289943	0.236698	0.076075	0.204154	0.073139	0.508658
0.061196	0.046791	0.044872	0.111317	0.052525	0.041676	0.044152	0.030475	0.190541
0.331013	0.73543	0.885005	0.658566	0.470868	0.35641	0.853347	0.321423	0.673698
0.056777	0.122698	0.050928	0.210128	0.095865	0.058885	0.152562	0.046642	0.153864
0.043236	0.04872	0.04647	0.094529	0.047269	0.045847	0.053338	0.046074	0.057359

TCGA.UZ.A	TCGA.DW.7	TCGA.A4.A	TCGA.J7.A	TCGA.HE.A	TCGA.B9.A	TCGA.MH.A	TCGA.HE.A	TCGA.HE.7
0.040135	0.048523	0.325556	0.031379	0.103915	0.041347	0.099616	0.037031	0.064778
0	0.303475	0.024066	0.033843	0.030115	0.193695	0.06541	0.08526	0
0.946463	0.68415	0.931389	0.930794	0.895956	0.940334	0.889638	0.617512	0.922287
0.026691	0.021339	0.031774	0.020152	0.018431	0.031644	0.18339	0.034277	0.025341
0	0	0	0	0	0	0	0	0
0.06137	0.062407	0.241327	0.91066	0.564005	0.118544	0.291926	0.215981	0.315945
0.023192	0.030422	0.134799	0.040001	0.053938	0.35357	0.351387	0.034614	0.074226
0.012493	0.012019	0.0163	0.012708	0.014775	0.016005	0.150732	0.017249	0.013418
0	0.350968	0.036478	0.055125	0.039832	0.314853	0.112004	0.200259	0
0.093016	0.121004	0.197777	0.079723	0.134183	0.266105	0.292419	0.170521	0.282313
0.041055	0.160932	0.08463	0.122086	0.080598	0.114556	0.139011	0.06304	0.090951
0.270007	0.234788	0.02395	0.025287	0.029696	0.088997	0.057175	0.085796	0
0.035647	0.039164	0.044862	0.049937	0.075361	0.051195	0.061885	0.043446	0.05561
0	0	0	0	0	0	0	0	0
0	0	0	0	0	0	0	0	0
0	0	0	0	0	0	0	0	0
0.046947	0.029992	0.078118	0.042697	0.096255	0.075535	0.244322	0.035698	0.123727
0.137972	0.218734	0.04633	0.049332	0.082868	0.064127	0.063953	0.072953	0
0.024627	0.030579	0.024622	0.033276	0.032741	0.434278	0.112547	0.032839	0.048096
0	0	0.057801	0.079547	0.066567	0.449516	0.285642	0.378625	0
0.057596	0.129767	0.214509	0.092803	0.140243	0.378211	0.334807	0.079507	0.08883
0.031181	0.046121	0.441356	0.03372	0.526759	0.061913	0.524037	0.032771	0.026432
0.096596	0.115584	0.173411	0.460934	0.211454	0.728948	0.496722	0.1356	0.251906
0.062316	0.088705	0.103207	0.079713	0.097853	0.123056	0.11941	0.101119	0.101598
0.062954	0.157815	0.07979	0.12795	0.068058	0.118026	0.148283	0.071145	0.104681
0.59446	0.074097	0.592522	0.135909	0.633191	0.839151	0.793394	0.230837	0.146801
0.076791	0.086488	0.180001	0.18272	0.239101	0.48636	0.377477	0.143673	0.326286
0.028262	0.016262	0.125126	0.023621	0.109113	0.792163	0.04245	0.045751	0.389155
0.026769	0.050049	0.223938	0.031896	0.046243	0.050846	0.073124	0.043862	0.046233
0.160813	0.069458	0.212551	0.042161	0.370558	0.794065	0.761795	0.131909	0.115446
0.816673	0.025045	0.423836	0.303142	0.240441	0.838831	0.147602	0.05529	0.751211
0.013743	0.014115	0.031923	0.016554	0.026099	0.514564	0.209059	0.016381	0.017469
0.084553	0.097301	0.102588	0.091847	0.094228	0.631364	0.438859	0.08792	0.15676
0.050105	0.227762	0.378642	0.170068	0.352851	0.894973	0.640649	0.136081	0.068398
0.025324	0.026575	0.172533	0.029114	0.041226	0.029456	0.029543	0.027146	0.025454
0	0	0	0	0	0	0	0	0
0.05202	0.077291	0.057142	0.067628	0.057729	0.450805	0.205271	0.063627	0.068831
0.118956	0.107527	0.283197	0.9052	0.615011	0.15875	0.360178	0.261418	0.39671
0.02215	0.029369	0.047089	0.025368	0.029461	0.211026	0.085295	0.031524	0.032622
0.061151	0.056004	0.084324	0.075367	0.063192	0.374078	0.164773	0.079206	0.075269
0.155341	0.117999	0.446027	0.207753	0.619937	0.863431	0.406841	0.469217	0.826817
0.012285	0.011863	0.014882	0.014518	0.015331	0.012916	0.025282	0.015347	0.012801
0.05372	0.18106	0.197532	0.299794	0.238574	0.507248	0.470161	0.121639	0.515682
0.051099	0.05293	0.07396	0.04752	0.09479	0.114193	0.272536	0.046849	0.14473
0.315593	0.256684	0.477429	0.156861	0.643773	0.864538	0.554167	0.512262	0.816051
0.043799	0.049653	0.147723	0.041948	0.237425	0.101787	0.189111	0.092944	0.09094
0.033702	0.039723	0.061294	0.036855	0.085575	0.045492	0.052423	0.053137	0.054912

TCGA.SX.A	TCGA.G7.A	TCGA.IA.A	TCGA.GL.A	TCGA.P4.A	TCGA.Y8.A	TCGA.G7.A	TCGA.P4.A	TCGA.BQ.7
0.08298	0.045781	0.053246	0.12039	0.036547	0.067244	0.34431	0.513603	0.044915
0.101797	0.088645	0.058652	0.127311	0.041457	0.093577	0.31816	0.027123	0.041787
0.93335	0.88345	0.906336	0.921702	0.642622	0.925613	0.936915	0.920888	0.560669
0.027945	0.01758	0.039503	0.020249	0.034725	0.028555	0.022397	0.037326	0.032952
0	0	0	0	0	0	0	0	0
0.206905	0.17875	0.448113	0.139683	0.471355	0.243912	0.030183	0.097837	0.433943
0.208983	0.114366	0.057495	0.474129	0.028896	0.056246	0.126109	0.058317	0.048091
0.019864	0.015863	0.014246	0.010817	0.016721	0.013879	0.012794	0.012901	0.01059
0.213674	0.287121	0.085537	0.192242	0.041505	0.210069	0	0.06496	0.101264
0.153771	0.056188	0.138921	0.075573	0.133382	0.035188	0.056542	0.061615	0.125971
0.097154	0.118734	0.119251	0.205018	0.166829	0.056189	0.068792	0.186181	0.068979
0.066869	0.076773	0.035168	0.161963	0.022019	0.126224	0.247847	0.029404	0.025758
0.063222	0.044967	0.054327	0.044307	0.050134	0.058561	0.044223	0.037815	0.050459
0	0	0	0	0	0	0	0	0
0	0	0	0	0	0	0	0	0
0	0	0	0	0	0	0	0	0
0.082401	0.024566	0.109465	0.024233	0.037008	0.02019	0.026577	0.022267	0.070663
0.078058	0.066027	0.057016	0.06188	0.034759	0.050362	0.161462	0.037518	0.03043
0.047089	0.031012	0.03343	0.033049	0.028964	0.029609	0.042382	0.037222	0.035742
0.338497	0.355837	0.133126	0	0.0665	0	0	0.074668	0.114241
0.264659	0.218912	0.063771	0.732083	0.035341	0.104821	0.207394	0.090811	0.087619
0.045453	0.033201	0.067539	0.084516	0.152062	0.361056	0.460777	0.931101	0.070305
0.202125	0.105894	0.189379	0.106982	0.205988	0.203097	0.055158	0.090655	0.125743
0.073971	0.092846	0.086974	0.095805	0.093103	0.08027	0.068999	0.097223	0.10554
0.109377	0.128258	0.126587	0.224059	0.166296	0.06461	0.074726	0.19911	0.05706
0.634722	0.220994	0.632851	0.895551	0.194629	0.822543	0.870102	0.852686	0.446965
0.183657	0.098938	0.226428	0.068621	0.293133	0.118254	0.065905	0.069149	0.226044
0.096368	0.057516	0.089827	0.014358	0.183658	0.047	0.096795	0.745183	0.071794
0.054593	0.032601	0.054549	0.050379	0.033085	0.044269	0.224887	0.473739	0.047571
0.288596	0.195446	0.356818	0.844503	0.14385	0.654783	0.554717	0.624952	0.344795
0.532926	0.746181	0.103631	0.190275	0.220143	0.339441	0.457528	0.874179	0.141792
0.019583	0.015209	0.028273	0.009998	0.01659	0.014235	0.021186	0.011943	0.015184
0.110102	0.068431	0.105673	0.168714	0.062367	0.387569	0.059844	0.095855	0.094698
0.600398	0.399281	0.085373	0.922106	0.048281	0.220649	0.622369	0.152959	0.079718
0.055883	0.024334	0.032036	0.03894	0.027682	0.02689	0.183438	0.339756	0.029507
0	0	0	0	0	0	0	0	0
0.064228	0.046313	0.067872	0.047673	0.044727	0.058226	0.073109	0.051699	0.072809
0.303226	0.273774	0.493818	0.198327	0.494397	0.32713	0.069237	0.161219	0.468934
0.05692	0.051683	0.069274	0.5863	0.028357	0.073322	0.077488	0.043275	0.03759
0.064266	0.074217	0.070699	0.065874	0.083105	0.051991	0.075078	0.067943	0.066959
0.386384	0.514477	0.529991	0.643448	0.511113	0.636021	0.776409	0.879475	0.443625
0.014882	0.01476	0.014598	0.0106	0.014098	0.017237	0.012945	0.014623	0.009333
0.128275	0.190502	0.181832	0.140877	0.184651	0.041146	0.104354	0.087284	0.163352
0.066616	0.033356	0.078375	0.03748	0.036128	0.026839	0.032908	0.06543	0.067849
0.53378	0.336762	0.571538	0.579889	0.558854	0.473443	0.444573	0.893022	0.604944
0.087673	0.065325	0.149555	0.070466	0.145103	0.060288	0.028	0.064445	0.173326
0.056758	0.04033	0.063344	0.027784	0.065966	0.047313	0.031839	0.057537	0.083419

TCGA.V9.A	TCGA.UZ.A	TCGA.UZ.A	TCGA.G7.6	TCGA.IA.A	TCGA.J7.8	TCGA.A4.7	TCGA.SX.A	TCGA.2K.A
0.102234	0.037292	0.052348	0.144909	0.04672	0.0314	0.041551	0.042959	0.04798
0.05441	0.058528	0.211896	0.062836	0.061156	0	0	0.060822	0.039093
0.941087	0.930036	0.668009	0.94861	0.939402	0.875105	0.922736	0.934578	0.852461
0.027516	0.021579	0.022569	0.02357	0.015133	0.021192	0.029345	0.019367	0.019456
0	0	0	0	0	0	0	0	0
0.39607	0.317505	0.08873	0.350417	0.182772	0.581034	0.879169	0.238315	0.621014
0.050715	0.054967	0.021492	0.129947	0.114321	0.056968	0.097334	0.045179	0.024227
0.014755	0.01229	0.015768	0.016287	0.012256	0.01427	0.013728	0.013157	0.010824
0.064292	0.162479	0.252023	0.106156	0.143442	0.377813	0	0.250051	0.084594
0.149417	0.172866	0.083936	0.140662	0.095902	0.222226	0.192045	0.058683	0.159649
0.080165	0.07445	0.034178	0.071515	0.066399	0.146658	0.1209	0.07465	0.056471
0.024056	0.039108	0.117188	0.04894	0.052575	0	0	0.08686	0.024634
0.056847	0.045973	0.037203	0.050883	0.052177	0.137366	0.098466	0.046823	0.055506
0	0	0	0	0	0	0	0	0
0	0	0	0	0	0	0	0	0
0	0	0	0	0	0	0	0	0
0.081984	0.077697	0.021492	0.06436	0.04414	0.139863	0.145637	0.02873	0.072189
0.0407	0.041302	0.078984	0.059719	0.061562	0	0	0.076781	0.043738
0.046169	0.033106	0.024844	0.031226	0.028125	0.039485	0.043209	0.029917	0.04873
0.093974	0.24507	0	0.146292	0.270156	0	0	0.354659	0.075948
0.075121	0.112383	0.040439	0.15864	0.171726	0.168615	0.146572	0.074407	0.043147
0.117181	0.035213	0.024684	0.122926	0.03873	0.030669	0.034517	0.049011	0.097811
0.301874	0.398948	0.072296	0.168636	0.185611	0.186834	0.213953	0.101669	0.228002
0.107503	0.08215	0.079926	0.108752	0.099146	0.091396	0.093094	0.079071	0.094034
0.093824	0.076149	0.044555	0.06454	0.065711	0.159618	0.120821	0.053817	0.055725
0.63302	0.642777	0.390114	0.67046	0.775989	0.690215	0.330314	0.440194	0.467753
0.22904	0.330324	0.082134	0.207018	0.180861	0.329119	0.25168	0.118009	0.247159
0.326003	0.089936	0.015315	0.321195	0.031575	0.476715	0.295246	0.206407	0.090728
0.053923	0.033958	0.035472	0.092034	0.03762	0.0345	0.044114	0.039223	0.032808
0.419666	0.318368	0.09869	0.34488	0.417908	0.415155	0.30701	0.160843	0.28207
0.550121	0.22291	0.027803	0.753832	0.178274	0.57282	0.416421	0.617055	0.132242
0.026769	0.022882	0.015512	0.020754	0.019167	0.018881	0.023107	0.015001	0.032804
0.103055	0.147644	0.093711	0.101141	0.118188	0.096842	0.096806	0.072266	0.100942
0.152523	0.536426	0.057498	0.460976	0.676317	0.231708	0.158595	0.490053	0.145804
0.038322	0.029363	0.024075	0.055412	0.025129	0.022521	0.023692	0.030152	0.030018
0	0	0	0	0	0	0	0	0
0.059638	0.071955	0.044945	0.064466	0.056225	0.055476	0.064033	0.04472	0.060972
0.436586	0.370022	0.167976	0.408926	0.231347	0.593932	0.878086	0.288397	0.684164
0.052853	0.024524	0.025625	0.057312	0.03913	0.060051	0.067253	0.034144	0.045412
0.087511	0.069962	0.05441	0.060955	0.059947	0.071726	0.068999	0.080415	0.07677
0.742434	0.589601	0.206215	0.72545	0.429827	0.756379	0.739743	0.638749	0.522006
0.014503	0.009927	0.012556	0.013943	0.012603	0.015162	0.010449	0.013604	0.010303
0.196595	0.37426	0.086679	0.272699	0.174147	0.283721	0.296063	0.132164	0.228935
0.095792	0.10207	0.024096	0.087204	0.050636	0.133877	0.149573	0.045039	0.068434
0.642652	0.690124	0.379098	0.578935	0.527532	0.731408	0.751416	0.513592	0.618217
0.160825	0.130221	0.039955	0.197847	0.10124	0.346407	0.320664	0.127806	0.155291
0.08275	0.057028	0.043134	0.071335	0.045789	0.097184	0.08333	0.049836	0.084937

TCGA.HE.A	TCGA.SX.A	TCGA.DW.7	TCGA.IA.A	TCGA.5P.A	TCGA.GL.A	TCGA.A4.A	TCGA.5P.A	TCGA.2Z.A
0.051233	0.062431	0.041396	0.045337	0.044423	0.250596	0.044363	0.045875	0.047064
0.051398	0.18344	0.045356	0.059586	0	0	0.068766	0.046978	0.100683
0.941839	0.748295	0.914612	0.887255	0.906329	0.90492	0.504589	0.920524	0.793983
0.048982	0.021093	0.028183	0.021276	0.01976	0.012356	0.017592	0.017334	0.027161
0	0	0	0	0	0	0	0	0
0.66329	0.111026	0.640948	0.234367	0.368874	0.089675	0.22411	0.427037	0.258327
0.065044	0.176658	0.031979	0.045927	0.089148	0.051531	0.045708	0.032565	0.128375
0.01968	0.012442	0.01454	0.019814	0.017281	0.010369	0.013148	0.016199	0.015093
0.153377	0.280558	0.069095	0.130919	0	0	0.158374	0.125832	0.224178
0.244388	0.102564	0.186825	0.064322	0.070253	0.031059	0.104874	0.118922	0.150691
0.088875	0.082174	0.074283	0.09128	0.211933	0.060881	0.052755	0.04619	0.07289
0.04438	0.109815	0.026379	0.060451	0	0	0.060865	0.042	0.055052
0.064599	0.053912	0.068554	0.046102	0.061717	0.036715	0.049254	0.057088	0.057385
0	0	0	0	0	0	0	0	0
0	0	0	0	0	0	0	0	0
0	0	0	0	0	0	0	0	0
0.094934	0.074732	0.09216	0.085415	0.031248	0.020681	0.03122	0.047565	0.083663
0.059253	0.070815	0.054695	0.053288	0	0	0.074846	0.047185	0.055718
0.036104	0.046213	0.040222	0.024792	0.028113	0.032173	0.028639	0.032213	0.029044
0.193331	0.34651	0.094279	0.174325	0	0	0.270927	0.206481	0.290061
0.100628	0.195116	0.094898	0.09625	0.366598	0.138449	0.107383	0.042636	0.149477
0.057616	0.045204	0.029665	0.026009	0.025462	0.308081	0.02814	0.040712	0.169656
0.341848	0.160413	0.228196	0.097532	0.097369	0.187467	0.065502	0.218437	0.232634
0.106954	0.073209	0.085429	0.064331	0.07252	0.087366	0.078879	0.074675	0.087978
0.074726	0.090293	0.103366	0.076692	0.239986	0.058958	0.036725	0.066193	0.073025
0.617079	0.363172	0.375637	0.17374	0.771967	0.336077	0.329165	0.388531	0.751968
0.29623	0.125343	0.294301	0.127197	0.126722	0.073814	0.130068	0.246952	0.297939
0.175857	0.023223	0.13482	0.039598	0.475015	0.75869	0.113532	0.083682	0.066603
0.052486	0.03663	0.044385	0.029006	0.032049	0.219072	0.047488	0.039759	0.045371
0.408487	0.247077	0.351953	0.11724	0.646895	0.162394	0.171406	0.190671	0.734525
0.388954	0.028867	0.226205	0.046503	0.57538	0.914347	0.156604	0.397316	0.060998
0.021255	0.015576	0.018727	0.016875	0.015741	0.009425	0.017645	0.012856	0.015711
0.092233	0.108966	0.130105	0.1518	0.103751	0.232895	0.078354	0.11082	0.087022
0.212314	0.534729	0.07957	0.052051	0.453402	0.465011	0.467317	0.145489	0.266371
0.038359	0.043129	0.026987	0.031907	0.023651	0.135277	0.027906	0.030717	0.038177
0	0	0	0	0	0	0	0	0
0.075265	0.061427	0.057427	0.042576	0.034525	0.067233	0.066438	0.047285	0.06673
0.671381	0.23524	0.607567	0.329784	0.4004	0.154393	0.293635	0.488004	0.287019
0.058872	0.053583	0.078778	0.050287	0.264425	0.023665	0.030194	0.03186	0.024399
0.097353	0.048882	0.090638	0.056377	0.067916	0.054879	0.065835	0.067917	0.06988
0.620578	0.301958	0.513758	0.307424	0.734539	0.930362	0.442566	0.677782	0.321043
0.016398	0.013081	0.013527	0.014072	0.016296	0.010794	0.012776	0.014334	0.014124
0.207505	0.126817	0.297533	0.11605	0.093077	0.058215	0.125579	0.326877	0.230228
0.079906	0.071037	0.084062	0.075985	0.043361	0.02321	0.06211	0.055446	0.081811
0.656448	0.421599	0.581197	0.465168	0.763401	0.929261	0.531961	0.546608	0.433114
0.243399	0.080181	0.311861	0.108713	0.083744	0.042381	0.089552	0.098275	0.112895
0.103244	0.031788	0.093584	0.049371	0.04628	0.031811	0.049915	0.066622	0.050426

TCGA.A4.7	TCGA.G7.6	TCGA.HE.7	TCGA.B1.A	TCGA.A4.7	TCGA.UZ.A	TCGA.DW.7	TCGA.P4.A	TCGA.Q2.A
0.069841	0.045643	0.064429	0.031986	0.367626	0.221325	0.049499	0.041756	0.046288
0	0.410536	0.373552	0.15861	0	0	0	0.06019	0
0.88749	0.891869	0.954494	0.772212	0.882742	0.941869	0.949624	0.870386	0.921272
0.03233	0.025001	0.017958	0.0154	0.036122	0.021268	0.039721	0.019993	0.029788
0	0	0	0	0	0	0	0	0
0.409736	0.175877	0.06456	0.109458	0.449136	0.16223	0.043367	0.220526	0.333839
0.269923	0.035839	0.116875	0.133706	0.085349	0.036661	0.085657	0.353436	0.090593
0.013121	0.013555	0.016845	0.019137	0.015331	0.014023	0.015489	0.013875	0.016037
0	0	0	0.125957	0	0	0	0.145959	0
0.16455	0.138406	0.092813	0.051604	0.111617	0.104304	0.114316	0.119187	0.132889
0.130695	0.213212	0.097907	0.101114	0.245619	0.055653	0.28581	0.257081	0.21293
0.469201	0	0.266905	0.053166	0	0	0	0.056636	0
0.058716	0.038187	0.041909	0.035094	0.059872	0.042348	0.037006	0.051975	0.047109
0	0	0	0	0	0	0	0	0
0	0	0	0	0	0	0	0	0
0	0	0	0	0	0	0	0	0
0.094812	0.052106	0.039836	0.032133	0.051757	0.033755	0.032774	0.054788	0.03297
0	0	0.170471	0.060009	0	0	0	0.063339	0
0.042485	0.037479	0.022346	0.036409	0.038461	0.027164	0.040257	0.046883	0.031318
0	0	0	0.341317	0	0	0	0.24991	0
0.33982	0.074022	0.202438	0.194908	0.174035	0.093404	0.166583	0.367925	0.097535
0.136964	0.0322	0.037332	0.029036	0.396901	0.397511	0.340834	0.036561	0.287382
0.179849	0.113578	0.091156	0.121873	0.077263	0.143971	0.205251	0.106743	0.113534
0.095343	0.096363	0.100531	0.084159	0.102197	0.082454	0.09283	0.096422	0.114491
0.120597	0.188569	0.101424	0.11012	0.237115	0.046902	0.225676	0.298558	0.194757
0.432809	0.359678	0.671943	0.293327	0.279414	0.788465	0.188568	0.845985	0.738814
0.217144	0.146442	0.061071	0.117219	0.11292	0.141205	0.077585	0.210321	0.127601
0.035563	0.655955	0.01869	0.025715	0.599561	0.741094	0.639345	0.036747	0.620298
0.051804	0.050294	0.053949	0.040717	0.342671	0.108817	0.04734	0.032633	0.045368
0.197352	0.205236	0.24373	0.154869	0.200453	0.26443	0.085154	0.578666	0.478342
0.302165	0.685668	0.472089	0.033667	0.578767	0.824271	0.970359	0.037092	0.643903
0.016895	0.014785	0.013725	0.01738	0.017936	0.013967	0.015966	0.013964	0.018841
0.099102	0.118698	0.155755	0.10672	0.071301	0.081189	0.09422	0.091855	0.088059
0.696077	0.085022	0.668617	0.482932	0.141662	0.477926	0.389805	0.669252	0.090405
0.027452	0.03383	0.029791	0.026635	0.135688	0.069411	0.032359	0.032559	0.0275
0	0	0	0	0	0	0	0	0
0.063397	0.049352	0.04875	0.043451	0.059336	0.069981	0.064162	0.045555	0.053965
0.474054	0.236716	0.094907	0.193733	0.703607	0.247695	0.068541	0.252492	0.443791
0.037475	0.051759	0.028588	0.030055	0.050206	0.041549	0.028216	0.084982	0.056604
0.08494	0.05854	0.062108	0.06624	0.067882	0.079248	0.068745	0.082012	0.066559
0.417848	0.702491	0.386643	0.248701	0.699366	0.869438	0.889172	0.472508	0.750768
0.013273	0.011109	0.012651	0.013837	0.013461	0.011995	0.01041	0.015508	0.015947
0.227957	0.15499	0.064667	0.15042	0.14305	0.143904	0.076753	0.26064	0.081175
0.102152	0.06797	0.057161	0.058868	0.065922	0.061082	0.057631	0.061393	0.036444
0.482012	0.759796	0.216075	0.340051	0.745809	0.863474	0.215453	0.774214	0.811802
0.201285	0.098396	0.039864	0.073579	0.14623	0.068499	0.047718	0.07798	0.127168
0.048438	0.066772	0.05112	0.040401	0.058212	0.047379	0.035921	0.065666	0.053412

TCGA.KV.A	TCGA.Y8.A	TCGA.MH.A	TCGA.BQ.5	TCGA.Y8.A	TCGA.PJ.A	TCGA.B9.5	TCGA.B9.A	TCGA.BQ.7
0.096582	0.04647	0.04258	0.474968	0.273231	0.053103	0.290206	0.046791	0.049391
0.079411	0.102421	0.028795	0	0	0	0.237844	0.136959	0
0.683534	0.853631	0.934801	0.896766	0.877668	0.918502	0.955504	0.699455	0.948912
0.01524	0.015021	0.046743	0.044234	0.017279	0.030107	0.022853	0.018784	0.036663
0	0	0	0	0	0	0	0	0
0.223568	0.183236	0.269542	0.44049	0.376113	0.52515	0.086565	0.166932	0.156071
0.071607	0.150455	0.044256	0.076537	0.321971	0.171147	0.217319	0.372908	0.055948
0.012703	0.011238	0.019365	0.019016	0.012709	0.015144	0.013429	0.012338	0.015383
0.168626	0.263641	0.054003	0	0.488405	0	0	0.305423	0
0.1392	0.080581	0.193429	0.301473	0.06149	0.221623	0.104125	0.073996	0.123088
0.055687	0.117223	0.121396	0.12985	0.102957	0.090366	0.072811	0.063689	0.12653
0.073752	0.08417	0.037224	0	0	0	0.194209	0.119037	0
0.054536	0.050165	0.045074	0.056369	0.065401	0.05177	0.052935	0.042914	0.044469
0	0	0	0	0	0	0	0	0
0	0	0	0	0	0	0	0	0
0	0	0	0	0	0	0	0	0
0.099319	0.068018	0.029764	0.376715	0.087327	0.086381	0.047121	0.02503	0.038052
0.083703	0.042586	0.054151	0	0	0	0.080386	0.064248	0
0.032636	0.025838	0.034087	0.569512	0.053514	0.039165	0.035668	0.035718	0.026149
0.232207	0	0.083204	0	0	0	0	0.293136	0
0.113473	0.256515	0.092096	0.139675	0.355595	0.325539	0.259981	0.429669	0.104192
0.262109	0.241416	0.042188	0.482531	0.372091	0.067815	0.530155	0.097074	0.038125
0.135809	0.217895	0.185539	0.411432	0.261468	0.320753	0.069728	0.221673	0.116241
0.089266	0.084987	0.096757	0.10491	0.110502	0.098405	0.109336	0.090246	0.105963
0.050019	0.188433	0.114701	0.141276	0.090116	0.085233	0.07232	0.056188	0.125534
0.468075	0.468649	0.453771	0.332994	0.38839	0.679996	0.445171	0.787185	0.721173
0.165483	0.137331	0.129381	0.463406	0.221589	0.314818	0.082427	0.090813	0.114305
0.047897	0.012617	0.133216	0.535023	0.055448	0.203184	0.471661	0.016359	0.5659
0.04209	0.036438	0.040123	0.520868	0.201641	0.068669	0.219545	0.038678	0.035477
0.106698	0.162558	0.238302	0.190649	0.238115	0.607216	0.151197	0.525056	0.415381
0.079904	0.028064	0.640593	0.577951	0.277692	0.314194	0.846127	0.161824	0.802398
0.021859	0.010793	0.018488	0.542393	0.019055	0.016247	0.0122	0.011307	0.014504
0.084231	0.210057	0.084695	0.47291	0.068323	0.086425	0.100919	0.259105	0.139621
0.494859	0.693026	0.429845	0.126761	0.627292	0.365094	0.648263	0.875977	0.201931
0.032157	0.038253	0.031296	0.418459	0.155093	0.026814	0.206585	0.028879	0.025162
0	0	0	0	0	0	0	0	0
0.055408	0.053125	0.053528	0.530865	0.057736	0.060011	0.069816	0.042322	0.061502
0.283229	0.272515	0.325841	0.611403	0.412457	0.51175	0.130034	0.23458	0.216224
0.055267	0.024385	0.0488	0.043778	0.049169	0.081821	0.042459	0.035012	0.105755
0.068721	0.051365	0.069114	0.523957	0.070264	0.068702	0.068099	0.050873	0.06661
0.365796	0.248008	0.433442	0.672994	0.491239	0.637944	0.643945	0.244023	0.765967
0.014755	0.012561	0.017078	0.013963	0.012673	0.014305	0.013389	0.011667	0.011991
0.193218	0.168633	0.200359	0.491838	0.186764	0.27954	0.055705	0.088451	0.095964
0.05491	0.056929	0.044761	0.374445	0.0828	0.094318	0.052527	0.038871	0.058236
0.486599	0.463385	0.473067	0.732924	0.467305	0.675355	0.290925	0.393929	0.679073
0.138004	0.066499	0.092515	0.11964	0.140218	0.231084	0.045756	0.064293	0.049026
0.056858	0.042556	0.045117	0.054226	0.06471	0.112243	0.042919	0.045624	0.064677

TCGA.5P.A	TCGA.GL.A	TCGA.MH.A	TCGA.AL.7	TCGA.2Z.A	TCGA.DW.7	TCGA.GL.8	TCGA.5P.A	TCGA.2Z.A
0.033653	0.042703	0.054329	0.134063	0.039393	0.061377	0.063115	0.054467	0.280232
0.041323	0.15049	0	0	0.143241	0.244383	0.129724	0.045663	0.022184
0.905257	0.628348	0.927532	0.866236	0.939608	0.281189	0.935672	0.770486	0.911792
0.024322	0.033057	0.02061	0.028786	0.018379	0.024741	0.020157	0.021958	0.027099
0	0	0	0	0	0	0	0	0
0.267431	0.163357	0.416735	0.360586	0.083456	0.087099	0.130267	0.289762	0.554232
0.033297	0.207769	0.112479	0.040364	0.163794	0.262777	0.21957	0.087489	0.034047
0.016318	0.014936	0.016562	0.014197	0.015438	0.014996	0.017494	0.014847	0.0146
0.111125	0.21989	0	0	0.236733	0.361459	0.235342	0.110351	0.051894
0.113683	0.136693	0.234295	0.216543	0.102125	0.122549	0.126799	0.085745	0.212131
0.089862	0.072323	0.095056	0.105956	0.081501	0.084275	0.058551	0.117761	0.053001
0.036068	0.093073	0	0	0.068889	0.165261	0.074158	0.043399	0.027093
0.052506	0.030159	0.059156	0.052154	0.043743	0.043321	0.043969	0.059336	0.046641
0	0	0	0	0	0	0	0	0
0	0	0	0	0	0	0	0	0
0	0	0	0	0	0	0	0	0
0.059242	0.029543	0.103045	0.110639	0.036407	0.050164	0.043073	0.043079	0.062248
0.053675	0.07547	0	0	0.073405	0.103443	0.086657	0.04921	0.04184
0.047053	0.029202	0.046492	0.031174	0.029814	0.044229	0.026762	0.028085	0.029638
0.204637	0.396394	0	0	0.415233	0	0.415816	0.267778	0.092761
0.041694	0.275356	0.195967	0.118678	0.217515	0.280157	0.22788	0.147192	0.09599
0.027943	0.029363	0.043076	0.159022	0.060323	0.078355	0.291928	0.045622	0.470526
0.119666	0.112347	0.422662	0.273078	0.239773	0.11137	0.077162	0.128139	0.286341
0.096931	0.090537	0.083288	0.093672	0.089297	0.081221	0.090676	0.07143	0.091417
0.09734	0.072914	0.083901	0.112917	0.07715	0.071907	0.060622	0.1146	0.054542
0.25519	0.336232	0.796183	0.642574	0.755318	0.486416	0.659843	0.444824	0.608336
0.177629	0.099264	0.358646	0.269009	0.174132	0.098384	0.087039	0.170855	0.35346
0.024939	0.037335	0.27344	0.453369	0.435066	0.017153	0.085671	0.027239	0.47145
0.032604	0.040895	0.043205	0.12127	0.040691	0.060904	0.040558	0.041538	0.284298
0.179563	0.125514	0.713827	0.27677	0.572285	0.311052	0.359375	0.308857	0.407895
0.125708	0.207309	0.742018	0.53747	0.740592	0.019872	0.825907	0.035128	0.536102
0.019133	0.017683	0.015191	0.021595	0.011758	0.01609	0.012588	0.013419	0.017212
0.068049	0.079893	0.132905	0.082319	0.136162	0.116418	0.061241	0.122329	0.11071
0.057786	0.675343	0.519064	0.111708	0.644658	0.661764	0.48459	0.408695	0.125074
0.02385	0.023791	0.024455	0.070211	0.035449	0.029742	0.023452	0.035058	0.217711
0	0	0	0	0	0	0	0	0
0.061721	0.059014	0.054089	0.068848	0.050303	0.068301	0.064001	0.056338	0.049172
0.354803	0.21483	0.444424	0.402443	0.150452	0.129939	0.177627	0.351478	0.571747
0.033527	0.027616	0.088641	0.047092	0.054521	0.042285	0.027724	0.026731	0.033608
0.079379	0.072325	0.084049	0.072576	0.064303	0.059342	0.078809	0.070098	0.070166
0.358982	0.370281	0.737267	0.759452	0.754792	0.114578	0.35852	0.352239	0.744048
0.016033	0.013363	0.014085	0.011023	0.013834	0.013263	0.012847	0.014024	0.013785
0.211388	0.101006	0.468291	0.316828	0.222838	0.168421	0.07101	0.269185	0.298575
0.062883	0.030842	0.119352	0.123489	0.053993	0.086276	0.05994	0.05881	0.072304
0.490104	0.471206	0.563187	0.803749	0.651503	0.247109	0.412344	0.486916	0.941594
0.127439	0.042778	0.249095	0.250558	0.057192	0.048429	0.068201	0.148989	0.276829
0.065665	0.048167	0.046731	0.066054	0.048575	0.053442	0.050305	0.056296	0.095136

TCGA.A4.8	TCGA.BQ.7	TCGA.PJ.A	TCGA.SX.A	TCGA.O9.A	TCGA.B9.A	TCGA.2Z.A	TCGA.BQ.5	TCGA.Y8.A
0.049217	0.060599	0.376217	0.054967	0.05996	0.052029	0.051092	0.545731	0.048512
0.208235	0.051529	0	0.072022	0.171095	0.037109	0.08163	0	0.125712
0.94585	0.942463	0.954659	0.940097	0.429655	0.845083	0.923389	0.938656	0.75157
0.025132	0.025952	0.030509	0.01463	0.034688	0.020594	0.021299	0.024078	0.016686
0	0	0	0	0	0	0	0	0
0.152417	0.40294	0.145724	0.225157	0.197463	0.379049	0.156366	0.280727	0.214435
0.145388	0.050004	0.241998	0.040267	0.048467	0.05961	0.145561	0.043023	0.023942
0.014969	0.012404	0.014765	0.011469	0.014824	0.013585	0.012422	0.016783	0.013141
0.227993	0.080831	0	0.12001	0.273213	0.048503	0.23988	0	0.25253
0.088966	0.153772	0.14415	0.092703	0.166466	0.4123	0.090767	0.108806	0.073296
0.07673	0.077191	0.08136	0.062527	0.109826	0.12261	0.069677	0.129627	0.044325
0.107989	0.035501	0	0.054654	0.084511	0.057325	0.081686	0	0.133026
0.054394	0.046982	0.049831	0.040411	0.045282	0.062442	0.032687	0.062193	0.033086
0	0	0	0	0	0	0	0	0
0	0	0	0	0	0	0	0	0
0	0	0	0	0	0	0	0	0
0.036551	0.106649	0.046231	0.089808	0.047956	0.271356	0.029221	0.032157	0.033796
0.118934	0.053103	0	0.053889	0.065547	0.060875	0.081353	0	0.07835
0.042461	0.048661	0.028834	0.036181	0.045472	0.222937	0.032306	0.037557	0.029767
0.276804	0.214658	0	0.211323	0.40071	0.073048	0.389468	0	0.370792
0.224484	0.074166	0.295944	0.085835	0.082024	0.12814	0.239925	0.072575	0.050574
0.024564	0.028566	0.528531	0.037197	0.058119	0.040556	0.094177	0.606911	0.395679
0.100379	0.302662	0.217376	0.224348	0.225748	0.492563	0.090413	0.101417	0.152931
0.11397	0.096632	0.091352	0.081726	0.096635	0.079105	0.074438	0.119772	0.068172
0.064136	0.063797	0.078164	0.054575	0.089458	0.130716	0.06438	0.12273	0.051294
0.691279	0.695731	0.550973	0.372512	0.188808	0.741175	0.228562	0.839461	0.467414
0.065013	0.214296	0.133679	0.179383	0.133668	0.315004	0.11293	0.11513	0.178535
0.024185	0.471756	0.263885	0.026547	0.02658	0.123185	0.013157	0.465564	0.027866
0.034759	0.043216	0.303237	0.047704	0.043391	0.048922	0.037439	0.510432	0.034285
0.165082	0.357799	0.18345	0.206879	0.171205	0.31941	0.11636	0.700932	0.133354
0.109369	0.765648	0.778916	0.144368	0.024087	0.237771	0.040404	0.571932	0.098036
0.017382	0.043367	0.01475	0.015842	0.019685	0.441234	0.016563	0.015194	0.013917
0.070787	0.131217	0.07537	0.122998	0.104853	0.231312	0.103416	0.140525	0.069231
0.648045	0.320152	0.680681	0.16877	0.126988	0.123505	0.577994	0.072343	0.173021
0.022407	0.025567	0.283613	0.045479	0.047739	0.030084	0.031249	0.36837	0.026248
0	0	0	0	0	0	0	0	0
0.049073	0.081325	0.053023	0.053876	0.097364	0.342192	0.059331	0.052592	0.041569
0.174625	0.448698	0.22945	0.31614	0.280413	0.447523	0.217878	0.336403	0.292252
0.02914	0.038342	0.042091	0.039595	0.034336	0.053427	0.029822	0.045118	0.023052
0.073778	0.087928	0.076458	0.056971	0.073615	0.319015	0.078947	0.056464	0.062265
0.296954	0.858241	0.531003	0.362091	0.227438	0.577265	0.208887	0.819743	0.315006
0.015379	0.013273	0.013101	0.013245	0.015241	0.01287	0.013707	0.01411	0.014996
0.139884	0.196587	0.146575	0.183769	0.115916	0.375894	0.250494	0.086417	0.183383
0.051362	0.095751	0.054468	0.066211	0.05328	0.259455	0.031994	0.047087	0.040446
0.38459	0.676657	0.472936	0.506446	0.435716	0.687446	0.379418	0.8394	0.429119
0.032227	0.136726	0.084345	0.103545	0.093171	0.175492	0.078638	0.081819	0.105335
0.045324	0.077578	0.054029	0.05725	0.054999	0.057778	0.040613	0.05408	0.039194

TCGA.B1.A	TCGA.5P.A	TCGA.BQ.5	TCGA.B3.8	TCGA.UN.A	TCGA.2Z.A	TCGA.A4.7	TCGA.BQ.7	TCGA.DZ.6
0.051194	0.07658	0.225881	0.070324	0.049159	0.387843	0.062052	0.051515	0.166354
0	0.032782	0.060166	0	0	0	0	0.036079	0
0.932104	0.92487	0.930528	0.763114	0.941854	0.881861	0.612267	0.178171	0.938874
0.019644	0.02569	0.017687	0.032468	0.029899	0.020369	0.026448	0.030278	0.030593
0	0	0	0	0	0	0	0	0
0.578024	0.282663	0.311763	0.621026	0.136938	0.386974	0.333815	0.46501	0.078063
0.048229	0.236276	0.332845	0.143846	0.317368	0.157176	0.117517	0.284538	0.506292
0.013473	0.017224	0.015771	0.013122	0.012515	0.017169	0.015503	0.012913	0.012671
0	0.064437	0.093042	0	0	0	0	0.045451	0
0.173193	0.101258	0.152059	0.198049	0.097263	0.088149	0.132106	0.116926	0.098913
0.050358	0.125246	0.117399	0.124357	0.076212	0.145662	0.213281	0.151853	0.05466
0	0.022832	0.044412	0	0	0	0	0.031262	0
0.042161	0.038298	0.049529	0.049641	0.050831	0.087148	0.041319	0.071263	0.046715
0	0	0	0	0	0	0	0	0
0	0	0	0	0	0	0	0	0
0	0	0	0	0	0	0	0	0
0.035385	0.0412	0.074273	0.072709	0.028433	0.043249	0.046476	0.241779	0.045891
0	0.045161	0.044345	0	0.337748	0.503621	0	0.032275	0
0.031706	0.0305	0.031926	0.027835	0.022982	0.033961	0.033471	0.044816	0.035765
0	0.098103	0.216725	0	0	0	0	0.049422	0
0.08699	0.388653	0.42591	0.169312	0.598041	0.278511	0.183757	0.703318	0.778401
0.056002	0.101197	0.474492	0.275652	0.038735	0.709155	0.136326	0.027873	0.420139
0.310419	0.06885	0.173171	0.276588	0.134594	0.175769	0.079923	0.495678	0.07734
0.082939	0.100451	0.092645	0.099607	0.057309	0.08002	0.087212	0.094333	0.102819
0.04836	0.132565	0.127498	0.110284	0.060744	0.149734	0.215541	0.159778	0.057099
0.488003	0.854215	0.857097	0.554944	0.7841	0.712571	0.189515	0.475986	0.682214
0.279568	0.093799	0.216179	0.344051	0.114849	0.161847	0.152215	0.350476	0.10928
0.30962	0.682345	0.425695	0.033777	0.070758	0.141951	0.029001	0.013043	0.074354
0.040065	0.047395	0.169438	0.047978	0.03405	0.328048	0.045846	0.074291	0.138325
0.270087	0.565927	0.763625	0.278438	0.503587	0.638119	0.265263	0.771074	0.52573
0.681416	0.734886	0.728599	0.095976	0.09104	0.303328	0.021359	0.015016	0.850911
0.015618	0.014595	0.017154	0.016222	0.015009	0.019163	0.016636	0.013193	0.020799
0.092323	0.091162	0.184474	0.073321	0.12021	0.128843	0.094239	0.11775	0.093851
0.139629	0.671646	0.684619	0.365289	0.89574	0.527514	0.182144	0.764847	0.742727
0.030972	0.038821	0.116443	0.029953	0.026888	0.275842	0.03543	0.024364	0.079603
0	0	0	0	0	0	0	0	0
0.049082	0.055652	0.072567	0.06792	0.061122	0.063072	0.070991	0.073284	0.06547
0.6079	0.32379	0.379767	0.622783	0.208305	0.610884	0.388269	0.765996	0.138873
0.03912	0.370995	0.349153	0.039736	0.030953	0.224864	0.06892	0.079398	0.054613
0.067344	0.068457	0.058673	0.075473	0.070516	0.069715	0.085571	0.075218	0.07014
0.748352	0.85745	0.891624	0.462448	0.451033	0.651531	0.397407	0.14491	0.109654
0.013146	0.012338	0.012082	0.012796	0.013086	0.015799	0.011587	0.010737	0.010774
0.279555	0.205964	0.187907	0.335611	0.07816	0.250697	0.14542	0.122269	0.078993
0.074855	0.057169	0.092238	0.071506	0.029016	0.051143	0.070263	0.267522	0.045381
0.617864	0.856858	0.958441	0.605925	0.743434	0.701932	0.460967	0.281163	0.310046
0.237234	0.126174	0.166738	0.326977	0.051095	0.176533	0.125837	0.686707	0.041202
0.070319	0.036616	0.064114	0.105941	0.033069	0.070527	0.068729	0.06163	0.061299

TCGA.A4.7	TCGA.WN.A	TCGA.2Z.A	TCGA.SX.A	TCGA.2Z.A	TCGA.Y8.A	TCGA.UZ.A	TCGA.BQ.7	TCGA.UZ.A
0.057025	0.061081	0.05595	0.07916	0.050732	0.060451	0.044481	0.056956	0.059467
0.056504	0	0	0.346586	0.088152	0	0.108082	0.02637	0
0.488618	0.833623	0.740518	0.730506	0.494369	0.714082	0.214369	0.925616	0.489507
0.022502	0.026743	0.023799	0.027717	0.02849	0.020414	0.020878	0.024627	0.018845
0	0	0	0	0	0	0	0	0
0.226155	0.410842	0.265443	0.090853	0.195693	0.032034	0.209147	0.561874	0.050262
0.034163	0.052326	0.078834	0.045923	0.023982	0.686902	0.029108	0.064176	0.021497
0.017193	0.015623	0.016695	0.015827	0.012512	0.014223	0.014071	0.014469	0.012138
0.114132	0	0	0	0.135634	0	0.190464	0.037892	0
0.105576	0.090525	0.12639	0.1457	0.113169	0.068081	0.078442	0.208286	0.101015
0.068962	0.119635	0.240894	0.271787	0.060091	0.085816	0.287779	0.149582	0.048776
0.068073	0	0	0.170964	0.059636	0.338392	0.084497	0.025326	0.214826
0.048125	0.070175	0.066174	0.059327	0.054562	0.045647	0.038272	0.048682	0.036268
0	0	0	0	0	0	0	0	0
0	0	0	0	0	0	0	0	0
0	0	0	0	0	0	0	0	0
0.035776	0.039448	0.044361	0.032592	0.138366	0.031438	0.038022	0.098444	0.043146
0.068488	0	0	0.093619	0.053569	0	0.067958	0.033429	0.168824
0.042542	0.044333	0.031251	0.04616	0.035062	0.039844	0.031397	0.035425	0.025298
0.272875	0	0	0	0.311976	0	0.38156	0.054637	0
0.06032	0.109438	0.184842	0.080174	0.029061	0.630438	0.033926	0.106108	0.031434
0.041029	0.036656	0.109669	0.130359	0.061375	0.123079	0.051842	0.030626	0.283047
0.097131	0.206766	0.084545	0.249372	0.272436	0.253551	0.078253	0.253584	0.0844
0.080685	0.071735	0.086837	0.106892	0.096315	0.073965	0.066422	0.103074	0.088218
0.064547	0.110344	0.220184	0.237203	0.055877	0.077827	0.334346	0.107416	0.045963
0.248985	0.718199	0.661826	0.116304	0.115577	0.529721	0.154532	0.253345	0.073979
0.123677	0.25413	0.121592	0.084459	0.155817	0.142741	0.090279	0.329062	0.066845
0.082623	0.042125	0.034308	0.021343	0.052841	0.01852	0.026966	0.061942	0.013381
0.042893	0.036879	0.037875	0.046034	0.041494	0.03535	0.028528	0.046742	0.039939
0.123234	0.620665	0.590854	0.084203	0.157281	0.448055	0.106892	0.248793	0.044024
0.089854	0.076104	0.038848	0.056324	0.076575	0.020127	0.026461	0.303114	0.061938
0.017158	0.014718	0.012473	0.020125	0.011452	0.017327	0.015159	0.01463	0.01264
0.097408	0.104324	0.078333	0.133867	0.080817	0.076396	0.078693	0.106809	0.105465
0.052679	0.428398	0.404666	0.267644	0.135088	0.897141	0.180834	0.136191	0.080069
0.024657	0.029188	0.026755	0.03207	0.038645	0.031579	0.019886	0.027486	0.026511
0	0	0	0	0	0	0	0	0
0.053845	0.077884	0.049744	0.058511	0.05769	0.061706	0.052939	0.062617	0.06784
0.242932	0.497251	0.419161	0.164121	0.267838	0.041763	0.255481	0.617078	0.138131
0.029454	0.051188	0.027482	0.027461	0.036299	0.028053	0.024931	0.050494	0.023515
0.077258	0.090852	0.069408	0.063	0.072317	0.070678	0.070262	0.055685	0.072829
0.370933	0.564559	0.308212	0.169627	0.315397	0.294101	0.266935	0.526525	0.089739
0.014994	0.013806	0.015128	0.015622	0.010905	0.014749	0.015588	0.010696	0.010811
0.107854	0.130298	0.10205	0.094915	0.134753	0.15521	0.115556	0.324593	0.125195
0.053078	0.044227	0.049818	0.040536	0.051623	0.039176	0.045599	0.099738	0.071803
0.488892	0.567628	0.464738	0.260574	0.494816	0.093553	0.396986	0.600614	0.260075
0.068658	0.115908	0.116556	0.050317	0.094209	0.047846	0.07109	0.271645	0.05238
0.062568	0.072414	0.055896	0.036499	0.048379	0.026846	0.039238	0.089683	0.034847

TCGA.P4.A	TCGA.BQ.5	TCGA.MH.A	TCGA.GL.A	TCGA.GL.A	TCGA.SX.A	TCGA.F9.A	TCGA.DZ.6	TCGA.GL.7
0.208311	0.100671	0.040893	0.040581	0.070489	0.041753	0.073579	0.04336	0.60064
0	0.039536	0.095466	0.044873	0.041334	0.191822	0.028375	0.039535	0
0.846182	0.943699	0.519639	0.899223	0.87597	0.916087	0.91431	0.936289	0.834566
0.033539	0.03809	0.034548	0.012972	0.026427	0.014848	0.02009	0.019693	0.049137
0	0	0	0	0	0	0	0	0
0.758974	0.323913	0.125744	0.554999	0.544899	0.067443	0.471057	0.513207	0.78453
0.060383	0.157766	0.057307	0.032678	0.111117	0.015625	0.064641	0.040141	0.04781
0.014154	0.013968	0.017541	0.010213	0.014096	0.013901	0.012948	0.010818	0.014285
0	0.096799	0.21852	0.119106	0.078058	0.331746	0.064309	0.067542	0
0.446859	0.081661	0.148006	0.082176	0.143139	0.057791	0.130618	0.146883	0.426536
0.168117	0.077525	0.073829	0.054729	0.17139	0.065107	0.110882	0.098066	0.168527
0	0.034888	0.086259	0.036863	0.049027	0.130207	0.029487	0.025699	0
0.078963	0.062566	0.037258	0.036136	0.08997	0.028234	0.070951	0.045257	0.045411
0	0	0	0	0	0	0	0	0
0	0	0	0	0	0	0	0	0
0	0	0	0	0	0	0	0	0
0.452007	0.06215	0.036006	0.060824	0.088502	0.037543	0.064194	0.084447	0.529555
0	0.027298	0.107372	0.032431	0.046854	0.105029	0.034886	0.040291	0
0.405215	0.028837	0.038217	0.029732	0.056657	0.033668	0.244782	0.04428	0.507623
0	0.191908	0.389899	0.148398	0.112542	0.356156	0.112364	0.086928	0
0.126401	0.104906	0.106752	0.034584	0.175889	0.044471	0.125148	0.079954	0.108696
0.039464	0.305332	0.062262	0.038487	0.067008	0.435625	0.039498	0.099773	0.493404
0.563569	0.124667	0.155755	0.261896	0.249414	0.080056	0.221712	0.151242	0.605861
0.111047	0.117417	0.078599	0.087221	0.093913	0.071326	0.07652	0.10988	0.082861
0.167777	0.072754	0.080064	0.055485	0.12706	0.063267	0.121299	0.08681	0.160109
0.403368	0.812778	0.540997	0.493314	0.648946	0.199792	0.6524	0.528703	0.273818
0.513361	0.145987	0.11332	0.215775	0.266646	0.075214	0.251255	0.276489	0.617355
0.50331	0.505165	0.054612	0.039154	0.407008	0.018838	0.579352	0.164459	0.523256
0.169384	0.045236	0.036152	0.039804	0.073618	0.03364	0.043753	0.0473	0.595236
0.356308	0.393464	0.335767	0.31533	0.554241	0.104407	0.496252	0.361444	0.124758
0.560422	0.788072	0.03365	0.083923	0.446857	0.030861	0.581486	0.305182	0.530078
0.488654	0.015304	0.024865	0.010321	0.081318	0.022144	0.391088	0.022397	0.5355
0.446731	0.155265	0.065277	0.279639	0.144314	0.071712	0.142188	0.116646	0.652073
0.175692	0.42834	0.592835	0.092785	0.357574	0.044292	0.215966	0.149289	0.092546
0.057673	0.029146	0.026403	0.025383	0.029777	0.024499	0.03341	0.024052	0.436625
0	0	0	0	0	0	0	0	0
0.404271	0.070807	0.054641	0.05038	0.093278	0.055707	0.297769	0.068576	0.509421
0.831391	0.446007	0.202842	0.576666	0.563134	0.150542	0.554367	0.542294	0.846677
0.059843	0.037846	0.030174	0.110461	0.071029	0.02678	0.044046	0.055232	0.060293
0.385887	0.065562	0.071591	0.048922	0.093843	0.061922	0.273411	0.075515	0.477964
0.72416	0.777248	0.397721	0.53372	0.658128	0.224081	0.718805	0.633261	0.617433
0.014527	0.014421	0.015392	0.00976	0.015739	0.01291	0.01063	0.012119	0.014377
0.499434	0.136419	0.145012	0.190906	0.24097	0.084894	0.211942	0.190373	0.577297
0.433289	0.066007	0.052865	0.06119	0.100883	0.035247	0.075129	0.080169	0.542335
0.733588	0.659556	0.467914	0.636424	0.75714	0.291126	0.725022	0.631922	0.649117
0.428521	0.134138	0.090551	0.177673	0.293872	0.030187	0.174726	0.227421	0.59159
0.084033	0.060016	0.039206	0.048599	0.061705	0.033013	0.057525	0.081318	0.052699

TCGA.G7.A	TCGA.Y8.A	TCGA.BQ.7	TCGA.HE.A	TCGA.B9.7	TCGA.MH.A	TCGA.B9.A	TCGA.P4.A	TCGA.A4.8
0.048693	0.058759	0.189403	0.086547	0.052437	0.066008	0.047098	0.108435	0.050289
0.062246	0.050817	0.090025	0.270567	0.255352	0.037998	0.042423	0.130648	0.22407
0.784589	0.92368	0.925305	0.948224	0.92851	0.844079	0.912652	0.941828	0.853115
0.018027	0.019311	0.021983	0.019821	0.029309	0.021532	0.024237	0.029412	0.041672
0	0	0	0	0	0	0	0	0
0.127512	0.457978	0.208518	0.212133	0.058274	0.248454	0.486557	0.264457	0.126063
0.052952	0.053742	0.391227	0.047218	0.385045	0.02836	0.030272	0.14924	0.030813
0.014686	0.01389	0.013031	0.012301	0.011269	0.014872	0.013491	0.016414	0.014062
0.15986	0.140178	0.203449	0.27317	0.382129	0.043979	0.090765	0.213909	0.327337
0.079938	0.105649	0.135589	0.123317	0.113323	0.158156	0.1531	0.13136	0.124389
0.199419	0.080926	0.286702	0.047281	0.069156	0.061712	0.093613	0.081637	0.062278
0.0652	0.044391	0.069927	0.139901	0.32548	0.031117	0.030388	0.061882	0.114953
0.036137	0.064605	0.045819	0.050495	0.050045	0.036622	0.053143	0.037506	0.044541
0	0	0	0	0	0	0	0	0
0	0	0	0	0	0	0	0	0
0	0	0	0	0	0	0	0	0
0.038377	0.08384	0.072571	0.026202	0.065156	0.05488	0.065857	0.050974	0.030993
0.069787	0.060177	0.059448	0.098758	0.17875	0.043745	0.067317	0.073717	0.086634
0.025251	0.04836	0.036449	0.022736	0.032658	0.035911	0.036071	0.038594	0.031922
0.361938	0.104628	0.295128	0.412384	0.427081	0.057339	0.193413	0.404008	0.44939
0.140706	0.092173	0.467965	0.081504	0.444735	0.054791	0.060745	0.120745	0.055746
0.033595	0.103674	0.243473	0.126485	0.033887	0.051706	0.043162	0.327464	0.404236
0.057447	0.284898	0.093049	0.130916	0.155455	0.434422	0.219504	0.207068	0.093725
0.094568	0.107302	0.106578	0.088419	0.083362	0.082628	0.09681	0.098897	0.104699
0.248087	0.087371	0.281687	0.055067	0.077654	0.053482	0.07272	0.090401	0.062858
0.387113	0.339839	0.869186	0.503642	0.304198	0.092124	0.642573	0.793701	0.335542
0.124592	0.25013	0.132808	0.122571	0.127136	0.268953	0.182575	0.126878	0.097354
0.018084	0.113527	0.427502	0.347181	0.021721	0.024909	0.136888	0.294081	0.02939
0.037413	0.044151	0.137462	0.039758	0.04633	0.038832	0.048113	0.070038	0.035543
0.145451	0.298827	0.606137	0.141962	0.25298	0.114192	0.440104	0.500557	0.12981
0.026779	0.490207	0.746349	0.802077	0.072845	0.031802	0.242639	0.573873	0.047701
0.018342	0.017629	0.016992	0.014975	0.016829	0.016686	0.016961	0.020336	0.014552
0.075579	0.107567	0.099885	0.061982	0.097028	0.143664	0.07542	0.06862	0.078931
0.382845	0.273237	0.846528	0.34598	0.883633	0.040953	0.168285	0.629688	0.19768
0.030517	0.038209	0.127826	0.027466	0.02619	0.032782	0.032757	0.031583	0.023284
0	0	0	0	0	0	0	0	0
0.049975	0.066554	0.060966	0.056844	0.053615	0.045267	0.059622	0.058921	0.054069
0.19172	0.527186	0.257042	0.268563	0.071303	0.319687	0.526544	0.30202	0.192946
0.032297	0.036078	0.204774	0.027572	0.026998	0.02501	0.033566	0.032367	0.026874
0.052212	0.063808	0.069952	0.066891	0.073889	0.056484	0.063349	0.082695	0.079783
0.282199	0.508782	0.714671	0.718877	0.210257	0.32363	0.613982	0.716241	0.27024
0.013805	0.013512	0.011636	0.015039	0.011897	0.01561	0.012115	0.014391	0.013045
0.173768	0.193251	0.142413	0.13668	0.155133	0.249148	0.178634	0.143214	0.097922
0.037738	0.085389	0.071551	0.036738	0.051117	0.07936	0.051382	0.058037	0.042254
0.448559	0.56082	0.626412	0.513441	0.208203	0.481187	0.583107	0.555681	0.40985
0.04695	0.147406	0.074064	0.079882	0.04096	0.166786	0.240758	0.150136	0.073029
0.030727	0.06269	0.060511	0.051289	0.041262	0.056602	0.078917	0.051366	0.04903

TCGA.P4.A	TCGA.G7.7	TCGA.2Z.A	TCGA.5P.A	TCGA.AT.A	TCGA.2Z.A	TCGA.5P.A	TCGA.2Z.A	TCGA.EV.5
0.442288	0.063172	0.060559	0.07302	0.481253	0.153704	0.054047	0.040355	0.173617
0	0	0.059137	0.117817	0.225988	0.797396	0	0.092366	0.287161
0.917913	0.931324	0.726484	0.89837	0.942678	0.930734	0.875427	0.901724	0.96596
0.033282	0.041181	0.021081	0.028239	0.016713	0.038439	0.128641	0.018877	0.019337
0	0	0	0	0	0	0	0	0
0.339825	0.155626	0.343179	0.161595	0.163231	0.224335	0.151567	0.263367	0.061319
0.079792	0.037218	0.055379	0.032861	0.147913	0.036814	0.031904	0.059386	0.299443
0.015646	0.019232	0.01403	0.015454	0.014728	0.012769	0.01493	0.016767	0.016006
0	0	0.129942	0.259643	0.347397	0.801021	0	0.145794	0
0.175049	0.091758	0.110419	0.115863	0.098351	0.103412	0.126431	0.136733	0.085975
0.121599	0.071598	0.068649	0.052315	0.095008	0.053174	0.182455	0.074654	0.071393
0.407259	0	0.050874	0.120509	0.19501	0.736879	0	0.064756	0.27859
0.048772	0.04457	0.04901	0.034853	0.04138	0.045892	0.046941	0.047542	0.060071
0	0	0	0	0	0	0	0	0
0	0	0	0	0	0	0	0	0
0	0	0	0	0	0	0	0	0
0.038905	0.043986	0.061616	0.044432	0.060844	0.038664	0.048046	0.043286	0.032015
0	0	0.06904	0.139062	0.106981	0.644313	0.520881	0.054337	0.146056
0.041793	0.027968	0.031309	0.02809	0.030979	0.028217	0.033663	0.030765	0.02931
0	0	0.247188	0.390483	0.447575	0.856945	0	0.361435	0
0.130536	0.129592	0.095859	0.057628	0.158752	0.080345	0.112444	0.131241	0.440532
0.576108	0.071258	0.327356	0.586557	0.713184	0.042199	0.031399	0.309772	0.663401
0.186937	0.116618	0.09984	0.146811	0.276535	0.078004	0.086583	0.15846	0.14308
0.12007	0.103367	0.086792	0.084015	0.108625	0.074315	0.079504	0.084221	0.117953
0.110968	0.079093	0.080033	0.054022	0.094003	0.057792	0.183476	0.087037	0.067259
0.33684	0.269127	0.363755	0.324466	0.363021	0.830247	0.561329	0.749691	0.837725
0.16782	0.094917	0.173756	0.127841	0.119967	0.116471	0.109443	0.251159	0.09513
0.105198	0.714299	0.025043	0.065897	0.315752	0.79491	0.616779	0.184961	0.78269
0.393368	0.052668	0.036741	0.043219	0.372812	0.043821	0.040945	0.033578	0.074635
0.266328	0.180288	0.212918	0.096398	0.166725	0.288804	0.4932	0.29146	0.703244
0.210128	0.773032	0.035949	0.17195	0.678313	0.893633	0.641187	0.351901	0.971328
0.018737	0.020546	0.017075	0.022489	0.015679	0.016055	0.015839	0.014002	0.011147
0.071048	0.177034	0.083671	0.089245	0.083955	0.086314	0.075475	0.144325	0.240655
0.133775	0.108752	0.425727	0.282023	0.564916	0.240145	0.066508	0.480806	0.833202
0.328098	0.033106	0.029776	0.03886	0.341129	0.030663	0.023767	0.030805	0.060279
0	0	0	0	0	0	0	0	0
0.084596	0.053669	0.058373	0.053293	0.045257	0.065203	0.060768	0.056246	0.035661
0.42435	0.233263	0.37777	0.232549	0.211625	0.244001	0.209219	0.298954	0.100096
0.058313	0.041921	0.031321	0.029065	0.023846	0.03296	0.041881	0.033769	0.055423
0.086116	0.078588	0.08374	0.068031	0.069881	0.094327	0.072626	0.071211	0.052941
0.536414	0.852725	0.31891	0.322857	0.601261	0.901434	0.684493	0.663622	0.976862
0.013892	0.018397	0.013811	0.015025	0.015158	0.013685	0.014427	0.014502	0.013425
0.160581	0.164368	0.138354	0.141792	0.162313	0.093146	0.110137	0.336764	0.116135
0.0466	0.057326	0.061056	0.072498	0.075436	0.037607	0.053077	0.073821	0.054052
0.532131	0.814069	0.478132	0.415446	0.462605	0.926517	0.681234	0.611158	0.680463
0.162183	0.077556	0.189858	0.056355	0.096188	0.09613	0.062224	0.069879	0.028158
0.071368	0.045067	0.059507	0.042364	0.042833	0.058057	0.048841	0.038526	0.040834

TCGA.UZ.A	TCGA.A4.7	TCGA.SX.A	TCGA.2Z.A	TCGA.G7.6	TCGA.A4.7	TCGA.B1.A	TCGA.WN.A	TCGA.BQ.5
0.048078	0.045034	0.158952	0.070574	0.447542	0.041719	0.069614	0.043637	0.033312
0.119054	0.174978	0.108473	0.109589	0	0.056297	0.050544	0.029935	0.031966
0.44397	0.944875	0.412716	0.696742	0.839233	0.93043	0.902465	0.899563	0.907161
0.021801	0.023679	0.015401	0.020391	0.027471	0.016501	0.023363	0.022868	0.026397
0	0	0	0	0	0	0	0	0
0.168136	0.14457	0.091203	0.170014	0.325151	0.296872	0.362974	0.837835	0.485248
0.033239	0.18065	0.491635	0.026501	0.1404	0.160955	0.028393	0.099624	0.068145
0.014763	0.014057	0.013623	0.010911	0.01462	0.017823	0.014975	0.013751	0.011311
0.272035	0.160565	0.270451	0	0	0.070336	0.1136	0.053508	0.043281
0.120503	0.159471	0.048228	0.096987	0.119293	0.126505	0.159659	0.077962	0.201096
0.076709	0.070114	0.096576	0.041498	0.484768	0.07132	0.058955	0.084901	0.12975
0.189669	0.064935	0.086047	0.115518	0	0.035467	0.051089	0.021942	0.027561
0.033726	0.039308	0.049475	0.03198	0.05454	0.049783	0.061555	0.163912	0.076602
0	0	0	0	0	0	0	0	0
0	0	0	0	0	0	0	0	0
0	0	0	0	0	0	0	0	0
0.040769	0.064417	0.026624	0.031185	0.043337	0.053296	0.088935	0.034186	0.128302
0.062645	0.050666	0.073222	0.074633	0	0.045033	0.055844	0.021073	0.039761
0.029266	0.031965	0.030865	0.024253	0.024906	0.037314	0.032149	0.031302	0.041675
0.364752	0.370854	0.388885	0	0	0.181566	0.257499	0.103962	0.054307
0.084754	0.232861	0.52481	0.031102	0.220763	0.215353	0.080647	0.3478	0.150243
0.249992	0.031521	0.23622	0.042994	0.382136	0.040053	0.225323	0.031193	0.03128
0.114674	0.092315	0.118574	0.134007	0.101359	0.224923	0.132651	0.157528	0.232042
0.088086	0.095881	0.075133	0.103365	0.11696	0.115397	0.078966	0.07648	0.110919
0.081679	0.070598	0.092372	0.055424	0.487905	0.085155	0.062321	0.108075	0.13953
0.327148	0.748783	0.695805	0.124321	0.67155	0.760056	0.604121	0.848881	0.618095
0.117183	0.098809	0.099287	0.112556	0.137464	0.209097	0.19171	0.17506	0.32446
0.015588	0.095773	0.015327	0.018057	0.575636	0.347466	0.025628	0.133534	0.496262
0.03538	0.043029	0.10364	0.041733	0.445077	0.053054	0.038682	0.049144	0.042584
0.150284	0.366253	0.396419	0.090848	0.414161	0.77909	0.223454	0.545177	0.533079
0.026715	0.743658	0.022331	0.030689	0.611644	0.547142	0.277375	0.224238	0.520049
0.014418	0.013856	0.043507	0.014373	0.015296	0.020617	0.016798	0.012339	0.016532
0.074887	0.113464	0.082716	0.127885	0.150251	0.103036	0.089517	0.257504	0.13941
0.17048	0.595504	0.82923	0.056694	0.262054	0.520472	0.237355	0.457891	0.221785
0.042987	0.025664	0.067099	0.02598	0.416104	0.039918	0.025678	0.032589	0.025439
0	0	0	0	0	0	0	0	0
0.052844	0.051043	0.069152	0.054913	0.06033	0.074734	0.055568	0.046685	0.068739
0.216679	0.191378	0.156249	0.262481	0.388887	0.372337	0.426561	0.844564	0.554472
0.027194	0.051429	0.040552	0.021842	0.088708	0.150038	0.048833	0.136978	0.083853
0.063903	0.067187	0.065807	0.070691	0.062123	0.086376	0.068682	0.06394	0.077361
0.226163	0.486518	0.147998	0.187993	0.61821	0.763368	0.438212	0.662792	0.757561
0.012508	0.011814	0.012863	0.011098	0.010572	0.012725	0.014057	0.012033	0.014051
0.099327	0.265467	0.127802	0.125999	0.10605	0.147668	0.207466	0.162797	0.293713
0.050103	0.066033	0.030673	0.03103	0.059591	0.060501	0.085456	0.04376	0.127572
0.414284	0.342143	0.325744	0.383841	0.678669	0.695721	0.550936	0.608004	0.804326
0.096339	0.094519	0.040627	0.071359	0.104165	0.109825	0.21164	0.510597	0.29168
0.05428	0.049559	0.043122	0.035713	0.074661	0.075436	0.074456	0.108218	0.081515

TCGA.5P.A	TCGA.B1.A	TCGA.BQ.5	TCGA.BQ.5	TCGA.EV.5	TCGA.BQ.5	TCGA.5P.A	TCGA.5P.A	TCGA.DW.7
0.042778	0.0527	0.062687	0.045115	0.041888	0.038062	0.166147	0.040072	0.034278
0.096009	0.385175	0.034562	0.050466	0	0.036311	0.134437	0.030145	0.053193
0.931767	0.611879	0.882496	0.908135	0.796538	0.784442	0.932452	0.553838	0.94013
0.022928	0.020499	0.024298	0.022157	0.130797	0.021915	0.025553	0.021739	0.030415
0	0	0	0	0	0	0	0	0
0.148248	0.109585	0.415321	0.25874	0.345024	0.402297	0.167186	0.393358	0.360342
0.117543	0.371824	0.098039	0.092225	0.195475	0.259786	0.045027	0.172824	0.046799
0.016979	0.017223	0.01055	0.023789	0.017103	0.044482	0.015812	0.014003	0.016436
0.238657	0.395167	0.049824	0.072278	0	0.069956	0.229323	0.045025	0.075627
0.204273	0.138889	0.155214	0.099716	0.11745	0.178172	0.090789	0.090495	0.180391
0.231545	0.153371	0.173274	0.215318	0.089581	0.141534	0.089207	0.275263	0.077765
0.055221	0	0.028927	0.03203	0	0.033774	0.080149	0.038209	0.039871
0.056768	0.040626	0.045614	0.041842	0.05434	0.106147	0.041627	0.077539	0.049435
0	0	0	0	0	0	0	0	0
0	0	0	0	0	0	0	0	0
0	0	0	0	0	0	0	0	0
0.074067	0.037003	0.078994	0.042294	0.05246	0.070726	0.028969	0.048375	0.045975
0.052812	0	0.026665	0.050376	0	0.022577	0.079283	0.035604	0.051338
0.041597	0.027444	0.046175	0.028398	0.050448	0.043074	0.030628	0.03042	0.036978
0.43483	0.469136	0.067274	0.201203	0.251168	0.121644	0.451049	0.092256	0.232515
0.165622	0.448961	0.167536	0.147448	0.822333	0.254113	0.10632	0.375551	0.069118
0.040044	0.158874	0.040482	0.02344	0.03068	0.024567	0.215489	0.044884	0.023528
0.29008	0.095092	0.143633	0.119074	0.117222	0.139405	0.114487	0.199221	0.258525
0.083245	0.084428	0.091066	0.088828	0.120512	0.104013	0.073016	0.087659	0.110362
0.225253	0.16928	0.149419	0.235964	0.148969	0.192623	0.082629	0.292178	0.082573
0.44539	0.584571	0.703997	0.717223	0.259395	0.713052	0.509903	0.619894	0.475608
0.393218	0.114161	0.188903	0.204042	0.223397	0.233437	0.081618	0.222888	0.256639
0.097907	0.019636	0.553549	0.357986	0.028903	0.049331	0.175083	0.055818	0.046138
0.043498	0.044673	0.061051	0.042831	0.03451	0.045019	0.079254	0.037816	0.043822
0.253505	0.283453	0.452614	0.349504	0.260644	0.517337	0.140411	0.452219	0.290388
0.250216	0.070419	0.490533	0.565024	0.048501	0.077817	0.41606	0.073479	0.126988
0.030706	0.018855	0.012537	0.013484	0.011815	0.011218	0.013254	0.040366	0.013609
0.181655	0.080804	0.125312	0.118213	0.137973	0.21731	0.082228	0.090035	0.066479
0.671847	0.806284	0.17617	0.261717	0.157106	0.519386	0.363305	0.533198	0.10308
0.026247	0.029077	0.034438	0.027435	0.027314	0.030779	0.051285	0.027817	0.019109
0	0	0	0	0	0	0	0	0
0.053483	0.040704	0.05175	0.049631	0.051004	0.075995	0.066235	0.057636	0.051333
0.209771	0.147808	0.460831	0.315963	0.292034	0.452784	0.248751	0.459166	0.420939
0.024105	0.039825	0.076452	0.046472	0.0647	0.120394	0.026186	0.047251	0.044041
0.079641	0.072476	0.068354	0.070901	0.101489	0.085048	0.076944	0.072875	0.071728
0.58363	0.24198	0.687495	0.732784	0.451298	0.414339	0.310282	0.306008	0.479377
0.013341	0.0152	0.010528	0.013852	0.015069	0.012057	0.014163	0.01819	0.014319
0.432607	0.263207	0.156248	0.140027	0.194856	0.351227	0.119225	0.100573	0.210268
0.084856	0.034232	0.07546	0.051558	0.08575	0.088884	0.049634	0.054945	0.061318
0.478935	0.361606	0.736155	0.803123	0.494378	0.474927	0.428173	0.483815	0.569793
0.055069	0.072856	0.16699	0.097367	0.133787	0.233418	0.089393	0.079806	0.215349
0.048356	0.047601	0.087027	0.063909	0.058875	0.079783	0.05895	0.061933	0.074897

TCGA.G7.7	TCGA.BQ.5	TCGA.BQ.7	TCGA.5P.A	TCGA.UZ.A	TCGA.HE.A	TCGA.5P.A9K4.01A.11D.A42K.C
0.053486	0.782868	0.052901	0.049124	0.133825	0.051826	0.093016
0.306076	0.069888	0.065106	0.046328	0.074948	0.17413	0.089623
0.926054	0.505949	0.946671	0.912051	0.902051	0.9134	0.939124
0.024002	0.020264	0.017688	0.030007	0.013958	0.054894	0.020518
0	0	0	0	0	0	0
0.347517	0.231699	0.392523	0.350141	0.248639	0.107656	0.147304
0.16185	0.595296	0.031956	0.034411	0.06985	0.219159	0.028985
0.014736	0.015322	0.01303	0.01672	0.010984	0.016048	0.010537
0.313735	0.138727	0.09692	0.189575	0.251863	0.385571	0.200706
0.146349	0.251991	0.131226	0.151927	0.051675	0.19094	0.074233
0.073586	0.290894	0.0698	0.106281	0.059813	0.179149	0.057006
0.340823	0.066919	0.044579	0.043164	0.056999	0.102424	0.068461
0.050151	0.051415	0.048484	0.043377	0.040337	0.050248	0.04372
0	0	0	0	0	0	0
0	0	0	0	0	0	0
0	0	0	0	0	0	0
0.088192	0.520777	0.054612	0.041872	0.044994	0.0333	0.039913
0.458091	0.098845	0.046913	0.043949	0.036822	0.105895	0.062296
0.026561	0.506525	0.037948	0.035047	0.023278	0.045316	0.028627
0.219611	0	0.198727	0.269707	0.272768	0	0.324724
0.172581	0.77928	0.080934	0.068719	0.13525	0.289123	0.05305
0.051182	0.438748	0.075626	0.050177	0.396666	0.044779	0.296002
0.1147	0.768162	0.170769	0.176213	0.228094	0.102345	0.076077
0.08219	0.109772	0.125115	0.092423	0.075694	0.105768	0.07868
0.077477	0.290534	0.059748	0.120839	0.063445	0.170619	0.070953
0.32973	0.832617	0.476759	0.649694	0.242974	0.640475	0.068475
0.156308	0.684718	0.22757	0.216559	0.158504	0.101064	0.063052
0.429012	0.019293	0.040704	0.427317	0.019732	0.076925	0.016192
0.047491	0.774168	0.040611	0.038442	0.055193	0.045889	0.039413
0.185036	0.79586	0.248613	0.430269	0.17385	0.172355	0.090554
0.624138	0.024422	0.130096	0.60089	0.19567	0.23943	0.040624
0.019611	0.594803	0.014688	0.016293	0.0108	0.031209	0.011459
0.11243	0.693916	0.115168	0.095642	0.139638	0.085688	0.109569
0.356894	0.89841	0.136982	0.162231	0.25597	0.427554	0.027808
0.029462	0.619255	0.026618	0.031355	0.086314	0.040121	0.027537
0	0	0	0	0	0	0
0.070613	0.660576	0.059637	0.050274	0.05212	0.065499	0.056241
0.396037	0.31796	0.453753	0.406853	0.365152	0.166222	0.182783
0.025246	0.62062	0.039103	0.038966	0.023782	0.065032	0.025499
0.06741	0.65138	0.060513	0.070568	0.048296	0.093468	0.060215
0.739017	0.173571	0.49017	0.716753	0.325123	0.322639	0.843365
0.01304	0.016227	0.011338	0.011649	0.010223	0.014426	0.011474
0.198253	0.510415	0.228309	0.309938	0.197562	0.101977	0.063527
0.065788	0.564391	0.056119	0.058816	0.062022	0.046845	0.0498
0.644219	0.428027	0.541305	0.683923	0.496687	0.403122	0.509695
0.132189	0.085343	0.222822	0.145293	0.11413	0.072365	0.047999
0.049231	0.051529	0.057958	0.062171	0.034425	0.048989	0.047047
