# Supplementary material for: cg04448376, cg24387542, cg08548498, and cg14621323 as a Novel Signature to Predict Prognosis in Kidney Renal Papillary Cell Carcinoma
Source: Biomed Res Int. 2020 Dec 17;2020:4854390. doi: 10.1155/2020/4854390 (PMC7759405; doi:10.1155/2020/4854390)
Supplement: Supplementary Materials — Table S1: DMGs. Table S2: DEGs. Table S3: the 9 hub genes. Table S4: the methylated sites of 9 hub genes. Table S5: univariate Cox proportional hazards regression analysis (P < 0.05) of the methylated site data in the training dataset. Table S6: the signature risk score composed of 4 site combinations in the training and test dataset. Table S7: the expression of 4 methylated sites in GSE126441. Table S8: functional analysis of the selected 9 hub genes. Fig.S1: identification of the hub genes from DMGs and DEGs. The Venn diagram shows that there are nine hub genes in 79 DMGs and 5100 DEGs. The hub genes are opposite fold change. [file 4854390.f1.zip › Table S6.docx]

| **Table S6 The signature riskScore composed of 4 sites combinations in the training and test dataset.** | | | | | | | | | |
| --- | --- | --- | --- | --- | --- | --- | --- | --- | --- |
| **Training** | | | | | **Test** | | | |  |
| id | futime | fustat | riskScore | risk | id | futime | fustat | riskScore | risk |
| TCGA.DZ.6135.01A.11D.1963.05 | 6.216438 | 0 | 1.176541 | high | TCGA.BQ.7045.01A.31D.1963.05 | 4.021918 | 1 | 0.470276 | high |
| TCGA.SX.A7SP.01A.11D.A356.05 | 1.358904 | 1 | 0.595029 | high | TCGA.4A.A93X.01A.11D.A36Y.05 | 1.068493 | 0 | 0.788671 | high |
| TCGA.2Z.A9JK.01A.11D.A42K.05 | 3.249315 | 0 | 1.147037 | high | TCGA.5P.A9K3.01A.11D.A42K.05 | 1.290411 | 1 | 0.523801 | high |
| TCGA.BQ.7062.01A.11D.1963.05 | 0.317808 | 0 | 0.703882 | high | TCGA.B9.A44B.01A.11D.A254.05 | 0.989041 | 0 | 0.94544 | high |
| TCGA.BQ.5877.01A.11D.1590.05 | 0.739726 | 1 | 1.971624 | high | TCGA.J7.8537.01A.11D.2399.05 | 0.879452 | 1 | 2.113362 | high |
| TCGA.DZ.6132.01A.11D.1963.05 | 6.739726 | 0 | 1.264246 | high | TCGA.AL.7173.01A.11D.2137.05 | 5.643836 | 0 | 2.281309 | high |
| TCGA.UZ.A9PJ.01A.11D.A383.05 | 2.183562 | 1 | 0.678791 | high | TCGA.BQ.5888.01A.11D.1590.05 | 1.10411 | 0 | 1.344874 | high |
| TCGA.WN.AB4C.01A.11D.A42K.05 | 3.254795 | 0 | 0.811549 | high | TCGA.B1.A47O.01A.11D.A254.05 | 2.306849 | 0 | 1.312973 | high |
| TCGA.B9.A8YI.01A.21D.A36Y.05 | 1.024658 | 0 | 0.359058 | low | TCGA.UZ.A9Q0.01A.12D.A42K.05 | 9.227397 | 0 | 0.997806 | high |
| TCGA.5P.A9K4.01A.11D.A42K.05 | 5.983562 | 0 | 0.317997 | low | TCGA.PJ.A8JU.01A.11D.A369.05 | 1.041096 | 0 | 0.824524 | high |
| TCGA.UZ.A9PN.01A.11D.A383.05 | 3.112329 | 1 | 0.533997 | high | TCGA.BQ.5882.01A.11D.1590.05 | 1.80274 | 1 | 1.67829 | high |
| TCGA.J7.A8I2.01A.12D.A369.05 | 0.841096 | 0 | 0.484182 | high | TCGA.DW.7839.01A.11D.2137.05 | 1.147945 | 0 | 0.995287 | high |
| TCGA.HE.A5NJ.01A.11D.A26R.05 | 4.728767 | 0 | 0.894134 | high | TCGA.IA.A83W.01A.11D.A356.05 | 5.59726 | 1 | 0.769688 | high |
| TCGA.BQ.7048.01A.11D.1963.05 | 7.778082 | 0 | 1.144229 | high | TCGA.HE.7130.01A.11D.1963.05 | 4.849315 | 0 | 2.374865 | high |
| TCGA.BQ.5886.01A.11D.1590.05 | 2.169863 | 0 | 1.708067 | high | TCGA.BQ.5879.01A.11D.1590.05 | 1.909589 | 1 | 2.289391 | high |
| TCGA.5P.A9JU.01A.11D.A42K.05 | 1.021918 | 0 | 2.860393 | high | TCGA.GL.A9DD.01A.11D.A36Y.05 | 0.934247 | 0 | 0.97257 | high |
| TCGA.A4.7732.01A.11D.2137.05 | 1.594521 | 0 | -0.49186 | low | TCGA.P4.AAVO.01A.11D.A42K.05 | 6.575342 | 0 | 2.705056 | high |
| TCGA.J7.6720.01A.11D.2137.05 | 1.063014 | 0 | -0.19858 | low | TCGA.B1.A656.01A.11D.A31Y.05 | 1.693151 | 0 | 0.453657 | high |
| TCGA.BQ.7053.01A.11D.1963.05 | 4.153425 | 0 | 0.671225 | high | TCGA.SX.A7SL.01A.11D.A356.05 | 2.254795 | 0 | 1.143516 | high |
| TCGA.A4.A48D.01A.11D.A254.05 | 1.038356 | 0 | 0.446536 | low | TCGA.5P.A9KA.01A.11D.A42K.05 | 1.082192 | 0 | 0.843381 | high |
| TCGA.EV.5901.01A.11D.1590.05 | 2.849315 | 0 | 0.76266 | high | TCGA.Y8.A8S1.01A.11D.A36Y.05 | 1.106849 | 0 | 1.592326 | high |
| TCGA.A4.A4ZT.01A.11D.A26R.05 | 1.413699 | 0 | -0.65017 | low | TCGA.IA.A40Y.01A.11D.A254.05 | 0.169863 | 0 | 2.308271 | high |
| TCGA.G7.A8LC.01A.11D.A369.05 | 1.923288 | 0 | -0.68232 | low | TCGA.HE.A5NK.01A.11D.A26R.05 | 6.10411 | 0 | 0.635059 | high |
| TCGA.Y8.A8S0.01A.11D.A36Y.05 | 0.50137 | 0 | 0.22155 | low | TCGA.Q2.A5QZ.01A.11D.A28O.05 | 1.172603 | 0 | 2.96427 | high |
| TCGA.F9.A4JJ.01A.11D.A254.05 | 0.890411 | 1 | 2.197712 | high | TCGA.Y8.A8RZ.01A.11D.A36Y.05 | 0.306849 | 0 | 0.875698 | high |
| TCGA.BQ.5887.01A.11D.1963.05 | 2.112329 | 0 | 1.952632 | high | TCGA.BQ.7049.01A.11D.1963.05 | 3.736986 | 1 | 1.50948 | high |
| TCGA.2Z.A9JG.01A.11D.A42K.05 | 4.553425 | 0 | -1.48007 | low | TCGA.P4.AAVK.01A.11D.A42K.05 | 4.356164 | 0 | 0.986085 | high |
| TCGA.2Z.A9J9.01A.11D.A42K.05 | 6.284932 | 0 | 1.630005 | high | TCGA.GL.7773.01A.11D.2137.05 | 1.076712 | 0 | 1.322688 | high |
| TCGA.IA.A40U.01A.11D.A254.05 | 1.4 | 0 | -1.3728 | low | TCGA.GL.A4EM.01A.11D.A254.05 | 0.087671 | 0 | 1.128166 | high |
| TCGA.B9.5155.01A.01D.1590.05 | 1.821918 | 0 | -0.72508 | low | TCGA.G7.6796.01A.11D.1963.05 | 5.953425 | 0 | 0.569896 | high |
| TCGA.BQ.5878.01A.11D.1590.05 | 5.309589 | 0 | 1.232855 | high | TCGA.P4.A5EA.01A.11D.A28O.05 | 0.550685 | 1 | 2.342533 | high |
| TCGA.UZ.A9PU.01A.11D.A42K.05 | 2.556164 | 1 | 1.340914 | high | TCGA.P4.A5E8.01A.11D.A28O.05 | 3.578082 | 1 | 0.681213 | high |
| TCGA.B1.A655.01A.11D.A31Y.05 | 1.39726 | 0 | 0.538906 | high | TCGA.UZ.A9PL.01A.11D.A383.05 | 8.635616 | 0 | 1.014553 | high |
| TCGA.UZ.A9PS.05A.11D.A42K.05 | 5.936986 | 0 | 1.185681 | high | TCGA.B1.5398.01A.02D.1590.05 | 3.427397 | 0 | 1.960783 | high |
| TCGA.5P.A9KC.01A.11D.A42K.05 | 1.032877 | 0 | 2.125854 | high | TCGA.2Z.A9JO.01A.11D.A42K.05 | 2.6 | 0 | 1.107965 | high |
| TCGA.EV.5902.01A.11D.1590.05 | 1.583562 | 0 | -0.44328 | low | TCGA.BQ.5884.01A.11D.1590.05 | 2.169863 | 0 | 1.045337 | high |
| TCGA.MH.A55Z.01A.11D.A26R.05 | 2.315068 | 0 | -1.6151 | low | TCGA.Y8.A898.01A.11D.A356.05 | 1.30137 | 0 | 0.679542 | high |
| TCGA.BQ.5880.01A.11D.1590.05 | 3.6 | 1 | 1.946708 | high | TCGA.PJ.A5Z8.01A.11D.A28O.05 | 1.643836 | 0 | 1.050483 | high |
| TCGA.UZ.A9PV.01A.11D.A42K.05 | 3.956164 | 0 | -1.92718 | low | TCGA.HE.A5NF.01A.11D.A26R.05 | 7.073973 | 0 | 0.633802 | high |
| TCGA.B1.A47M.01A.11D.A254.05 | 1.805479 | 0 | 0.220508 | low | TCGA.2Z.A9J3.01A.12D.A383.05 | 4.852055 | 1 | 1.090693 | high |
| TCGA.2Z.A9JN.01A.21D.A42K.05 | 2.027397 | 0 | 1.462514 | high | TCGA.5P.A9K9.01A.11D.A42K.05 | 1.613699 | 1 | 2.662552 | high |
| TCGA.A4.A5Y0.01A.11D.A31Y.05 | 0.690411 | 0 | 2.207113 | high | TCGA.G7.A8LD.01A.11D.A369.05 | 1.408219 | 1 | 1.158734 | high |
| TCGA.WN.A9G9.01A.12D.A36Y.05 | 2.167123 | 0 | 0.397427 | low | TCGA.2Z.A9J7.01A.11D.A383.05 | 0.380822 | 1 | 0.660854 | high |
| TCGA.2Z.A9JI.01A.11D.A42K.05 | 4.293151 | 0 | 1.759946 | high | TCGA.PJ.A5Z9.01A.11D.A28O.05 | 1.479452 | 0 | -0.37462 | low |
| TCGA.5P.A9K0.01A.11D.A42K.05 | 1.854795 | 0 | -0.92619 | low | TCGA.BQ.5890.01A.11D.1590.05 | 0.638356 | 0 | 0.216431 | low |
| TCGA.HE.A5NH.01A.11D.A26R.05 | 2.167123 | 0 | -0.19991 | low | TCGA.UZ.A9PQ.01A.11D.A42K.05 | 7.189041 | 1 | -0.05825 | low |
| TCGA.DW.7841.01A.11D.2137.05 | 1.219178 | 0 | -0.57767 | low | TCGA.UZ.A9PO.01A.11D.A383.05 | 5.235616 | 0 | -0.42959 | low |
| TCGA.2Z.A9J6.01A.11D.A383.05 | 4.742466 | 0 | -1.43079 | low | TCGA.Y8.A894.01A.11D.A369.05 | 1.553425 | 0 | -1.85079 | low |
| TCGA.BQ.7058.01A.11D.1963.05 | 0.939726 | 1 | -0.15892 | low | TCGA.G7.6795.01A.11D.1963.05 | 3.271233 | 0 | -1.68996 | low |
| TCGA.2Z.A9JE.01A.11D.A42K.05 | 4.734247 | 0 | -1.4269 | low | TCGA.2Z.A9J5.01A.21D.A383.05 | 8.356164 | 0 | -0.00467 | low |
| TCGA.UZ.A9PZ.01A.11D.A42K.05 | 1.8 | 0 | -0.38646 | low | TCGA.Y8.A896.01A.11D.A369.05 | 1.553425 | 0 | -0.12842 | low |
| TCGA.2Z.A9J8.01A.11D.A42K.05 | 7.643836 | 0 | -1.52871 | low | TCGA.HE.A5NL.01A.11D.A26R.05 | 2.10137 | 0 | 0.391779 | low |
| TCGA.G7.7502.01A.11D.2202.05 | 2.153425 | 0 | 0.103139 | low | TCGA.GL.A59T.01A.21D.A28O.05 | 1.49589 | 0 | -1.29296 | low |
| TCGA.MH.A854.01A.11D.A356.05 | 1.468493 | 0 | 1.892709 | high | TCGA.SX.A7SR.01A.12D.A369.05 | 2.205479 | 0 | -1.27032 | low |
| TCGA.BQ.5885.01A.11D.1590.05 | 2.523288 | 0 | -0.00059 | low | TCGA.DW.7840.01A.11D.2137.05 | 0.312329 | 0 | -0.04699 | low |
| TCGA.IA.A83T.01A.11D.A356.05 | 7.353425 | 1 | 1.433224 | high | TCGA.5P.A9JZ.01A.11D.A42K.05 | 7.517808 | 0 | 0.10997 | low |
| TCGA.P4.AAVL.01A.11D.A42K.05 | 1.334247 | 1 | 0.697089 | high | TCGA.2Z.A9JT.01A.11D.A42K.05 | 1.873973 | 0 | -0.4956 | low |
| TCGA.2Z.A9JL.01A.11D.A42K.05 | 2.756164 | 0 | -0.09767 | low | TCGA.F9.A7VF.01A.11D.A341.05 | 0.2 | 0 | -0.07422 | low |
| TCGA.BQ.5892.01A.11D.1590.05 | 0.99726 | 0 | 0.477387 | high | TCGA.B9.5156.01A.01D.1590.05 | 3.665753 | 0 | 0.074245 | low |
| TCGA.B9.A5W8.01A.11D.A28O.05 | 1.435616 | 0 | 0.58691 | high | TCGA.KV.A74V.01A.11D.A341.05 | 0.950685 | 0 | -1.55286 | low |
| TCGA.GL.A9DE.01A.11D.A36Y.05 | 1.358904 | 0 | 0.228324 | low | TCGA.SX.A71W.01A.12D.A356.05 | 2.947945 | 0 | -1.30476 | low |
| TCGA.MH.A857.01A.11D.A356.05 | 2.438356 | 0 | 0.310842 | low | TCGA.A4.7996.01A.11D.2202.05 | 1.386301 | 0 | -0.08721 | low |
| TCGA.GL.A59R.01A.11D.A26R.05 | 1.035616 | 0 | -1.92742 | low | TCGA.B1.A47N.01A.11D.A254.05 | 3.041096 | 0 | -1.16719 | low |
| TCGA.5P.A9K8.01A.11D.A42K.05 | 3.586301 | 0 | 0.269831 | low | TCGA.A4.8311.01A.11D.2399.05 | 1.99726 | 0 | -0.94045 | low |
| TCGA.GL.8500.01A.11D.2399.05 | 2.30411 | 0 | -1.6635 | low | TCGA.B1.A654.01A.11D.A31Y.05 | 1.687671 | 0 | -2.12412 | low |
| TCGA.BQ.7046.01A.11D.1963.05 | 8.076712 | 0 | 0.200941 | low | TCGA.Y8.A8RY.01A.11D.A36Y.05 | 2.106849 | 0 | 0.380105 | low |
| TCGA.GL.7966.01A.11D.2202.05 | 0.306849 | 0 | 2.343091 | high | TCGA.O9.A75Z.01A.11D.A341.05 | 1.290411 | 0 | -0.29267 | low |
| TCGA.BQ.5893.01A.11D.1590.05 | 0.660274 | 1 | 1.8812 | high | TCGA.DW.5561.01A.01D.1590.05 | 1.134247 | 0 | -0.4301 | low |
| TCGA.2Z.A9JD.01A.11D.A42K.05 | 2.90411 | 0 | -0.40221 | low | TCGA.B1.7332.01A.11D.2137.05 | 3.126027 | 0 | -0.05988 | low |
| TCGA.IZ.A6M8.01A.11D.A31Y.05 | 1.361644 | 0 | 0.409943 | low | TCGA.A4.7997.01A.11D.2202.05 | 2.539726 | 0 | 0.351484 | low |
| TCGA.A4.7288.01A.11D.2137.05 | 5.087671 | 0 | -0.7086 | low | TCGA.IZ.8196.01A.11D.2399.05 | 1.778082 | 0 | -2.15555 | low |
| TCGA.A4.7287.01A.11D.2137.05 | 1.635616 | 1 | 2.086298 | high | TCGA.2Z.A9JQ.01A.11D.A42K.05 | 2.131507 | 0 | -1.88111 | low |
| TCGA.5P.A9JV.01A.12D.A42K.05 | 5.59726 | 0 | 1.302815 | high | TCGA.G7.A4TM.01A.11D.A31Y.05 | 2.10411 | 0 | 0.302886 | low |
| TCGA.DW.5560.01A.01D.1590.05 | 4.071233 | 0 | 0.356913 | low | TCGA.HE.7129.01A.11D.1963.05 | 6.827397 | 0 | -0.73595 | low |
| TCGA.SX.A7SQ.01A.12D.A369.05 | 3.468493 | 0 | -0.39126 | low | TCGA.P4.A5EB.01A.11D.A28O.05 | 8.260274 | 0 | -0.99375 | low |
| TCGA.G7.6797.01A.11D.1963.05 | 2.09589 | 0 | -2.20325 | low | TCGA.KV.A6GE.01A.11D.A31Y.05 | 2.424658 | 0 | -0.90067 | low |
| TCGA.P4.A5E6.01A.11D.A28O.05 | 8.315068 | 0 | -1.50727 | low | TCGA.DZ.6133.01A.11D.1963.05 | 4.265753 | 1 | -1.07189 | low |
| TCGA.BQ.7056.01A.11D.1963.05 | 1.676712 | 0 | 1.83395 | high | TCGA.BQ.7050.01A.11D.1963.05 | 5.106849 | 0 | 0.182167 | low |
| TCGA.5P.A9JW.01A.11D.A42K.05 | 8.879452 | 0 | 0.888254 | high | TCGA.MH.A55W.01A.11D.A26R.05 | 2.857534 | 0 | -1.51335 | low |
| TCGA.A4.8310.01A.11D.2399.05 | 2.136986 | 0 | -1.39009 | low | TCGA.SX.A71U.01A.12D.A341.05 | 3.717808 | 0 | 0.213396 | low |
| TCGA.A4.A5Y1.01A.11D.A28O.05 | 1.082192 | 1 | 0.310481 | low | TCGA.A4.A57E.01A.11D.A26R.05 | 0.706849 | 1 | -1.76688 | low |
| TCGA.B3.8121.01A.21D.2399.05 | 1.038356 | 0 | 1.080861 | high | TCGA.SX.A7SM.01A.11D.A356.05 | 0.994521 | 1 | -0.45075 | low |
| TCGA.SX.A7SN.01A.11D.A356.05 | 1.583562 | 0 | 0.242943 | low | TCGA.Y8.A897.01A.11D.A369.05 | 1.50411 | 0 | -0.14249 | low |
| TCGA.BQ.7051.01A.12D.1963.05 | 4.2 | 0 | -1.53503 | low | TCGA.B3.A6W5.01A.12D.A341.05 | 0.846575 | 0 | -1.91453 | low |
| TCGA.SX.A7SU.01A.11D.A369.05 | 3.646575 | 0 | 0.710808 | high | TCGA.A4.A5XZ.01A.11D.A31Y.05 | 1.312329 | 0 | -0.6942 | low |
| TCGA.SX.A71S.01A.11D.A341.05 | 4.120548 | 0 | -0.53287 | low | TCGA.2Z.A9JR.01A.12D.A42K.05 | 0.441096 | 0 | -0.55331 | low |
| TCGA.G7.6789.01A.11D.1963.05 | 0.334247 | 1 | 2.819485 | high | TCGA.B9.A5W7.01A.11D.A31Y.05 | 1.60274 | 0 | -1.90759 | low |
| TCGA.G7.6792.01A.21D.1963.05 | 7.575342 | 0 | 1.253345 | high |  |  |  |  |  |
| TCGA.AL.A5DJ.01A.11D.A26R.05 | 4.10411 | 1 | 1.720637 | high |  |  |  |  |  |
| TCGA.B9.A69E.01A.11D.A31Y.05 | 1.238356 | 0 | 1.839398 | high |  |  |  |  |  |
| TCGA.A4.7286.01A.11D.2137.05 | 1.517808 | 0 | -0.05965 | low |  |  |  |  |  |
| TCGA.A4.8518.01A.11D.2399.05 | 1.643836 | 0 | 1.713197 | high |  |  |  |  |  |
| TCGA.2Z.A9JS.01A.21D.A42K.05 | 1.347945 | 1 | 0.568437 | high |  |  |  |  |  |
| TCGA.P4.A5E7.01A.31D.A28O.05 | 7.257534 | 0 | 1.601614 | high |  |  |  |  |  |
| TCGA.A4.7585.01A.11D.2137.05 | 2.931507 | 1 | 1.91798 | high |  |  |  |  |  |
| TCGA.DW.7963.01B.11D.A28O.05 | 3.39726 | 0 | 0.670701 | high |  |  |  |  |  |
| TCGA.IA.A83V.01A.11D.A356.05 | 8.057534 | 1 | 1.376813 | high |  |  |  |  |  |
| TCGA.DW.7838.01A.11D.2137.05 | 2.038356 | 0 | 1.450146 | high |  |  |  |  |  |
| TCGA.2Z.A9J2.01A.11D.A383.05 | 4.917808 | 0 | 2.264942 | high |  |  |  |  |  |
| TCGA.A4.8515.01A.11D.2399.05 | 1.942466 | 0 | -1.64317 | low |  |  |  |  |  |
| TCGA.UZ.A9PS.01A.11D.A42K.05 | 5.936986 | 0 | 0.853323 | high |  |  |  |  |  |
| TCGA.Y8.A895.01A.11D.A369.05 | 1.326027 | 0 | -0.60813 | low |  |  |  |  |  |
| TCGA.A4.7828.01A.11D.2137.05 | 0.652055 | 0 | 2.072505 | high |  |  |  |  |  |
| TCGA.A4.7584.01A.11D.2137.05 | 1.780822 | 0 | -1.89743 | low |  |  |  |  |  |
| TCGA.DZ.6131.01A.11D.1963.05 | 6.654795 | 0 | 1.163438 | high |  |  |  |  |  |
| TCGA.SX.A7SS.01A.11D.A369.05 | 4.424658 | 0 | -2.52815 | low |  |  |  |  |  |
| TCGA.UZ.A9PK.01A.11D.A383.05 | 9.783562 | 0 | -0.23511 | low |  |  |  |  |  |
| TCGA.2K.A9WE.01A.11D.A383.05 | 0.586301 | 0 | 1.057512 | high |  |  |  |  |  |
| TCGA.2Z.A9JP.01A.11D.A42K.05 | 2.446575 | 0 | -0.80889 | low |  |  |  |  |  |
| TCGA.5P.A9K2.01A.11D.A42K.05 | 6.553425 | 0 | -1.01371 | low |  |  |  |  |  |
| TCGA.P4.A5ED.01A.11D.A28O.05 | 7.715068 | 0 | 2.062859 | high |  |  |  |  |  |
| TCGA.HE.A5NI.01A.11D.A26R.05 | 0.986301 | 0 | -1.06118 | low |  |  |  |  |  |
| TCGA.2Z.A9J1.01A.11D.A383.05 | 6.29589 | 0 | -0.09144 | low |  |  |  |  |  |
| TCGA.B1.A657.01A.11D.A31Y.05 | 1.756164 | 0 | -0.01006 | low |  |  |  |  |  |
| TCGA.DW.7837.01A.11D.2137.05 | 3.128767 | 0 | -1.01662 | low |  |  |  |  |  |
| TCGA.BQ.7059.01A.11D.1963.05 | 0.635616 | 0 | 1.084366 | high |  |  |  |  |  |
| TCGA.F9.A8NY.01A.11D.A369.05 | 0.09863 | 0 | 3.53848 | high |  |  |  |  |  |
| TCGA.G7.7501.01A.11D.2202.05 | 1.709589 | 1 | 2.063707 | high |  |  |  |  |  |
| TCGA.GL.A9DC.01A.11D.A36Y.05 | 1.073973 | 0 | 1.125453 | high |  |  |  |  |  |
| TCGA.KV.A6GD.01A.11D.A31Y.05 | 1.534247 | 0 | 1.500517 | high |  |  |  |  |  |
| TCGA.SX.A71R.01A.12D.A341.05 | 3.991781 | 0 | 1.538177 | high |  |  |  |  |  |
| TCGA.DW.7842.01A.11D.2137.05 | 0.252055 | 0 | 1.061898 | high |  |  |  |  |  |
| TCGA.DZ.6134.01A.11D.1963.05 | 5.084932 | 0 | 1.070264 | high |  |  |  |  |  |
| TCGA.UZ.A9PR.01A.11D.A42K.05 | 7.19726 | 0 | -0.54317 | low |  |  |  |  |  |
| TCGA.MH.A562.01A.11D.A26R.05 | 1.687671 | 0 | -0.61334 | low |  |  |  |  |  |
| TCGA.AT.A5NU.01A.11D.A28O.05 | 0.183562 | 0 | -1.66434 | low |  |  |  |  |  |
| TCGA.BQ.5883.01A.11D.1590.05 | 3.40274 | 0 | 0.902161 | high |  |  |  |  |  |
| TCGA.A4.A5DU.01A.11D.A28O.05 | 1.358904 | 0 | -0.85061 | low |  |  |  |  |  |
| TCGA.BQ.7060.01A.11D.1963.05 | 0.586301 | 0 | -0.24581 | low |  |  |  |  |  |
| TCGA.DW.7836.01A.11D.2137.05 | 2.994521 | 0 | -0.8646 | low |  |  |  |  |  |
| TCGA.UZ.A9Q1.01A.11D.A42K.05 | 4.279452 | 0 | 1.723602 | high |  |  |  |  |  |
| TCGA.SX.A71V.01A.11D.A341.05 | 2.4 | 0 | -1.07788 | low |  |  |  |  |  |
| TCGA.V9.A7HT.01A.11D.A341.05 | 2.643836 | 0 | 1.303017 | high |  |  |  |  |  |
| TCGA.MH.A855.01A.11D.A356.05 | 1.010959 | 0 | 0.341126 | low |  |  |  |  |  |
| TCGA.IZ.8195.01A.31D.2399.05 | 1.854795 | 0 | 1.057254 | high |  |  |  |  |  |
| TCGA.G7.6790.01A.11D.1963.05 | 4.315068 | 0 | -0.15946 | low |  |  |  |  |  |
| TCGA.G7.6793.01A.11D.1963.05 | 0.920548 | 1 | 4.325335 | high |  |  |  |  |  |
| TCGA.UN.AAZ9.01A.11D.A383.05 | 1.50137 | 0 | -0.5354 | low |  |  |  |  |  |
| TCGA.BQ.5894.01A.11D.1590.05 | 0.230137 | 0 | 2.128578 | high |  |  |  |  |  |
| TCGA.SX.A7SO.01A.11D.A356.05 | 3.443836 | 0 | 0.948675 | high |  |  |  |  |  |
| TCGA.EV.5903.01A.11D.1590.05 | 2.073973 | 0 | 1.233438 | high |  |  |  |  |  |
| TCGA.MH.A561.01A.11D.A26R.05 | 2.30411 | 0 | 0.30999 | low |  |  |  |  |  |
| TCGA.5P.A9KF.01A.11D.A42K.05 | 2.315068 | 0 | -1.24641 | low |  |  |  |  |  |
| TCGA.B9.7268.01A.11D.2137.05 | 1.838356 | 0 | -3.20302 | low |  |  |  |  |  |
| TCGA.A4.8098.01A.11D.2399.05 | 2.454795 | 0 | -1.46896 | low |  |  |  |  |  |
| TCGA.B9.A8YH.01A.11D.A36Y.05 | 1.457534 | 0 | -2.41232 | low |  |  |  |  |  |
| TCGA.5P.A9KE.01A.11D.A42K.05 | 2.257534 | 0 | 0.474666 | high |  |  |  |  |  |
| TCGA.5P.A9K6.01A.11D.A42K.05 | 5.243836 | 0 | -0.30864 | low |  |  |  |  |  |
| TCGA.BQ.5881.01A.11D.1590.05 | 3.983562 | 0 | 0.858115 | high |  |  |  |  |  |
| TCGA.2Z.A9JM.01A.12D.A42K.05 | 2.558904 | 0 | -1.03147 | low |  |  |  |  |  |
| TCGA.5P.A9JY.01A.11D.A42K.05 | 4.131507 | 1 | 0.701345 | high |  |  |  |  |  |
| TCGA.BQ.7061.01A.11D.1963.05 | 0.59726 | 0 | 1.976792 | high |  |  |  |  |  |
| TCGA.A4.8630.01A.11D.2399.05 | 1.194521 | 0 | -0.35065 | low |  |  |  |  |  |
| TCGA.5P.A9KH.01A.11D.A42K.05 | 5.619178 | 0 | 0.82735 | high |  |  |  |  |  |
| TCGA.UZ.A9PX.01A.11D.A42K.05 | 5.427397 | 0 | -0.67826 | low |  |  |  |  |  |
| TCGA.IA.A83S.01A.11D.A356.05 | 7.147945 | 1 | 1.132962 | high |  |  |  |  |  |
| TCGA.F9.A7Q0.01A.11D.A369.05 | 1.090411 | 0 | -0.38935 | low |  |  |  |  |  |
| TCGA.BQ.5876.01A.11D.1590.05 | 1.605479 | 0 | 0.451537 | high |  |  |  |  |  |
| TCGA.A4.8312.01A.11D.2399.05 | 1.939726 | 0 | 0.036665 | low |  |  |  |  |  |
| TCGA.IZ.A6M9.01A.11D.A31Y.05 | 1.030137 | 0 | -0.06424 | low |  |  |  |  |  |
| TCGA.2Z.A9JJ.01A.11D.A42K.05 | 3.323288 | 0 | 1.446761 | high |  |  |  |  |  |
| TCGA.A4.A6HP.01A.11D.A31Y.05 | 1.038356 | 0 | -0.96656 | low |  |  |  |  |  |
| TCGA.BQ.7055.01A.11D.1963.05 | 2.189041 | 0 | -0.63473 | low |  |  |  |  |  |
| TCGA.G7.A8LB.01A.11D.A369.05 | 1.49863 | 0 | -0.29167 | low |  |  |  |  |  |
| TCGA.MH.A856.01A.11D.A356.05 | 2.594521 | 0 | 1.117086 | high |  |  |  |  |  |
| TCGA.4A.A93Y.01A.11D.A36Y.05 | 0.964384 | 0 | 2.302545 | high |  |  |  |  |  |
| TCGA.UZ.A9PP.01A.11D.A42K.05 | 8.246575 | 0 | 1.253347 | high |  |  |  |  |  |
| TCGA.G7.A8LE.01A.11D.A369.05 | 6.720548 | 0 | -0.85149 | low |  |  |  |  |  |
| TCGA.MH.A560.01A.11D.A26R.05 | 2.531507 | 0 | -0.66085 | low |  |  |  |  |  |
| TCGA.P4.AAVM.01A.11D.A42K.05 | 4.216438 | 0 | -0.78309 | low |  |  |  |  |  |
| TCGA.BQ.5875.01A.11D.1590.05 | 7.230137 | 0 | 2.547843 | high |  |  |  |  |  |
| TCGA.BQ.5889.01A.11D.1590.05 | 0.90137 | 1 | 1.425342 | high |  |  |  |  |  |
| TCGA.IA.A40X.01A.11D.A254.05 | 1.665753 | 0 | 0.334858 | low |  |  |  |  |  |
| TCGA.A4.8517.01A.11D.2399.05 | 1.635616 | 0 | -0.96086 | low |  |  |  |  |  |
