# Supplementary material for: cg04448376, cg24387542, cg08548498, and cg14621323 as a Novel Signature to Predict Prognosis in Kidney Renal Papillary Cell Carcinoma
Source: Biomed Res Int. 2020 Dec 17;2020:4854390. doi: 10.1155/2020/4854390 (PMC7759405; doi:10.1155/2020/4854390)
Supplement: Supplementary Materials — Table S1: DMGs. Table S2: DEGs. Table S3: the 9 hub genes. Table S4: the methylated sites of 9 hub genes. Table S5: univariate Cox proportional hazards regression analysis (P < 0.05) of the methylated site data in the training dataset. Table S6: the signature risk score composed of 4 site combinations in the training and test dataset. Table S7: the expression of 4 methylated sites in GSE126441. Table S8: functional analysis of the selected 9 hub genes. Fig.S1: identification of the hub genes from DMGs and DEGs. The Venn diagram shows that there are nine hub genes in 79 DMGs and 5100 DEGs. The hub genes are opposite fold change. [file 4854390.f1.zip › Table S7.docx]

| **id** | **cg14621323** | **cg08548498** | **cg24387542** | **cg04448376** |
| --- | --- | --- | --- | --- |
| GSM3601887 | 0.294915421 | 0.680527383 | 0.35220464 | 0.160951009 |
| GSM3601895 | 0.374189804 | 0.683495719 | 0.313330094 | 0.108012487 |
| GSM3601897 | 0.215251264 | 0.753347943 | 0.351207838 | 0.327208062 |
| GSM3601901 | 0.205553773 | 0.714112218 | 0.363079897 | 0.107391674 |
| GSM3601913 | 0.284552619 | 0.664723661 | 0.398777742 | 0.146040675 |
| GSM3601915 | 0.335603355 | 0.723946785 | 0.37787069 | 0.114409168 |
| GSM3601917 | 0.306211642 | 0.872031662 | 0.348584522 | 0.125461255 |
| GSM3601930 | 0.297014021 | 0.787422167 | 0.268819241 | 0.158702846 |
| GSM3601937 | 0.307122788 | 0.50391353 | 0.345616127 | 0.121530208 |
| GSM3601940 | 0.356991343 | 0.742525174 | 0.375425497 | 0.189019608 |
| GSM3601933 | 0.440866908 | 0.437822165 | 0.168760779 | 0.273627363 |
| GSM3601934 | 0.53121861 | 0.514867485 | 0.300449838 | 0.254237288 |
| GSM3601935 | 0.441837595 | 0.517864154 | 0.191871058 | 0.360560093 |
| GSM3601936 | 0.381984816 | 0.444100085 | 0.159528156 | 0.491365777 |

**Table S7 the expression of 4 methylated sites in GSE126441**
